# Case Study of *N*‐^*i*^Pr versus *N*‐Mes Substituted NHC Ligands in Nickel Chemistry: The Coordination and Cyclotrimerization of Alkynes at [Ni(NHC)_2_]

**DOI:** 10.1002/chem.202103093

**Published:** 2021-11-16

**Authors:** Lukas Tendera, Moritz Helm, Mirjam J. Krahfuss, Maximilian W. Kuntze‐Fechner, Udo Radius

**Affiliations:** ^1^ Institut für Anorganische Chemie Julius-Maximilians-Universität Würzburg Am Hubland 97074 Würzburg Germany

**Keywords:** alkyne complexes, cyclooligomerization, cyclotrimerization, *N*-heterocyclic carbenes, nickel complexes

## Abstract

A case study on the effect of the employment of two different NHC ligands in complexes [Ni(NHC)_2_] (NHC=^*i*^Pr_2_Im^Me^ **1^Me^**, Mes_2_Im **2**) and their behavior towards alkynes is reported. The reaction of a mixture of [Ni_2_(^*i*^Pr_2_Im^Me^)_4_(*μ‐(η* ^*2*^ : *η^2^)*‐COD)] **B**/ [Ni(^*i*^Pr_2_Im^Me^)_2_(*η^4^‐*COD)] **B’** or [Ni(Mes_2_Im)_2_] **2**, respectively, with alkynes afforded complexes [Ni(NHC)_2_(η^2^‐alkyne)] (NHC=^*i*^Pr_2_Im^Me^: alkyne=MeC≡CMe **3**, H_7_C_3_C≡CC_3_H_7_ **4**, PhC≡CPh **5**, MeOOCC≡CCOOMe **6**, Me_3_SiC≡CSiMe_3_ **7**, PhC≡CMe **8**, HC≡CC_3_H_7_ **9**, HC≡CPh **10**, HC≡C(*p*‐Tol) **11**, HC≡C(4‐^*t*^Bu‐C_6_H_4_) **12**, HC≡CCOOMe **13**; NHC=Mes_2_Im: alkyne=MeC≡CMe **14**, MeOOCC≡CCOOMe **15**, PhC≡CMe **16**, HC≡C(4‐^*t*^Bu‐C_6_H_4_) **17**, HC≡CCOOMe **18**). Unusual rearrangement products **11 a** and **12 a** were identified for the complexes of the terminal alkynes HC≡C(*p*‐Tol) and HC≡C(4‐^*t*^Bu‐C_6_H_4_), **11** and **12**, which were formed by addition of a C−H bond of one of the NHC *N*‐^*i*^Pr methyl groups to the C≡C triple bond of the coordinated alkyne. Complex **2** catalyzes the cyclotrimerization of 2‐butyne, 4‐octyne, diphenylacetylene, dimethyl acetylendicarboxylate, 1‐pentyne, phenylacetylene and methyl propiolate at ambient conditions, whereas **1^Me^** is not a good catalyst. The reaction of **2** with 2‐butyne was monitored in some detail, which led to a mechanistic proposal for the cyclotrimerization at [Ni(NHC)_2_]. DFT calculations reveal that the differences between **1^M^** ^e^ and **2** for alkyne cyclotrimerization lie in the energy profile of the initiation steps, which is very shallow for **2**, and each step is associated with only a moderate energy change. The higher stability of **3** compared to **14** is attributed to a better electron transfer from the NHC to the metal to the alkyne ligand for the *N*‐alkyl substituted NHC, to enhanced Ni‐alkyne backbonding due to a smaller C_NHC_−Ni−C_NHC_ bite angle, and to less steric repulsion of the smaller NHC ^*i*^Pr_2_Im^Me^.

## Graphical abstract

**Large versus small**: The impact of two different NHC ligands in complexes [Ni(NHC)_2_] and the behavior of the complexes towards alkynes is presented. It demonstrates how small differences in the stereo‐electronic features of closely related ligands can significantly alter the reactivity pattern. The reaction of (suitable precursors of) both complexes with alkynes gave *η* ^2^‐(*C*,*C*)‐alkyne complexes [Ni(NHC)_2_](*η* ^2^‐alkyne)], although the number of complexes available for [Ni(Mes_2_Im)_2_] is limited to small alkynes and good acceptor alkynes.

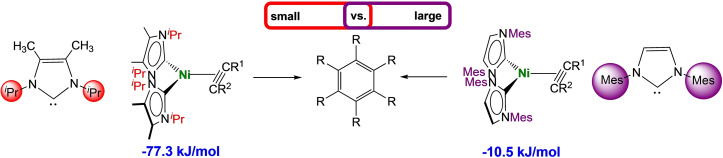

## Introduction

Transition‐metal‐catalyzed [2+2+2] cycloaddition reactions are elegant, atom‐efficient and group tolerant processes which involve the formation of several C−C bonds in a single step.^[1]^ These reactions offer the convenient access to a wide variety of carbocycles and heterocycles, mostly aromatic, starting from simple and inexpensive substrates.^[1]^ After Reppe et al. provided their pioneering report on the first cyclopolymerization of acetylene using a mixture of NiBr_2_ and CaC_2_ as the precatalyst,^[2]^ many different unsaturated substrates such as alkynes, diynes, alkenes, imines, isocyanates, isothiocyanates and CO_2_ were transformed in cycloaddition reactions to yield highly substituted derivatives of benzenes, pyridines, pyridones, pyrones, thiopyridones and cyclohexanes. Since then, catalytic systems such as NiBr_2_/dppe in the presence of Zn powder or [Ni(COD)_2_]‐based systems have been applied to many substrates.[^1b^, ^1c^, ^1d^, ^1e^, ^1f^, ^1g^, ^1h^, ^1i^, ^1j^, ^1k^, ^1l^, ^3^] Nickel complexes of *N*‐heterocyclic carbenes (NHCs) were also explored in cycloaddition reactions in the last two decades, mainly by Louie[^3a^, ^3b^] and Montgomery[^3c^, ^4^] and co‐workers. The Louie group commonly employed an *in situ* prepared catalyst system using [Ni(COD)_2_] as a nickel source and two equivalents of a sterically bulky and electron rich NHC ligand such as Dipp_2_Im (=1,3‐bis{2,6‐di‐*iso*‐propylphenyl}‐imidazolin‐2‐ylidene) or Dipp_2_Im^H2^ (=1,3‐bis{2,6‐di‐*iso*‐propylphenyl}‐imidazolidin‐2‐ylidene), that supposedly forms complexes of the type [Ni(NHC)_2_] or [Ni(NHC)] as the pre‐catalyst. These catalyst systems are highly efficient in the cyclization of different carbohydrates such as diynes or alkynes with ketones, aldehydes, nitriles, isocyanates and other substrates.[^3a^, ^3b^, ^5^] For example, the cycloaddition of alkynes or diynes with isocyanates to afford 2‐pyridones and pyrimidinediones is highly efficient and occurs with a high degree of chemoselectivity if a 1 : 1 mixture of [Ni(COD)_2_]/Dipp_2_Im^H2^ was used as catalyst.^[6]^ For this Ni/NHC‐catalyst system, alkyne cyclotrimerization was largely inhibited.^[6]^ However, differences in reactivity, yield, and selectivity have been observed in these Ni/NHC‐catalyzed cycloaddition reactions depending on the NHC ligand applied. The influence of the electronic and steric properties of the NHC ligand employed, for example Dipp_2_Im vs. Dipp_2_Im^H2^ vs. Mes_2_Im (=1,3‐dimesitylimidazolin‐2‐ylidene), to different cyclization reactions seems currently not to be completely understood.^[7]^ However, Montgomery et al. demonstrated that stereo‐electronic properties of NHC ligands play a crucial role for the regioselectivity observed for related nickel catalyzed allene hydrosilylation and reductive coupling reactions of aldehydes and alkynes.[^8^, ^9^] The regioselectivity of the latter is supposedly controlled by steric repulsion between the NHC ligand and the alkyne substituents in the first, rate determining oxidative addition step.^[9e]^

We reported earlier that complexes [Ni_2_(NHC)_4_(*μ‐(η* ^*2*^ : *η^2^)*‐COD)] of alkyl substituted NHCs such as ^*i*^Pr_2_Im (=1,3‐di‐*iso*‐propyl‐imidazolin‐2‐ylidene) or ^*n*^Pr_2_Im, which act as a source of [Ni(NHC)_2_], are efficient catalysts for the insertion of diphenyl acetylene into the C−C bond of biphenylene leading to 9,10‐di(phenyl)phenanthrene.^[10]^ The reaction rate of the formation of 9,10‐di(phenyl)phenanthrene depends on the steric demand of the NHC employed, giving the highest rates for the sterically most hindered NHC used. However, alkyne cyclooligomerization was suppressed at the reaction conditions employed (60–80 °C) for diphenyl acetylene, but excess of other alkynes (3‐hexyne or 2‐butyne) afforded traces of the cyclooligomerization product. As we are currently interested to evaluate the differences in the reactivity of complexes [Ni(NHC)_2_] of NHCs of different size,^[11]^ we decided to (re‐)evaluate the reactivity of complexes [Ni(NHC)_2_] with alkynes in some detail.

As all the work presented so far point to a decisive role of the sterics of the NHC ligand, we decided to reduce the steric demand of the *N*‐aryl substituted NHC on going from Dipp to Mes substituted NHC and to increase the steric demand of the *N*‐alkyl substituted NHC by backbone methylation. It has been demonstrated previously that backbone substitution at the C4 and C5 position of the imidazole framework, for example by methylation, greatly effects the stereo‐electronics of the NHC ligands as repulsion between the C4/C5 methyl group and the *N*‐organyl substituent leads to smaller C_carbene_‐N‐C_substituent_ angles.[^7^, ^12^] Thus, the NHCs we use for this study are Mes_2_Im and ^*i*^Pr_2_Im^Me^ (=1,3‐di‐*iso‐*propyl‐4,5‐dimethylimidazolin‐2‐ylidene).

## Results and Discussion

The reaction pathways and the results of key‐processes in transition metal chemistry and catalysis, such as oxidative addition, reductive elimination, migratory insertion, transmetalation, and β‐hydride elimination, depend decisively on the sterics of the (NHC) co‐ligands used and on the degree of electron transfer from the metal to the substrates and thus to the nature, sterics and number of co‐ligands.^[13]^ We recently investigated differences in the reactivity of the NHC‐stabilized nickel(0) complexes [Ni_2_(^*i*^Pr_2_Im)_4_(*μ‐(η* ^*2*^ : *η^2^)*‐COD)] **A** ^[10]^ as a source of [Ni(^*i*^Pr_2_Im)_2_] **1** and [Ni(Mes_2_Im)_2_] **2** in some detail.^[11]^ In course of our work on C−F bond activation and catalytic defluoroborylation of polyfluoroarenes using the complexes **A** ^[14]^ and **2**,^[15]^ we provided evidence from experiment and theory that, depending on the NHC ligand used, the insertion of [Ni(NHC)_2_] into the C−F bond of hexafluorobenzene proceeds via a concerted oxidative addition pathway for the small NHC ^*i*^Pr_2_Im and via a radical pathway for the more bulky NHC Mes_2_Im. Additionally, we found for both mechanisms a competitive NHC‐assisted reaction pathway which seems to be of general importance in transition metal NHC chemistry.^[11a]^ Furthermore, we provided a detailed study on the steric influence of NHCs of different size on the stabilization of nickel *π*‐complexes, since such complexes are very important intermediates in many different catalytic cycles.^[16]^ Therefore we investigated the reaction of [Ni_2_(^*i*^Pr_2_Im)_4_(*μ‐(η* ^*2*^ : *η^2^)*‐COD)] **A**, i.e., [Ni(^*i*^Pr_2_Im)_2_] **1**, and [Ni(Mes_2_Im)_2_] **2** with different olefines, aldehydes and ketones, which led to the formation of complexes of the type [Ni(NHC)_2_(*η* ^2^‐R_2_C=CR_2_)], [Ni(NHC)_2_(*η* ^2^‐O=CHR)] and [Ni(NHC)_2_(*η* ^2^‐O=CR_2_)]. Whereas **A** readily formed alkene complexes with olefins of different size, complex **2** reacted only with the smallest olefin ethylene or with activated acceptor olefins such as acrylates. Thus, the NHC nitrogen substituent influences the reactivity for steric reasons. However, these studies also pointed at the fact that substrate binding and electron transfer in bis‐NHC nickel complexes can be fine‐tuned very well beyond the accessibility of the metal center by steric protection and complex stability with respect to co‐ligand or NHC dissociation. A subtle influence of sterics to the electronic behavior of [Ni(NHC)_2_] lies in the C_NHC_−M−C_NHC_ bite‐angle the NHC ligands will adopt in the final product and in the propensity of the complexes [Ni(NHC)_2_] to get involved into radical electron transfer processes.^[17]^ Herein we want to expand this study on the reactivity of NHC‐stabilized nickel complexes towards simple alkynes using [Ni(Mes_2_Im)_2_] **2** and suitable sources of [Ni(^*i*^Pr_2_Im^Me^)_2_] **1^Me^**. As mentioned above, we reported some alkyne complexes [Ni(^*i*^Pr_2_Im)_2_(*η* ^2^‐R−C≡C−R‘) starting from [Ni(^*i*^Pr_2_Im)_2_] **1**, earlier,[^10^, ^18^] which were included in this study if appropriate.

**Scheme 1:**
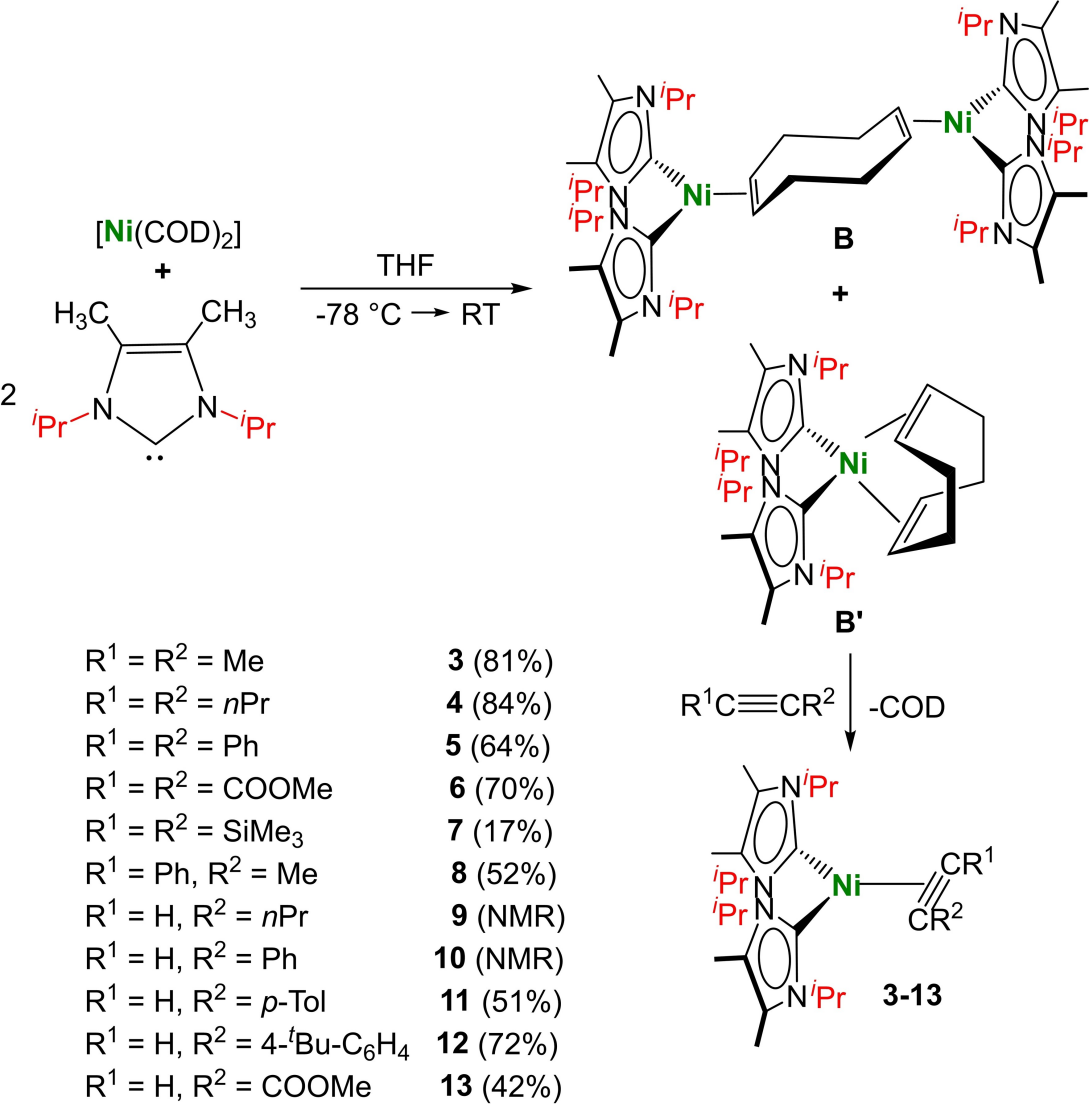
Synthesis of [Ni_2_(^*i*^Pr_2_Im^Me^)_4_(*μ‐(η* ^*2*^ : *η^2^)*‐COD)] **B** and [Ni(^*i*^Pr_2_Im^Me^)_2_(*η^4^‐*COD)] **B’** and the reaction of the mixture with alkynes to yield the complexes [Ni(^*i*^Pr_2_Im^Me^)_2_(*η* ^2^‐MeC≡CMe)] **3**, [Ni(^*i*^Pr_2_Im^Me^)_2_(*η* ^2^‐H_7_C_3_C≡CC_3_H_7_)] **4**, [Ni(^*i*^Pr_2_Im^Me^)_2_(*η* ^2^‐PhC≡CPh)] **5**, [Ni(^*i*^Pr_2_Im^Me^)_2_(*η* ^2^‐MeOOCC≡CCOOMe)] **6**, [Ni(^*i*^Pr_2_Im^Me^)_2_(*η* ^2^‐Me_3_SiC≡CSiMe_3_)] **7**, [Ni(^*i*^Pr_2_Im^Me^)_2_(*η* ^2^‐PhC≡CMe)] **8**, [Ni(^*i*^Pr_2_Im^Me^)_2_(*η* ^2^‐HC≡CC_3_H_7_)] **9**, [Ni(^*i*^Pr_2_Im^Me^)_2_(*η* ^2^‐HC≡CPh)] **10**, [Ni(^*i*^Pr_2_Im^Me^)_2_(*η* ^2^‐HC≡C(*p*‐Tol))] **11**, [Ni(^*i*^Pr_2_Im^Me^)_2_(*η* ^2^‐HC≡C(4‐^*t*^Bu‐C_6_H_4_))] **12** and [Ni(^*i*^Pr_2_Im^Me^)_2_(*η* ^2^‐HC≡CCOOMe)] **13**.

The complex [Ni_2_(^*i*^Pr_2_Im^Me^)_4_(*μ‐(η* ^*2*^ : *η^2^)*‐COD)] **B** of the backbone methylated NHC ^*i*^Pr_2_Im^Me^ was synthesized – as reported for **A** – from the reaction of [Ni(COD)_2_] with two equivalents of ^*i*^Pr_2_Im^Me^ (Scheme 1). As observed for **A**, the yellow solid obtained consists of two complexes, the dinuclear reaction product **B** and the mononuclear complex [Ni(^*i*^Pr_2_Im^Me^)_2_(*η^4^‐*COD)] **B’** as a by‐product in various amounts (up to approximately 40 %). As **B** and **B’** typically show identical reactivity with respect to alkynes (the same was observed previously for **A** and its mononuclear counterpart [Ni(^*i*^Pr_2_Im)_2_(*η^4^‐*COD)]), we did not further purify the mixture for the following reactions.

**Figure 1:**
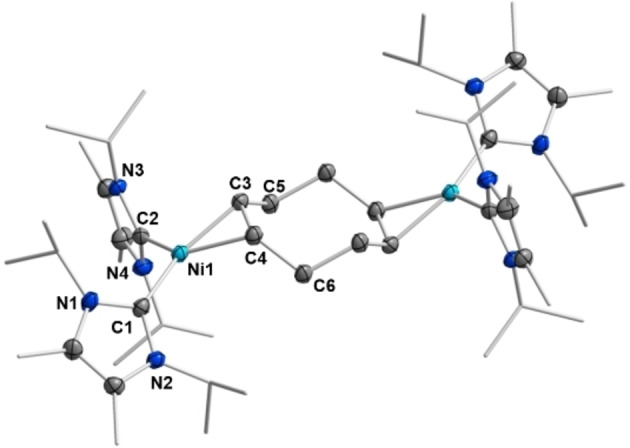
Molecular structure of [Ni_2_(^*i*^Pr_2_Im^Me^)_4_(*μ‐(η* ^*2*^ : *η^2^)*‐COD)] **B** in the solid state (ellipsoids were set at the 50 % probability level). The hydrogen atoms were omitted for clarity. Selected bond lengths [Å] and angles [°] of **B**: Ni1−C1 1.9117(19), Ni1−C2 1.9122(19), Ni1−C3 1.9749(19), Ni1−C4 1.9734(19), C3−C4 1.428(2), C3−C5 1.515(3), C4−C6 1.513(3); C1−Ni1−C2 118.65(8), C1−Ni1−C3 138.56(8), C1−Ni1−C4 96.15(8), C2−Ni1−C3 102.72(8), C2−Ni1−C4 145.08(8), C3−Ni1−C4 42.42(7).

Dinuclear **B** and mononuclear **B’** can be distinguished easily in their ^1^H and ^13^C{^1^H} NMR spectra. The resonances of the NHC ligand of **B** were detected as a broad doublet at 1.42 ppm for the *iso*‐propyl methyl protons, a singlet at 1.88 ppm for the backbone methyl protons and a septet at 6.03 ppm for the *iso*‐propyl methine protons, whereas sharp resonances were found for the NHC ligand of complex **B’** at 1.33 ppm (d), 1.86 ppm (s) and 5.90 ppm (sept.). In the ^13^C{^1^H} NMR spectra the resonances for the carbene carbon atoms were detected in close proximity at 206.5 ppm (**B**) and 205.4 ppm (**B’**). Complex **B** was structurally characterized (Figure 1), it adopts in the solid state a distorted pseudo‐square planar geometry at both nickel atoms. The complex is isostructural to [Ni_2_(^*i*^Pr_2_Im)_4_(*μ‐(η* ^*2*^ : *η^2^)*‐COD)] **A**,^[14a]^ and both complexes have almost identical Ni−C_carbene_ distances (**B**: 1.9117(19) Å and 1.9122(19) Å; **A**: 1.906(3) Å and 1.904(3) Å) and similar C_carbene_−Ni−C_carbene_ angles (**B**: 138.56(8)°; **A**: 142.55(14)°).

The reaction of a mixture of [Ni_2_(^*i*^Pr_2_Im^Me^)_4_(*μ‐(η* ^*2*^ : *η^2^)*‐COD)] **B** and [Ni(^*i*^Pr_2_Im^Me^)_2_(*η^4^‐*COD)] **B’** with equimolar amounts of 2‐butyne, 4‐octyne, diphenylacetylene, dimethyl acetylendicarboxylate, bis(trimethylsilyl)acetylene, 1‐phenyl‐1‐propyne, 1‐pentyne, phenylacetylene, *p*‐tolylacetylene, 4‐(*tert*‐butyl)phenylacetylene and methyl propiolate selectively afforded the corresponding *η* ^2^‐(*C*,*C*)‐alkyne complexes [Ni(^*i*^Pr_2_Im^Me^)_2_(*η* ^2^‐MeC≡CMe)] **3**, [Ni(^*i*^Pr_2_Im^Me^)_2_(*η* ^2^‐H_7_C_3_C≡CC_3_H_7_)] **4**, [Ni(^*i*^Pr_2_Im^Me^)_2_(*η* ^2^‐PhC≡CPh)] **5**, [Ni(^*i*^Pr_2_Im^Me^)_2_(*η* ^2^‐MeOOCC≡CCOOMe)] **6**, [Ni(^*i*^Pr_2_Im^Me^)_2_(*η* ^2^‐Me_3_SiC≡CSiMe_3_)] **7**, [Ni(^*i*^Pr_2_Im^Me^)_2_(*η* ^2^‐PhC≡CMe)] **8**, [Ni(^*i*^Pr_2_Im^Me^)_2_(*η* ^2^‐HC≡CC_3_H_7_)] **9**, [Ni(^*i*^Pr_2_Im^Me^)_2_(*η* ^2^‐HC≡CPh)] **10**, [Ni(^*i*^Pr_2_Im^Me^)_2_(*η* ^2^‐HC≡C(*p*‐Tol))] **11**, [Ni(^*i*^Pr_2_Im^Me^)_2_(*η* ^2^‐HC≡C(4‐^*t*^Bu‐C_6_H_4_))] **12**, and [Ni(^*i*^Pr_2_Im^Me^)_2_(*η* ^2^‐HC≡CCOOMe)] **13** (Scheme 1).

The complexes **3**–**13** were isolated as yellow or orange‐red, air and moisture sensitive powders and were characterized using ^1^H NMR, ^13^C{^1^H} NMR and IR spectroscopy (see Supporting Information). The complexes were obtained as analytically pure material except for the complexes of the terminal alkynes 1‐pentyne and phenylacetylene, [Ni(^*i*^Pr_2_Im^Me^)_2_(*η* ^2^‐HC≡CC_3_H_7_)] **9** and [Ni(^*i*^Pr_2_Im^Me^)_2_(*η* ^2^‐HC≡CPh)] **10**, which are only stable in solution and decompose upon removal of the solvent. The reactions of **B**/**B’** with alkynes proceeded in quantitative yield if performed on NMR scale; the yield of isolated **7**, however, is rather low due to losses in the crystallization process to get analytically pure material. Important ^1^H and ^13^C{^1^H} NMR data of the compounds **3**–**13** are summarized in Table 1. In the ^1^H NMR and ^13^C{^1^H} NMR spectra the signals for the NHC ligands were observed in the typical regions expected, and for the complexes **8**–**13** of unsymmetrical or terminal alkynes the set of NHC resonances is doubled due to a lowering of the complexes’ symmetry. Each alkyne proton of **9**–**13** is shifted upon coordination to nickel by 4.87–5.48 ppm to lower fields compared to the uncoordinated alkyne and was observed as a singlet in the range between 6.71 and 7.64 ppm. Strong backbonding from the metal atom to the ligand is also reflected in the ^13^C{^1^H} NMR spectra of these complexes as a significant low‐field coordination shift of 41.7–61.9 ppm occurs upon complexation.[^10^, ^11b^] The observed IR stretching vibrations of the alkyne triple bonds (1659–1785 cm^−1^) in the complexes **3**–**13** are also significantly shifted to lower wavenumbers compared to the uncoordinated alkynes, which show typical stretching vibrations between 2100 cm^−1^ and 2310 cm^−1^, and thus reflect a lower bond order upon coordination to nickel.^[19]^ The *ν*_C≡C_ coordination shift (▵*ν*_C≡C_) of complex **5** (1754 cm^−1^), for example, is −469 cm^−1^ compared to uncoordinated diphenylacetylene (2223 cm^−1^) and much larger compared to ▵*ν*_C≡C_ reported for the corresponding phosphine complex [(PPh_3_)_2_Ni(*η* ^2^‐PhC≡CPh)] (−419 cm^−1^).^[20]^ Thus, these complexes may rather be described as metallacyclopropenes, according to the Dewar‐Chatt‐Duncanson model.^[21]^

**Table 1:** ^13^C{^1^H} NMR and ^1^H NMR shifts [ppm] of the alkyne carbon and terminal alkyne hydrogen atoms as well as IR C≡C stretching vibrations [cm^−1^] of the complexes **3**–**13** (*δ*_C_=^13^C{^1^H} NMR shift of the alkyne carbon atoms; ▵*δ*_C_=^13^C{^1^H} coordination shift of the alkyne carbon atoms; *δ*_H_=^1^H NMR shift of the terminal alkyne hydrogen atoms; ▵*δ*_H_=^1^H coordination shift of the terminal alkyne hydrogen atoms; *δ*_C__NHC_=^13^C{^1^H} NMR shift of the NHC carbene carbon atoms, *ν*_C≡C_=IR stretching vibration of the alkyne triple bond).[^20b^, ^22^]

Compound	*δ*_C_	▵*δ*_C_	*δ*_H_	▵*δ*_H_	*δ*_C__NHC_	*ν*_C≡C_
3	121.6	47.2			205.1	1785
4	126.4	46.2			205.5	1778
5	139.2	49.1	–	–	201.7	1754
6	136.8	61.9	–	–	194.3	1749
7	159.8	47.3	–	–	205.1	1659
8	127.1	47.3	–	–	203.3	1760
	137.2	51.4				
9	111.7	43.4	6.71	4.94	204.2	
	138.1	53.6			204.8	
10	125.3	41.7	7.64	4.92	202.3	
	127.9	50.7			202.5	
11	123.9	46.9	7.61	4.87	202.6	1687
	138.1	54.1			202.9	
12	123.9	46.9	7.62	4.87	202.6	1683
	138.0	54.0			202.9	
13	129.6	53.6	7.64	5.48	198.6	1702
	131.9	56.9			198.8

**Figure 2:**
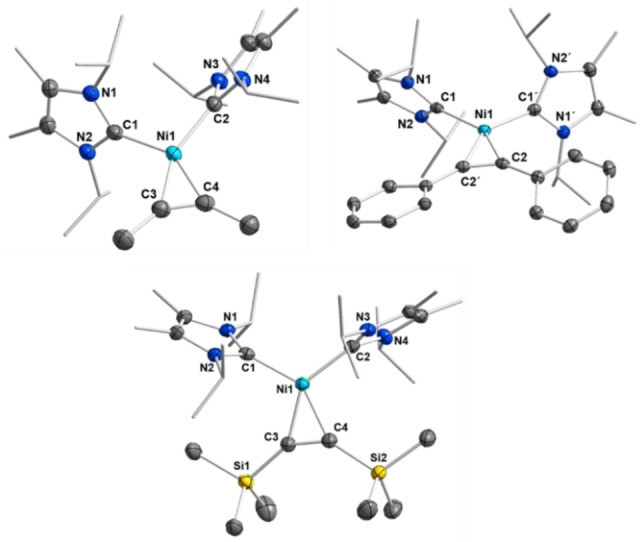
Molecular structures of [Ni(^*i*^Pr_2_Im^Me^)_2_(*η* ^2^‐MeC≡CMe)] **3** (top left), [Ni(^*i*^Pr_2_Im^Me^)_2_(*η* ^2^‐PhC≡CPh)] **5** (top right) and [Ni(^*i*^Pr_2_Im^Me^)_2_(*η* ^2^‐Me_3_SiC≡CSiMe_3_)] **7** (bottom) in the solid state (ellipsoids set at the 50 % probability level). The hydrogen atoms were omitted for clarity.

Crystals of [Ni(^*i*^Pr_2_Im^Me^)_2_(*η* ^2^‐MeC≡CMe)] **3**, [Ni(^*i*^Pr_2_Im^Me^)_2_(*η* ^2^‐PhC≡CPh)] **5** and [Ni(^*i*^Pr_2_Im^Me^)_2_(*η* ^2^‐Me_3_SiC≡CSiMe_3_)] **7** suitable for X‐ray diffraction were obtained from saturated hexane or pentane solutions at −30 °C (Figure 2, Table 4, for selected bond lengths and angles see the Supporting Information Figures S2–S4). Each of the complexes adopt a distorted pseudo‐square planar geometry, spanned by the two NHCs and the alkyne ligand. The Ni−C_NHC_ distances lie in the range between 1.9097(14) and 1.9251(13) Å and are thus in line with Ni−C_NHC_ distances reported previously for [Ni(Me^*i*^PrIm)_2_(*η* ^2^‐PhC≡CPh)] **C** (1.896(6)/1.915(4) Å) and [Ni(^*i*^Pr_2_Im)_2_(*η* ^2^‐MeC≡CMe)] **D** (1.917(8)/1.934(7) Å).^[10]^ The distances from nickel to the alkyne carbon atoms (Ni−C_alkyne_: 1.8804(14)–1.9047(16) Å) are slightly shorter than the Ni−C_NHC_ distances. The C≡C separation of the alkyne ligands (1.285(2) Å–1.304(3) Å; **C**: 1.310(6) Å, **D**: 1.286(13) Å) are remarkably enlarged compared to the uncoordinated alkynes.^[22]^ The alkyne ligands are slightly twisted out of the C_carbene_−Ni−C_carbene_ plane with twist angles between 7.90(8)° (**5**) and 9.27(12)° (**7**). This deviation from planarity is considerably larger compared to the values observed for **C** (1.76(19)°) and **D** (1.96(26)°) and we attribute this deviation to increased steric repulsion of the ligand ^*i*^Pr_2_Im^Me^ with methyl substituents in the backbone compared to ^*i*^Pr_2_Im and/or the Me^*i*^PrIm analogues.

**Scheme 2:**
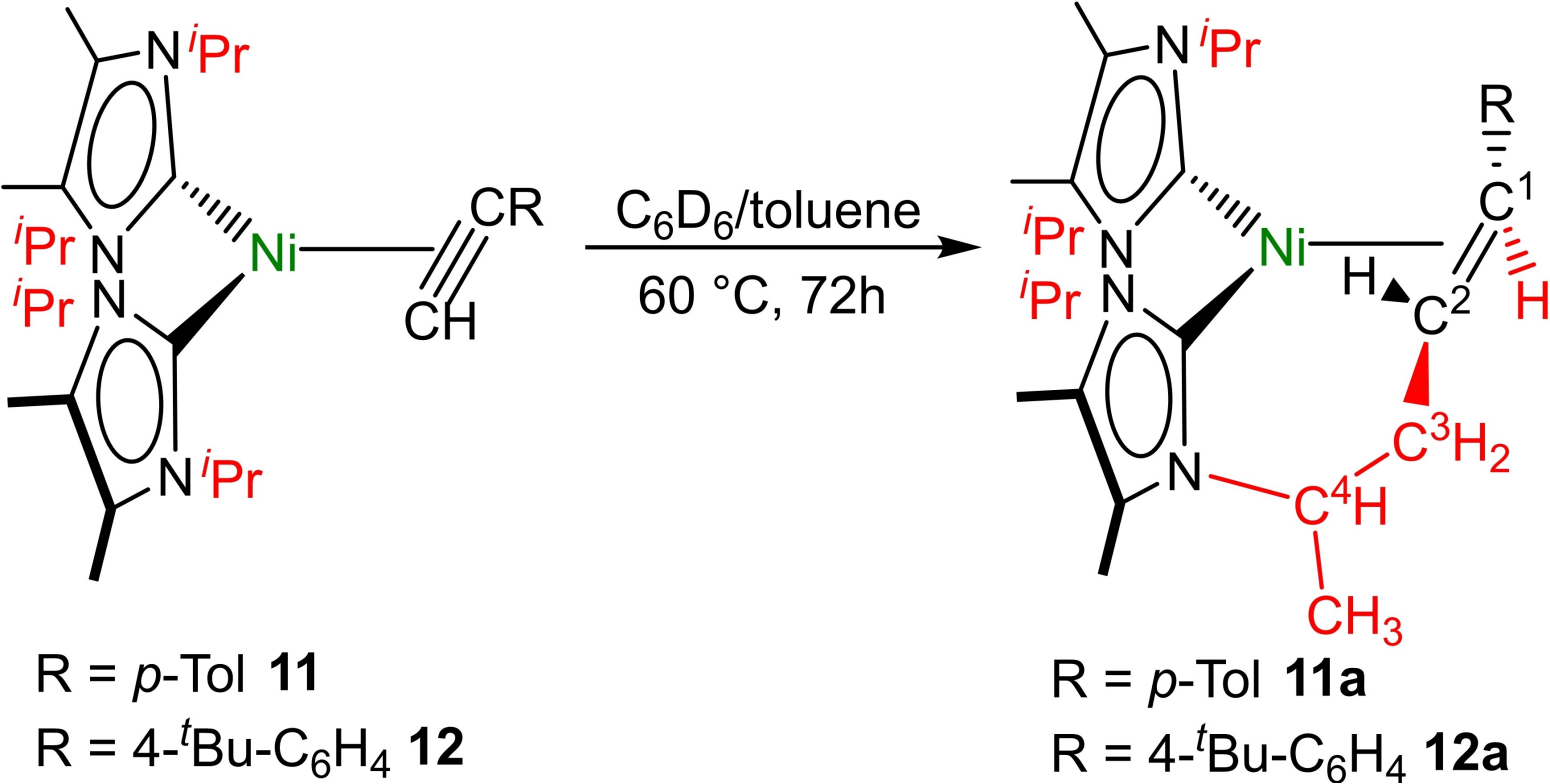
Synthesis of the decomposition products **11 a** and **12 a**.

**Figure 3:**
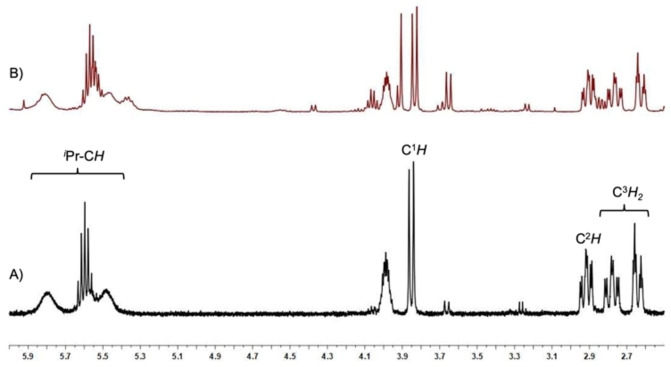
Part of the ^1^H NMR spectrum of compound **11 a** (A, bottom) and the *in situ* ^1^H NMR spectrum of the synthesis of compound **12 a** (B, top) in the range between 2.5 ppm and 6.0 ppm, showing the characteristic signals of the 6‐membered metallacycles formed.

Many of the complexes **3**–**13** are unstable upon heating and the result of thermal exposure in solution depends on the alkyne ligand coordinated. While [Ni(^*i*^Pr_2_Im^Me^)_2_(*η* ^2^‐PhC≡CPh)] **5** and [Ni(^*i*^Pr_2_Im^Me^)_2_(*η* ^2^‐MeOOCC≡CCOOMe)] **6** are stable in solution at 100 °C for days, complexes [Ni(^*i*^Pr_2_Im^Me^)_2_(*η* ^2^‐MeC≡CMe)] **3** and [Ni(^*i*^Pr_2_Im^Me^)_2_(*η* ^2^‐HC≡CPh)] **10** decompose already at room temperature, but much more rapidly upon heating with formation of so far unidentified products. Although we could not identify many of the decomposition products, for the thermal decomposition of the terminal alkyne complexes [Ni(^*i*^Pr_2_Im^Me^)_2_(*η* ^2^‐HC≡C(*p*‐Tol))] **11** and [Ni(^*i*^Pr_2_Im^Me^)_2_(*η* ^2^‐HC≡C(4‐^*t*^Bu‐C_6_H_4_))] **12** we characterized the rearrangement products **11 a** and **12 a** (Scheme 2 and Figure 3) after heating of benzene or toluene solutions of these complexes to 60 °C for 72 h. In addition to **11 a** or **12 a** other, so far unidentified side‐products were formed. However, the complexes **11 a** and **12 a** result from an interesting addition of a C−H bond of one of the NHC *N*‐*iso‐*propyl substituent methyl groups across the C≡C triple bond of the coordinated alkyne (Scheme 2).

We reported recently that NHC ligands are no good spectator ligands in cobalt NHC half sandwich alkyne chemistry and that they react in the coordination sphere of cobalt with terminal alkynes under coupling of the NHC and the alkyne ligand.^[23a]^ Related decomposition pathways involving coordinated alkynes and NHC ligands are also known.^[23]^ For the alkyne complexes of [Ni(NHC)_2_] we did not observe this kind of NHC alkyne coupling so far, but the complexes **11 a** and **12 a** were formed via an intramolecular C−C coupling reaction of the NHC *N*‐substituent. Formally, a hydrogen atom is transferred from the nearest *N*‐*iso‐*propyl methyl group of the NHC ligand to the coordinated alkyne carbon atom. The terminal alkyne carbon thus couples with the *iso*‐propyl methyl carbon with formation of a 6‐membered metallacycle and reduction of the C≡C triple bond to an *η* ^2^‐(*C*,*C*)‐coordinated alkene.

Red crystals of compound **11 a** were isolated for a complete characterization of this complex including X‐ray analysis, while **12 a** was only characterized *in situ* via the characteristic ^1^H NMR resonances in the NMR spectrum (see Figure 3, for the full NMR spectra see Supporting Information Figures S34–S40). In each case, the resonances of the olefinic protons of **11 a** and **12 a** were detected as a doublet at 3.85 ppm (C=C*H*R) for the proton at C^1^ (see Scheme 2 and Figure 3) and a doublet of doublets of doublets at 2.91 ppm for the proton at C^2^. The two diastereotopic protons of the CH_2_ group at C^3^ give rise to two separate resonances at 2.64 ppm (ddd) and 2.78 ppm (ddd), while the former *i*Pr methine proton was detected as a broad multiplet at 3.99 ppm. The three remaining *iso*‐propyl methine protons of the NHC ligands give rise to three partially overlapping and broadened septets in the range between 5.30 ppm and 5.90 ppm. In the ^13^C{^1^H} NMR spectrum of complex **11 a** the resonances of the olefinic carbon atoms are shifted towards higher fields compared to complex **11** and were detected at 34.1 ppm (C^2^) and 51.9 ppm (C^1^). The signals for the C^3^ carbon atom and the former *iso*‐propyl methine carbon C^4^ were observed at 40.2 and 54.1 ppm, respectively. The carbene carbon atom resonance of the NHC ligand involved in the metallacycle is also shifted to higher fields at 191.7 ppm, whereas the resonance of the unaffected NHC carbon atom was found at 204.5 ppm.

**Figure 4:**
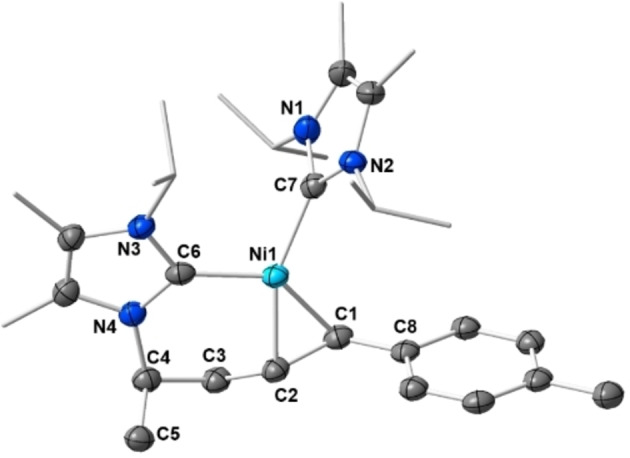
Molecular structure of **11 a** in the solid state (ellipsoids set at the 50 % probability level). The hydrogen atoms were omitted for clarity. Selected bond lengths [Å] and angles [°] of **11 a**: Ni1−C7 1.9140(15), Ni1−C6 1.9072(15), Ni1−C1 1.9945(14), Ni1−C2 1.9321(14), C1−C2 1.439(2), C1−C8 1.474(2), C2−C3 1.516(2), C3−C4 1.532(2), C4−C5 1.533(2); C6−Ni1−C7 109.53(6), C1−Ni1−C7 110.67(6), C1−Ni1−C2 42.96(6), C2−Ni1−C6 95.74(6), C1−C2−C3 121.19(13), C2−C1−C8 123.28(13), plane (C1−Ni1−C2) – plane (N1−C7−N2) 88.58(9), plane (C1−Ni1−C2) – plane (N3−C6−N4) 32.51(11), plane (N3−C6−N4) – plane (N1−C7−N2) 77.05(11).

Crystals of **11 a** suitable for X‐ray diffraction were obtained from storing a saturated solution of the complex in hexane at −30 °C (Figure 4). Complex **11 a** adopts a distorted pseudo‐square planar geometry in the solid state. The distance Ni1−C6 of 1.9072(15) Å and Ni1−C7 of 1.9140(15) Å to the NHC ligand carbon atoms are unexceptional and lie in the same range as observed for the alkyne complexes **3**, **5** and **7**. The distances of the nickel center to the olefin carbon atoms of 1.9945(14) Å (Ni1−C1) and 1.9321(14) Å (Ni1−C2) are larger compared to the Ni‐C_alkyne_ distances observed for the alkyne complexes, but in line with Ni‐C_olefin_ distances observed for **A** and **B** and related compounds. The C1−C2 separation of 1.439(2) Å is consistent with C=C bond lengths observed for other [Ni(NHC)_2_(*η* ^2^‐olefin)] complexes.^[11b]^ The nickel atom, the olefin carbon atoms C1, C2 and the NHC carbon atom C7 are perfectly aligned in a plane and the intact NHC ligand is nearly perfectly perpendicular oriented to this plane (88.58(9)°). The NHC ligand of the metallacycle (i.e., plane N3−C6−N4) is twisted towards the plane C1−Ni1−C2 with an angle of 32.51(11)°. The olefin adopts *trans*‐configuration with angles of 121.19(13)° (C1−C2−C3) and 123.28(13)° (C2−C1−C8) between the C=C‐bond vector and the substituents. The C2−C3 distance of the new bond between the olefin and the *iso*‐propyl carbon atom is 1.516(2) Å and thus clearly a single bond. The 6‐membered metallacycle adopts a distorted chair‐conformation.

**Scheme 3:**
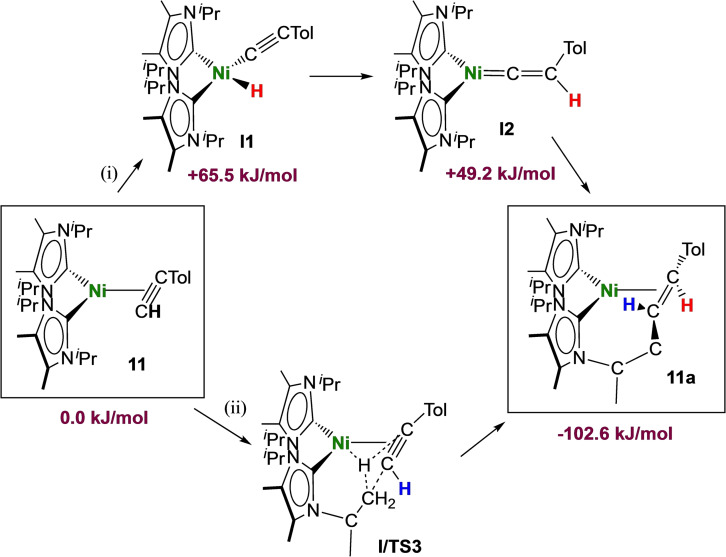
Pathways proposed for the formation of **11 a** via rearrangement of [Ni(^*i*^Pr_2_Im^Me^)_2_(*η* ^2^‐HC≡C(*p*‐Tol))] **11**. Results obtained from DFT calculations (BP86//def2‐TZVP(Ni)/def2‐SVP(C,N,H)) are included, given are ZPE corrected energies (maroon).

Scheme 3 sketches two reasonable reaction pathways for the rearrangement of [Ni(^*i*^Pr_2_Im^Me^)_2_(*η* ^2^‐HC≡C(*p*‐Tol))] **11** to product **11 a**. The first pathway (i) involves the rearrangement of the terminal alkyne ligand to a nickel vinylidene complex along the typical hydrido alkinyl route, which occurs with insertion of nickel into the C−H bond of the coordinated terminal alkyne ligand and subsequent hydride rearrangement to the *β*‐C atom.^[24]^ Insertion of the vinylidene into the NHC methyl C−H bond would lead then to complex **11 a**. Another likely pathway (ii) involves a concerted or nickel mediated addition of the NHC methyl C−H bond across the C≡C triple bond of the coordinated alkyne. DFT calculations (BP86//def2‐TZVP(Ni)/def2‐SVP(C,N,H)) reveal first of all that the rearrangement of **11** to yield **11 a** is a surprisingly strong exothermic process (▵E=−102.6 kJ/mol), and that the corresponding nickel hydrido alkinyl (+65.5 kJ/mol) and nickel vinylidene (+49.2 kJ/mol) complexes are significantly above the alkyne complex in energy, so that the barrier of process (i) is at least +65.5 kJ/mol. For the pathway (ii), we investigated either a concerted or a nickel mediated C−H addition to the coordinated alkyne. However, we were not able to locate any transition state here and every attempt to model likely nickel hydrido intermediates resulted in the ground state geometry of **11 a**. As DFT calculations gave no conclusive answer, we prepared complex **11** using deuterated p‐tolylacetylene and repeated the rearrangement with the resulting complex **11‐D**. As shown in Scheme 3, the deuterium label of **11‐D** should appear in the final product at different positions, depending on the pathway involved. The vinylidene pathway should lead to deuterium at the former *β*‐position of the coordinated alkyne (H atom marked in red in Scheme 3), the concerted/nickel‐mediated addition should lead to deuterium at the former *α*‐position of the coordinated alkyne (H atom marked in blue in Scheme 3). The result of the deuteration experiment revealed that the deuterium atom stays at the *α*‐carbon atom C^2^ (see Scheme 2 and Figure S40 of the Supporting Information) and therefore it is likely that the complexes **11 a** and **12 a** are formed according to a concerted or nickel‐mediated C−H bond activation pathway with addition of the NHC methyl C−H bond to the triple bond, in accordance with pathway (ii) of Scheme 3.

As it is known that [Ni(NHC)_2_] catalysts for cyclooligomerization reactions were prepared *in situ* from [Ni(COD)_2_] and a bulky and electron rich NHC ligand such as Dipp_2_Im, Dipp_2_Im^H2^ or Mes_2_Im,^[3b]^ we reacted isolated [Ni(Mes_2_Im)_2_] **2** with alkynes. Initial NMR experiments revealed that complex **2** cyclotrimerizes 2‐butyne quantitatively and therefore we investigated the catalytic activity and stereoselectivity of complex **2** in cyclotrimerization reactions using different internal and terminal alkynes (see Table 2). NMR spectra of the reactions of 2‐butyne, 4‐octyne, diphenylacetylene, dimethyl acetylendicarboxylate, 1‐pentyne, phenylacetylene and methyl propiolate with 5 mol% of [Ni(Mes_2_Im)_2_] **2** in C_6_D_6_ at 60 °C were recorded and the consumption of the alkynes was monitored. The catalyst was then removed by filtration over a pad of silica gel and the products were analyzed using ^1^H and ^13^C{^1^H} NMR spectroscopy as well as GC/MS. In all cases the cyclotrimerization of internal alkynes proceeded in quantitative yield on NMR scale (isolated yields were only determined for the preparation of hexaphenylbenzene, in this case the TON is 30) and no formation of side‐products was detected, with exception of the cyclotrimerization of 1‐pentyne, where traces of tetramerization products were observed (see Supporting Information). The reactions with terminal alkynes did not show any specific stereoselectivity and afforded mixtures of the 1,2,4‐ and 1,3,5‐stereoisomers. The exact determination of the product ratio via integration of the ^1^H NMR spectrum was only possible for the reaction of methyl propiolate due to overlapping NMR resonances for the products of the other alkynes. The use of internal alkynes yielded hexa‐substituted benzene derivatives, and the cyclotrimerization of diphenylacetylene to give hexaphenylbenzene proceeded much faster compared to the cyclotrimerization of other alkynes (entry 3, Table 2). This reaction was finished after five minutes at room temperature using a small catalyst load of just 1 mol%. As the product is almost insoluble in C_6_D_6_ it was isolated directly from the NMR tube as a colorless solid in 88 % yield.

**Table 2:** Scope of the catalytic cyclotrimerization of alkynes with [Ni(Mes_2_Im)_2_] **2**.

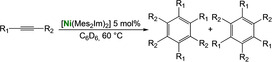			
Entry	Substrate	Products^[a]^	*t* [h]
1	2‐Butyne	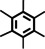	3
2	Phenylacetylene	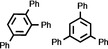	3
3	Diphenylacetylene	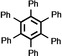	5 min
		88 %^[b,c]^	
4	1‐Pentyne	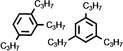	4
		+ traces of tetramerization	
5	4‐Octyne	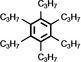	48
6	Methyl propiolate	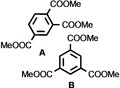	4
		(**A**: 85 %, **B**: 15 %)	
7	Dimethyl acetylenedicarboxylate	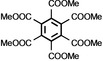	3

[a] Reaction conditions: [Ni(Mes_2_Im)_2_] **2** (5 mol%), alkyne (1.0 equiv.), C_6_D_6_ (0.6 mL), 60 °C, 20 h. Products after total consumption of the substrates, checked by NMR and GC/MS. Product ratios were determined by ^1^H NMR integration, if possible. [b] [Ni(Mes_2_Im)_2_] **2** (1 mol%), rt, 5 minutes. [c] Yield of isolated material after workup.

**Figure 5:**
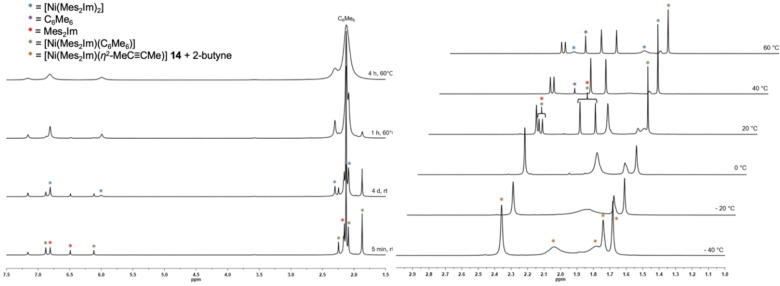
a) Time‐resolved ^1^H NMR spectrum of the reaction of [Ni(Mes_2_Im)_2_] **2** with 2‐butyne (4 equiv.; C_6_D_6_). b) Variable temperature ^1^H NMR spectrum of the reaction of [Ni(Mes_2_Im)_2_] **2** with 2‐butyne (4 equiv.; thf‐d_8_).

To gain further insight into the mechanistic details of the catalysis we analyzed the reaction of **2** with 2‐butyne. Therefore, we initially performed the reaction of **2** with a slight excess of 2‐butyne (4 equiv.) in a Young NMR tube (see Figure 5a). Addition of the alkyne led to an immediate color change from deep violet, which is the color of **2**, to bright yellow, which rapidly darkened after a few seconds. The analysis of the reaction mixture by NMR spectroscopy after five minutes at room temperature revealed the formation of hexamethylbenzene, free NHC Mes_2_Im and a new, well defined complex **E**. After 4 d at room temperature, some re‐formation of complex **2** was observed, resonances of free Mes_2_Im were still detectable and the signals assigned to complex **E** started to decrease. Finally, heating of the sample for 4 h at 60 °C led to a complete disappearance of the resonances for the NHC and for complex **E** and a full recovery of complex **2** plus the final cyclotrimerization product hexamethylbenzene was observed. The presence of uncoordinated carbene in the solution indicates that complex **E** might be a mono‐NHC complex [(Mes_2_Im)Ni(*η* ^6^‐C_6_Me_6_)] **E**, stabilized by hexamethylbenzene. A similar arene‐stabilized complex has been reported previously by Ogoshi et al.^[25]^ for a larger NHC, i.e., [Ni(Dipp_2_Im)(*η* ^6^‐C_6_H_5_Me)]. Despite of several attempts, we were not able to isolate this complex. Furthermore, the absence of 2‐butyne after five minutes at room temperature indicates that oligomerization proceeds very fast and quantitatively. To learn more details about this process, especially at which temperature the catalysis sets in, we additionally performed a variable temperature NMR experiment of the reaction from −40 °C to +60 °C in steps of 10 °C (see Figure 5b). At −40 °C, the reaction mixture had a bright yellow color and the NMR spectrum showed the formation of the alkyne complex [Ni(Mes_2_Im)_2_(*η* ^2^‐MeC≡CMe)] **14** (see below), similar as observed for complex **B/B’** with the smaller NHC ligand. Resonances of the trimerization product, free Mes_2_Im as well as the signals of complex **E** were already detected at temperatures of 0–10 °C. Integration of the resonances was consistent with the formation of a mono‐NHC arene complex [(Mes_2_Im)Ni(*η* ^6^‐C_6_Me_6_)]. After raising the temperature to 40 °C, the alkyne was completely consumed, the resonance of hexamethylbenzene increased and both, the NHC Mes_2_Im as well as the complex [(Mes_2_Im)Ni(*η* ^6^‐C_6_Me_6_)] **E**, were detected. Finally, at 60 °C, the recovery of complex **2** and the decrease of the resonances of the uncoordinated NHC and the mono‐NHC complex **E** occurred. We also performed the reaction of [Ni(^*i*^Pr_2_Im^Me^)_2_(*η* ^2^‐MeC≡CMe)] **3** with an excess of 2‐butyne, to see if **1^Me^** is also suitable for the catalytic trimerization. In contrast to complex **2** no cyclization was observed after 20 h at room temperature, but heating the reaction mixture to higher temperatures of 80 °C and above led to slow transformation of 2‐butyne to hexamethylbenzene.

**Scheme 4:**
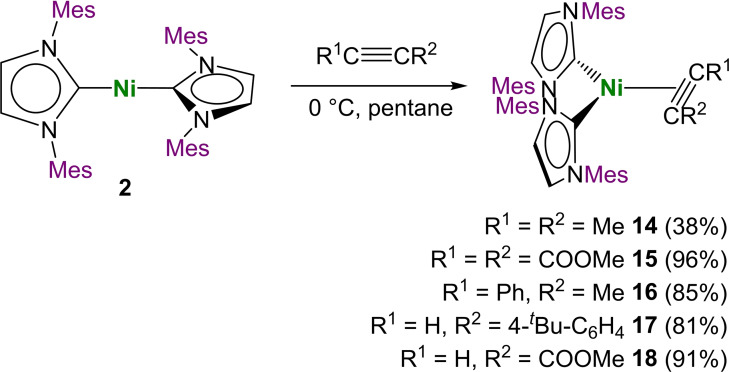
Synthesis of [Ni(Mes_2_Im)_2_(*η* ^2^‐MeC≡CMe)] **14**, [Ni(Mes_2_Im)_2_(*η* ^2^‐MeOOCC≡CCOOMe)] **15**, [Ni(Mes_2_Im)_2_(*η* ^2^‐PhC≡CMe)] **16**, [Ni(Mes_2_Im)_2_(*η* ^2^‐HC≡C(4‐^*t*^Bu‐C_6_H_4_))] **17** and [Ni(Mes_2_Im)_2_(*η* ^2^‐HC≡CCOOMe)] **18**.

We also tried to isolate some of the possible intermediates [Ni(Mes_2_Im)_2_(*η* ^2^‐R^1^C≡CR^2^)], [Ni(Mes_2_Im)(*η* ^2^‐R^1^C≡CR^2^)_2_] (for R^1^=R^2^=Me: **F**) or [(Mes_2_Im)Ni(*η* ^6^‐C_6_R_6_)] (for R=Me: **E**) of the catalysis from reactions of **2** with stoichiometric amounts, i.e., 1, 2, or 3 equivalents, of alkyne. However, all attempts to isolate complexes [Ni(Mes_2_Im)(*η* ^2^‐R^1^C≡CR^2^)_2_] and [(Mes_2_Im)Ni(*η* ^6^‐C_6_R_6_)] failed so far, but some complexes of the type [Ni(Mes_2_Im)_2_(*η* ^2^‐R^1^C≡CR^2^)] were obtained in pure form. The complexes with *η* ^2^‐(*C*,*C*)‐coordinated alkyne [Ni(Mes_2_Im)_2_(*η* ^2^‐MeC≡CMe)] **14**, [Ni(Mes_2_Im)_2_(*η* ^2^‐MeOOCC≡CCOOMe)] **15**, [Ni(Mes_2_Im)_2_(*η* ^2^‐PhC≡CMe)] **16**, [Ni(Mes_2_Im)_2_(*η* ^2^‐HC≡C(4‐^*t*^Bu‐C_6_H_4_))] **17** and [Ni(Mes_2_Im)_2_(*η* ^2^‐HC≡CCOOMe)] **18** precipitated as yellow to brown powders if the reactions were carried out at 0 °C in pentane or hexane, which made their isolation possible. These complexes are, once isolated, stable at room temperature in the solid state (see Scheme 4). The complexes **14** to **18** were fully characterized including elemental analysis and single crystal X‐ray structures for **14**, **15**, **16** and **17**. However, due to significant line broadening and signal overlap at room temperature or 0 °C, NMR spectroscopy of **14**, **16** and **17** was performed at −80 °C.

In general, the stability of complexes [Ni(Mes_2_Im)_2_(*η* ^2^‐R^1^C≡CR^2^)] depend on the steric demand of the alkyne used, but also on the electronic properties of the alkyne ligand. As observed previously for olefin complexes,^[11b]^ the steric bulk of the NHC ligand Mes_2_Im of complex **2** limits the coordination of a third ligand to the nickel atom, which is in stark contrast to the behavior of complexes **1/1^Me^**. Alkynes with electron withdrawing substituents increase *π*‐backbonding from the nickel atom to the alkyne and increase the stability of the alkyne complex in solution at room temperature. As noticed above, alkyl and/or aryl substituted alkynes lead to decomposition of the alkyne complexes with extrusion of one NHC ligand at temperatures slightly above 0 °C. Unlike the complexes **3**–**13**, the NMR spectra of the compounds **14**–**18** reveal remarkably broadened resonances for the bulkier NHC ligand Mes_2_Im due to hindered rotation, as it was previously reported by us for similar *π*‐complexes with ketone or aldehyde ligands.^[11b]^ Even the low temperature NMR spectra of **14**, **16** and **17** revealed some signal broadening. Nevertheless, all characteristic resonances were assigned and the integration of the resonances is consistent with the presence of one alkyne ligand per two NHC ligands in complexes of the type [Ni(Mes_2_Im)_2_(*η* ^2^‐alkyne)]. Important ^1^H and ^13^C{^1^H} NMR data of **14**–**18** are summarized in Table 3. In the ^1^H NMR spectra of **14**–**18** the *ortho* and *para* mesityl methyl protons give rise to up to four broadened resonances in the range between 1.74 and 2.37 ppm. The alkyne protons of the compounds **17** and **18** each can be observed as a singlet at 6.11 ppm (**17**) and 6.94 ppm (**18**). In the ^13^C{^1^H} NMR spectra the signals of the carbene carbon atoms were detected in the range between 198.2 and 207.0 ppm. The resonances of the alkyne carbon atoms are shifted to lower fields upon coordination and were observed in the range between 118.6 and 136.7 ppm. The *ν*_C≡C_ stretching vibrations of the complexes **14**–**18** are shifted to lower wavenumbers in the range between 1701 cm^−1^ and 1808 cm^−1^.

**Table 3:** ^13^C{^1^H} NMR and ^1^H NMR shifts [ppm] of the alkyne carbon and terminal alkyne hydrogen atoms as well as IR C≡C stretching vibrations [cm^−1^] of the complexes **14**–**18** (*δ*_C_=^13^C{^1^H} NMR shift of the alkyne carbon atoms; ▵*δ*_C_=^13^C{^1^H} coordination shift of the alkyne carbon atoms; *δ*_H_=^1^H NMR shift of the terminal alkyne hydrogen atoms; ▵*δ*_H_=^1^H coordination shift of the terminal alkyne hydrogen atoms; *δ*_C NHC_=^13^C{^1^H} NMR shift of the NHC carbene carbon atoms, *ν*_C≡C_=IR stretching vibration of the alkyne triple bond)[^20b^, ^22^].

Compound	*δ*_C_	▵*δ*_C_	*δ*_H_	▵*δ*_H_	*δ*_C NHC_	*ν*_C≡C_
**14**	118.6^[a]^	44.2			207.0^[a]^	1808
**15**	136.7	61.8			198.2	1713
**16**	123.9^[a]^	44.1	–	–	205.8^[a]^	1756
	135.6^[a]^	49.8			206.0^[a]^	
**17**	122.8^[a]^	45.8	6.11^[a]^	3.36	202.2^[a]^	1701
	131.5^[a^	47.5			206.5^[a]^	
**18**	134.6	58.6	6.94	4.78	201.8	1711
	136.6	61.6			202.4

[a] THF‐d_8_, −80 °C

**Figure 6:**
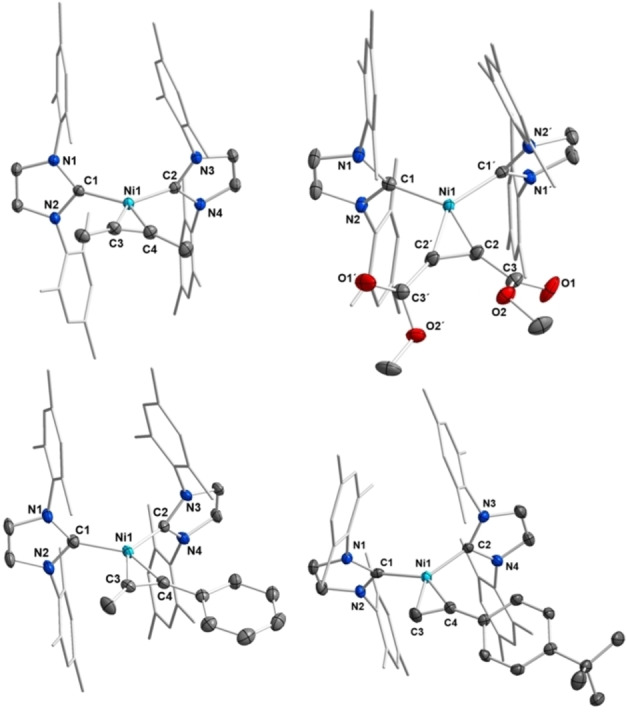
Molecular structures of [Ni(Mes_2_Im)_2_(*η* ^2^‐MeC≡CMe)] **14** (top left), [Ni(Mes_2_Im)_2_(*η* ^2^‐MeOOCC≡CCOOMe)] **15** (top right), [Ni(Mes_2_Im)_2_(*η* ^2^‐PhC≡CMe)] **16** (bottom left) and [Ni(Mes_2_Im)_2_(*η* ^2^‐HC≡C(4‐^*t*^Bu‐C_6_H_4_))] **17** (bottom right) in the solid state (ellipsoids set at 50 % probability level). The hydrogen atoms and a hexane molecule (17) were omitted for clarity.

Crystals suitable for X‐ray diffraction of **14**, **15**, **16** and **17** were obtained by either storing a saturated solution of the complex in hexane or pentane at −30 °C or by layering a saturated benzene solution of the complex with hexane at room temperature (**15**). The molecular structures of **14**, **15**, **16** and **17** are provided in Figure 6 (for selected bond lengths and angles see Supporting Information Figures S6‐S9). Important crystallographic data of these complexes and a comparison to the complexes [Ni(Me^*i*^PrIm)_2_(*η* ^2^‐PhC≡CPh)] **C**,^[10]^ [Ni(^*i*^Pr_2_Im)_2_(*η* ^2^‐MeC≡CMe)] **D**,^[10]^ [Ni(^*i*^Pr_2_Im^Me^)_2_(*η* ^2^‐MeC≡CMe)] **3**, [Ni(^*i*^Pr_2_Im^Me^)_2_(*η* ^2^‐PhC≡CPh)] **5** and [Ni(^*i*^Pr_2_Im^Me^)_2_(*η* ^2^‐Me_3_SiC≡CSiMe_3_)] **7** are given in Table 4. All complexes adopt a distorted pseudo‐square planar geometry, spanned by two NHCs and the alkyne ligand. All molecular structures reveal much larger C_NHC_−N−C_NHC_ bite angles of 122.24(6)° (**14**), 118.47(12)° (**15**), 118.5(2) (**16**) and 124.59(14)° (**17**) compared to the ^*i*^Pr_2_Im and ^*i*^Pr_2_Im^Me^ Ni complexes of the *N*‐alkyl substituted carbenes (**C**: 109.27(19)°, **D**: 100.4(3)°,^[10]^ **3**: 102.42(6)°, **5**: 110.66(8)°, **7**: 114.54(6)°), which is associated with the increased steric demand of the bulkier NHC Mes_2_Im. The C−C distances of the alkyne ligands of the complexes **14** (1.280(2) Å) and **17** (1.277(5) Å) are slightly shorter compared to the complexes with the small carbenes (1.285(2) Å (**3**)–1.310(6) Å (**C**)), which is consistent with decreased *π*‐backbonding.

**Table 4:** Comparison of important bond lengths and bond angles of [Ni(Me^*i*^PrIm)_2_(*η* ^2^‐PhC≡CPh)] **C**,^[10]^ [Ni(^*i*^Pr_2_Im)_2_(*η* ^2^‐MeC≡CMe)] **D**,^[10]^ [Ni(^*i*^Pr_2_Im^Me^)_2_(*η* ^2^‐MeC≡CMe)] **3**, [Ni(^*i*^Pr_2_Im^Me^)_2_(*η* ^2^‐PhC≡CPh)] **5**, [Ni(^*i*^Pr_2_Im^Me^)_2_(*η* ^2^‐Me_3_SiC≡CSiMe_3_)] **7**, [Ni(Mes_2_Im)_2_(*η* ^2^‐MeC≡CMe)] **14**, [Ni(Mes_2_Im)_2_(*η* ^2^‐MeOOCC≡CCOOMe)] **15**, [Ni(Mes_2_Im)_2_(*η* ^2^‐PhC≡CMe)] **16** and [Ni(Mes_2_Im)_2_(*η* ^2^‐HC≡C(4‐^*t*^Bu‐C_6_H_4_))] **17** (d_Ni−NHC_=Ni−C distance to the carbene carbon atom; d_C−C_=C−C distance of the alkyne, twist angle: twist between the planes NHC−Ni−NHC and C−Ni−C).

Compound	d_Ni−NHC_ [Å]	d_C−C_ [Å]	∢ NHC−Ni−NHC [°]	twist angle [°]
C	1.896(6)	1.310(6)	109.27(19)	1.76(19)
	1.915(4)			
D	1.917(8)	1.286(13)	1.917(8)	1.286(13)
	1.934(7)			
3	1.9097(14)	1.285(2)	102.42(6)	8.32(8)
	1.9239(14)			
5	1.9251(13)	1.302(3)	110.66(8)	7.90(8)
7	1.9183(15)	1.304(2)	114.54(6)	9.27(12)
	1.9149(15)			
14	1.9098(14)	1.280(2)	122.24(6)	9.60(7)
	1.9127(14)			
15	1.917(2)	1.300(4)	118.47(12)	3.26(13)
16	1.927(5)	1.291(7)	118.5(2)	5.73(22)
	1.913(5)			
17	1.921(3)	1.277(5)	124.59(14)	1.50(17)
	1.912(3)			

**Scheme 5:**
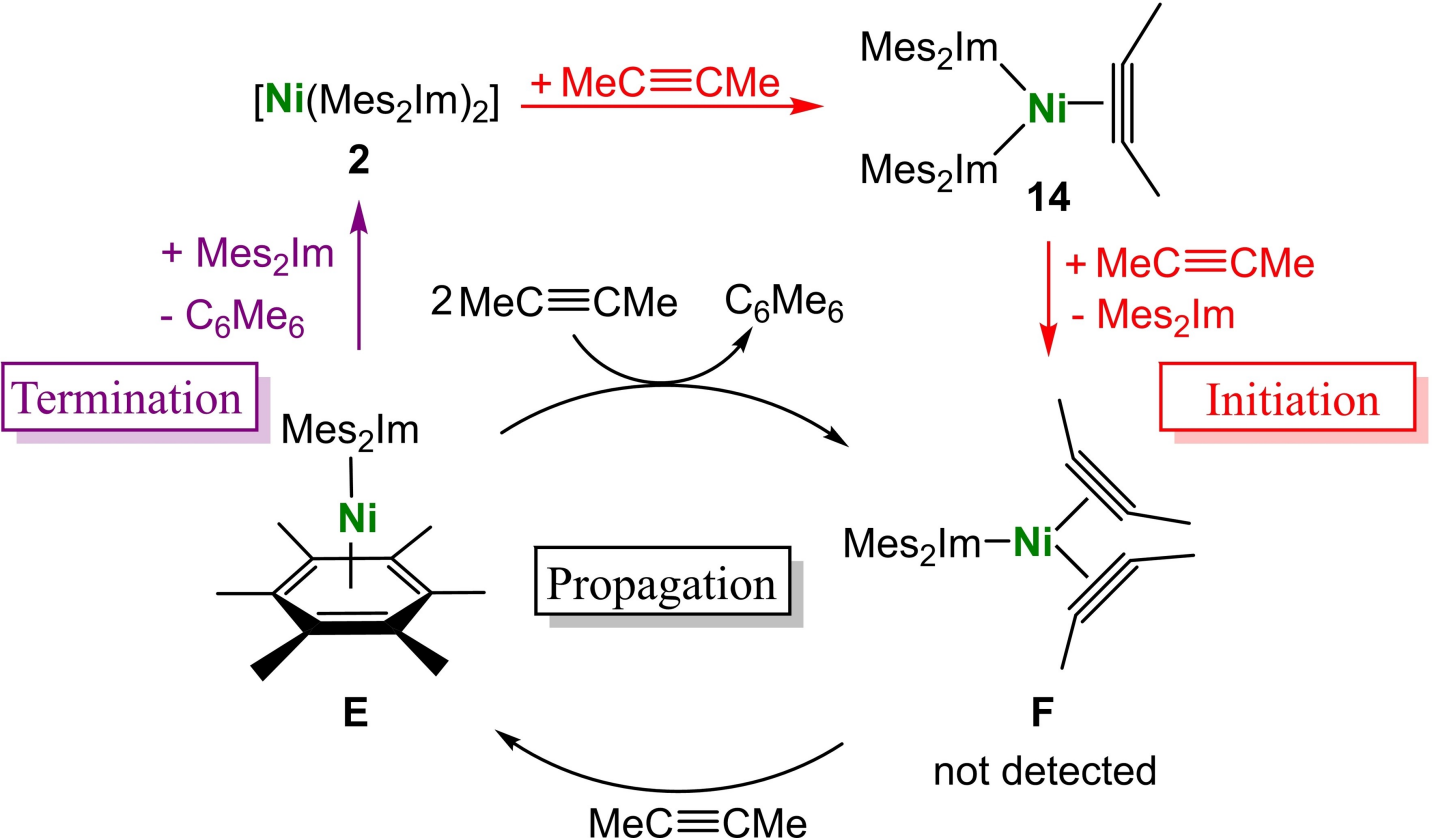
Proposed mechanism of the NHC nickel‐catalyzed cyclotrimerization of 2‐butyne.

NMR experiments as well as the isolation of the NHC nickel alkyne complexes point to a mechanism for the NHC Ni mediated alkyne trimerization as depicted in Scheme 5 for the trimerization of 2‐butyne. The first step of the catalytic cycle is the coordination of the alkyne to deep‐purple [Ni(Mes_2_Im)_2_] **2** to yield bright yellow [Ni(Mes_2_Im)_2_(*η* ^2^‐MeC≡CMe)] **14**, a step which occurs at low temperatures. In a second step, another alkyne molecule coordinates to the nickel atom to replace one of the NHC ligands with formation of the bis(alkyne) complex [Ni(Mes_2_Im)(*η* ^2^‐MeC≡Me)_2_] **F**. We have no evidence currently for the formation of **F**, but Louie et al.^[7]^ and Cavell et al.^[26]^ reported previously the synthesis of comparable mono‐NHC stabilized nickel olefin complexes of the type [(NHC)Ni(*η* ^2^‐R_2_C=CR_2_)_2_] using bulky NHC ligands such as Mes_2_Im or Dipp_2_Im. As we never detected intermediate **F**, we assume that the following reaction step, the addition of another equivalent alkyne to **F** with cyclization of the alkynes to give [(Mes_2_Im)Ni(*η* ^6^‐C_6_R_6_)] **E**, is very fast. Complex **E** was detected by NMR spectroscopy but defied all efforts at isolation. As the complexes **2** or **14** were never observed during catalysis, we propose that the formation of **2** and **14** are the initial steps to prepare the catalytic active species [Ni(Mes_2_Im)(*η* ^2^‐MeC≡Me)_2_] **F** (“Initiation” in Scheme 5, highlighted in red) and that the effective catalytic process occurs as a shuttle between the complexes **F** and **E** (“Propagation” in Scheme 5). At the end of the catalysis, the NHC ligand re‐coordinates to the nickel atom of **E** with elimination of the aromatic trimerization product and recovery of complex **2** (“Termination” in Scheme 5, highlighted in violet). This last step only occurs if the concentration of alkyne is very low, otherwise [Ni(Mes_2_Im)(*η* ^2^‐MeC≡Me)_2_] **F** will be formed directly to close the catalytic cycle. As our NMR studies on the reaction of **2** with a slight excess of 2‐butyne clearly reveal is this last step associated with the highest barrier.

**Figure 7:**
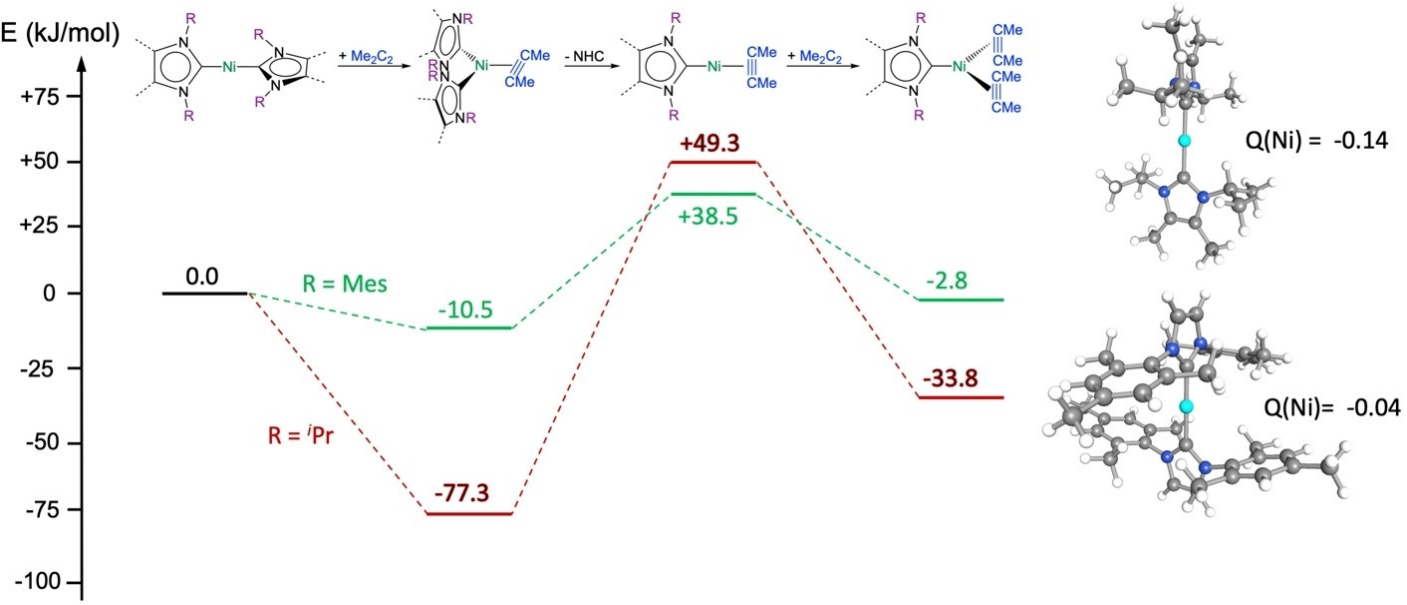
Energy profile of the initiation steps of the cyclotrimerization of 2‐butyne with [Ni(NHC)_2_] (NHC=^*i*^Pr_2_Im^Me^ **1^Me^**, red; Mes_2_Im **2**, green) according to DFT calculations (BP86//def2‐TZVP(Ni)/def2‐SVP(C,N,H)) and calculated NBO charges at the nickel atoms of [Ni(NHC)_2_]. Given are the ZPE corrected ground state energies in kJ/mol.

So what is the difference between [Ni(^*i*^Pr_2_Im)_2_] **1** or [Ni(^*i*^Pr_2_Im^Me^)_2_] **1^Me^** and [Ni(Mes_2_Im)_2_] **2** in the behavior towards alkynes? All three compounds form alkyne complexes, but only the complexes of the sterically more encumbered Mes_2_Im ligand enter the catalytic cycle at ambient temperatures. To answer this question DFT calculations (BP86//def2‐TZVP(Ni)/def2‐SVP(C,N,H)) have been performed on the initiation steps of the cyclotrimerization of 2‐butyne with [Ni(NHC)_2_] (NHC=^*i*^Pr_2_Im^Me^, Mes_2_Im; see Scheme 5). The results of these computations are given in Figure 7.

A comparison of the energy profile of the cyclotrimerization initiation steps of 2‐butyne with [Ni(^*i*^Pr_2_Im^Me^)_2_] **1^Me^** (red) and [Ni(Mes_2_Im)_2_] **2** (green) reveals that the profile is very shallow for **2** and each step is associated with a moderate energy change. The formation of the alkyne complexes [Ni(^*i*^Pr_2_Im^Me^)_2_(*η* ^2^‐MeC≡CMe)] **3** and [Ni(Mes_2_Im)_2_(*η* ^2^‐MeC≡CMe)] **14** is connected with a very different energy gain, −77.3 kJ/mol for **3** and only −10.5 kJ/mol for **14**. Assuming a dissociative process, the dissociation of the NHC ligand from [Ni(NHC)_2_(*η* ^2^‐MeC≡CMe)] requires +126.6 kJ/mol for the ^*i*^Pr_2_Im^Me^ complex, whereas for the Mes_2_Im complex only +49 kJ/mol are needed. The attachment of another alkyne to [Ni(NHC)(*η* ^2^‐MeC≡CMe)] is exothermic in both cases, −41.3 kJ/mol for the formation of [Ni(Mes_2_Im)(*η* ^2^‐MeC≡CMe)_2_] and −83.1 kJ/mol for [Ni(^*i*^Pr_2_Im^Me^)(*η* ^2^‐MeC≡CMe)_2_]. Thus, the potential surface of the nickel complex [Ni(Mes_2_Im)_2_] with both, low energy gain for alkyne addition and low energy loss for NHC dissociation, is nicely suited for catalysis, whereas for [Ni(^*i*^Pr_2_Im^Me^)_2_] **1^Me^** the alkyne complex seems to be too stable for further ligand loss (either alkyne or NHC) to enter a catalytic cycle at ambient temperatures.

As there is a distinct difference in the coordination of alkyne, specifically 2‐butyne, to [Ni(^*i*^Pr_2_Im^Me^)_2_] **1^Me^** (red) and [Ni(Mes_2_Im)_2_] **2** (green) it is interesting to track down the differences. Both ligands are different in their stereo‐electronic features. For this purpose the steric demand of the NHCs ^*i*^Pr_2_Im, ^*i*^Pr_2_Im^Me^ and Mes_2_Im expressed by their %*V*_bur_ (“percent buried volume”)[^12^, ^27^] was re‐evaluated on the basis of DFT geometry optimized structures (BP86//def2‐TZVP(all)) of [(NHC)Ni(CO)_3_]. With the aid of the Web application Samb*V*ca^[28]^ we obtained %*V*_bur_; values of ^*i*^Pr_2_Im (26.5 %)<^*i*^Pr_2_Im^Me^ (27.7 %)<Mes_2_Im (33.2 %),^[29]^ for fixed Ni−C_carbene_ distances of 2.00 Å, which are perfectly in line with our experimental findings. The *σ*‐donor and *π*‐acceptor properties of the NHC ligands can be described via the TEP (“Tolman electronic parameter”)[^27^, ^30^] and ^31^P or ^77^Se NMR shifts of NHC phosphinidenes and selenourea compounds,^[31]^ respectively. While our BP86//def2‐TZVP(all)‐calculated TEP values reveal no significant differences for ^*i*^Pr_2_Im (2054)∼Mes_2_Im (2055)∼^*i*^Pr_2_Im^Me^ (2056) in [(NHC)Ni(CO)_3_], the *π*‐acceptor abilities of the NHCs increase in the order ^*i*^Pr_2_Im (δ^31^P=−61.2 ppm, δ^77^Se=−3 ppm)<^*i*^Pr_2_Im^Me^ (δ^77^Se=−18 ppm)<Mes_2_Im (δ^31^P=−23 ppm, δ^77^Se=+27 ppm).^[31]^

As a consequence of these different donor and acceptor properties of the NHC ligands used in [Ni(NHC)_2_], different charges (see Figure 7; given are NBO charges) were calculated at nickel for the complexes [Ni(^*i*^Pr_2_Im^Me^)_2_] **1^Me^** (−0.14) and [Ni(Mes_2_Im)_2_] **2** (−0.04). Thus, the nickel atom of **1^Me^** is more electron‐rich compared to the metal atom of **2** and it should be expected that more electron density is transferred to the alkyne ligand of **1^Me^**. This is in line with the concept recently provided by Love and Kennepohl et al. for the stabilization of square planar d^10^ nickel *π*‐complexes bearing phosphine co‐ligands.^[32]^ These authors provided evidence that the stability of *π*‐complexes depends on the strength of the metal‐to‐ligand (alkyne or alkene) backbonding, which is critically influenced by charge transfer from the co‐ligands (here the NHCs) via the metal atom to the *π*‐acceptor ligand.

These expectations can be confirmed by the experimental data obtained for the complexes [Ni(^*i*^Pr_2_Im)_2_(*η* ^2^‐MeC≡CMe)] **D**,^[10]^ [Ni(^*i*^Pr_2_Im^Me^)_2_(*η* ^2^‐MeC≡CMe)] **3** and [Ni(Mes_2_Im)_2_(*η* ^2^‐MeC≡CMe)] **14**. As the molecular structure is known for all three complexes it should be noted here that the experimentally determined C≡C bond lengths in principle do not provide a good basis for this discussion, as the differences lie within the experimental error of the structure determination (3σ). However, the trend observed here is as expected, i.e., that the C≡C bond length of the alkyne ligand of the Mes_2_Im complex [Ni(Mes_2_Im)_2_(*η* ^2^‐MeC≡CMe)] **14** is the shortest while those of the complexes **D** and **3** are longer due to enhanced electron transfer to the alkyne: 1.280(2) (**14**) ≪1.285(2) (**3**) <1.286(13) (**D**). This order of the net donor properties is also reflected in the observed coordination shifts of the alkyne carbon atoms (▵*δ*_C_ [ppm]=44.2 (**14**) <47.2 (**3**) <47.5 (**D**))[^10^, ^20b^] and even more pronounced in the coordination shifts of the *ν*_C≡C_ stretching vibrations (▵*ν*_C≡C_ [cm^−1^]=425 (**14**) <448 (**3**) <455 (**D**))[^10^, ^20b^] (cf. Tables 1 and 3).

Different degrees of C≡C bond activation of the alkyne ligands of [Ni(^*i*^Pr_2_Im^Me^)_2_(*η* ^2^‐MeC≡CMe)] **3** and [Ni(Mes_2_Im)_2_(*η* ^2^‐MeC≡CMe)] **14** was also confirmed by DFT calculations, either using the C≡C distances (**3**: 1.304 Å, **14**: 1.297 Å), the calculated charges on the alkyne carbon atoms (e.g., NBO‐charges: **3**: −0.245, **14**: −0.225), calculated (uncorrected) C≡C stretching frequencies (**3**: 1852 cm^−1^; **14**: 1876 cm^−1^) or the C≡C Wiberg bond indices (**3**: 1.809, **14**: 1.835). A detailed analysis also reveals that alkyne activation (i.e., the strength of the *π*‐backbond) is indirectly influenced by the steric demand of the NHC ligand in so far, as the complexes [Ni(^*i*^Pr_2_Im^Me^)_2_(*η* ^2^‐MeC≡CMe)] **3** and [Ni(Mes_2_Im)_2_(*η* ^2^‐MeC≡CMe)] **14** adopt different angles C_NHC_−Ni−C_NHC_. It is well known that a decrease of the bite angle L−M−L (i.e., C_NHC_−Ni−C_NHC_) in d^10^‐[ML_2_] (L=neutral 2VE donor ligand) and related complexes is connected with a more favorable *π*‐backbonding in complexes d^10^‐[ML_2_(alkyne)] and thus an increase of the net charge donation from the metal center to the *π*‐ligand.[^17a^, ^17c^, ^33^] The bite angles of the complexes [Ni(NHC)_2_(*η* ^2^‐MeC≡CMe)] decrease in the order 122.24(6)° (**14**) ≫102.42(6)° (**3**) >100.4(3)° (**D**). To evaluate the contribution of the different bite angles we optimized the geometry of [Ni(^*i*^Pr_2_Im^Me^)_2_(*η* ^2^‐MeC≡CMe)] **3** with the fixed angle of geometry optimized [Ni(Mes_2_Im)_2_(*η* ^2^‐MeC≡CMe)] **14** (angle C_NHC_−Ni−C_NHC_ 123.60°, exp.:122.24(6)°). The potential for a change of the C_NHC_−Ni−C_NHC_ angle is very shallow, as the energies of both optimized structures of [Ni(^*i*^Pr_2_Im^Me^)_2_(*η* ^2^‐MeC≡CMe)] **3** differ by a mere 2.8 kJ/mol. However, the parameters evaluated above for the alkyne ligand of **3** and **14** adopt for the complex of the constrained geometry complex values within those computed for **3** and **14**, for example 1.301 Å for the C≡C distance (**3**: 1.304 Å, **14**: 1.297 Å), −0.233 for the NBO‐charges on the alkyne carbon atoms (**3**: −0.245, **14**: −0.225), and 1852 cm^−1^ for the C≡C stretching frequencies (**3**: 1852 cm^−1^; **14**: 1876 cm^−1^).

In total, we attribute the much higher stability of [Ni(^*i*^Pr_2_Im^Me^)_2_(*η* ^2^‐MeC≡CMe)] **3** with respect to [Ni(Mes_2_Im)_2_(*η* ^2^‐MeC≡CMe)] **14** to three main reasons: (i) electron transfer from the NHC to the metal to the alkyne ligand is higher for the *N*‐alkyl compared to the *N*‐aryl substituted NHC ligands in [Ni(NHC)_2_(*η* ^2^‐MeC≡CMe)] due to different electron donor/acceptor properties of the NHC ligand. (ii) Electron transfer from the metal center to the alkyne ligand is enhanced for the *N*‐alkyl compared to the *N*‐aryl substituted NHC ligands due to their different steric size, as smaller NHC ligands (such as ^*i*^Pr_2_Im^Me^ or ^*i*^Pr_2_Im) can adopt smaller C_NHC_−Ni−C_NHC_ bite angles, which leads to increased *π*‐backdonation to the alkyne. (iii) Ligand dissociation is facilitated for the complex of the sterically more encumbered NHC ligand, i.e., [Ni(Mes_2_Im)_2_(*η* ^2^‐MeC≡CMe)] **14** loses the NHC ligand more readily than [Ni(^*i*^Pr_2_Im)_2_(*η* ^2^‐MeC≡CMe)] **D** ^[10]^ and [Ni(^*i*^Pr_2_Im^Me^)_2_(*η* ^2^‐MeC≡CMe)] **3**. All these factors lead to a significantly enhanced stability of the alkyne complexes of the *N*‐alkyl substituted NHCs and are thus the reason why these complexes are not catalytically active for alkyne oligomerization at ambient temperatures.

## Conclusion

A case study on the effect of two different NHC ligands in complexes [Ni(NHC)_2_] (NHC=^*i*^Pr_2_Im^Me^ **1^Me^**, Mes_2_Im **2**) is reported; it presents some details to demonstrate how small differences in the stereo‐electronic features of closely related ligands can significantly alter the reactivity pattern. The reaction of (suitable precursors of) both complexes with alkynes afforded *η* ^2^‐(*C*,*C*)‐alkyne complexes [Ni(NHC)_2_](*η* ^2^‐alkyne)] (**3**–**18**), although the number of complexes available for [Ni(Mes_2_Im)_2_] **2** is limited to small alkynes and good acceptor alkynes. Many of the [Ni(^*i*^Pr_2_Im^Me^)_2_] complexes **3**–**13** are unstable upon heating, leading to various, in many cases unidentified decomposition products. However, for the thermal reaction of the complexes [Ni(^*i*^Pr_2_Im^Me^)_2_(*η* ^2^‐HC≡C(*p*‐Tol))] **11** and [Ni(^*i*^Pr_2_Im^Me^)_2_(*η* ^2^‐HC≡C(4‐^*t*^Bu‐C_6_H_4_))] **12** the isomers **11 a** and **12 a** were identified. DFT calculations, as well as deuteration experiments, were in accordance with the formation of **11 a** and **12 a** via a concerted or nickel‐mediated C−H addition of a NHC methyl C−H bond across the C≡C triple bond of the coordinated alkyne.

Complex **2** cyclotrimerizes alkynes at ambient conditions, which is in contrast to the behavior found for **1^Me^** or **1**. NMR exploration of the reaction of **2** with 2‐butyne gave evidence for the formation of the complexes [(Mes_2_Im)Ni(*η* ^6^‐C_6_Me_6_)] **E** and [Ni(Mes_2_Im)_2_(*η* ^2^‐MeC≡CMe)] **14** as intermediates of the reaction. A mechanism for the NHC‐nickel catalyzed cyclotrimerization of 2‐butyne was proposed, which involves coordination of the alkyne to [Ni(Mes_2_Im)_2_] **2** to yield [Ni(Mes_2_Im)_2_(*η* ^2^‐MeC≡CMe)] **14** and [Ni(Mes_2_Im)(*η* ^2^‐MeC≡Me)_2_] **F** with loss of one NHC ligand as the initiation step of the catalysis. The efficient steps of the catalytic cycle involve addition of 2‐butyne to [Ni(Mes_2_Im)(*η* ^2^‐MeC≡Me)_2_] **F** with cyclization to yield [(Mes_2_Im)Ni(*η* ^6^‐C_6_Me_6_)] **E** and re‐formation of **F** with arene release. The re‐coordination of the NHC ligand to the nickel atom of **E** with elimination of the aromatic trimerization product and recovery of complex **2** at the end of the catalysis is the termination of the catalytic cycle.

This study demonstrates for the example of bis‐NHC nickel alkyne complexes and their reactivity how valuable NHCs are in the fine‐tuning of substrate binding, electron transfer and reactivity. Although the differences in the TEP of both NHCs under investigation is small, the differences in the electron transfer of the complexes [Ni(NHC)_2_] to a coordinated substrate are quite impressive. The increase of the steric demand of the NHC lead, of course, to a different accessibility of the metal center (steric protection) and to different complex stabilities as co‐ligand/NHC dissociation is facilitated for the bulkier ligand. But we also demonstrate here that steric properties of the NHC significantly influence the donor properties of [M(NHC)_2_]‐moieties by the C_NHC_−M−C_NHC_ bite‐angle NHC ligands of different size can adopt in the final product. Furthermore, we have shown previously[^11a^, ^34^] that simple electron‐transfer processes are possible if the substrate cannot bind to a (sterically encumbered) complex [M(NHC)_2_] and that thus radical processes dominate its reactivity and catalysis. We anticipate that, as shown herein, further tuning of the NHC stereo‐electronics, keeping [M(NHC)_2_] units intact, will lead to the (further) design of catalysts which enter different reaction channels for similar (or even same) starting materials.

## Crystallographic details

Crystal data collection and processing parameters are given in the Supporting Information. Deposition Numbers 2100093 (**15**), 2100094 (**5**), 2100095 (**14**), 2100096 (**3**), 2100097 (**B**), 2100098 (**17**), 2100099 (**11 a**), 2100100 (**16**), and 2100101 (**7**) contain the supplementary crystallographic data for this paper. These data are provided free of charge by the joint Cambridge Crystallographic Data Centre and Fachinformationszentrum Karlsruhe Access Structures service.

## Conflict of interest

The authors declare no conflict of interest.

## Supporting information

As a service to our authors and readers, this journal provides supporting information supplied by the authors. Such materials are peer reviewed and may be re‐organized for online delivery, but are not copy‐edited or typeset. Technical support issues arising from supporting information (other than missing files) should be addressed to the authors.

Supporting Information
