# Supplementary material for: Case Study of N‐ i Pr versus N‐Mes Substituted NHC Ligands in Nickel Chemistry: The Coordination and Cyclotrimerization of Alkynes at [Ni(NHC)2]
Source: Chemistry. 2021 Nov 16;27(71):17849–61. doi: 10.1002/chem.202103093 (PMC9299202; doi:10.1002/chem.202103093)
Supplement: Supplementary file 1 — Supporting Information [file CHEM-27-17849-s001.pdf]

Chemistry–A European Journal

Supporting Information

Case Study of *N*-^{i}Pr versus *N*-Mes Substituted NHC Ligands in Nickel Chemistry: The Coordination and Cyclotrimerization of Alkynes at [Ni(NHC)$_2$]

Lukas Tendera, Moritz Helm, Mirjam J. Krahfuss, Maximilian W. Kuntze-Fechner, and Udo Radius*

Content:

1) Experimental Section
2) Crystallographic Details
3) NMR Spectra
4) Computational Details
5) Cartesian Coordinates of the DFT optimized geometries

1) Experimental Section

General

All reactions and subsequent manipulations were performed under an argon atmosphere using standard Schlenk techniques as reported previously [1] or in a glovebox (Innovative Technology Inc. and Braun Uni Lab). All reactions were carried out in oven-dried glassware. Toluene, hexane, pentane and THF were purified by distillation from an appropriate drying agent (sodium with benzophenone as indicator). C_6D_6, THF-d_8 and were purchased from Sigma-Aldrich. Mes_2Im,[2] $^{i}Pr_2Im^{Me}$,[3] [Ni(η^4-COD)$_2$][4] and [Ni(Mes_2Im)$_2$][5] were prepared according to published procedures. All other reagents were purchased from Aldrich or ABCR and used without further purification. NMR spectra were recorded at 298 K using Bruker Avance 400 (^1H, 400 MHz; ^{13}C, 100 MHz), or Bruker Avance NEO 400 (^1H, 400 MHz; ^{13}C, 100 MHz), or Bruker Avance 500 (^1H, 500 MHz; $^{13}C\{^1H\}$, 126 MHz) spectrometers. ^1H NMR chemical shifts are reported relative to TMS and were referenced via residual proton resonances of the corresponding deuterated solvent (C_6D_5H: 7.16 ppm, C_4D_7HO: 1.72, 3.58 ppm) whereas $^{13}C\{^1H\}$ NMR spectra are reported relative to TMS using the natural-abundance carbon resonances (C_6D_6: 128.06 ppm, THF-d_8: 25.31, 67.21 ppm). Coupling constants are given in Hertz. Elemental analyses were performed in the microanalytical laboratory of the Institute of Inorganic Chemistry, Universität Würzburg, using an Elementar vario micro cube. GC-MS analyses were performed using a Thermo Fisher Scientific Trace 1310 gas chromatograph (column: TG-SQC 5% phenyl methyl siloxane, 15 m, Ø 0.25 mm, film 0.25 μm; injector: 250 °C; oven: 40 °C (2min), 40 °C to 280 °C; carrier gas: He (1.2 mL min^{-1})).

Synthesis

Synthesis of [Ni$_2$($^{i}Pr_2Im^{Me}$)$_4$(μ-(η^2:η^2)-COD)] B and [Ni($^{i}Pr_2Im^{Me}$)$_2$(COD)] B'

A solution of $^{i}Pr_2Im^{Me}$ (680 mg, 3.77 mmol) in 15 mL of THF was cooled to -78 °C and added at this temperature to a solution of [Ni(COD)$_2$] (520 mg,1.89 mmol) in 15 mL THF. The reaction mixture was allowed to slowly warm to room temperature overnight and was then filtered over a pad of Celite. All volatiles were removed in vacuo and the remaining residue was suspended in 15 mL of hexane. The product was filtered off and dried in vacuo to give a yellow powder (653 mg). The isolated product contains a mixture of [Ni$_2$($^{i}Pr_2Im^{Me}$)$_4$(μ-(η^2:η^2)-COD)] **B** and [Ni($^{i}Pr_2Im^{Me}$)$_2$(η^4-COD)] **B'** (60:40).

Yellow crystals of [Ni$_2$($^{i}Pr_2Im^{Me}$)$_4$(μ-(η^2:η^2)-COD)] **B** suitable for single-crystal X-ray diffraction were obtained by slow evaporation of a saturated benzene solution at room temperature.

[$Ni_2(^iPr_2Im^{Me})_4(\mu$-($\eta^2$:$\eta^2$)-COD)] **B**

^{1}H-NMR (400.1 MHz, C_6D_6, 298 K): δ = 1.42 (d, br, 48H, ^{i}Pr-CH_3), 1.88 (s, 24H, NCCH_3CCH_3N), 2.22 (m, 4H, COD-CH_2), 2.59 (d, br, 4H, COD-CH_2), 2.84 (m, 4H, COD-C*H*), 6.03 (sept, 8H, $^3J_{HH}$ = 7.1 Hz, ^{i}Pr-C*H*).

^{13}C{^{1}H}-NMR (100.6 MHz, C_6D_6, 298 K): δ = 10.6 (NC*C*H$_3$*C*CH$_3$N), 23.0 (^{i}Pr-*C*H$_3$), 38.7 (COD-*C*H$_2$), 51.8 (^{i}Pr-*C*H), 54.5 (COD-*C*H), 122.2 (N*C*CH$_3$*C*CH$_3$N), 206.5 (N*C*N).

[$Ni(^iPr_2Im^{Me})_2(\eta^4$-COD)] **B'**

^{1}H-NMR (400.1 MHz, C_6D_6, 298 K): δ = 1.33 (d, 24H, $^3J_{HH}$ = 7.2 Hz, ^{i}Pr-CH_3), 1.86 (s, 12H, NCCH_3CCH_3N), 2.47 (s, 8H, COD-CH_2), 4.38 (s, 4H, COD-C*H*), 5.90 (sept, 4H, $^3J_{HH}$ = 7.2 Hz, ^{i}Pr-C*H*).

^{13}C{^{1}H}-NMR (100.6 MHz, C_6D_6, 298 K): δ = 10.6 (NC*C*H$_3$*C*CH$_3$N), 22.8 (^{i}Pr-*C*H$_3$), 33.6 (COD-*C*H$_2$), 51.9 (^{i}Pr-*C*H), 122.6 (N*C*CH$_3$*C*CH$_3$N), 205.4 (N*C*N).

Synthesis of [$Ni(^iPr_2Im^{Me})_2(\eta^2$-MeC≡CMe)] 3

2-Butyne (12.6 *µL*, 8.68 mg, 161 *µ*mol) was added at room temperature to a solution of a 60:40 mixture of [$Ni_2(^iPr_2Im^{Me})_4(\mu$-($\eta^2$:$\eta^2$)-COD)] **B** and [$Ni(^iPr_2Im^{Me})_2(\eta^4$-COD)] **B'** (76.0 mg, 156 *µ*mol Ni) in 5 mL of benzene. The reaction mixture was stirred for 10 min at room temperature. All volatiles were removed in vacuo and the remaining residue was dried in vacuo to give a yellow powder (60.0 mg, 127 *µ*mol, 81 %).

Yellow crystals of [$Ni(^iPr_2Im^{Me})_2(\eta^2$-MeC≡CMe)] **3** suitable for single-crystal X-ray diffraction were obtained from a saturated solution in hexane at -30 °C.

Elemental analysis $C_{26}H_{46}N_4Ni$ [473.38 g/mol] calculated: C 65.97, H 9.80, N 11.84; found: C 65.33, H 9.88, N 11.56

^{1}H-NMR (400 MHz, C_6D_6, 25 °C): δ = 1.27 (d, 24H, $^3J_{HH}$ = 7.2 Hz, ^{i}Pr-CH_3), 1.87 (s, 12H, NCCH_3CCH_3N), 2.75 (s, 6H, H_3CC≡CCH_3), 6.22 (sept, 4H, $^3J_{HH}$ = 7.2 Hz, ^{i}Pr-C*H*).

^{13}C{^{1}H-NMR (100 MHz, C_6D_6, 25 °C): δ = 10.5 (NC*C*H$_3$*C*CH$_3$N), 13.4 (H$_3$*C*C≡C*C*H$_3$), 22.3 (^{i}Pr-*C*H$_3$), 52.0 (^{i}Pr-*C*H), 121.6 (*C*≡*C*), 122.8 (N*C*CH$_3$*C*CH$_3$N), 205.1 (N*C*N).

IR (ATR [cm^{-1}]): 2969 (w), 2932 (w), 2884 (w), 2829 (w), 1785 (m), 1640 (vw), 1464 (w), 1407 (w), 1377 (vw), 1362 (w), 1338 (s), 1289 (s), 1264 (vs), 1203 (w), 1161 (vw), 1125 (w), 1098 (m), 1060 (w), 1027 (m), 961 (vw), 902 (w), 775 (vw), 753 (w), 693 (w), 678 (m), 574 (w), 551 (w), 469 (vw), 432 (vw).

Synthesis of [Ni(^{i}Pr$_2$Im^{Me})$_2$(η^2-H$_7$C$_3$C≡CC$_3$H$_7$)] 4

4-Octyne (35.5 μL, 26.6 mg, 242 μmol) was added at room temperature to a solution of a 60:40 mixture of [Ni$_2$(^{i}Pr$_2$Im^{Me})$_4$(μ-(η^2:η^2)-COD)] **B** and [Ni(^{i}Pr$_2$Im^{Me})$_2$(η^4-COD)] **B'** (109 mg, 224 μmol Ni) in 5 mL of toluene. The reaction mixture was stirred for 16 h at room temperature and was then filtered over a pad of Celite. All volatiles were removed in vacuo and the remaining residue was dried in vacuo to give a yellow powder (100 mg, 188 μmol, 84 %).

Elemental analysis $C_{30}H_{54}N_4Ni$ [529.48 g/mol] calculated: C 68.05, H 10.28, N 10.58; found: C 67.39, H 10.53, N 10.01.

^{1}H-NMR (400 MHz, C_6D_6, 25 °C): δ = 1.18 (t, 6H, $^3J_{HH}$ = 7.3 Hz, CH$_2$CH$_2$CH_3), 1.26 (d, 24H, $^3J_{HH}$ = 7.2 Hz, ^{i}Pr-CH_3), 1.85 (m, 4H, CH$_2$CH_2CH$_3$), 1.86 (s, 12H, NCCH_3CCH_3N), 3.07 (t, 4H, $^3J_{HH}$ = 7.3 Hz, CH_2CH$_2$CH$_3$), 6.15 (sept., 4H, $^3J_{HH}$ = 7.2 Hz, ^{i}Pr-CH).

^{13}C{^{1}H}-NMR (100 MHz, C_6D_6, 25 °C): δ = 10.5 (NCCH$_3$CCH$_3$N), 14.8 (CH$_2$CH$_2$$CH_3$), 22.1 (^{i}Pr-$CH_3$), 25.1 (CH$_2$$CH_2CH_3$), 31.5 ($CH_2CH_2CH_3$), 51.8 (^{i}Pr-$C$H), 122.8 (N$CCH_3$$CCH_3$N), 126.4 (H$_7C_3$$C$≡$CC_3H_7$), 205.5 (N$C$N).

IR (ATR [cm^{-1}]): 2968 (m), 2925 (m), 2863 (w), 2805 (w), 2166 (wv), 2055 (vw), 1996 (wv), 1935 (vw), 1778 (w), 1639 (wv), 1462 (w), 1406 (w), 1379 (m), 1363 (s), 1338 (w), 1305 (m), 1286 (m), 1263 (vs), 1205 (w), 1160 (vw), 1124 (w), 1097 (w), 1059 (w), 1018 (w), 959 (w), 924 (vw), 857 (vw), 751 (w), 691 (w), 679 (w), 594 (w), 461 (w).

Synthesis of [Ni(^{i}Pr$_2$Im^{Me})$_2$(η^2-PhC≡CPh)] 5

A solution of diphenylacetylene (44.6 mg, 250 μmol) in 5 mL of toluene was added at room temperature to a solution of a 60:40 mixture of [Ni$_2$(^{i}Pr$_2$Im^{Me})$_4$(μ-(η^2:η^2)-COD)] **B** and [Ni(^{i}Pr$_2$Im^{Me})$_2$(η^4-COD)] **B'** (118 mg, 243 μmol Ni) in 10 mL of toluene. The mixture was stirred for 3 h at room temperature and was then filtered over a pad of Celite. All volatiles were removed in vacuo and the remaining residue was suspended in 10 mL of pentane. The product was filtered off, washed with 3 mL of pentane and dried in vacuo to give a purple powder (93 mg, 155 μmol, 64 %).

Red crystals of [Ni(^{i}Pr$_2$Im^{Me})$_2$(η^2-PhC≡CPh)] **5** suitable for single-crystal X-ray diffraction were obtained from a saturated solution in hexane at -30 °C.

Elemental analysis $C_{36}H_{50}N_4Ni$ [597.52 g/mol] calculated: C 72.37, H 8.43, N 9.38; found: C 72.44, H 8.55, N 9.22.

^{1}H-NMR (400 MHz, C_6D_6, 25 °C): δ = 1.17 (d, 24H, $^3J_{HH}$ = 7.1 Hz, ^{i}Pr-CH_3), 1.81 (s, 12H, NCCH_3CCH_3N), 6.09 (sept, 4H, $^3J_{HH}$ = 7.1 Hz, ^{i}Pr-CH), 6.99 (m, 2H, aryl-CH_{para}), 7.20 (m, 4H, aryl-CH_{meta}), 7.69 (m, 4H, aryl-CH_{ortho}).

^{13}**C{^{1}H-NMR** (100 MHz, C_6D_6, 25 °C): δ = 10.5 (NC*C*H$_3$C*C*H$_3$N), 22.1 (^{i}Pr-*C*H$_3$), 52.5 (^{i}Pr-*C*H), 123.5 (N*C*CH$_3$*C*CH$_3$N), 123.8 (aryl-*C*H$_{para}$), 128.9 (aryl-*C*H$_{ortho}$), 139.0 (aryl-*C*$_{ipso}$), 139.2 (*C*≡*C*), 201.7 (N*C*N).

IR (ATR [cm^{-1}]): 3062 (w), 3035 (w), 2968 (w), 2930 (w), 2872 (w), 1754 (m), 1734 (m), 1635 (vw), 1582 (m), 1514 (vw), 1474 (w), 1434 (w), 1401 (m), 1347 (s), 1274 (s), 1210 (m), 1164 (w), 1129 (m), 1100 (m), 1065 (m), 1019 (m), 994 (w), 961 (w), 904 (w), 882 (w), 797 (w), 756 (vs), 692 (vs), 626 (w), 596 (m), 551 (w), 510 (w), 455 (w).

Synthesis of [Ni(^{i}Pr$_2$Im^{Me})$_2$(η^2-MeOOCC≡CCOOMe)] 6

Dimethyl acetylene dicarboxylate (35.1 µL, 40.5 mg, 285 µmol) was added at room temperature to a solution of a 60:40 mixture of [Ni$_2$(^{i}Pr$_2$Im^{Me})$_4$(μ-(η^2:η^2)-COD)] **B** and [Ni(^{i}Pr$_2$Im^{Me})$_2$(η^4-COD)] **B'** (135 mg, 277 µmol Ni) in 5 mL of toluene. The mixture was stirred for 1 h at room temperature and was then filtered over a pad of Celite. All volatiles were removed in vacuo and the remaining residue was suspended in 6 mL of hexane. The product was filtered off, washed with 3 mL of hexane and dried in vacuo to give an orange powder (108 mg,192 µmol, 70 %).

Elemental analysis $C_{28}H_{46}N_4NiO_4$ [561.39 g/mol] calculated: C 59.91, H 8.26, N 9.98; found: C 58.90, H 7.98, N 8.62.

^{1}**H-NMR** (400 MHz, C_6D_6, 25 °C): δ = 1.17 (d, 24H, $^3J_{HH}$ = 7.2 Hz, ^{i}Pr-C*H$_3$*), 1.74 (s, 12H, NC*H$_3$*CC*H$_3$*N), 3.55 (s, 6H, COOC*H$_3$*), 5.97 (sept., 4H, $^3J_{HH}$ = 7.2 Hz, ^{i}Pr-C*H*).

^{13}**C{^{1}H}-NMR** (100 MHz, C_6D_6, 25 °C): δ = 10.3 (NC*C*H$_3$*C*CH$_3$N), 21.9 (^{i}Pr-*C*H$_3$), 50.5 (COO*C*H$_3$), 53.1 (^{i}Pr-*C*H), 124.4 (N*C*CH$_3$*C*CH$_3$N), 136.8 (MeOOC*C*≡*C*COOMe), 170.7 (*C*OOCH$_3$), 194.3 (N*C*N).

IR (ATR [cm^{-1}]): 2973 (w), 2875 (w), 1749 (m), 1683 (s), 1659 (m), 1463 (w), 1426 (w), 1408 (w), 1354 (m), 1301 (w), 1290 (w), 1180 (m), 1125 (s).

Synthesis of [Ni(^{i}Pr$_2$Im^{Me})$_2$(η^2-Me$_3$SiC≡CSiMe$_3$] 7

Bis(trimethylsilyl)acetylene (48.1 µL, 37.0 mg, 217 µmol) was added at room temperature to a solution of a 60:40 mixture of [Ni$_2$(^{i}Pr$_2$Im^{Me})$_4$(μ-(η^2:η^2)-COD)] **B** and [Ni(^{i}Pr$_2$Im^{Me})$_2$(η^4-COD)] **B'** (98 mg, 201 µmol Ni) in 5 mL of benzene. The mixture was stirred for 18 h at room temperature and was then filtered over a pad of Celite. All volatiles were removed in vacuo and the remaining residue was dissolved in 3 mL of hexane and stored at -30 °C for one week. The

supernatant solution was removed via syringe to obtain yellow crystals (20 mg, 34.0 µmol, 17 %).
The obtained crystals of [Ni($^{i}Pr_2Im^{Me}$)$_2$(η^2-$Me_3SiC{\equiv}CSiMe_3$)] **7** were also suitable for single-crystal X-ray diffraction.
Elemental analysis $C_{30}H_{58}N_4NiSi_2$ [589.69 g/mol] calculated: C 61.11, H 9.91, N 9.50 found: C 61.21, H 10.12, N 9.64.

^{1}H-NMR (400 MHz, C_6D_6, 25 °C): δ = 0.41 (s, 18H, Si(C*H*$_3$)$_3$), 1.15 (d, 12H, $^3J_{HH}$ = 7.1 Hz, ^{i}Pr-C*H*$_3$), 1.41 (d, 12H, $^3J_{HH}$ = 7.1 Hz, ^{i}Pr-C*H*$_3$), 1.86 (s, 12H, NCC*H*$_3$CC*H*$_3$N), 5.90 (sept., 4H, $^3J_{HH}$ = 7.1 Hz, ^{i}Pr-C*H*).

^{13}C{^{1}H}-NMR (100 MHz, C_6D_6, 25 °C): δ = 2.3 (Si(*C*H$_3$)$_3$), 10.6 (NC*C*H$_3$C*C*H$_3$N), 22.2 (^{i}Pr-*C*H$_3$), 22.4 (^{i}Pr-*C*H$_3$), 51.8 (^{i}Pr-*C*H), 123.0 (N*C*CH$_3$*C*CH$_3$N), 159.8 (Me$_3$Si*C*≡*C*SiMe$_3$), 205.1 (N*C*N).

IR (ATR [cm^{-1}]): 2969 (m), 1659 (w), 1466 (w), 1438 (w), 1410 (w), 1358 (s), 1293 (m), 1253 (w), 1235 (w), 1216 (w), 1164 (vw), 1132 (w), 1106 (w), 1058 (w), 905 (vw), 849 (vs), 753 (m), 699 (w), 682 (w), 612 (vw), 587 (vw), 550 (w), 531 (w), 455 (vw).

Synthesis of [Ni($^{i}Pr_2Im^{Me}$)$_2$(η^2-PhC≡CMe)] 8

1-Phenyl-1-propyne (26.0 µL, 24.3 mg, 209 µmol) was added at room temperature to a solution a 60:40 mixture of [Ni$_2$($^{i}Pr_2Im^{Me}$)$_4$(*µ-(η^2:η^2)*-COD)] **B** and [Ni($^{i}Pr_2Im^{Me}$)$_2$(η^4-COD)] **B’** (94 mg, 193 µmol Ni) in 5 mL of benzene. The mixture was stirred for 2 h at room temperature and was then filtered over a pad of Celite. All volatiles were removed in vacuo and the remaining residue was suspended in 4 mL of hexane. The product was filtered off, washed with 3 mL of hexane and dried in vacuo to give an orange powder (54 mg, 101 µmol, 52 %).

Elemental analysis $C_{31}H_{48}N_4Ni$ [535.45 g/mol] calculated: C 69.54, H 9.04, N 10.46 found: C 67.95, H 8.89, N 10.17.

^{1}H-NMR (400 MHz, C_6D_6, 25 °C): δ = 1.20 (d, 12H, $^3J_{HH}$ = 7.1 Hz, ^{i}Pr-C*H*$_3$), 1.26 (d, 12H, $^3J_{HH}$ = 7.1 Hz, ^{i}Pr-C*H*$_3$), 1.82 (s, 6H, NCC*H*$_3$CC*H*$_3$N), 1.87 (s, 6H, NCC*H*$_3$CC*H*$_3$N), 2.90 (s, 3H, C≡CC*H*$_3$), 6.11 (sept., 2H, $^3J_{HH}$ = 7.1 Hz, ^{i}Pr-C*H*), 6.17 (sept., 2H, $^3J_{HH}$ = 7.1 Hz, ^{i}Pr-C*H*), 6.99 (m, 1H, aryl-C*H*$_{para}$), 7.25 (m, 2H, aryl-C*H*$_{meta}$), 7.54 (m, 1H, aryl-C*H*$_{ortho}$), 7.56 (m, 1H, aryl-C*H*$_{ortho}$).

^{13}C{^{1}H}-NMR (100. MHz, C_6D_6, 25 °C): δ = 10.5 (NC*C*H$_3$C*C*H$_3$N), 10.6 (NC*C*H$_3$C*C*H$_3$N), 15.3 (C≡C*C*H$_3$), 22.1 (^{i}Pr-*C*H$_3$), 22.3 (^{i}Pr-*C*H$_3$), 52.2 (^{i}Pr-*C*H), 52.4 (^{i}Pr-*C*H), 122.7 (aryl-*C*H$_{para}$), 123.2 (N*C*CH$_3$*C*CH$_3$N), 123.2 (N*C*CH$_3$*C*CH$_3$N), 127.1 (Ph*C*≡*C*), 127.9 (aryl-*C*H$_{meta}$), 129.5 (aryl-*C*H$_{ortho}$), 137.2 (C≡*C*Me), 138.8 (aryl-*C*$_{ipso}$), 203.32 (N*C*N).

IR (ATR [cm^{-1}]): 2967 (m), 2930 (w), 2872 (w), 2820 (w), 2082 (vw), 1760 (m), 1584 (m), 1478 (w), 1463 (w), 1436 (w), 1404 (w), 1385 (w), 1364 (w),1344 (m), 1290 (s), 1270 (vs), 1208 (w), 1162 (w),1099 (w), 1064 (w), 1025 (vw), 962 (w), 904 (w), 779 (w), 756 (s), 697 (s), 680 (m), 657 (w), 613 (vw), 552 (w), 530 (w), 460 (vw).

NMR Experiment for the Synthesis of [Ni(^{i}Pr$_2$Im^{Me})$_2$(η^2-HC≡CC$_3$H$_7$)] 9

A Young-NMR-Tube was charged with a solution of a 60:40 mixture of [Ni$_2$(^{i}Pr$_2$Im^{Me})$_4$(μ-(η^2:η^2)-COD)] **B** and [Ni(^{i}Pr$_2$Im^{Me})$_2$(η^4-COD)] **B' 1** (13.0 mg, 26.5 μmol Ni) in 0.6 mL of C_6D_6. 1-Pentyne (2.84 μL, 1.96 mg; 28.8 μmol) was added at room temperature and the mixture was shaken to give a yellow solution. After 5 min the solution was analyzed via NMR spectroscopy and the formation of [Ni(^{i}Pr$_2$Im^{Me})$_2$(η^2-HC≡CC$_3$H$_7$)] was detected.

^{1}H-NMR (400 MHz, C_6D_6, 25 °C): δ = 1.22 (t, 3H, $^3J_{HH}$ = 7.0 Hz, CH$_2$CH$_2$CH_3), 1.23 (d, 12H, $^3J_{HH}$ = 7.2 Hz, *i*Pr-CH_3), 1.29 (d, 12H, $^3J_{HH}$ = 7.2 Hz, *i*Pr-CH_3), 1.86 (s, 6H, NCCH_3CCH_3N), 1.88 (s, 6H, NCCH_3CCH_3N), 1.94 (tq, 2H, $^3J_{HH}$ = 7.2 Hz, CH$_2$CH_2CH$_3$), 3.13 (td, 2H, $^3J_{HH}$ = 7.2 Hz, $^4J_{HH}$ = 1.7 Hz, CH_2CH$_2$CH$_3$), 6.16 (sept., 2H, $^3J_{HH}$ = 7.2 Hz, *i*Pr-C*H*), 6.20 (sept., 2H, $^3J_{HH}$ = 7.2 Hz, *i*Pr-C*H*), 6.71 (t, 1H, $^4J_{HH}$ = 1.6 Hz, C≡C*H*).

^{13}C{^{1}H}-NMR (100 MHz, C_6D_6, 25 °C): δ = 10.4 (NC*C*H$_3$C*C*H$_3$N), 10.5 (NC*C*H$_3$C*C*H$_3$N), 14.8 (CH$_2$CH$_2$*C*H$_3$), 22.0 (^{i}Pr-*C*H$_3$), 22.2 (^{i}Pr-*C*H$_3$), 25.4 (CH$_2$*C*H$_2$CH$_3$), 32.8 (*C*H$_2$CH$_2$CH$_3$), 52.0 (^{i}Pr-*C*H), 52.1 (^{i}Pr-*C*H), 111.7 (C≡*C*H), 122.9 (N*C*CH$_3$*C*CH$_3$N), 138.1 (H$_7$C$_3$*C*≡C), 204.2 (N*C*N), 204.8 (N*C*N).

NMR Experiment for the Synthesis of [Ni(^{i}Pr$_2$Im^{Me})$_2$(η^2-HC≡CPh)] 10

A Young-NMR-Tube was charged with a solution of a 60:40 mixture of [Ni$_2$(^{i}Pr$_2$Im^{Me})$_4$(μ-(η^2:η^2)-COD)] **B** and [Ni(^{i}Pr$_2$Im^{Me})$_2$(η^4-COD)] **B'** (22.2 mg, 45.5 μmol) in 0.6 mL of C_6D_6. Phenylacetylene (5.00 μL, 4.65 mg; 45.5 μmol) was added at room temperature and the mixture was shaken to give an orange solution. After 5 min the solution was analyzed via NMR spectroscopy and the formation of [Ni(^{i}Pr$_2$Im^{Me})$_2$(η^2-HC≡CPh)] was detected.

^{1}H-NMR (400 MHz, C_6D_6, 25 °C): δ = 1.20 (d, 12H, $^3J_{HH}$ = 7.2 Hz, ^{i}Pr-CH_3), 1.24 (d, 12H, $^3J_{HH}$ = 7.2 Hz, ^{i}Pr-CH_3), 1.82 (s, 6H, NCCH_3CCH_3N), 1.87 (s, 6H, NCCH_3CCH_3N), 6.06 (sept, $^3J_{HH}$ = 7.2 Hz, 2H, ^{i}Pr-C*H*), 6.18 (sept, $^3J_{HH}$ = 7.2 Hz, 2H, ^{i}Pr-C*H*), 7.01 (m, 1H, aryl-CH_{para}), 7.22 (m, 2H, aryl-CH_{meta}), 7.63 (m, 2H, aryl-CH_{ortho}), 7.64 (s, 1H, C≡C*H*).

^{13}C{^{1}H-NMR (100 MHz, C_6D_6, 25 °C): δ = 10.5 (NC*C*H$_3$C*C*H$_3$N), 22.0 (^{i}Pr-*C*H$_3$), 22.2 (^{i}Pr-*C*H$_3$), 52.3 (^{i}Pr-*C*H), 52.5 (^{i}Pr-*C*H), 123.3 (N*C*CH$_3$*C*CH$_3$N), 123.4 (N*C*CH$_3$*C*CH$_3$N), 123.7 (aryl-

CH_{para}), 125.3 (C≡C*H*), 128.0 (aryl-CH_{meta}), 129.7 (aryl-CH_{ortho}), 138.6 (aryl-C_{ipso}), 202.3 (N*C*N), 202.5 (N*C*N).

Synthesis of [Ni(^{i}Pr$_2$Im^{Me})$_2$(η^2-HC≡C(*p*-Tol))] 11

p-Tolylacetylene (27.6 μL, 25.3 mg, 217 μmol) was added at room temperature to a solution of a 60:40 mixture of [Ni$_2$(^{i}Pr$_2$Im^{Me})$_4$(μ-(η^2:η^2)-COD)] **B** and [Ni(^{i}Pr$_2$Im^{Me})$_2$(η^4-COD)] **B'** (98 mg, 201 μmol) in 5 mL of benzene. The mixture was stirred for 1 h at room temperature and was then filtered over a pad of Celite. All volatiles were removed in vacuo and the remaining residue was suspended in 6 mL of hexane. The product was filtered off, washed with 3 mL of hexane and dried in vacuo to give a light brown powder (55 mg, 103 μmol, 51 %)

Elemental analysis $C_{31}H_{48}N_4Ni$ [535.45 g/mol] calculated: C 69.54, H 9.04, N 10.46 found: C 68.95, H 8.84, N 9.99.

^{1}H-NMR (400 MHz, C_6D_6, 25 °C): δ = 1.23 (d, 12H, $^3J_{HH}$ = 6.9 Hz, ^{i}Pr-CH_3), 1.24 (d, 12H, $^3J_{HH}$ = 6.9 Hz, ^{i}Pr-CH_3), 1.84 (s, 6H, NCCH_3CCH_3N), 1.87 (s, 6H, NCCH_3CCH_3N), 2.14 (s, 3H, aryl-CH_3), 6.08 (sept., 2H, $^3J_{HH}$ = 6.9 Hz, ^{i}Pr-C*H*), 6.19 (sept., 2H, $^3J_{HH}$ = 6.9 Hz, ^{i}Pr-C*H*), 7.04 (d, 2H, $^3J_{HH}$ = 7.8 Hz, aryl-CH_{meta}), 7.58 (d, 2H, $^3J_{HH}$ = 7.8 Hz, aryl-CH_{ortho}), 7.61 (s, 1H, C≡C*H*).

^{13}C{^{1}H}-NMR (100 MHz, C_6D_6, 25 °C): δ = 10.4 (NC*C*H$_3$C*C*H$_3$N), 10.5 (NC*C*H$_3$C*C*H$_3$N), 21.4 (aryl-*C*H$_3$), 22.0 (^{i}Pr-*C*H$_3$), 22.2 (^{i}Pr-*C*H$_3$), 52.2 (^{i}Pr-*C*H), 52.5 (^{i}Pr-*C*H), 123.2 (N*C*CH$_3$*C*CH$_3$N), 123.3 (N*C*CH$_3$*C*CH$_3$N), 123.9 (C≡*C*H), 128.7 (aryl-*C*H$_{meta}$), 129.9 (aryl-*C*H$_{ortho}$), 132.6 (aryl-*C*(CH$_3$)), 135.4 (aryl-C_{ipso}), 138.1 (p-Tol*C*≡C), 202.6 (N*C*N), 202.9 (N*C*N).

IR (ATR [cm^{-1}]): 2964 (m), 2930 (w), 2158 (w), 2031 (w), 1976 (w), 1687 (w), 1668 (m), 1597 (wv), 1492 (w), 1462 (w), 1407 (w), 1383 (w), 1345 (s), 1291 (s), 1274 (vs), 1210 (w), 1162 (vw), 1128 (w), 1099 (vw), 1062 (w), 1018 (w), 961 (vw), 929 (w), 904 (vw), 869 (m), 817 (w), 748 (m), 718 (w), 679 (m), 644 (vw), 570 (m), 555 (w), 527 (w), 462 (vw), 418 (vw).

Synthesis of [Ni(^{i}Pr$_2$Im^{Me})$_2$(η^2-HC≡C(4-^{t}Bu-C$_6$H$_4$))] 12

4-(*tert*-butyl)phenylacetylene (36.2 μL, 32.5 mg, 203 μmol) was added at room temperature to a solution of a 60:40 mixture of [Ni$_2$(^{i}Pr$_2$Im^{Me})$_4$(μ-(η^2:η^2)-COD)] **B** and [Ni(^{i}Pr$_2$Im^{Me})$_2$(η^4-COD)] **B'** (92 mg, 184 μmol) in 5 mL of benzene. The mixture was stirred for 1 h at room temperature and was then filtered over a pad of Celite. All volatiles were removed in vacuo and the remaining residue was washed with 1 mL of hexane and dried in vacuo to give an orange powder (77 mg, 133 μmol, 72 %).

Elemental analysis $C_{34}H_{54}N_4Ni$ [577.53 g/mol] calculated: C 70.71, H 9.43, N 9.70 found: C 71.92, H 9.46, N 8.63.

^{1}H-NMR (400 MHz, C_6D_6, 25 °C): δ = 1.23 (d, 12H, $^3J_{HH}$ = 7.0 Hz, ^{i}Pr-CH_3), 1,25 (s, 9H, C(CH_3)$_3$), 1.25 (d, 12H, $^3J_{HH}$ = 7.0 Hz, ^{i}Pr-CH_3), 1.84 (s, 6H, NCCH_3CCH_3N), 1.88 (s, 6H,

NCCH_3CCH_3N), 6.09 (sept., 2H, $^3J_{HH}$ = 7.0 Hz, ^{i}Pr-CH), 6.21 (sept., 2H, $^3J_{HH}$ = 7.0 Hz, ^{i}Pr-CH), 7.27 (d, 2H, $^3J_{HH}$ = 8.5 Hz, aryl-CH_{meta}), 7.61 (d, 2H, $^3J_{HH}$ = 8.5 Hz, aryl-CH_{ortho}), 7.62 (s, 1H, C≡CH).

^{13}C{^{1}H}-NMR (100 MHz, C_6D_6, 25 °C): δ = 10.4 (NCCH$_3$CCH$_3$N), 10.5 (NCCH$_3$CCH$_3$N), 22.0 (^{i}Pr-CH$_3$), 22.1 (^{i}Pr-CH$_3$), 31.7 (C(CH$_3$)$_3$), 34.5 (C(CH$_3$)$_3$), 52.2 (^{i}Pr-CH), 52.5 (^{i}Pr-CH), 123.2 (NCCH$_3$$CCH_3$N), 123.3 (N$CCH_3$$CCH_3$N), 123.9 (C≡$C$H), 124.8 (aryl-$CH_{meta}$), 129.5 (aryl-$CH_{ortho}$), 135.6 (aryl-$C_{ipso}$), 138.0 ($H_4C_6$$C$≡C), 146.0 (aryl-$CH_{para}$), 202.6 (N$C$N), 202.9 (N$C$N).

IR (ATR [cm^{-1}]): 2964 (m), 2869 (w), 1683 (m), 1596 (vw), 1492 (w), 1460 (w), 1406 (w), 1382 (w), 1346 (vs), 1292 (m), 1275 (s), 1209 (w), 1163 (w), 1132 (w), 1101 (w), 1063 (vw), 1019 (m), 961 (vw), 905 (vw), 870 (w), 839 (w), 826 (w), 804 (w), 753 (vw), 688 (m), 677 (m), 649 (vw), 563 (w), 549 (w), 468 (vw).

Synthesis of [Ni(^{i}Pr$_2$Im^{Me})$_2$(η^2-HC≡CCOOMe)] 13

Methyl propiolate (27.2 μL, 27.4 mg, 325 μmol) was added at room temperature to a solution of a 60:40 mixture of [Ni$_2$(^{i}Pr$_2$Im^{Me})$_4$(μ-(η^2:η^2)-COD)] **B** and [Ni(^{i}Pr$_2$Im^{Me})$_2$(η^4-COD)] **B'** (154 mg, 316 μmol) in 5 mL of toluene. The mixture was stirred for 2 h at room temperature and was then filtered over a pad of Celite. All volatiles were removed in vacuo and the remaining residue was suspended in 6 mL of hexane. The product was filtered off, washed with 3 mL of hexane and dried in vacuo to give an orange powder (66 mg, 131 μmol, 42 %).

Elemental analysis $C_{26}H_{44}N_4NiO_2$ [503.36 g/mol] calculated: C 62.04, H 8.81, N 11.13 found: C 61.94, H 8.91, N 10.90.

^{1}H-NMR (400 MHz, C_6D_6, 25 °C): δ = 1.13 (d, 12H, $^3J_{HH}$ = 7.1 Hz, *i*Pr-CH_3), 1.26 (d, 12H, $^3J_{HH}$ = 7.1 Hz, *i*Pr-CH_3), 1.79 (s, 6H, NCCH_3CCH_3N), 1.80 (s, 6H, NCCH_3CCH_3N), 3.66 (s, 3H, COOCH_3), 6.01 (sept., 2H, $^3J_{HH}$ = 7.1 Hz, *i*Pr-CH), 6.07 (sept., 2H, $^3J_{HH}$ = 7.1 Hz, *i*Pr-CH), 7.64 (s, 1H, C≡CH).

^{13}C{^{1}H}-NMR (100 MHz, C_6D_6, 25 °C): δ = 10.3 (NCCH$_3$CCH$_3$N), 10.4 (NCCH$_3$CCH$_3$N), 21.8 (^{i}Pr-CH$_3$), 22.1 (^{i}Pr-CH$_3$), 50.2 (COOCH$_3$), 52.5 (^{i}Pr-CH), 52.8 (^{i}Pr-CH), 123.7 (NCCH$_3$$CCH_3$N), 123.8 (N$CCH_3$$CCH_3$N), 129.6 (C≡$C$H), 131.9 (MeOOC$C$≡$C$), 173.1 ($C$OOCH$_3$), 198.6 (N$C$N), 198.8 (N$C$N).

IR (ATR [cm^{-1}]): 3018 (wv), 2968 (w), 2934 (w), 2162 (wv), 2056 (wv), 1702 (m), 1634 (m), 1464 (w), 1407 (m), 1381 (w), 1384 (w), 1300 (w), 1281 (w), 1213 (vw), 1157 (s), 1130 (w), 1102 (w), 1019 (w), 963 (vw), 896 (w), 849 (w), 777 (w), 754 (w), 735 (w), 688 (w), 665 (vw), 555 (w), 464 (vw), 430 (vw), 410 (wv).

Synthesis of 11a

p-Tolylacetylene (43.6 *µ*L, 39.9 mg, 343 *µ*mol) was added at room temperature to a solution of a 60:40 mixture of [Ni_2(^{i}Pr_2Im^{Me})$_4$(*µ*-(η^2:η^2)-COD)] **B** and [Ni(^{i}Pr_2Im^{Me})$_2$(η^4-COD)] **B'** (148 mg, 304 *µ*mol) in 6 mL of toluene. After 1 h at room temperature all volatiles were removed in vacuo to remove cyclooctadiene and residual alkyne. The remaining residue was dissolved again in 6 mL of toluene and the solution was stirred for 72 h at 60 °C. The mixture was then filtered over a pad of Celite, all volatiles were removed in vacuo and the remaining residue was suspended in 3 mL of hexane. The resulting precipitate was filtered off and the remaining solution was stored at -30 °C for 6 days. The supernatant solution was removed via syringe and the residue was dried in vacuo to give a red crystalline powder (30 mg, 56.0 *µ*mol, 18 %).

The obtained crystals of **11a** were also suitable for single-crystal X-ray diffraction.

^{1}H-NMR (400 MHz, C_6D_6, 25 °C): δ = 0.94 (d, 3H, $^3J_{HH}$ = 7.2 Hz, *i*Pr-C*H$_3$*), 1.21 (d, 6H, $^3J_{HH}$ = 7.2 Hz, *i*Pr-C*H$_3$*), 1.36 (d, 3H, $^3J_{HH}$ = 6.5 Hz, NCHCH$_2$C*H*$_3$), 1.48 (br, 3H, *i*Pr-C*H$_3$*), 1.62 (s, 3H, NCC*H*$_3$CC*H*$_3$N), 1.73 (s, 6H, NCC*H*$_3$CC*H*$_3$N), 1.83 (s, 3H, NCC*H*$_3$CC*H*$_3$N), 2.19 (s, 3H, aryl-C*H$_3$*), 2.64 (ddd, 1H, $^2J_{HH}$ = 3.5 Hz, $^3J_{HH}$ = 3.5 Hz, $^3J_{HH}$ = 13.5 Hz, C=CHC*H*$_2$) 2.78 (ddd, 1H, $^2J_{HH}$ = 3.5 Hz, $^3J_{HH}$ = 12.0 Hz, $^3J_{HH}$ = 13.5 Hz, C=CHC*H*$_2$), 2.91 (ddd, 1H, $^3J_{HH}$ = 3.5 Hz, $^3J_{HH}$ = 9.8 Hz, $^3J_{HH}$ = 12.0 Hz, C=C*H*CH$_2$), 3.85 (d, 1H, $^3J_{HH}$ = 9.8 Hz, *p*-Tol*H*C=C), 3.99 (m, 1H, NC*H*CH$_2$CH$_3$), 5.48 (br, 1H, *i*Pr-C*H*), 5.60 (sept. 1H, $^3J_{HH}$ = 7.2 Hz, *i*Pr-C*H*), 5.80 (br, 1H, *i*Pr-C*H*), 7.00 (d, 2H, $^3J_{HH}$ = 7.8 Hz, aryl-C*H*$_{meta}$), 7.32 (d, 2H, $^3J_{HH}$ = 7.8 Hz, aryl-C*H*$_{ortho}$).

^{13}C{^{1}H}-NMR (100 MHz, C_6D_6, 25 °C): δ = 8.7 (NC*C*H$_3$C*C*H$_3$N), 10.3 (NC*C*H$_3$C*C*H$_3$N), 10.8 (NC*C*H$_3$C*C*H$_3$N), 21.3 (aryl-*C*H$_3$), 21.5 (NCHCH$_2$*C*H$_3$), 22.1 (^{i}Pr-*C*H$_3$), 22.1 ^{i}Pr-*C*H$_3$), 22.2 (^{i}Pr-*C*H$_3$), 22.5 (^{i}Pr-*C*H$_3$), 22.7 (^{i}Pr-*C*H$_3$), 34.1(C=*C*HCH$_2$), 40.2 (C=CH*C*H$_2$), 51.3 (^{i}Pr-*C*H), 51.9 (PhH*C*=C), 52.7 (^{i}Pr-*C*H), 54.1 (N*C*HCH$_2$CH$_3$), 120.9 (N*C*CH$_3$*C*CH$_3$N), 122.8 (N*C*CH$_3$*C*CH$_3$N), 123.9 (aryl-*C*H$_{meta}$), 124.4 (N*C*CH$_3$*C*CH$_3$N), 126.2 (aryl-*C*(CH$_3$)), 129.2 (aryl-*C*H$_{ortho}$) 150.5 (aryl-*C*$_{ipso}$), 191.7 (N*C*N), 204.5 (N*C*N).

NMR Experiment for the Synthesis of 12a

A Young-NMR-Tube was charged with a solution of a 60:40 mixture of [Ni_2(^{i}Pr_2Im^{Me})$_4$(*µ*-(η^2:η^2)-COD)] **B** and [Ni(^{i}Pr_2Im^{Me})$_2$(η^4-COD)] **B'** (15.0 mg, 30.5 *µ*mol) in 0.6 mL of C_6D_6. 4-(*tert*-butyl)phenylacetylene (4.02 *µ*L, 3.68 mg, 31.7 *µ*mol) was added at room temperature and the mixture was shaken to give a red solution. After 72 h at 60 °C the solution was analyzed via NMR spectroscopy and the formation of [Ni(^{i}Pr_2Im^{Me})$_2$(η^2-^{i}PrHC=CH(4-^{t}Bu-C_6H_4))] **12a** was detected.

Synthesis of [Ni(Mes$_2$Im)$_2$(η^2-MeC≡CMe) 14

2-Butyne (100 *µL*, 69.0 mg, 1.28 mmol) was added at 0 °C to a suspension of [Ni(Mes$_2$Im)$_2$] **2** (110 mg, 165 *µ*mol) in 8 mL of hexane. A yellow precipitate was formed, which was filtered off immediately and dried in vacuo to give a yellow powder (45.0 mg, 62.5 *µ*mol, 38 %).

Yellow crystals of [Ni(Mes$_2$Im)$_2$(η^2-MeC≡CMe) **14** suitable for single-crystal X-ray diffraction were obtained from a saturated solution in hexane at -30 °C.

Elemental analysis $C_{46}H_{54}N_4Ni$ [721.66 g/mol] calculated: C 76.56, H 7.54, N 7.76 found: C 74.25, H 7.50, N 7.51.

^{1}H-NMR (400 MHz, THF-d$_8$, -80 °C): δ = 1.74 (s, 6H, H_3CC≡CH_3), 1.78 (s, 12H, aryl$_{NHC}$-CH_{3ortho}), 2.04 (s, 12H, aryl$_{NHC}$-CH_{3ortho}), 2.36 (s, 12H, aryl$_{NHC}$-CH_{3para}), 6.52 (s, 4H, aryl$_{NHC}$-CH_{meta}), 6.67 6.52 (s, 4H, NC*H*C*H*N), 6.67 (s, 4H, aryl$_{NHC}$-CH_{meta}).

^{13}C{^{1}H-NMR (100 MHz, THF-d$_8$, -80 °C): δ = 13.7 (H$_3$*C*C≡*C*CH$_3$), 19.5 (aryl$_{NHC}$-*C*H$_{3ortho}$), 19.6 (aryl$_{NHC}$-*C*H$_{3ortho}$), 21.4 (aryl$_{NHC}$-*C*H$_{3para}$), 118.6 (*C*≡*C*), 122.3 (N*C*H*C*HN), 129.0 (aryl$_{NHC}$-*C*H$_{meta}$), 135.9 (aryl$_{NHC}$-*C*CH$_{3ortho/para}$), 136.0 (aryl$_{NHC}$-*C*CH$_{3ortho/para}$), 136.1 (aryl$_{NHC}$-*C*CH$_{3ortho/para}$), 139.3 (aryl$_{NHC}$-*C*$_{ipso}$), 207.0 (N*C*N).

IR (ATR [cm^{-1}]): 2911 (vw), 2837 (vw), 1808 (vw), 1483 (m), 1434 (w), 1375 (m), 1245 (vs), 1158 (vw), 1092 (vw), 1060 (m), 1031 (m), 966 (w), 914 (m), 847 (s), 713 (w), 679 (vs), 630 (vw), 591 (w), 572 (m), 446 (vw), 425 (m).

Synthesis of [Ni(Mes$_2$Im)$_2$(η^2-MeOOCC≡CCOOMe) 15

Dimethyl acetylenedicarboxylate (28.9 *µ*L, 33.5 mg, 236 *µ*mol) was added at 0 °C to a suspension of [Ni(Mes$_2$Im)$_2$] **2** (121 mg, 181 *µ*mol) in 5 mL of pentane. Immediately a brown precipitate was formed and the mixture was then stirred for 1 h at 0 °C. The supernatant solution was removed via syringe and the residue was dried in vacuo to give a light brown powder (140 mg, 173 *µ*mol, 96 %).

Brown crystals of [Ni(Mes$_2$Im)$_2$(η^2-MeOOCC≡CCOOMe) **15** suitable for single-crystal X-ray diffraction were obtained by layering a saturated benzene solution with hexane at room temperature.

Elemental analysis $C_{48}H_{54}N_4NiO_4$ [809.68 g/mol] calculated: C 71.20, H 6.72, N 6.92; found: C 70.80, H 6.90, N 6.75.

^{1}H-NMR (400 MHz, C_6D_6, 25 °C): δ = 2.06 (s, 24H, $aryl_{NHC}$-CH_{3ortho}), 2.23 (s, 12H, $aryl_{NHC}$-CH_{3para}), 3.47 (s, 6H, COOCH_3), 6.11 (s, 4H, NC*H*C*H*N), 6.61 (s, 8H, $aryl_{NHC}$-CH_{meta}).

^{13}C{^{1}H-NMR (100 MHz, C_6D_6, 25 °C): δ = 19.2 ($aryl_{NHC}$-CH_{3ortho}), 21.3 ($aryl_{NHC}$-CH_{3para}), 50.4 (COO*C*H_3), 123.4 (N*C*H*C*HN), 129.3 ($aryl_{NHC}$-CH_{meta}), 136.0 ($aryl_{NHC}$-*C*CH_{3ortho}), 136.6 ($aryl_{NHC}$-*C*CH_{3para}), 136.7 (*C*≡*C*), 138.4 ($aryl_{NHC}$-C_{ipso}), 165.9 (*C*OOMe), 198.2 (N*C*N).

IR (ATR [cm^{-1}]): 3161 (vw), 3116 (w), 2981 (w), 2914 (w), 2853 (w), 1713 (m), 1680 (s), 1590 (w), 1484 (m), 1441 (m), 1386 (m), 1290 (m), 1258 (m), 1193 (s), 1180 (s), 1107 (m), 1036 (m), 1015 (m), 919 (m), 844 (s), 740 (m), 693 (m), 569 (m), 424 (m).

Synthesis of [Ni(Mes$_2$Im)$_2$(η^2-PhC≡CMe) 16

1-Phenyl-1-propyne (29.3 *µL*, 27.0 mg, 232 *µ*mol) was added at 0 °C to a suspension of [Ni(Mes$_2$Im)$_2$] **2** (155 mg, 232 *µ*mol) in 6 mL of pentane. Immediately an orange precipitate was formed, and the mixture was then stirred for 1 h at 0 °C. The supernatant solution was removed via syringe and the residue was dried in vacuo to give an orange powder (155 mg, 198 *µ*mol, 85 %).

Orange crystals of [Ni(Mes$_2$Im)$_2$(η^2-PhC≡CMe) **16** suitable for single-crystal X-ray diffraction were obtained from a saturated solution in pentane at -30 °C.

Elemental analysis $C_{51}H_{56}N_4Ni$ [783.73 g/mol] calculated: C 78.16, H 7.20, N 7.15; found: C 77.89, H 7.53, N 7.05.

^{1}H-NMR (500 MHz, THF-d$_8$, -80 °C): δ = 1.77 (s, 12H, $aryl_{NHC}$-CH_{3ortho}), 1.82 (s, 6H, $aryl_{NHC}$-CH_{3ortho}), 2.10 (s, 6H, $aryl_{NHC}$-CH_{3ortho}), 2.14 (s, 3H, C≡CCH_3), 2.34 (s, 6H, $aryl_{NHC}$-CH_{3para}), 2.37 (s, 6H, $aryl_{NHC}$-CH_{3para}), 6.50 (s, br, 2H, $aryl_{NHC}$-CH_{meta}), 6.53 (s, br, 4H, $aryl_{NHC}$-CH_{meta}), 6.65 (d, 2H, $^3J_{HH}$ = 7.8 Hz, aryl-CH_{ortho}), 6.70 (s, br, 2H, $aryl_{NHC}$-CH_{meta}), 6.73 (m, 1H, aryl-CH_{para}), 6.77 (s, 2H, NC*H*C*H*N), 6.82 (s, 2H, NC*H*C*H*N), 6.91 (m, 2H, aryl-CH_{meta}).

^{13}C{^{1}H-NMR (126 MHz, THF-d$_8$, -80 °C): δ = 16.9 (C≡C*C*H_3), 19.5 ($aryl_{NHC}$-CH_{3ortho}), 19.6 ($aryl_{NHC}$-CH_{3ortho}), 19.9 ($aryl_{NHC}$-CH_{3ortho}), 20.5 ($aryl_{NHC}$-CH_{3ortho}), 21.3 ($aryl_{NHC}$-CH_{3para}), 21.4 ($aryl_{NHC}$-CH_{3para}), 122.6 (aryl-CH_{para}), 123.0 (N*C*H*C*HN), 123.9 (Ph*C*≡C), 127.0 (aryl-CH_{meta}), 129.0 ($aryl_{NHC}$-CH_{meta}), 129.1 ($aryl_{NHC}$-CH_{meta}), 129.3 ($aryl_{NHC}$-CH_{meta}), 129.6 ($aryl_{NHC}$-CH_{meta}), 131.0 (aryl-CH_{ortho}), 134.2 (aryl-C_{ipso}), 135.6 (C≡*C*Me), 135.8 ($aryl_{NHC}$-*C*CH_{3ortho}), 136.0($aryl_{NHC}$-*C*CH_{3ortho}), 136.1 ($aryl_{NHC}$-*C*CH_{3ortho}), 136.2 ($aryl_{NHC}$-*C*CH_{3ortho}), 136.3 ($aryl_{NHC}$-*C*CH_{3para}), 136.4 ($aryl_{NHC}$-*C*CH_{3para}), 139.0 ($aryl_{NHC}$-C_{ipso}), 139.1 ($aryl_{NHC}$-C_{ipso}), 205.8 (N*C*N), 206.0 (N*C*N).

IR (ATR [cm^{-1}]): 2949 (w), 2912 (w), 2837 (w), 1756 (m), 1585 (m), 1480 (s), 1434 (m), 1375 (s), 1262 (vs), 1245 (vs), 1158 (w), 1093 (w), 1063 (m), 1033 (m), 967 (w), 916 (m), 846 (vs), 757 (m), 716 (m), 700 (m), 682 (vs), 653 (m), 571 (m), 521 (w), 423 (m).

Synthesis of [Ni(Mes$_2$Im)$_2$(η^2-HC≡C(4-^{t}Bu-C$_6$H$_4$))] 17

4-(*tert*-Butyl)phenylacetylene (44.5 µL, 39.1 mg, 247 µmol) was added at 0 °C to a suspension of [Ni(Mes$_2$Im)$_2$] **2** (165 mg, 247 µmol) in 6 mL of pentane. Immediately an orange precipitate was formed, and the mixture was then stirred for 1 h at 0 °C. The supernatant solution was removed via syringe and the residue was dried in vacuo to give an orange powder (166 mg, 201 µmol, 81 %).

Red crystals of [Ni(Mes$_2$Im)$_2$(η^2-HC≡C(4-^{t}Bu-C$_6$H$_4$))] **17** suitable for single-crystal X-ray diffraction were obtained from a saturated solution in hexane at -30 °C.

Elemental analysis $C_{54}H_{62}N_4Ni$ [825.81 g/mol] calculated: C 78.54, H 7.57, N 6.78; found: C 78.13, H 8.16, N 5.75.

^{1}H-NMR (400 MHz, THF-d$_8$, -80 °C): δ = 1.22 (s, 9H, ^{t}Bu-C*H*$_3$), 1.76 (s, br, 6H, aryl$_{NHC}$-C*H*$_{3ortho}$), 1.82 (s, br, 6H, aryl$_{NHC}$-C*H*$_{3ortho}$), 1.85 (s, br, 12H, aryl$_{NHC}$-C*H*$_{3ortho}$), 2.34 (s, 12H, aryl$_{NHC}$-C*H*$_{3para}$), 6.11 (s, 1H, *H*C≡C), 6.59 (s, br, 2H, aryl$_{NHC}$-C*H*$_{meta}$), 6.61 (s, br, 2H, aryl-C$_6$*H*$_4$), 6.63 (s, br, 6H, aryl$_{NHC}$-C*H*$_{meta}$), 6.78 (s, 2H, NC*H*C*H*N), 6.84 (s, 2H, NC*H*C*H*N), 6.94 (d, 2H, $^3J_{HH}$ = 8.5 Hz, aryl-C$_6$*H*$_4$).

^{13}C{^{1}H-NMR (100 MHz, THF-d$_8$, -80 °C): δ = 19.2 (aryl$_{NHC}$-*C*H$_{3ortho}$), 19.7 (aryl$_{NHC}$-*C*H$_{3ortho}$), 20.8 (aryl$_{NHC}$-*C*H$_{3ortho}$), 21.1 (aryl$_{NHC}$-*C*H$_{3para}$), 21.3 (aryl$_{NHC}$-*C*H$_{3para}$), 31.6 (C(*C*H$_3$)$_3$), 34.7 (*C*(CH$_3$)$_3$), 122.6 (N*C*H*C*HN), 122.8 (H*C*≡C), 123.0 (N*C*H*C*HN), 123.8 (*C*$_6$H$_4$), 129.1 (aryl$_{NHC}$-*C*H$_{meta}$), 129.2 (aryl$_{NHC}$-*C*H$_{meta}$), 129.6 (aryl$_{NHC}$-*C*H$_{meta}$), 130.7 (aryl-*C*$_{ipso}$), 130.9 (*C*$_6$H$_4$), 131.5 (C≡*C*(C$_6$H$_4$)), 135.5 (aryl$_{NHC}$-*C*CH$_{3para}$), 136.4 (aryl$_{NHC}$-*C*CH$_{3ortho}$), 136.5 (aryl$_{NHC}$-*C*CH$_{3ortho}$), 136.8 (aryl$_{NHC}$-*C*CH$_{3ortho}$), 139.0 (aryl$_{NHC}$-*C*$_{ipso}$), 139.4 (aryl$_{NHC}$-*C*$_{ipso}$), 145.6 (aryl-*C*(^{t}Bu)), 202.2 (N*C*N), 206.5 (N*C*N).

IR (ATR [cm^{-1}]): 2953 (m), 2917 (m), 2856 (m), 1701 (w), 1596 (w), 1548 (s), 1485 (m), 1373 (m), 1291 (m), 1258 (vs), 1242 (vs), 1158 (w), 1086 (s), 1063 (m), 915 (m), 848 (s), 837 (s), 725 (m), 680 (vs), 571 (s), 546 (m), 422 (m).

Synthesis of [Ni(Mes$_2$Im)$_2$(η^2-HC≡CCOOMe)] 18

Methyl propiolate (20.7 µL, 20.8 mg, 247 µmol) was added at 0 °C to a suspension of [Ni(Mes$_2$Im)$_2$] **2** (127 mg, 190 µmol) in 5 mL of pentane. Immediately a brown precipitate was

formed, and the mixture was then stirred for 1 h at 0 °C. The supernatant solution was removed via syringe and the residue was dried in vacuo to give a light brown powder (130 mg, 173 μmol, 91 %).

Elemental analysis $C_{46}H_{52}N_4NiO_2$ [751.64 g/mol] calculated: C 73.51, H 6.97, N 7.45; found: C 73.53, H 7.11, N 7.27.

^{1}H-NMR (400 MHz, C_6D_6, 25 °C): δ = 1.92 (s, 12H, aryl$_{NHC}$-CH_{3ortho}), 2.12 (s, 12H, aryl$_{NHC}$-CH_{3ortho}), 2.23 (s, 6H, aryl$_{NHC}$-CH_{3para}), 2.26 (s, 6H, aryl$_{NHC}$-CH_{3para}), 3.50 (s, 3H, COOCH_3), 6.06 (s, 2H, NC*H*C*H*N), 6.18 (s, 2H, NC*H*C*H*N), 6.60 (s, 4H, aryl$_{NHC}$-CH_{meta}), 6.66 (s, 4H, aryl$_{NHC}$-CH_{meta}), 6.94 (s, 1H, *H*C≡C).

^{13}C{^{1}H-NMR (100 MHz, C_6D_6, 25 °C): δ = 19.0 (aryl$_{NHC}$-*C*H_{3ortho}), 19.1 (aryl$_{NHC}$-*C*H_{3ortho}), 21.3 (aryl$_{NHC}$-*C*H_{3para}), 50.2 (COO*C*H_3), 122.3 (N*C*H*C*HN), 122.6 (N*C*H*C*HN), 129.2 (aryl$_{NHC}$-*C*H_{meta}), 134.6 (H*C*≡C), 135.5 (aryl$_{NHC}$-*C*CH_{3ortho}), 136.3 (aryl$_{NHC}$-*C*CH_{3para}), 136.6 (C≡*C*COOMe), 138.5 (aryl$_{NHC}$-C_{ipso}), 138.8 (aryl$_{NHC}$-C_{ipso}), 165.8 (*C*OOMe), 201.8 (N*C*N), 202.4 (N*C*N).

IR (ATR [cm^{-1}]): 2947 (w), 2912 (w), 2851 (w), 1711 (m), 1656 (m), 1484 (m), 1380 (m), 1284 (m), 1274 (m), 1256 (m), 1160 (vs), 1070 (m), 1034 (m), 968 (w), 917 (m), 846 (s), 724 (m), 689 (s), 577 (m), 424 (m).

General Procedure for the Synthesis of substituted benzene derivatives

In a Young-NMR-Tube [Ni(Mes$_2$Im)$_2$] **2** (10 mg, 15.0 μmol, 5 mol%) was dissolved in 0.7 mL of C_6D_6. The alkyne (1.0 equiv.) was then added to the solution. The reaction mixture was heated to 60 °C and the reaction progress was monitored hourly by ^{1}H NMR spectroscopy. After the alkyne consumption was complete the reaction mixture was poured in air into 5 mL of benzene and was then filtered over a pad of silica gel. The filtrate was evaporated in vacuo and the products were determined by NMR spectroscopy and GC/MS.

Hexamethylbenzene

^{1}H-NMR (400 MHz, C_6D_6, 25 °C): δ = 2.13 (s, 18H, C_6(CH_3)$_6$).

^{13}C{^{1}H-NMR (100 MHz, C_6D_6, 25 °C): δ = 16.9 (C_6(*C*H_3)$_6$), 131.8 (*C*$_6$(CH_3)$_6$).

GC/MS Ret.: 7.72; m/z: 162 $[M]^+$.

Hexapropylbenzene

^{1}**H-NMR** (400 MHz, C_6D_6, 25 °C): δ = 1.02 (t, 18H, $^{3}J_{HH}$ = 7.4 Hz, $CH_2CH_2CH_3$), 1.62 (m, 12H, $CH_2CH_2CH_3$), 2.65 (m, 12H, $CH_2CH_2CH_3$).

^{13}**C{^{1}H-NMR** (100 MHz, C_6D_6, 25 °C): δ = 15.4 ($CH_2CH_2CH_3$), 25.7 ($CH_2CH_2CH_3$), 32.7 ($CH_2CH_2CH_3$), 137.0 (aryl-C_q).

GC/MS Ret.: 10.70; m/z: 330 $[M]^+$.

Hexamethyl-benzenehexacarboxylate

^{1}**H-NMR** (400 MHz, C_6D_6, 25 °C): δ = 3.43 (s, 18H, OCH_3).

^{13}**C{^{1}H-NMR** (100 MHz, C_6D_6, 25 °C): δ = 52.9 (OCH_3), 134.5 (aryl-C_q), 165.5 (*C*OOMe).

GC/MS Ret.: 13.39; m/z: 395 $[M\text{-}OMe]^+$.

Trimethyl-1,2,4-benzenetricarboxylate and Trimethyl-1,3,5-benzenetricarboxylate

<u>Trimethyl-1,2,4-benzenetricarboxylate (85%)</u>

^{1}**H-NMR** (400 MHz, C_6D_6, 25 °C): δ = 3.38 (s, 3H, OCH_3), 3.44 (s, 3H, OCH_3), 3.53 (s, 3H, OCH_3), 7.37 (d, 1H, $^{3}J_{HH}$ = 8.0 Hz, aryl-6-C*H*), 7.90 (dd, 1H, $^{3}J_{HH}$ = 8.0 Hz, $^{4}J_{HH}$ = 1.6 Hz aryl-5-C*H*), 8.52 (d, 1H, $^{4}J_{HH}$ = 1.6 Hz, aryl-3-C*H*).

^{13}**C{^{1}H-NMR** (100 MHz, C_6D_6, 25 °C): δ = 51.9 (OCH_3), 52.3 (OCH_3), 52.4 (OCH_3), 129.1 (aryl-6-*C*H), 130.5 (aryl-3-*C*H), 132.3 (aryl-5-*C*H), 132.3 (aryl-2-C_q), 132.7 (aryl-1-C_q), 137.0 (aryl-4-C_q), 165.0 (4-*C*OOMe), 166.6 (2-*C*OOMe), 167.4 (1-*C*OOMe).

GC/MS Ret.: 9.92; m/z: 252 $[M]^+$.

<u>Trimethyl-1,3,5-benzenetricarboxylate (15%)</u>

^{1}**H-NMR** (400 MHz, C_6D_6, 25 °C): δ = 3.41 (s, 9H, OCH_3), 9.02 (s, 3H, aryl-2,4,6-C*H*).

^{13}**C{^{1}H-NMR** (100 MHz, C_6D_6, 25 °C): δ = 52.0 (OCH_3), 131.7 (aryl-1,3,5-C_q), 134.6 (aryl-2,4,6-*C*H), 165.1 (1,3,5-*C*OOMe).

GC/MS Ret.: 10.26; m/z: 252 $[M]^+$.

1,2,4-Triphenylbenzene and 1,3,5-Triphenylbenzene

GC/MS Ret.: 13.64, 14.52; m/z: 306 $[M]^+$.

1,2,4-Tripropylbenzene and 1,3,5-Tripropylbenzene

GC/MS Ret.: 7.77, 7.87; m/z: 204 $[M]^+$.

Hexaphenylbenzene

In a young NMR-Tube $[Ni(Mes_2Im)_2]$ **2** (3.00 mg, 1 mol%) and diphenylacetylene (80.1 mg, 449 *µ*mol) were dissolved in 0.7 mL C_6D_6. The reaction mixture was sonicated for five minutes whereby a colorless solid precipitated. The supernatant solution was removed via syringe and the residue was washed with hexane and dried in vacuo to give an off-white powder (71.0 mg, 133 *µ*mol, 88 %).

Elemental analysis $C_{42}H_{30}$ [534.70 g/mol] calculated: C 94.34, H 5.66; found: C 94.28, H 5.81.

^{1}H-NMR (400 MHz, C_6D_6, 25 °C): δ = 6.73 (m, 6H, aryl-C_6H_{5para}), 6.83 (m, 12H, aryl-C_6H_{5meta}), 7.12 (m, 12H, aryl-C_6H_{5ortho}).

^{13}C{^{1}H-NMR (100 MHz, C_6D_6, 25 °C): δ = 125.9 (aryl-C_6H_{5para}), 127.3 (aryl-C_6H_{5meta}), 132.0 (aryl-C_6H_{5ortho}), 141.2 (aryl-C_q), 141.3 (aryl-C_q).

GC/MS Ret.: 14.33; m/z: 534 $[M]^+$.

2) Crystallographic Details

Crystals were immersed in a film of perfluoropolyether oil on a glass fiber MicroMount^{TM} (MiTeGen) and transferred to a Bruker D8 Apex-2 diffractometer with CCD area detector and graphite-monochromated Mo-K_{α} radiation equipped with an Oxford Cryosystems low-temperature device or a Rigaku XtaLAB Synergy-DW diffractometer with HyPix-6000HE detector and monochromated Cu-K_{α} equipped with an Oxford Cryo 800 cooling unit. Data were collected at 100 K. The images were processed with the Bruker or Crysalis software packages and equivalent reflections were merged. Corrections for Lorentz-polarization effects and absorption were performed if necessary and the structures were solved by direct methods. Subsequent difference Fourier syntheses revealed the positions of all other non-hydrogen atoms. The structures were solved by using the ShelXTL software package.[6] All non-hydrogen atoms were refined anisotropically. Hydrogen atoms were usually assigned to idealized positions and were included in structure factors calculations. In case of the molecular structure **11a** the squeeze function was used to include disordered solvent molecules into the model.

Crystallographic data for the structures reported in this paper have been deposited with the Cambridge Crystallographic Data Centre as supplementary publication no.s CCDC 2100093-CCDC 2100101: CCDC 2100093 (**15**), CCDC 2100094 (**5**), CCDC 2100095 (**14**), CCDC 2100096 (**3**), CCDC 2100097 (**B**), CCDC 2100098 (**17**), CCDC 2100099 (**11a**), CCDC 2100100 (**16**), CCDC 2100101 (**7**). Copies of the data can be obtained free of charge on application to CCDC.

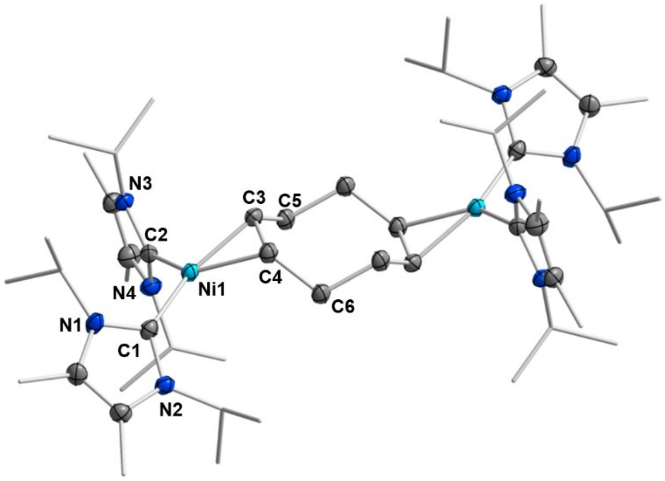

Figure S1. Molecular structure of [$Ni_2(^{i}Pr_2Im^{Me})_4(\mu$-$(\eta^2:\eta^2)$-COD)] **B** in the solid state (ellipsoids were set at the 50% probability level). The hydrogen atoms were omitted for clarity. Selected bond lengths [Å] and angles [°] of **B**: Ni1–C1 1.9117(19), Ni1–C2 1.9122(19), Ni1–C3 1.9749(19), Ni1–C4 1.9734(19), C3–C4 1.428(2), C3–C5 1.515(3), C4–C6 1.513(3); C1–Ni1–C2 118.65(8), C1–Ni1–C3 138.56(8), C1–Ni1–C4 96.15(8), C2–Ni1–C3 102.72(8), C2–Ni1–C4 145.08(8), C3–Ni1–C4 42.42(7).

Crystal data for B: $C_{52}H_{92}N_8Ni_2$, M_r = 946.72, yellow block, 0.204 × 0.138 × 0.106 mm, triclinic space group P-1, a = 11.6384(3) Å, b = 14.6564(4) Å, c = 18.2667(3) Å, α = 97.575(2)°, β = 105.266(2)°, γ = 112.868(2)°, V = 2671.86(12) Å^3, T = 99.9(6) K, Z = 2, $\rho_{calcd.}$ = 1.177 g cm^{-3}, μ = 1.171 mm^{-1}, F(000) = 1032, 54303 reflections in h(–14/13), k(–16/18), l(–22/22) measured in the range 2.599° < θ < 74.503°, 10876 independent reflections, 10876 observed reflections [I >2σ(I)], 707 parameters, 764 restraints; all data: R_1 = 0.0656 and wR_2 = 0.1727, I >2σ(I): R_1 = 0.0602 and wR_2 = 0.1661, *Goof* 1.055, largest difference peak/hole 0.786/–0.760 e Å^{-3}.

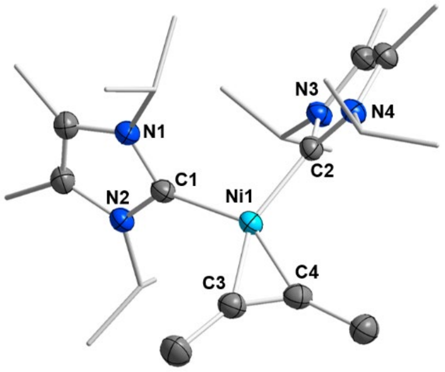

Figure S2. Molecular structure of [Ni(^{i}Pr$_2$Im^{Me})$_2$(η^2-MeC≡CMe)] **3** in the solid state (ellipsoids set at the 50% probability level). The hydrogen atoms were omitted for clarity. Selected bond lengths [Å] and angles [°] of **3**: Ni1–C1 1.9097(14), Ni1–C2 1.9239(14), Ni1–C3 1.8805(15), Ni1–C4 1.9026(14), C3–C4 1.285(2), C1–Ni1–C2 102.42(6), C1–Ni1–C3 105.16(6), C2–Ni1–C4 112.93(6), C3–Ni1–C4 39.70(6), plane (C1–Ni1–C2) – plane (C3–Ni1–C4) 8.32(8).

Crystal data for 3: $C_{26}H_{46}N_4Ni$, M_r = 473.38, yellow block, 0.328 × 0.161 × 0.108 mm, monoclinic space group P2$_1$/c, a = 9.70880(10) Å, b = 15.88040(10) Å, c = 18.1762(2) Å, α = 90°, β = 102.9160(10)°, γ = 90°, V = 2731.50(5) Å^3, T = 100.00(10) K, Z = 4, $\rho_{calcd.}$ = 1.151 g cm^{-3}, μ = 1.145 mm^{-1}, F(000) = 1032, 56071 reflections in h(–12/12), k(–19/19), l(–22/21) measured in the range 3.738° < θ < 74.501°, 5591 independent reflections, 5591 observed reflections [I >2σ(I)], 294 parameters, 0 restraints; all data: R_1 = 0.0385 and wR_2 = 0.0970, I >2σ(I): R_1 = 0.0353 and wR_2 = 0.0944, *Goof* 1.095, largest difference peak/hole 0.272/–0.434 e Å^{-3}.

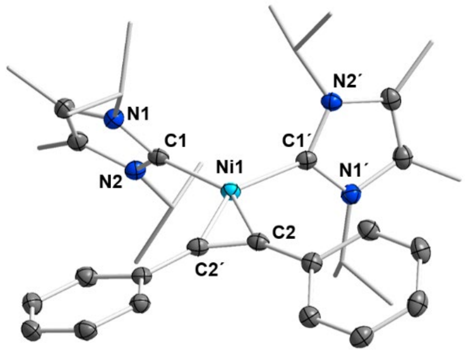

Figure S3. Molecular structure of [Ni(^{i}Pr$_2$Im^{Me})$_2$(η^2-PhC≡CPh)] **5** in the solid state (ellipsoids set at the 50% probability level). The hydrogen atoms were omitted for clarity. Selected bond lengths [Å] and angles [°] of **5**: Ni1–C1/C1´ 1.9251(13), Ni1–C2/C2´ 1.8804(14), C2–C2´ 1.302(3), C1–Ni1–C1´ 110.66(8), C1–Ni1–C2´ 104.57(6), C1´–Ni1–C2 104.57(6), C2–Ni1–C2´ 40.52(8), plane (C1–Ni1–C1´) – plane (C2–Ni1–C2´) 7.90(8).

Crystal data for 5: $C_{36}H_{50}N_4Ni$, M_r = 597.49, red block, 0.256 × 0.117 × 0.048 mm, monoclinic space group I2/a, a = 14.3550(2) Å, b = 10.7744(2) Å, c = 21.5899(3) Å, α = 90°, β = 95.0660(10)°, γ = 90°, V = 3326.19(9) Å^3, T = 99.98(10) K, Z = 4, $\rho_{calcd.}$ = 1.193 g cm^{-3}, μ = 1.049 mm^{-1}, F(000) = 1288, 16874 reflections in h(–17/17), k(–13/13), l(–26/19) measured in the range 4.111° < θ < 74.500°, 3385 independent reflections, 3385 observed reflections [I >2σ(I)], 192 parameters, 0 restraints; all data: R_1 = 0.0364 and wR_2 = 0.0933, I >2σ(I): R_1 = 0.0340 and wR_2 = 0.0917, *Goof* 1.072, largest difference peak/hole 0.409/–0.383 e Å^{-3}.

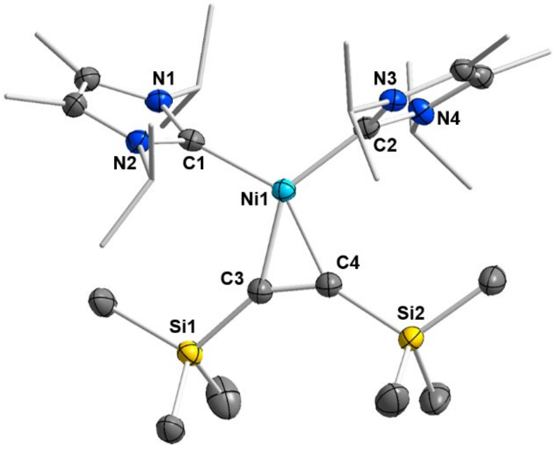

Figure S4. Molecular structure of [Ni(^{i}Pr$_2$Im^{Me})$_2$(η^2-Me$_3$SiC≡CSiMe$_3$] **7** in the solid state (ellipsoids set at the 50% probability level). The hydrogen atoms were omitted for clarity. Selected bond lengths [Å] and angles [°] of **7**: Ni1–C1 1.9183(15), Ni1–C2 1.9149(15), Ni1–C3 1.9047(16), Ni1–C4 1.9043(16), C3–C4 1.304(2), C3–Si1 1.8310(16), C4–Si2 1.8334(16), C1–Ni1–C2 114.54(6), C1–Ni1–C3 104.69(6), C2–Ni1–C4 101.13(6), C3–Ni1–C4 40.04(7), plane (C1–Ni1–C2) – plane (C3–Ni1–C4) 9.27(12).

Crystal data for 7: $C_{30}H_{58}N_4NiSi_2$, M$_r$ = 589.69, yellow block, 0.323 × 0.176 × 0.157 mm, monoclinic space group P2$_1$/n, a = 11.45070(10) Å, b = 21.4462(2) Å, c = 14.46620(10) Å, α = 90°, β = 99.7360(10)°, γ = 90°, *V* = 3501.36(5) Å^3, T = 99.95(18) K, Z = 4, $\rho_{calcd.}$ = 1.119 g cm^{-3}, μ = 1.614 mm^{-1}, *F*(000) = 1288, 33034 reflections in *h*(–14/14), *k*(–22/26), *l*(–17/18) measured in the range 3.723° < θ < 74.504°, 7151 independent reflections, 7151 observed reflections [$I > 2\sigma(I)$], 352 parameters, 0 restraints; all data: R_1 = 0.0412 and wR_2 = 0.1054, $I > 2\sigma(I)$: R_1 = 0.0381 and wR_2 = 0.1031, *Goof* 1.067, largest difference peak/hole 0.828/–0.314 e Å^{-3}.

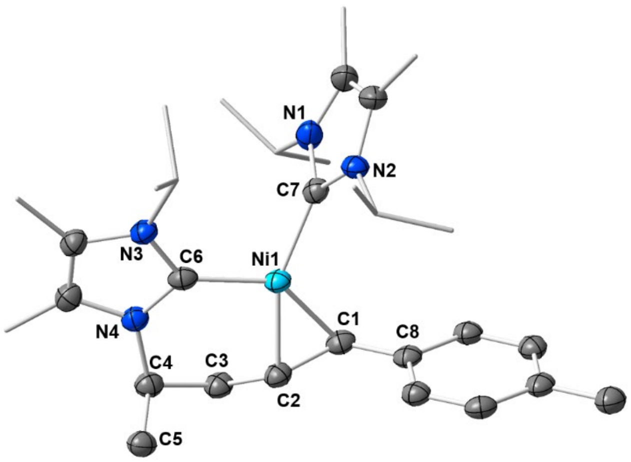

Figure S5. Molecular structure of **11a** in the solid state (ellipsoids set at 50% probability level). The hydrogen atoms have been omitted for clarity. Selected bond lengths [Å] and angles [°] of **11a**: Ni1–C7 1.9140(15), Ni1–C6 1.9072(15), Ni1–C1 1.9945(14), Ni1–C2 1.9321(14), C1–C2 1.439(2), C1–C8 1.474(2), C2–C3 1.516(2), C3–C4 1.532(2), C4–C5 1.533(2); C6–Ni1–C7 109.53(6), C1–Ni1–C7 110.67(6), C1–Ni1–C2 42.96(6), C2–Ni1–C6 95.74(6), C1–C2–C3 121.19(13), C2–C1–C8 123.28(13), plane (C1–Ni1–C2) – plane (N1–C7–N2) 88.58(9), plane (C1–Ni1–C2) – plane (N3–C6–N4) 32.51(11), plane (N3–C6–N4) – plane (N1–C7–N2) 77.05(11).

Crystal data for 11a: $C_{34}H_{55}N_4Ni$, M_r = 578.53, orange block, 0.278 × 0.183 × 0.140 mm, triclinic space group P-1, a = 10.2476(2) Å, b = 12.5503(2) Å, c = 13.3560(3) Å, α = 81.100(2)°, β = 88.985(2)°, γ = 79.456(2)°, V = 1668.29(6) Å^3, T = 99.97(16) K, Z = 2, $\rho_{calcd.}$ = 1.152 g cm^{-3}, μ = 1.024 mm^{-1}, F(000) = 630, 25615 reflections in h(–12/12), k(–15/15), l(–16/16) measured in the range 3.350° < θ < 74.495°, 6806 independent reflections, 6806 observed reflections [$I > 2\sigma(I)$], 367 parameters, 120 restraints; all data: R_1 = 0.0474 and wR_2 = 0.1699, $I > 2\sigma(I)$: R_1 = 0.0443 and wR_2 = 0.1599, *Goof* 0.774, largest difference peak/hole 0.665/–0.537 e Å^{-3}. The unit cell contains a half molecule of hexane which has been treated as a diffuse contribution to the overall scattering without specific atom positions by SQUEEZE.

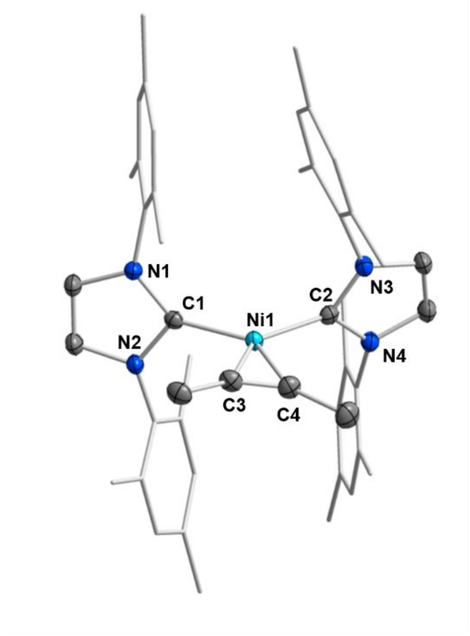

Figure S6. Molecular structure of [Ni(Mes_2Im)$_2$(η^2-MeC≡CMe)] **14** in the solid state (ellipsoids set at 50% probability level). The hydrogen atoms have been omitted for clarity. Selected bond lengths [Å] and angles [°] of **14**: Ni1–C1 1.9098(14), Ni1–C2 1.9127(14), Ni1–C3 1.9066(14), Ni1–C4 1.9055(15), C3–C4 1.280(2), C1–Ni1–C2 122.24(6), C1–Ni1–C3 99.62(6), C2–Ni1–C4 99.34(6), C3–Ni1–C4 39.24(7), plane (C1–Ni1–C2) – plane (C3–Ni1–C4) 9.60(7).

Crystal data for 14: $C_{46}H_{54}N_4Ni$, M_r = 721.62, yellow block, 0.160 × 0.120 × 0.080 mm, triclinic space group P-1, a = 12.8656(2) Å, b = 15.1103(3) Å, c = 20.4737(4) Å, α = 86.261(2)°, β = 89.7910(10)°, γ = 87.2690(10)°, V = 3967.17(13) Å^3, T = 100.00(10) K, Z = 4, $\rho_{calcd.}$ = 1.208 g cm^{-3}, μ = 0.970 mm^{-1}, F(000) = 1544, 70012 reflections in h(–16/15), k(–18/19), l(–25/25) measured in the range 2.163° < θ < 77.784°, 16605 independent reflections, 16605 observed reflections [I >2σ(I)], 947 parameters, 0 restraints; all data: R_1 = 0.0432 and wR_2 = 0.1061, I >2σ(I): R_1 = 0.0382 and wR_2 = 0.1012, *Goof* 1.086, largest difference peak/hole 0.981/–0.415 e Å^{-3}.

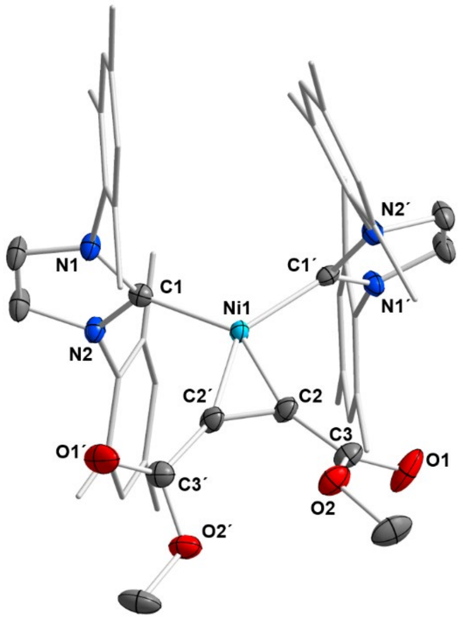

Figure S7. Molecular structure of [Ni(Mes$_2$Im)$_2$(η^2-MeOOCC≡CCOOMe)] **15** in the solid state (ellipsoids set at 50% probability level). The hydrogen atoms have been omitted for clarity. Selected bond lengths [Å] and angles [°] of **15**: Ni1–C1/C1´ 1.917(2), Ni1–C2/C2´ 1.873(2), C2–C2´ 1.300(4), C1–Ni1–C1´ 118.47(12), C1–Ni1–C2´ 100.49(9), C1´–Ni1–C2 100.49(9), C2–Ni1–C2´ 40.61(13), plane (C1–Ni1–C1´) – plane (C2–Ni1–C2´) 3.26(13).

Crystal data for 15: $C_{48}H_{54}N_4NiO_4$, M_r = 809.66, red block, 0.315 × 0.301 × 0.289 mm, monoclinic space group C2/c, a = 17.4692(18) Å, b = 11.9174(12) Å, c = 21.442(2) Å, α = 90°, β = 106.626(3)°, γ = 90°, V = 4277.2(7) Å^3, T = 100(2) K, Z = 4, $\rho_{calcd.}$ = 1.257 g cm^{-3}, μ = 0.502 mm^{-1}, F(000) = 1720, 16456 reflections in h(–22/14), k(–14/15), l(–21/27) measured in the range 1.982° < θ < 26.736°, 4528 independent reflections, 4528 observed reflections [I >2σ(I)], 265 parameters, 0 restraints; all data: R_1 = 0.0703 and wR_2 = 0.1107, I >2σ(I): R_1 = 0.0448 and wR_2 = 0.1006, $Goof$ 1.023, largest difference peak/hole 0.697/–0.591 e Å^{-3}.

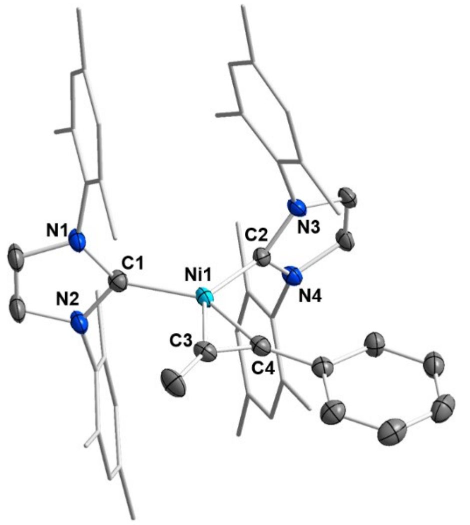

Figure S8. Molecular structure of [Ni(Mes_2Im)$_2$(η^2-PhC≡CMe)] **16** in the solid state (ellipsoids set at 50% probability level). The hydrogen atoms have been omitted for clarity. Selected bond lengths [Å] and angles [°] of **16**: : Ni1–C1 1.927(5), Ni1–C2 1.913(5), Ni1–C3 1.902(5), Ni1–C4 1.912(5), C3–C4 1.291(7), C1–Ni1–C2 118.5(2), C1–Ni1–C3 102.6(2), C2–Ni1–C4 99.5(2), C3–Ni1–C4 39.6(2), plane (C1–Ni1–C2) – plane (C3–Ni1–C4) 5.73(22).

Crystal data for 16: $C_{51}H_{56}N_4Ni$, M_r = 783.69, orange block, 0.339 × 0.084 × 0.054 mm, triclinic space group P-1, a = 12.6857(3) Å, b = 16.5374(4) Å, c = 20.4977(4) Å, α = 84.038(2)°, β = 88.408(2)°, γ = 87.209(2)°, V = 4270.73(17) Å^3, T = 99.9(7) K, Z = 4, $\rho_{calcd.}$ = 1.219 g cm^{-3}, μ = 0.943 mm^{-1}, F(000) = 1672, 17197 reflections in h(–15/15), k(–20/20), l(–25/25) measured in the range 2.689° < θ < 74.503°, 17197 independent reflections, 17197 observed reflections [I >2σ(I)], 1036 parameters, 0 restraints; all data: R_1 = 0.0856 and wR_2 = 0.2583, I >2σ(I): R_1 = 0.0802 and wR_2 = 0.2558, $Goof$ 1.112, largest difference peak/hole 1.902/–0.583 e Å^{-3}.

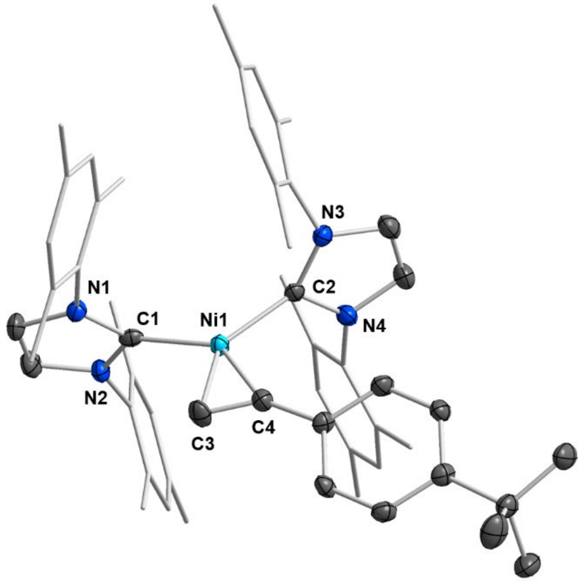

Figure S9. Molecular structure of Ni(Mes$_2$Im)$_2$(η^2-HC≡C(4-^{t}Bu-C$_6$H$_4$)] **17** in the solid state (ellipsoids set at 50% probability level). The hydrogen atoms and a hexane molecule have been omitted for clarity. Selected bond lengths [Å] and angles [°] of **17**: : Ni1–C1 1.921(3), Ni1–C2 1.912(3), Ni1–C3 1.876(4), Ni1–C4 1.916(3), C3–C4 1.277(5), C1–Ni1–C2 124.59(14), C1–Ni1–C3 96.26(15), C2–Ni1–C4 99.82(14), C3–Ni1–C4 39.33(15), plane (C1–Ni1–C2) – plane (C3–Ni1–C4) 1.50(17).

Crystal data for 17: $C_{54}H_{62}N_4Ni + C_6H_{14}$, M_r = 911.95, orange block, 0.230 × 0.192 × 0.120 mm, monoclinic space group P2$_1$/n, a = 17.0883(17) Å, b = 15.2716(15) Å, c = 20.1859(18) Å, α = 90°, β = 98.233(3)°, γ = 90°, V = 5213.5(9) Å^3, T = 100(2) K, Z = 4, $\rho_{calcd.}$ = 1.162 g cm^{-3}, μ = 0.413 mm^{-1}, F(000) = 1968, 96132 reflections in h(–21/21), k(–19/18), l(–25/25) measured in the range 1.462° < θ < 26.764°, 11068 independent reflections, 11068 observed reflections [$I > 2\sigma(I)$], 603 parameters, 30 restraints; all data: R_1 = 0.1316 and wR_2 = 0.1485, $I > 2\sigma(I)$: R_1 = 0.0687 and wR_2 = 0.1255, *Goof* 1.037, largest difference peak/hole 0.641/–0.593 e Å^{-3}.

3) NMR Spectra

[Ni_2(^{i}Pr_2Im^{Me})$_4$(μ-(η^2:η^2)-COD)] B and [Ni(^{i}Pr_2Im^{Me})$_2$(COD)] B’

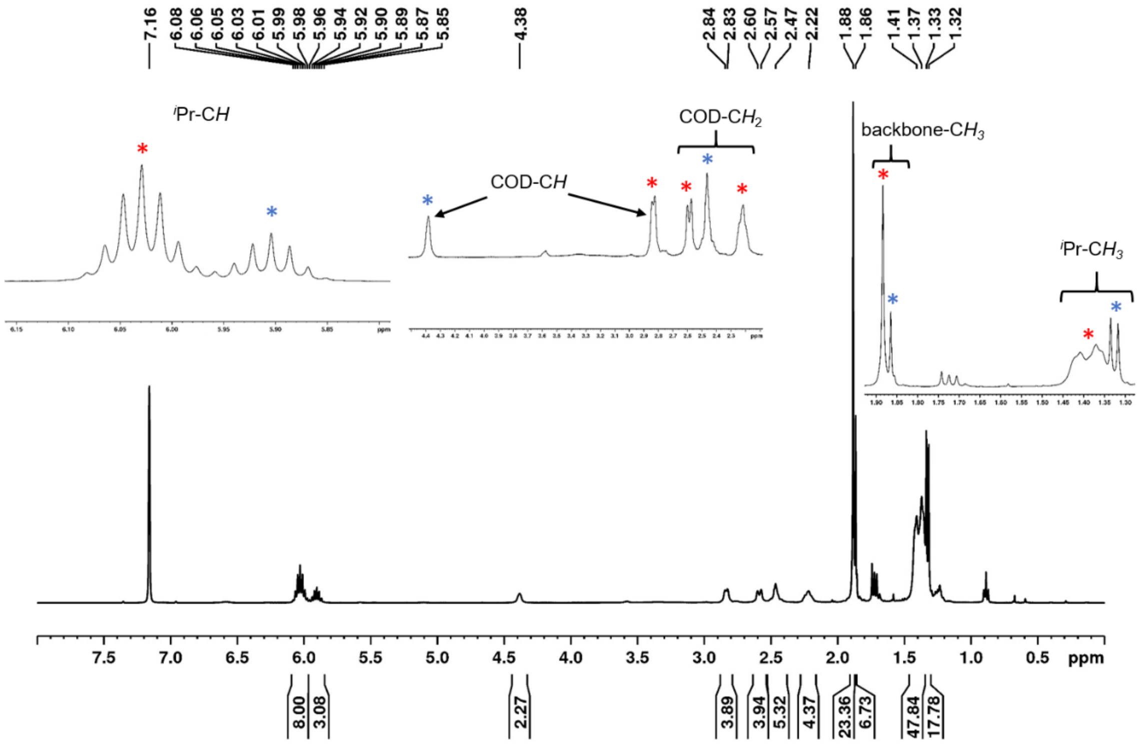

Figure S10. ^{1}H NMR spectrum of [Ni_2(^{i}Pr_2Im^{Me})$_4$(μ-(η^2:η^2)-COD)] **B** (*)and [Ni(^{i}Pr_2Im^{Me})$_2$(COD)] **B’** (*) (400MHz, 25 °C, C_6D_6).

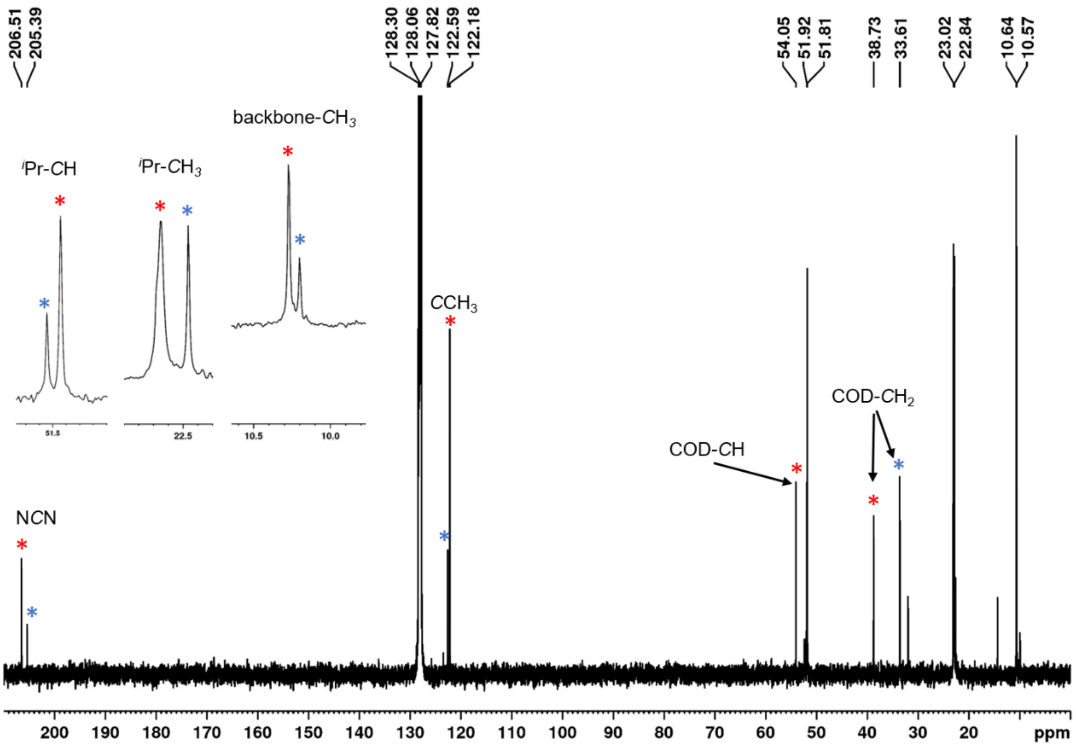

Figure S11. $^{13}C\{^1H\}$ NMR spectrum of [Ni_2(^{i}Pr_2Im^{Me})$_4$(μ-(η^2:η^2)-COD)] **B** (*)and [Ni(^{i}Pr_2Im^{Me})$_2$(COD)] **B’** (*) (100MHz, 25 °C, C_6D_6).

[Ni(^{i}Pr$_2$Im^{Me})$_2$(η^2-MeC≡CMe)] 3

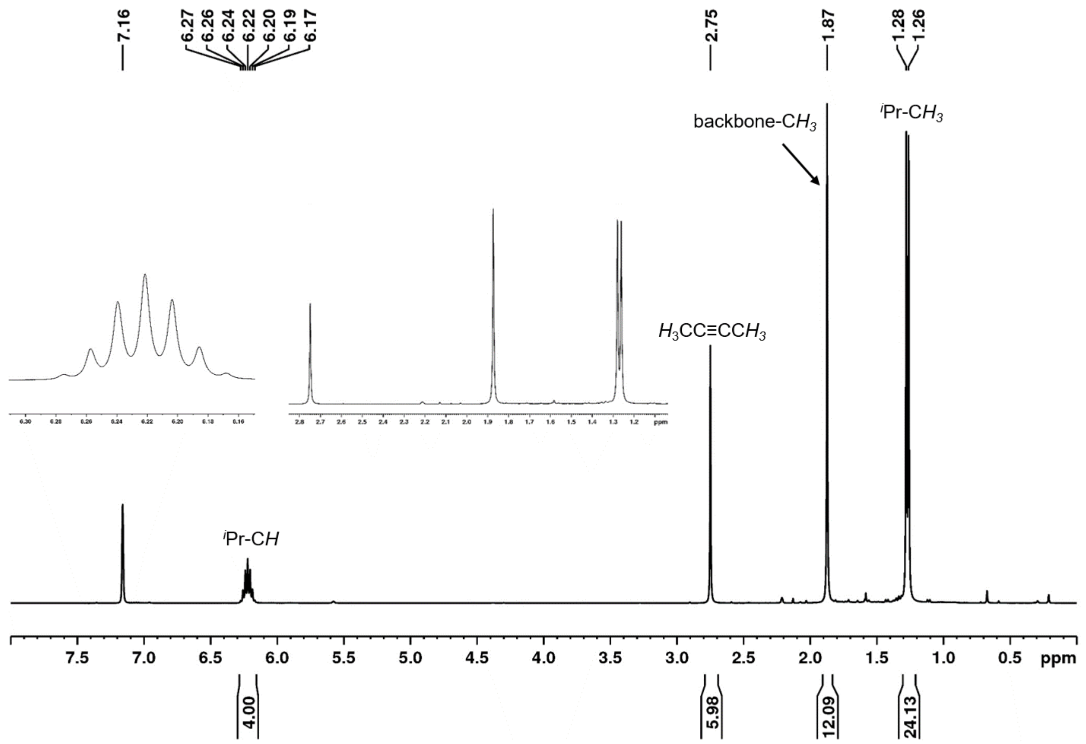

Figure S12. ^{1}H NMR spectrum of [Ni(^{i}Pr$_2$Im^{Me})$_2$(η^2-MeC≡CMe)] **3** (400MHz, 25 °C, C_6D_6).

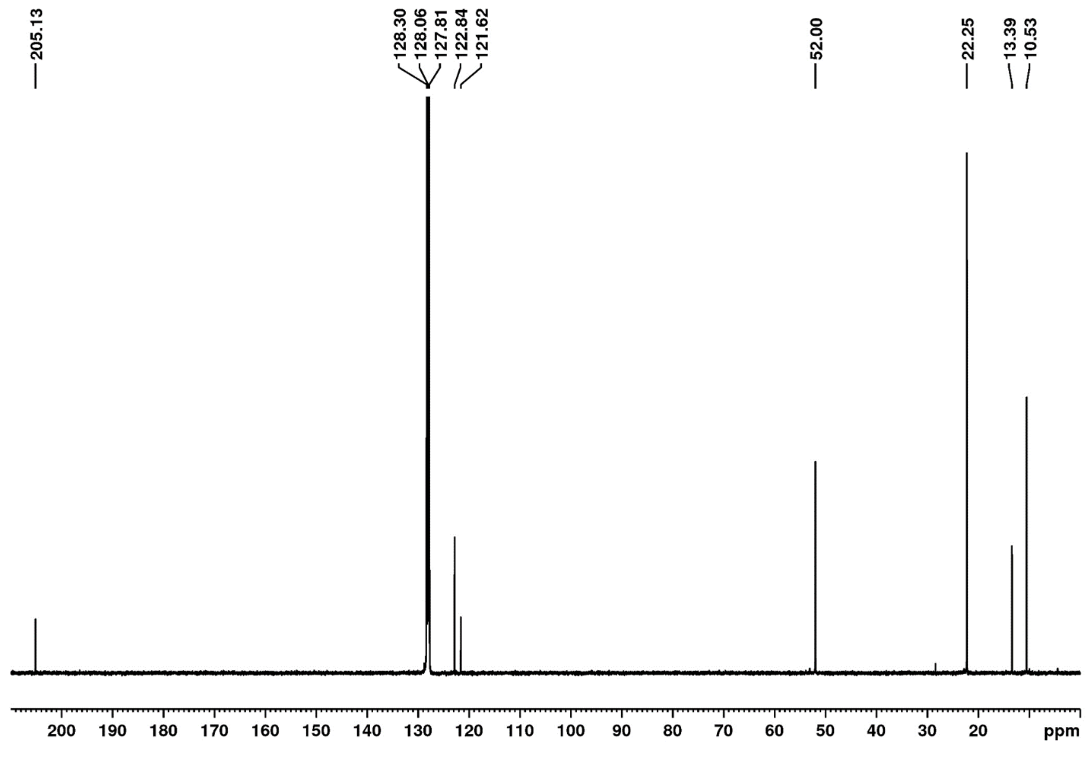

Figure S13. ^{13}C{^{1}H} NMR spectrum of [Ni(^{i}Pr$_2$Im^{Me})$_2$(η^2-MeC≡CMe)] **3** (100MHz, 25 °C, C_6D_6).

[Ni(^{i}Pr$_2$Im^{Me})$_2$(η^2-H$_7$C$_3$C≡CC$_3$H$_7$)] 4

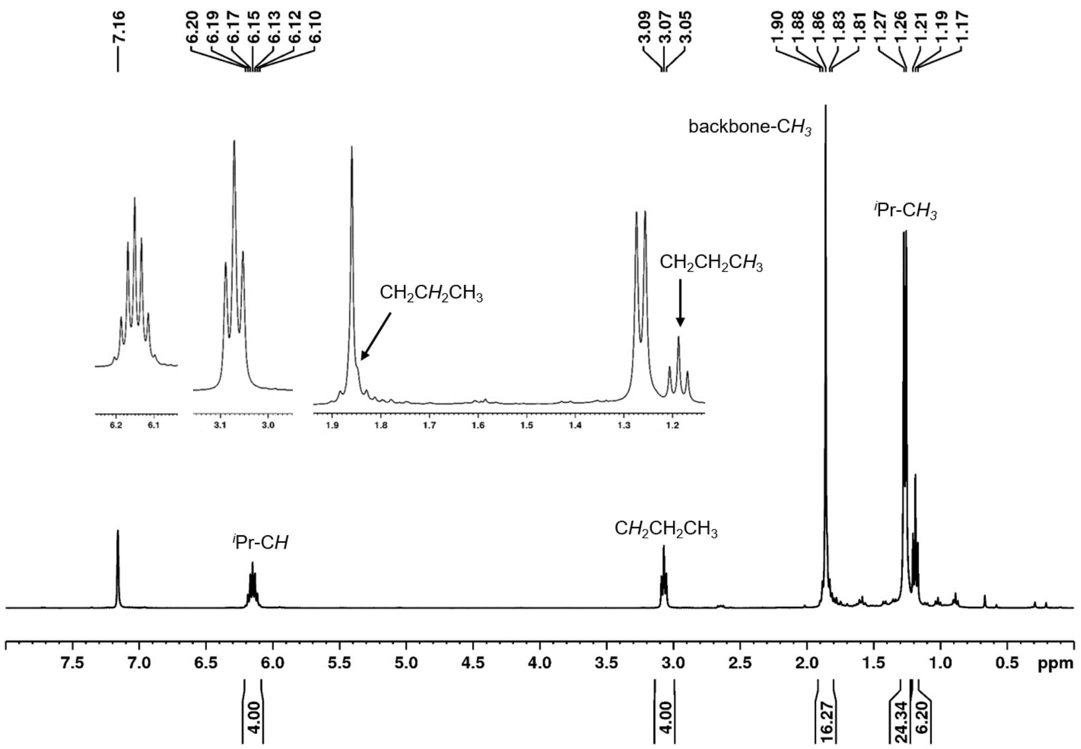

Figure S14. ^{1}H NMR spectrum of [Ni(^{i}Pr$_2$Im^{Me})$_2$(η^2-H$_7$C$_3$C≡CC$_3$H$_7$)] **4** (400MHz, 25 °C, C_6D_6).

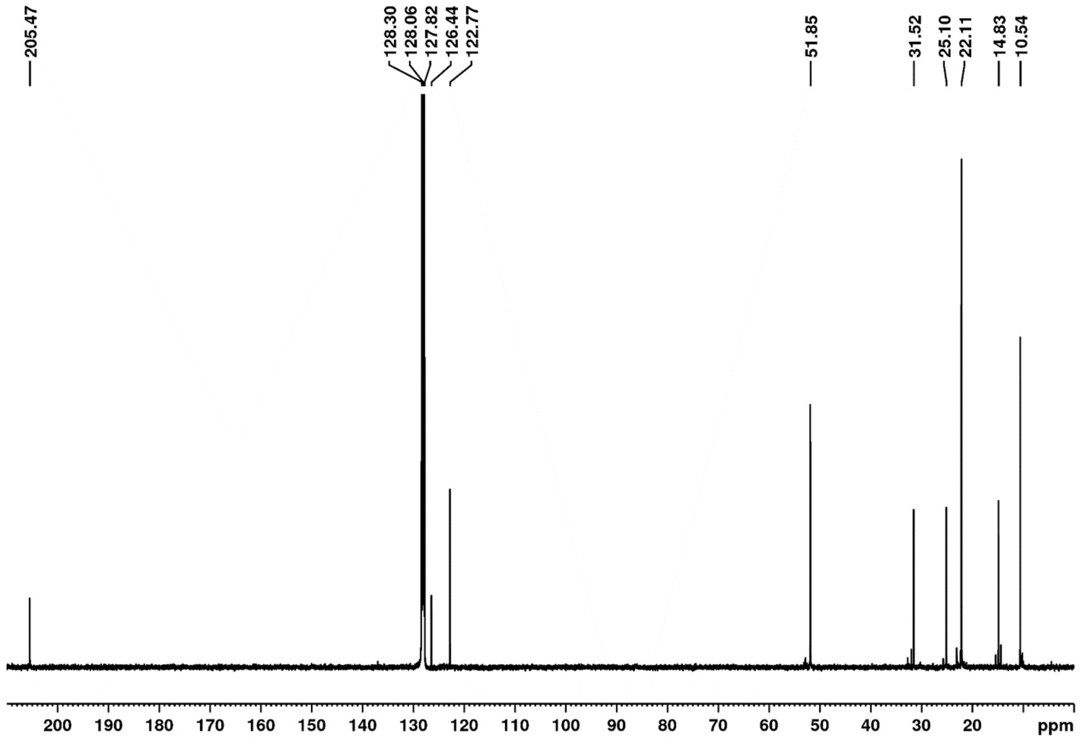

Figure S15. ^{13}C{^{1}H} NMR spectrum of [Ni(^{i}Pr$_2$Im^{Me})$_2$(η^2-H$_7$C$_3$C≡CC$_3$H$_7$)] **4** (100MHz, 25 °C, C_6D_6).

[Ni(^{i}Pr$_2$Im^{Me})$_2$(η^2-PhC≡CPh)] 5

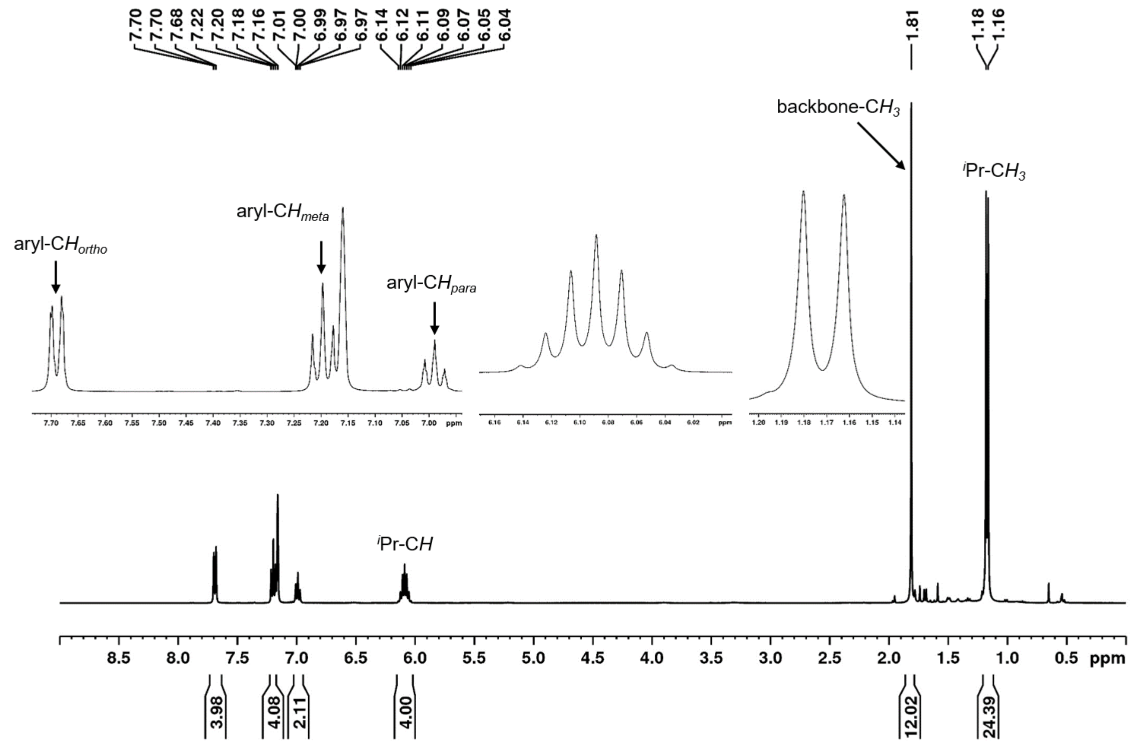

Figure S16. ^{1}H NMR spectrum of [Ni(^{i}Pr$_2$Im^{Me})$_2$(η^2-PhC≡CPh)] **5** (400MHz, 25 °C, C_6D_6).

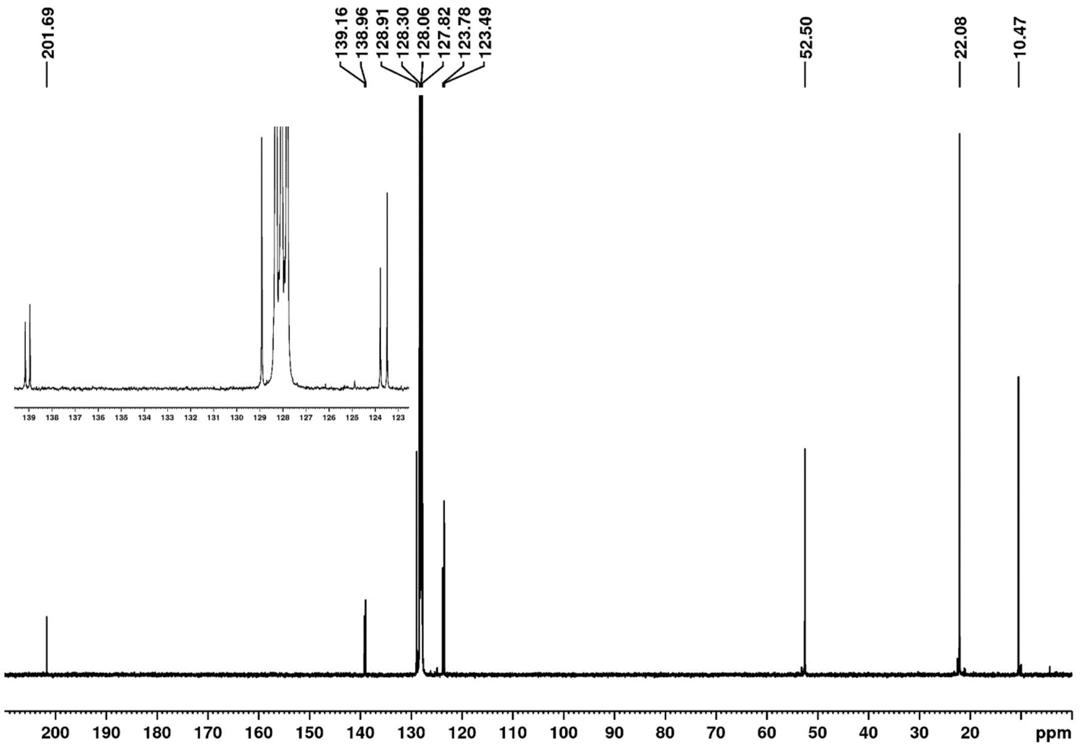

Figure S17. ^{13}C{^{1}H} NMR spectrum of [Ni(^{i}Pr$_2$Im^{Me})$_2$(η^2-PhC≡CPh)] **5** (100MHz, 25 °C, C_6D_6).

[Ni(^{i}Pr$_2$Im^{Me})$_2$(η^2-MeOOCC≡CCOOMe)] 6

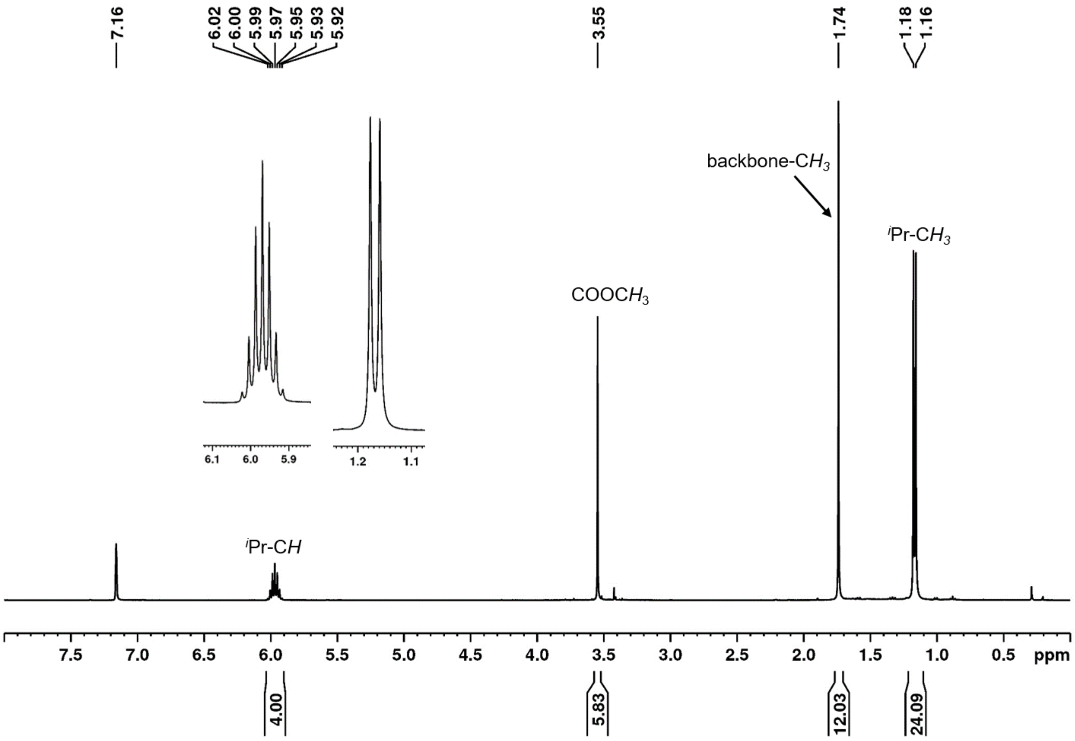

Figure S18. ^{1}H NMR spectrum of [Ni(^{i}Pr$_2$Im^{Me})$_2$(η^2-MeOOCC≡CCOOMe)] **6** (400MHz, 25 °C, C_6D_6).

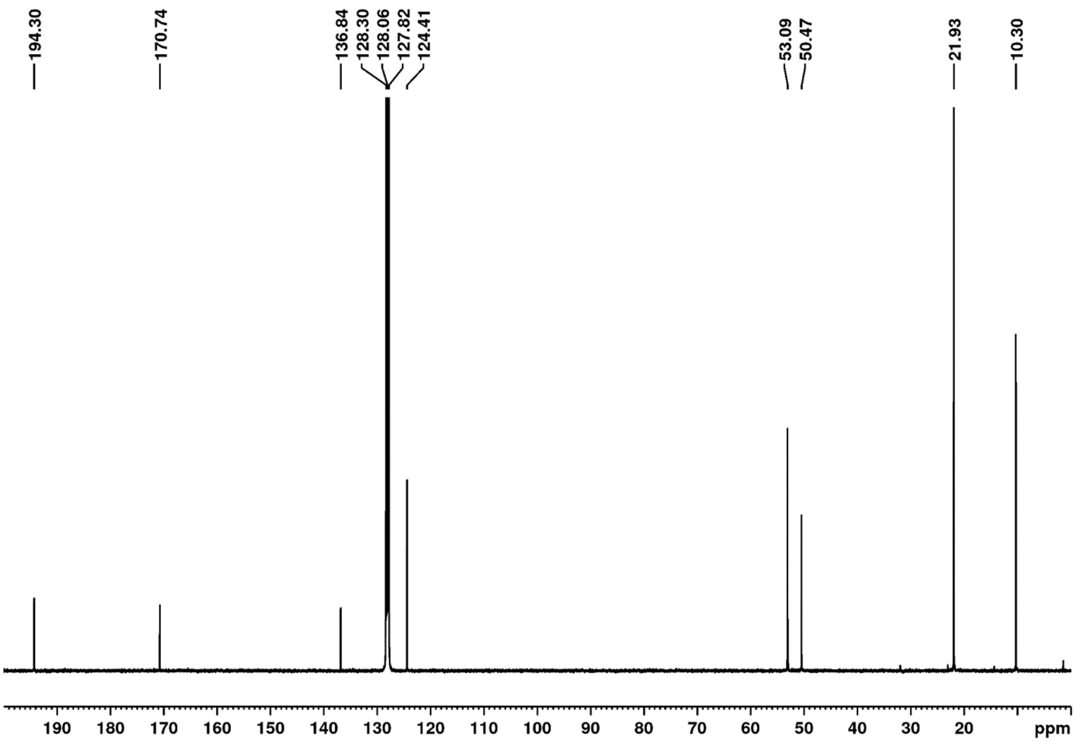

Figure S19. ^{13}C{^{1}H} NMR spectrum of [Ni(^{i}Pr$_2$Im^{Me})$_2$(η^2-MeOOCC≡CCOOMe)] **6** (100MHz, 25 °C, C_6D_6).

[Ni(^{i}Pr$_2$Im^{Me})$_2$(η^2-Me$_3$SiC≡CSiMe$_3$)] 7

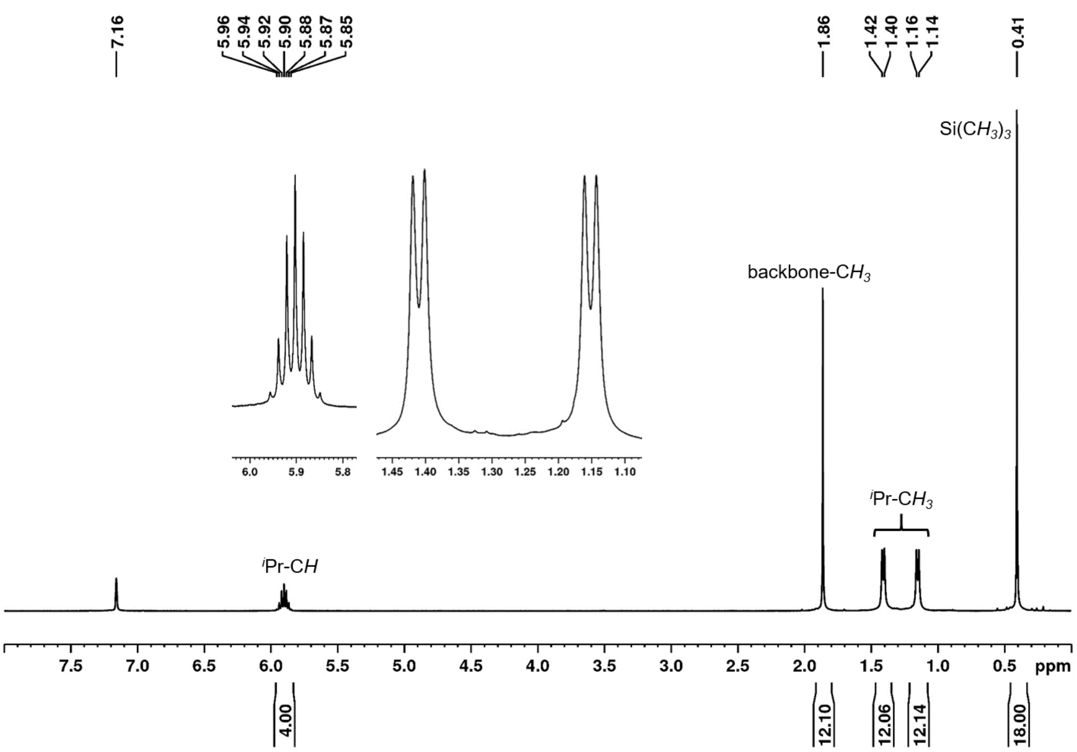

Figure S20. ^{1}H NMR spectrum of [Ni(^{i}Pr$_2$Im^{Me})$_2$(η^2-Me$_3$SiC≡CSiMe$_3$)] **7** (400MHz, 25 °C, C_6D_6).

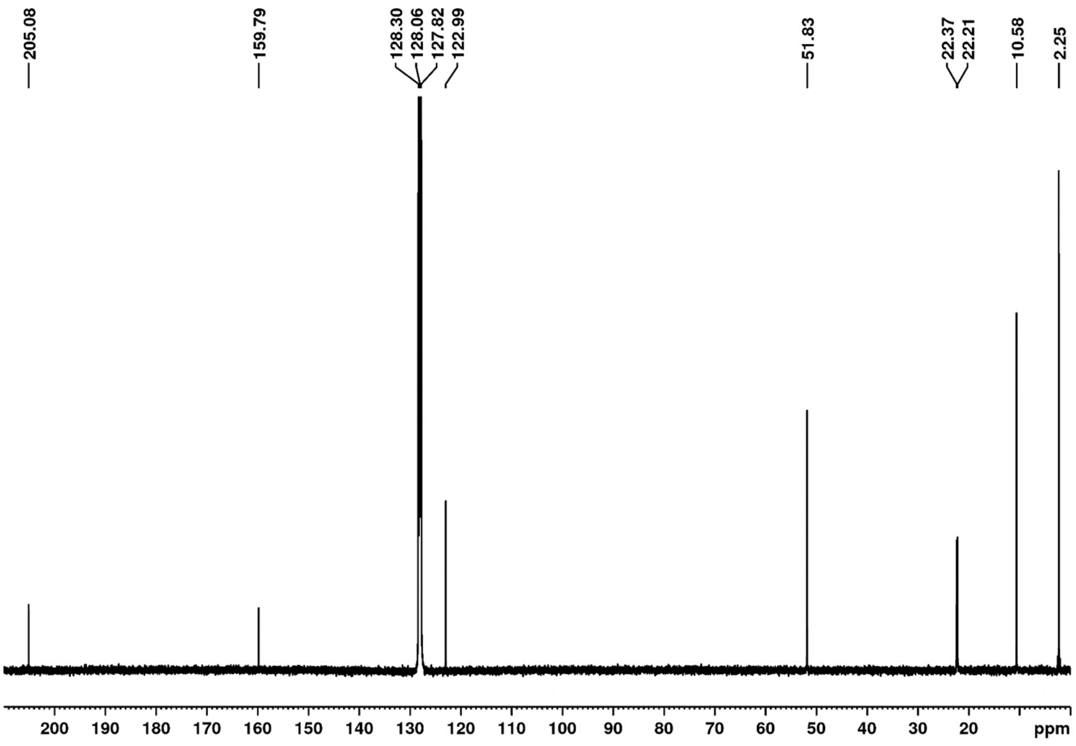

Figure S21. ^{13}C{^{1}H} NMR spectrum of [Ni(^{i}Pr$_2$Im^{Me})$_2$(η^2-Me$_3$SiC≡CSiMe$_3$)] **7** (100MHz, 25 °C, C_6D_6).

[Ni(^{i}Pr$_2$Im^{Me})$_2$(η^2-PhC≡CMe)] 8

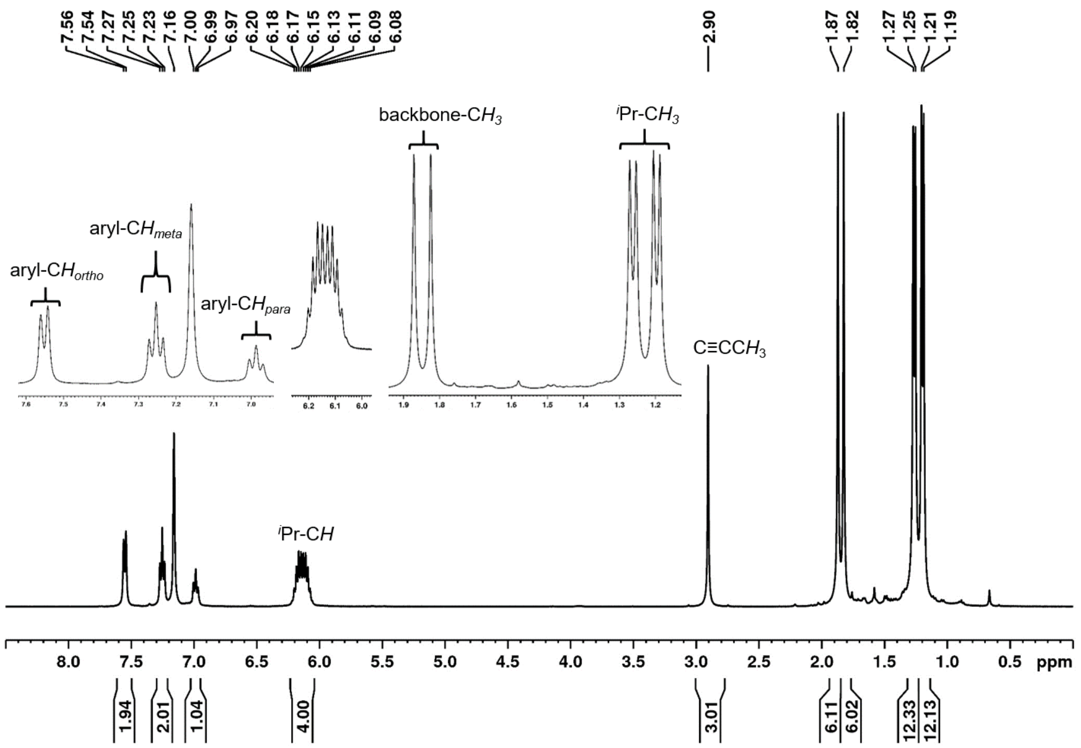

Figure S22. ^{1}H NMR spectrum of [Ni(^{i}Pr$_2$Im^{Me})$_2$(η^2-PhC≡CMe)] **8** (400MHz, 25 °C, C_6D_6).

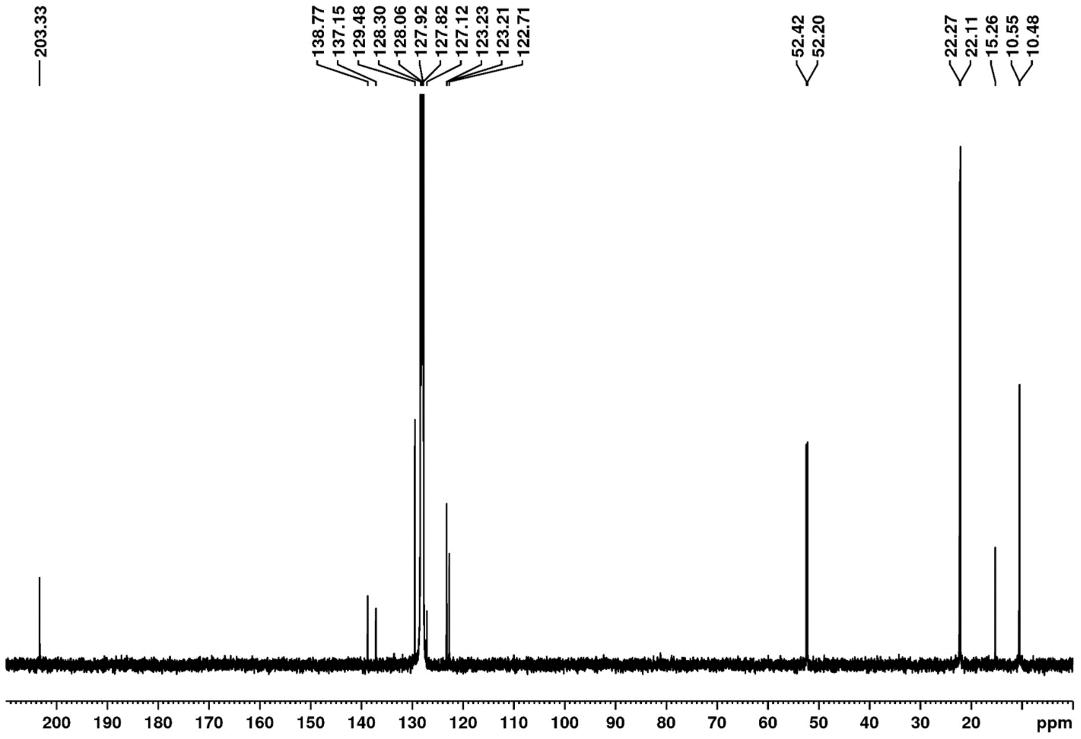

Figure S23. ^{13}C{^{1}H} NMR spectrum of [Ni(^{i}Pr$_2$Im^{Me})$_2$(η^2-PhC≡CMe)] **8** (100MHz, 25 °C, C_6D_6).

[Ni(^{i}Pr$_2$Im^{Me})$_2$(η^2-HC≡CC$_3$H$_7$)] 9

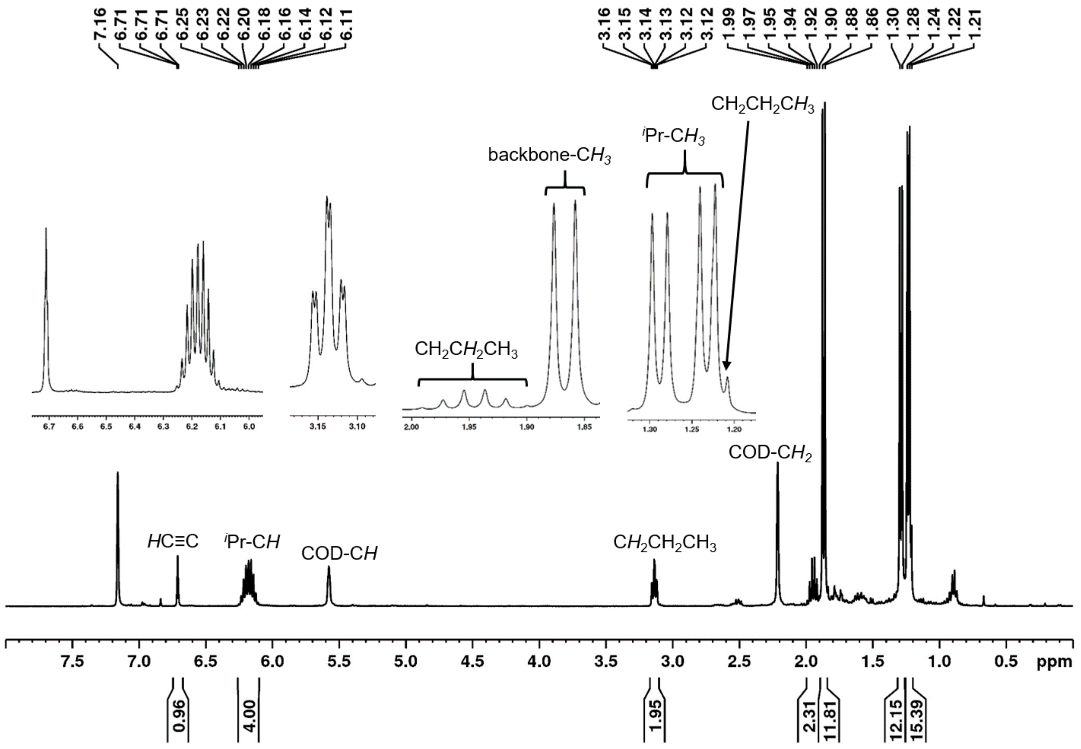

Figure S24. ^{1}H NMR spectrum of [Ni(^{i}Pr$_2$Im^{Me})$_2$(η^2-HC≡CC$_3$H$_7$)] **9** (400MHz, 25 °C, C_6D_6).

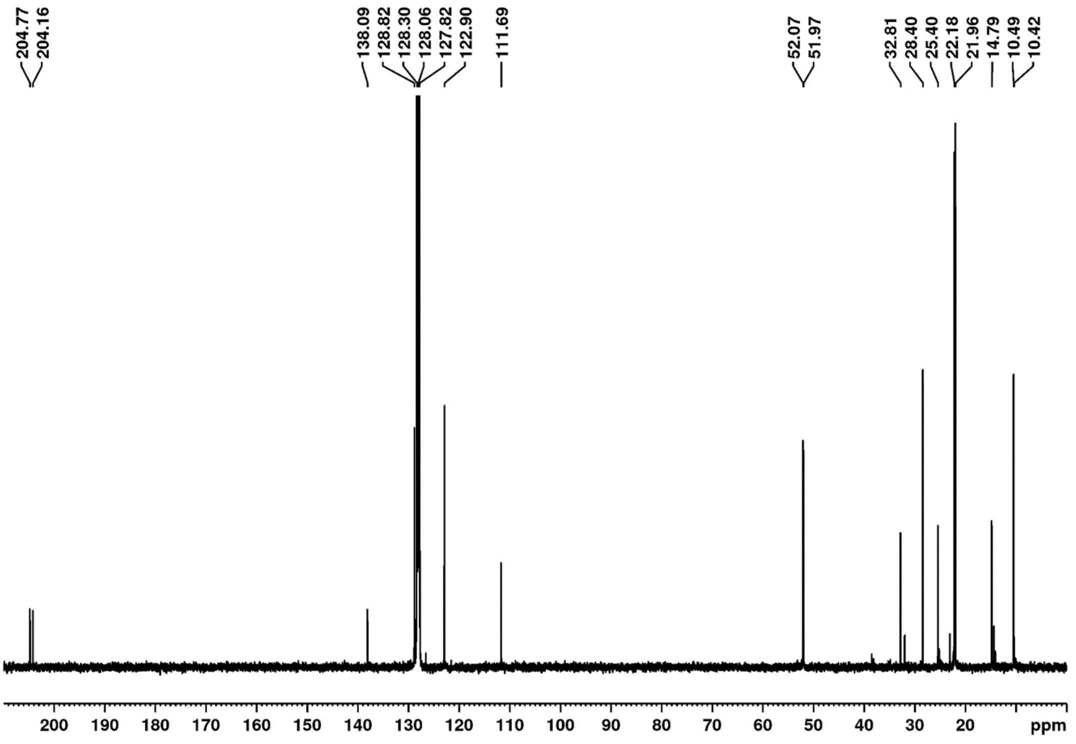

Figure S25. ^{13}C{^{1}H} NMR spectrum of [Ni(^{i}Pr$_2$Im^{Me})$_2$(η^2-HC≡CC$_3$H$_7$)] **9** (100MHz, 25 °C, C_6D_6).

[Ni(^{i}Pr$_2$Im^{Me})$_2$(η^2-HC≡CPh)] 10

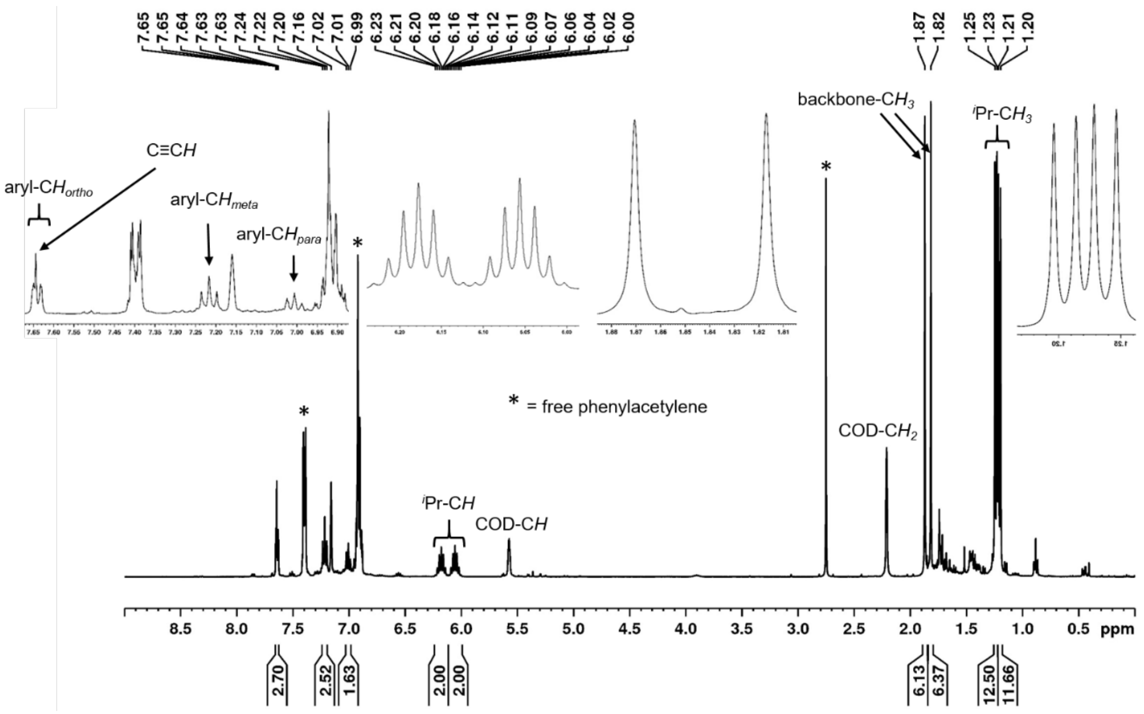

Figure S26. ^{1}H NMR spectrum of [Ni(^{i}Pr$_2$Im^{Me})$_2$(η^2-HC≡CPh)] **10** (400MHz, 25 °C, C_6D_6).

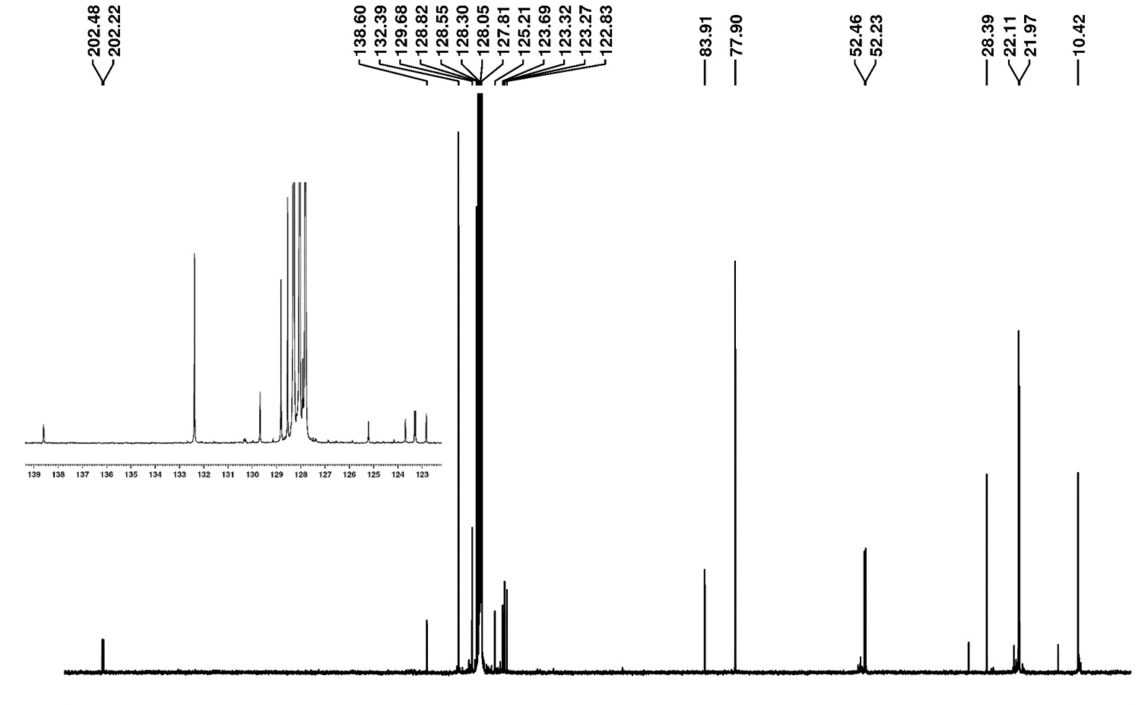

Figure S27. ^{13}C{^{1}H} NMR spectrum of [Ni(^{i}Pr$_2$Im^{Me})$_2$(η^2-HC≡CPh)] **10** (100MHz, 25 °C, C_6D_6).

[Ni(^{i}Pr$_2$Im^{Me})$_2$(η^2-HC≡C(*p*-Tol))] 11

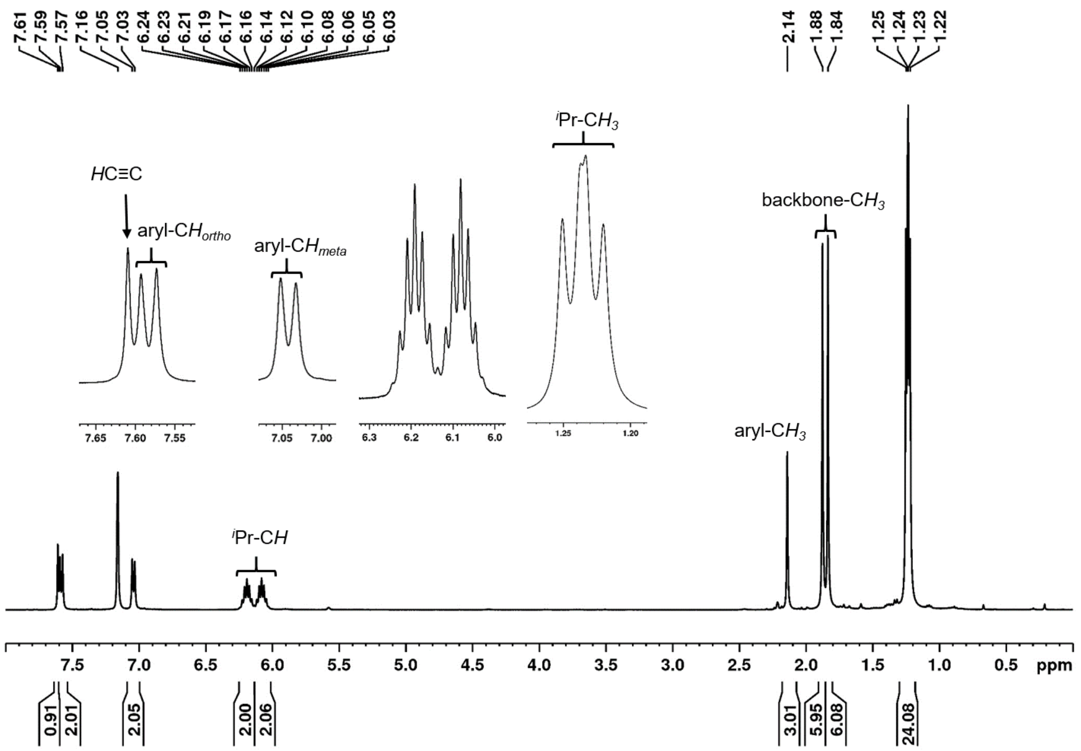

Figure S28. ^{1}H NMR spectrum of [Ni(^{i}Pr$_2$Im^{Me})$_2$(η^2-HC≡C(*p*-Tol))] **11** (400MHz, 25 °C, C_6D_6).

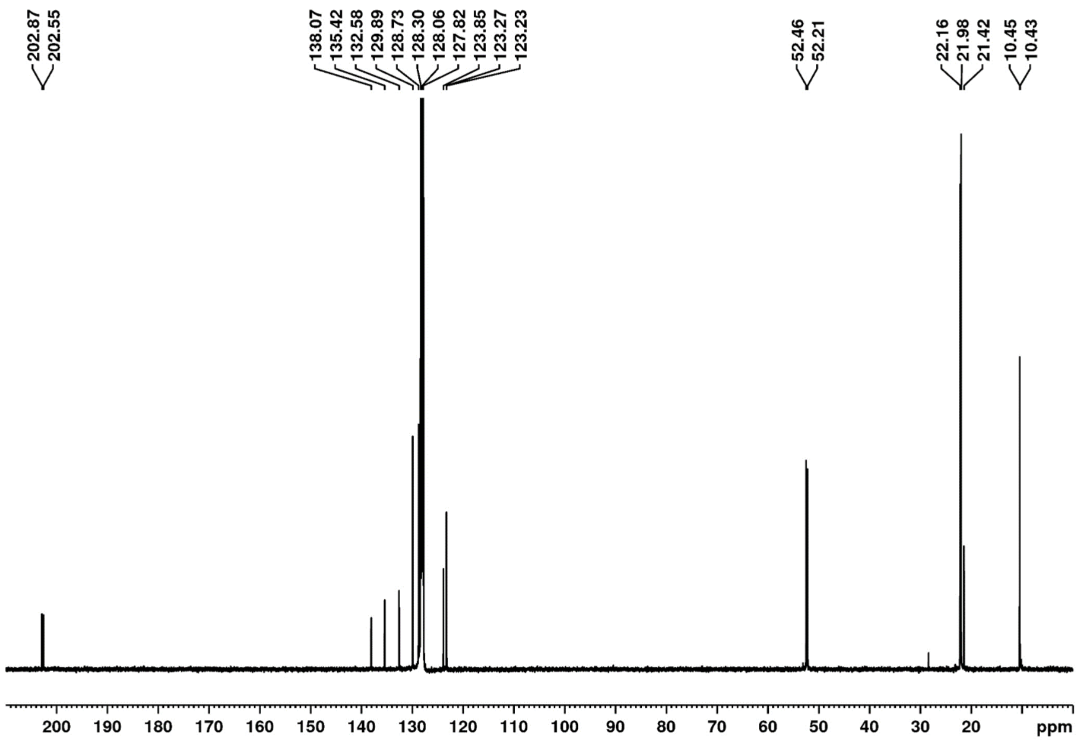

Figure S29. ^{13}C{^{1}H} NMR spectrum of [Ni(^{i}Pr$_2$Im^{Me})$_2$(η^2-HC≡C(*p*-Tol))] **11** (100MHz, 25 °C, C_6D_6).

[Ni(^{i}Pr$_2$Im^{Me})$_2$(η^2-HC≡C(4-^{t}Bu-C$_6$H$_4$))] 12

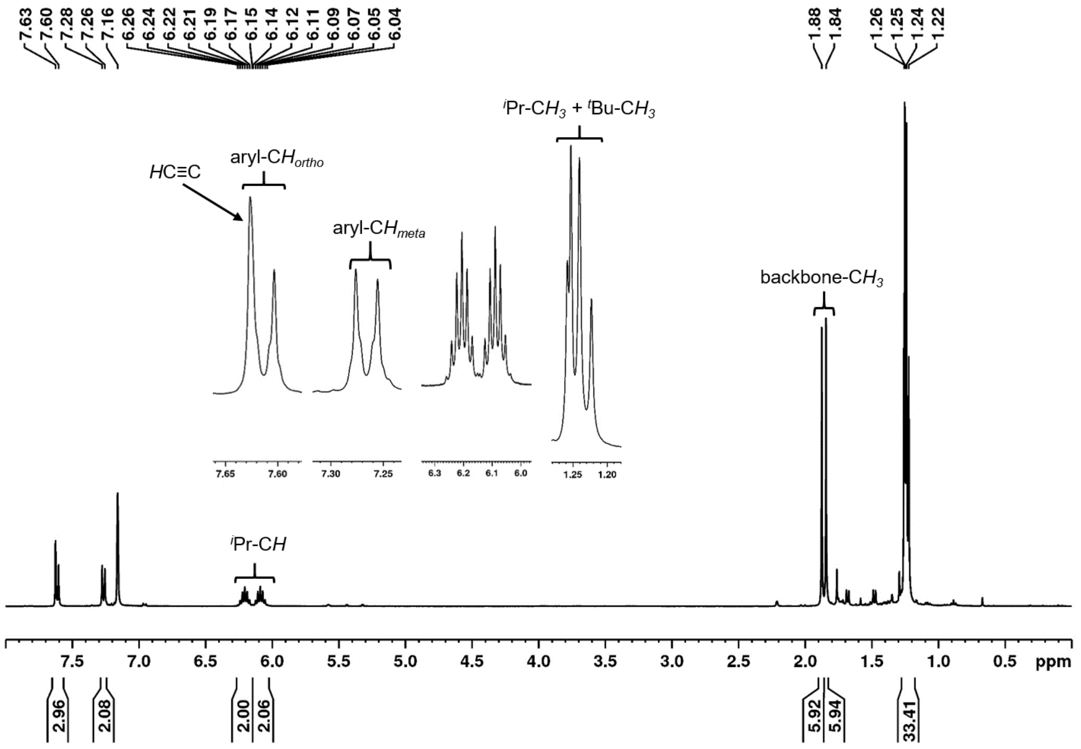

Figure S30. ^{1}H NMR spectrum of [Ni(^{i}Pr$_2$Im^{Me})$_2$(η^2-HC≡C(4-^{t}Bu-C$_6$H$_4$))] **12** (400MHz, 25 °C, C$_6$D$_6$).

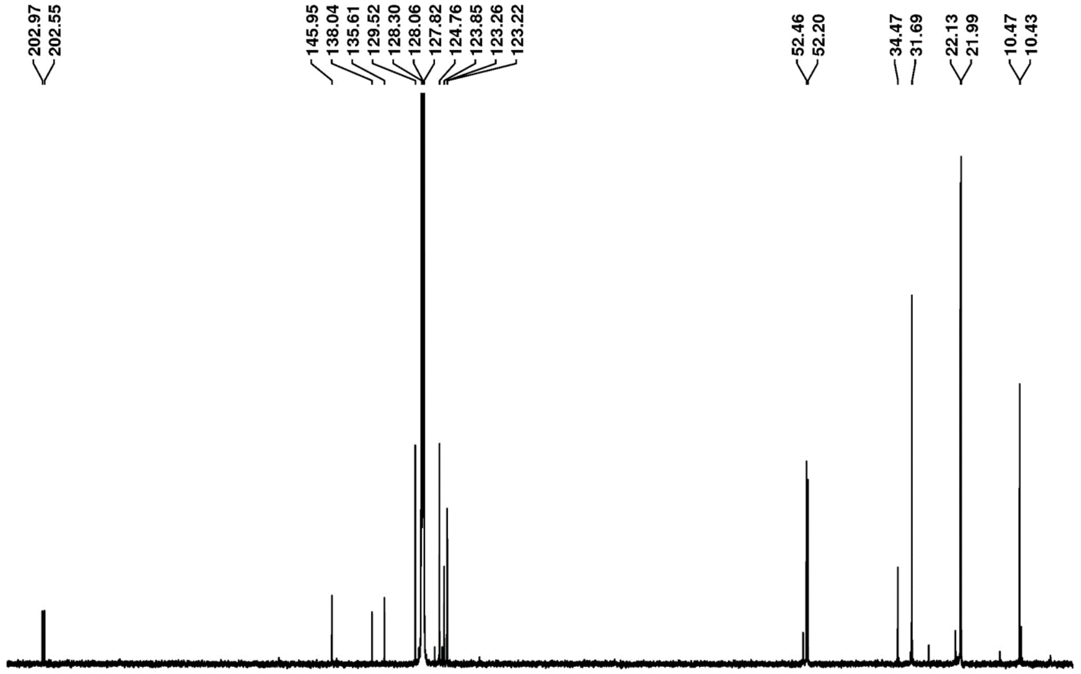

Figure S31. ^{13}C{^{1}H} NMR spectrum of [Ni(^{i}Pr$_2$Im^{Me})$_2$(η^2-HC≡C(4-^{t}Bu-C$_6$H$_4$))] **12** (100MHz, 25 °C, C$_6$D$_6$).

[Ni(^{i}Pr$_2$Im^{Me})$_2$(η^2-HC≡CCOOMe)] 13

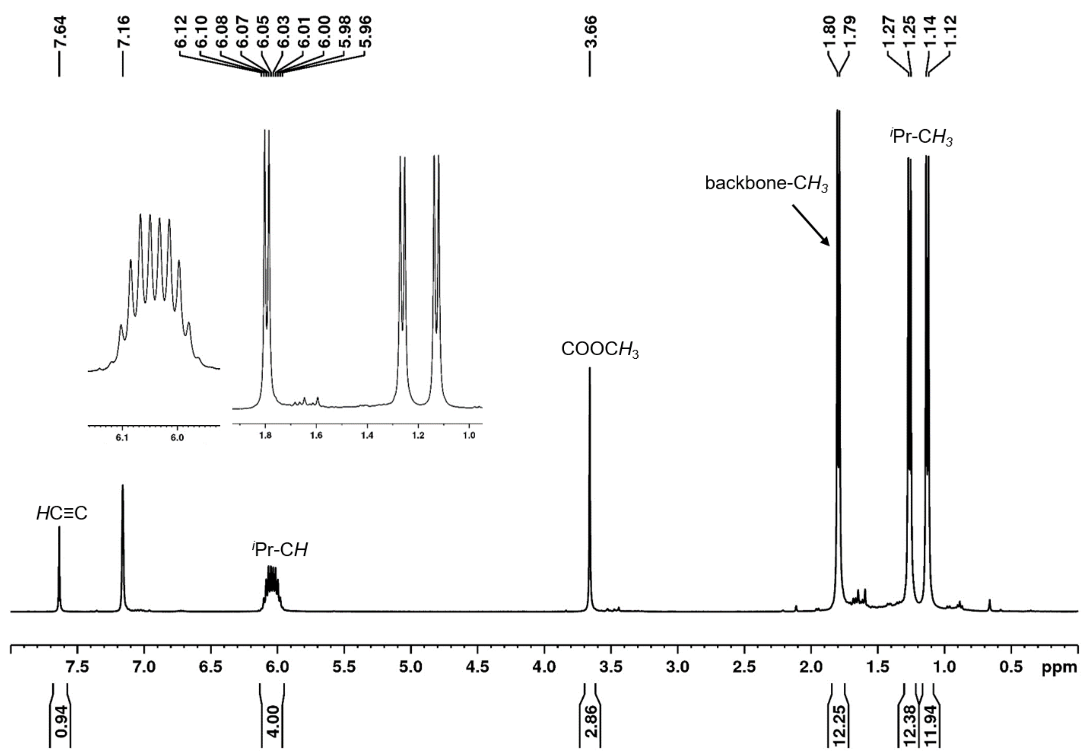

Figure S32. ^{1}H NMR spectrum of [Ni(^{i}Pr$_2$Im^{Me})$_2$(η^2-HC≡CCOOMe)] **13** (400MHz, 25 °C, C_6D_6).

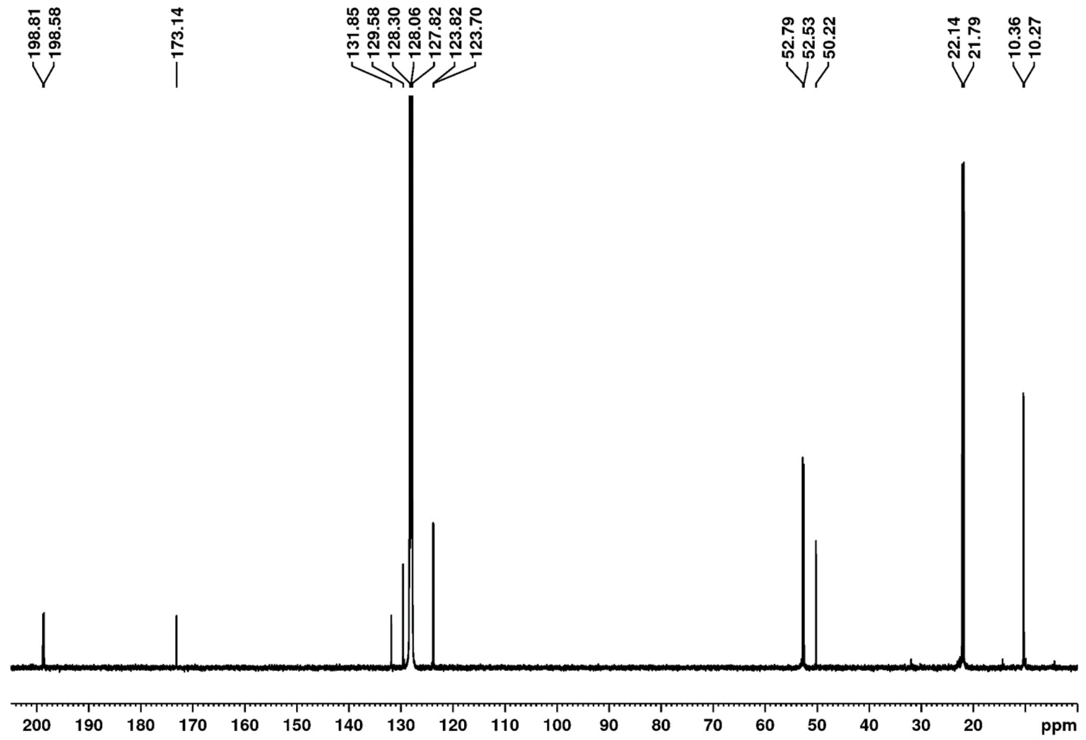

Figure S33. ^{13}C{^{1}H} NMR spectrum of [Ni(^{i}Pr$_2$Im^{Me})$_2$(η^2-HC≡CCOOMe)] **13** (100MHz, 25 °C, C_6D_6).

Rearrangement product 11a

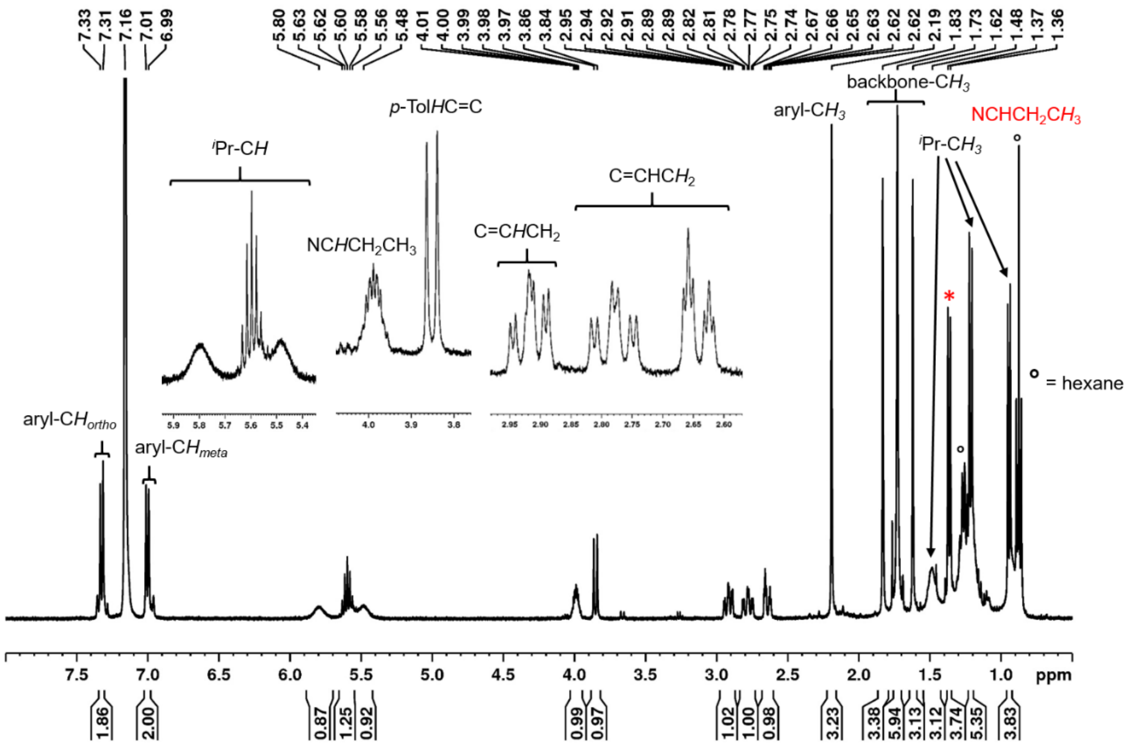

Figure S34. ^{1}H NMR spectrum of the rearrangement product **11a** (400MHz, 25 °C, C_6D_6).

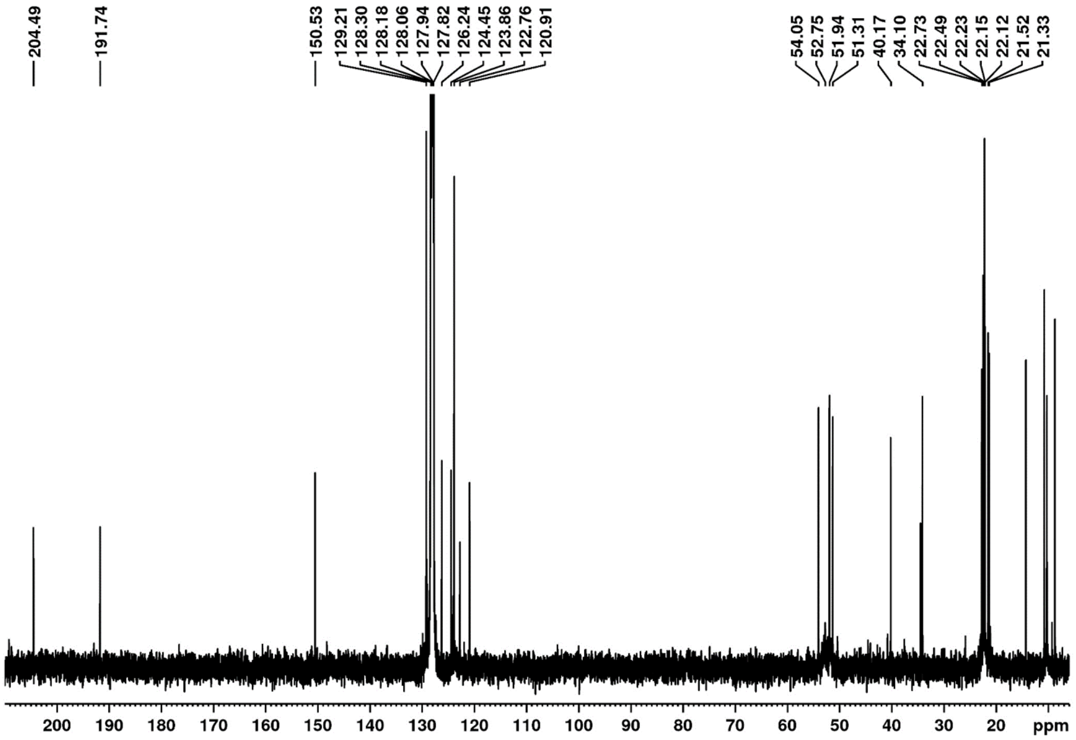

Figure S35. ^{13}C{^{1}H} NMR spectrum of the rearrangement product **11a** (100MHz, 25 °C, C_6D_6).

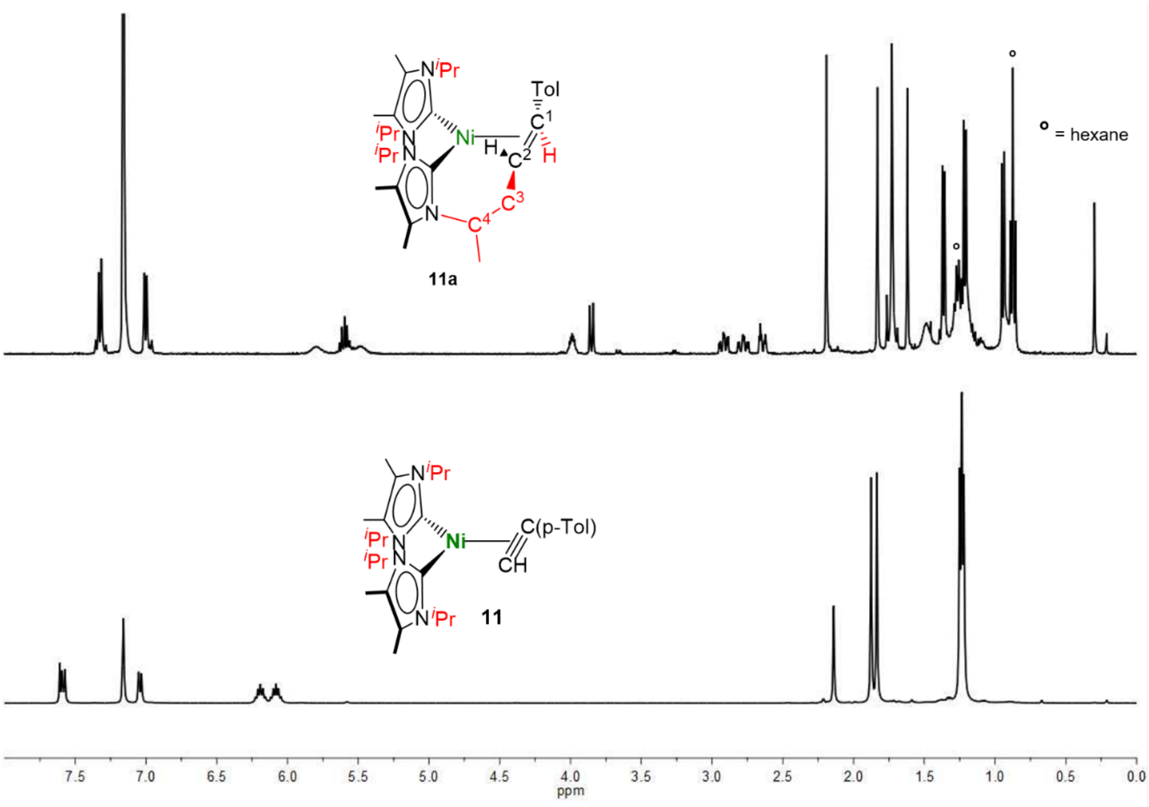

Figure S36. Comparison of the ^{1}H NMR spectra of [Ni(^{i}Pr$_2$Im^{Me})$_2$(η^2-HC≡C(p-Tol))] **11** (bottom) and the rearrangement product **11a** (top) (400MHz, 25 °C, C_6D_6).

Rearrangement product 12a

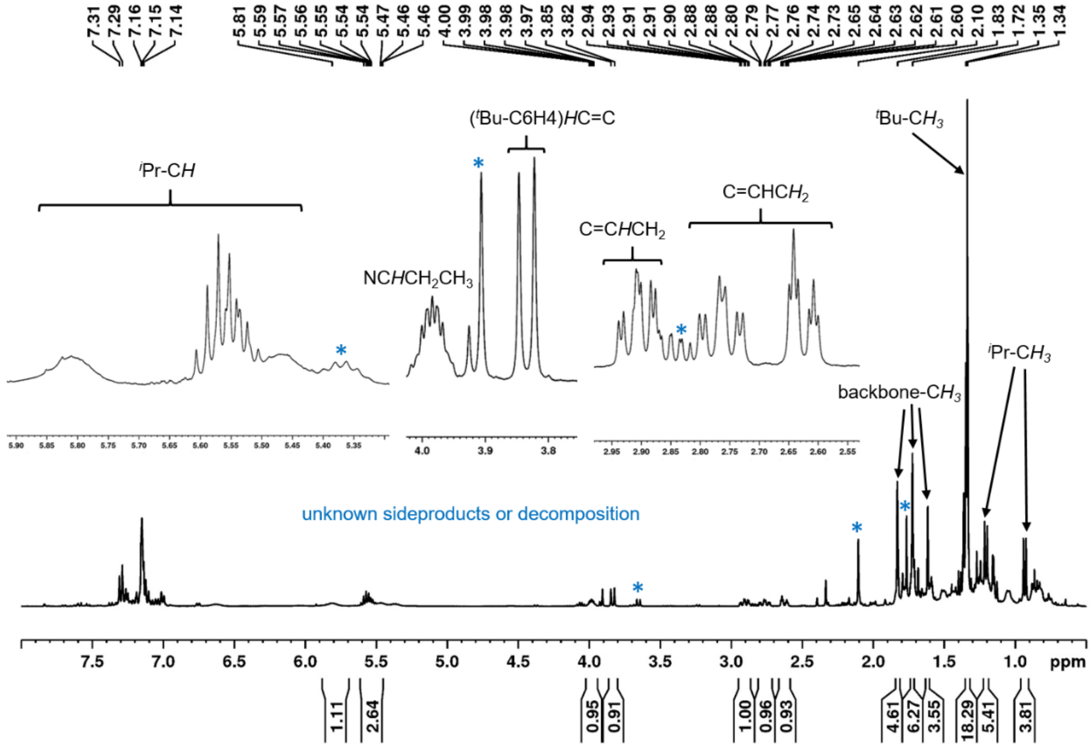

Figure S37. *In situ* ^{1}H NMR spectrum of the rearrangement product **12a** (400MHz, 25 °C, C_6D_6).

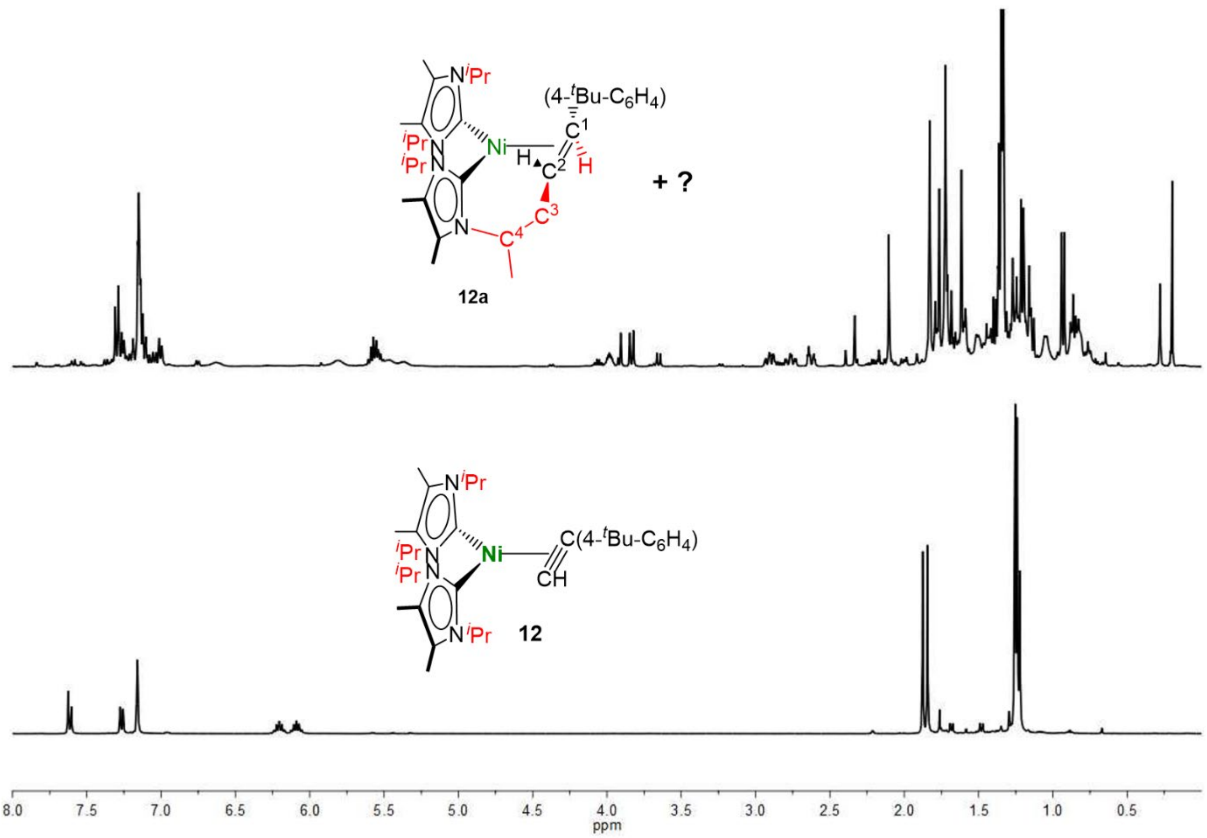

Figure S38. Comparison of the ^{1}H NMR spectra of [Ni(^{i}Pr$_2$Im^{Me})$_2$(η^2-HC≡C(4-^{t}Bu-C$_6$H$_4$))] **12** (bottom) and the rearrangement product **12a** (top) (400MHz, 25 °C, C_6D_6).

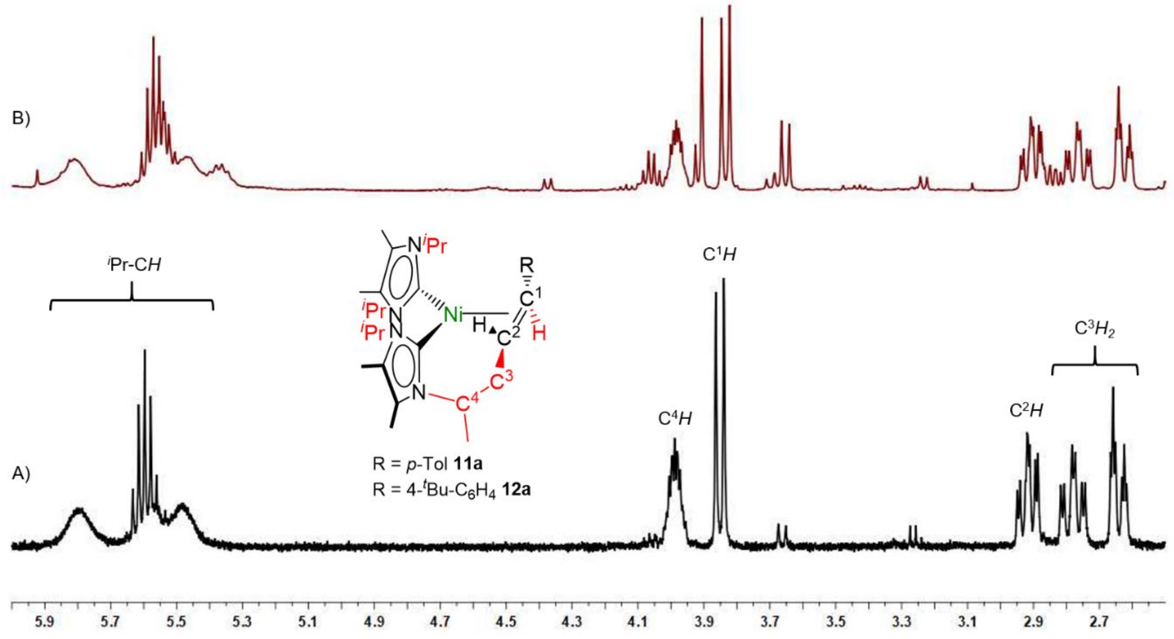

Figure S39. Part of the ^{1}H NMR spectra of the rearrangement products **11a** (A, bottom) and **12a** (B, top) (400MHz, 25 °C, C_6D_6).

Rearrangement product D-11a

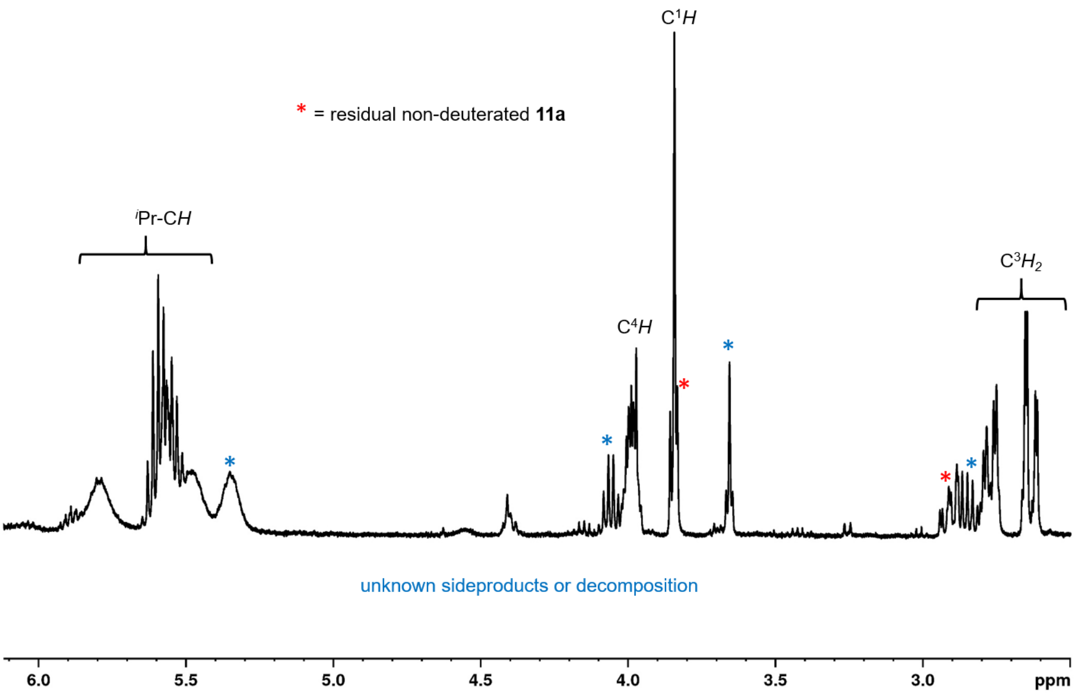

Figure S40. Part of the *in situ* ^{1}H NMR spectrum of **D-11a.** The hydrogen atom at C^1 appears as a singlet, since the coupling to the deuterium atom on C^2 is not resolved. (400MHz, 25 °C, C_6D_6).

[Ni(Mes$_2$Im)$_2$(η^2-MeC≡CMe) 14

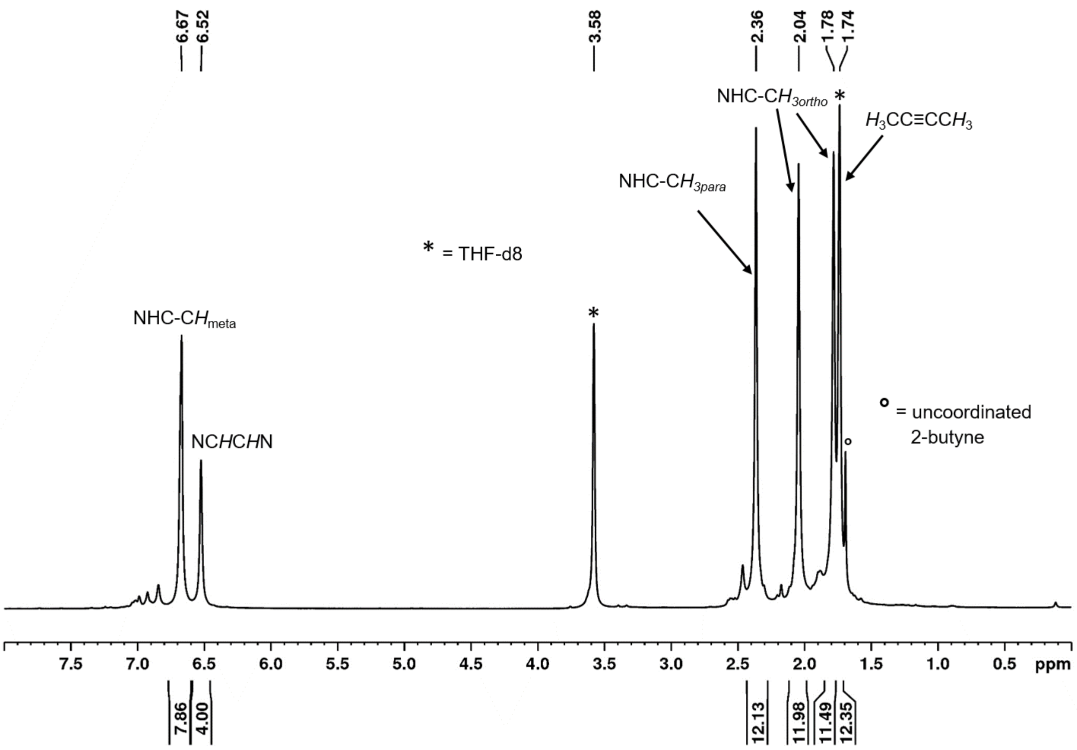

Figure S41. ^{1}H NMR spectrum of [Ni(Mes$_2$Im)$_2$(η^2-MeC≡CMe) **14** (400MHz, -80 °C, THF-d_8).

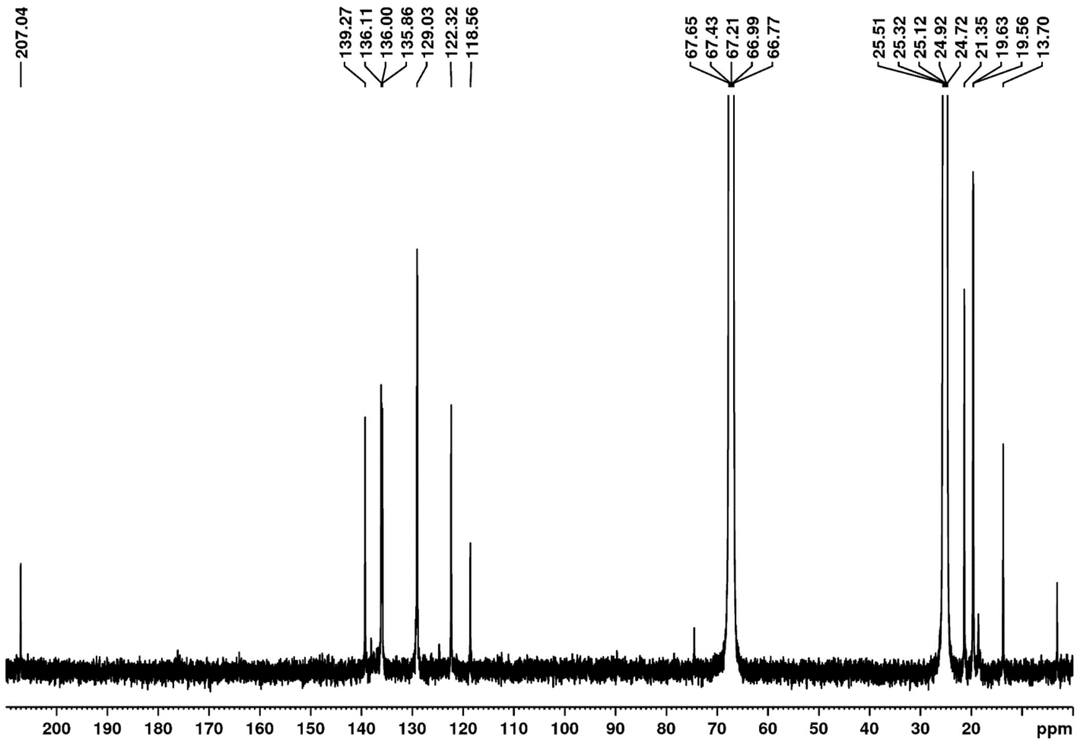

Figure S42. $^{13}C\{^{1}H\}$ NMR spectrum of [Ni(Mes$_2$Im)$_2$(η^2-MeC≡CMe) **14** (400MHz, -80 °C, THF-d$_8$).

[Ni(Mes$_2$Im)$_2$(η^2-MeOOCC≡CCOOMe) 15

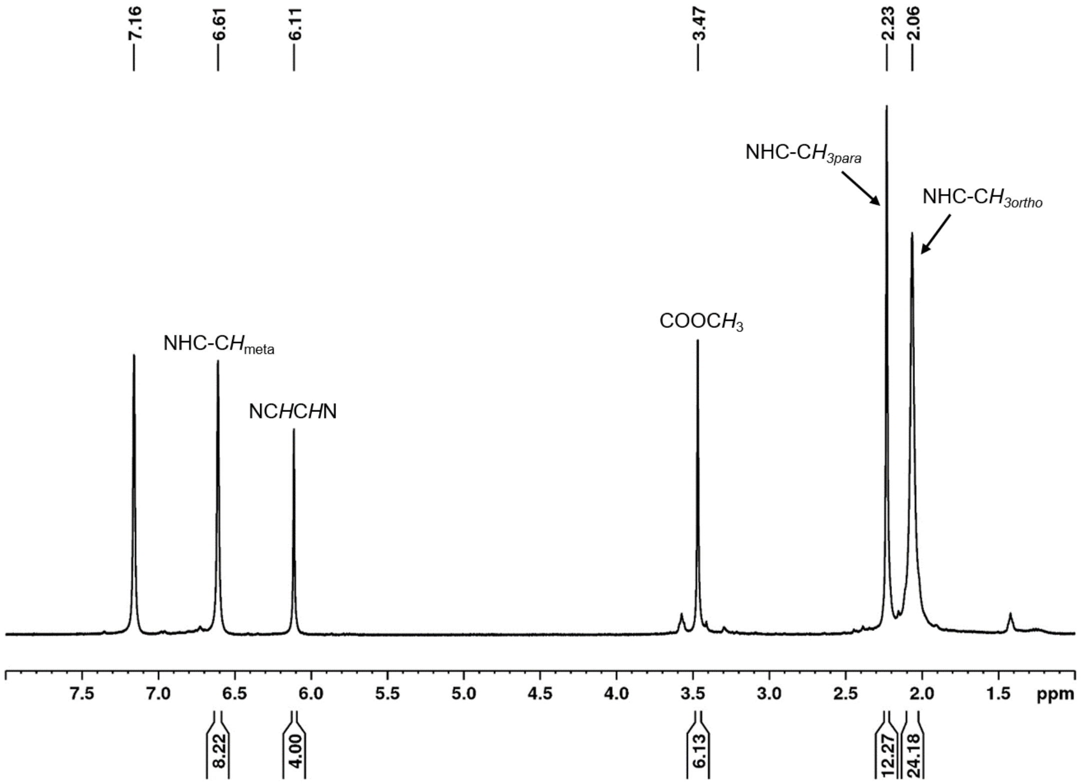

Figure S43. ^{1}H NMR spectrum of [Ni(Mes$_2$Im)$_2$(η^2-MeOOCC≡CCOOMe) **15** (400MHz, 25 °C, C$_6$D$_6$).

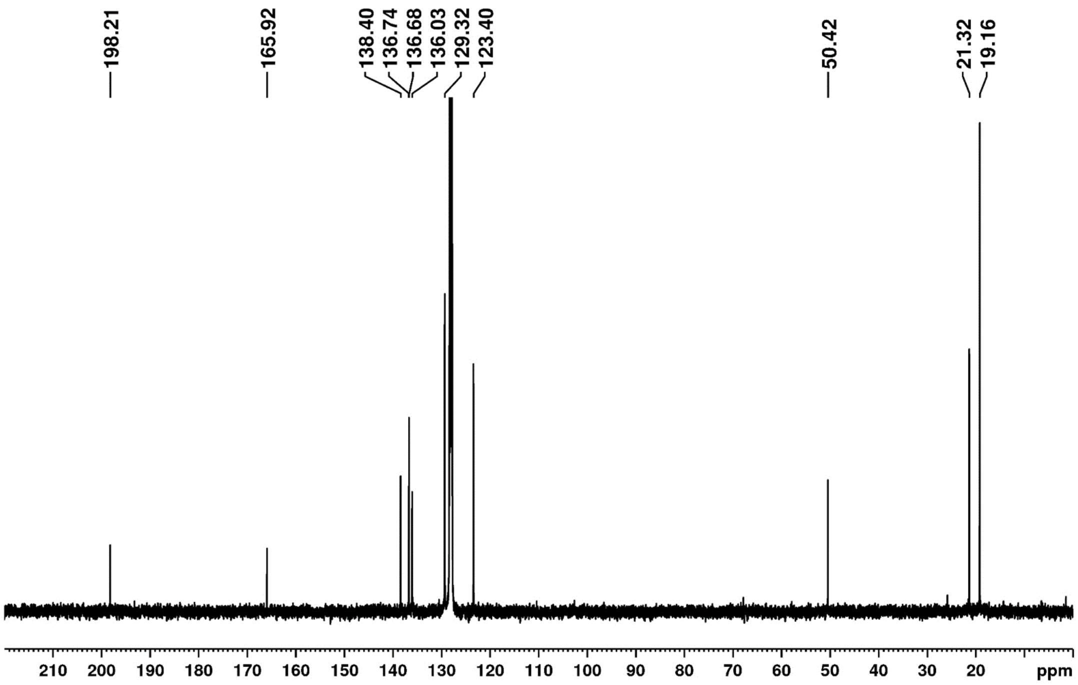

Figure S44. $^{13}C\{^{1}H\}$ NMR spectrum of $[Ni(Mes_2Im)_2(\eta^2\text{-}MeOOCC{\equiv}CCOOMe)$ **15** (100MHz, 25 °C, C_6D_6).

$[Ni(Mes_2Im)_2(\eta^2\text{-}PhC{\equiv}CMe)$ 16

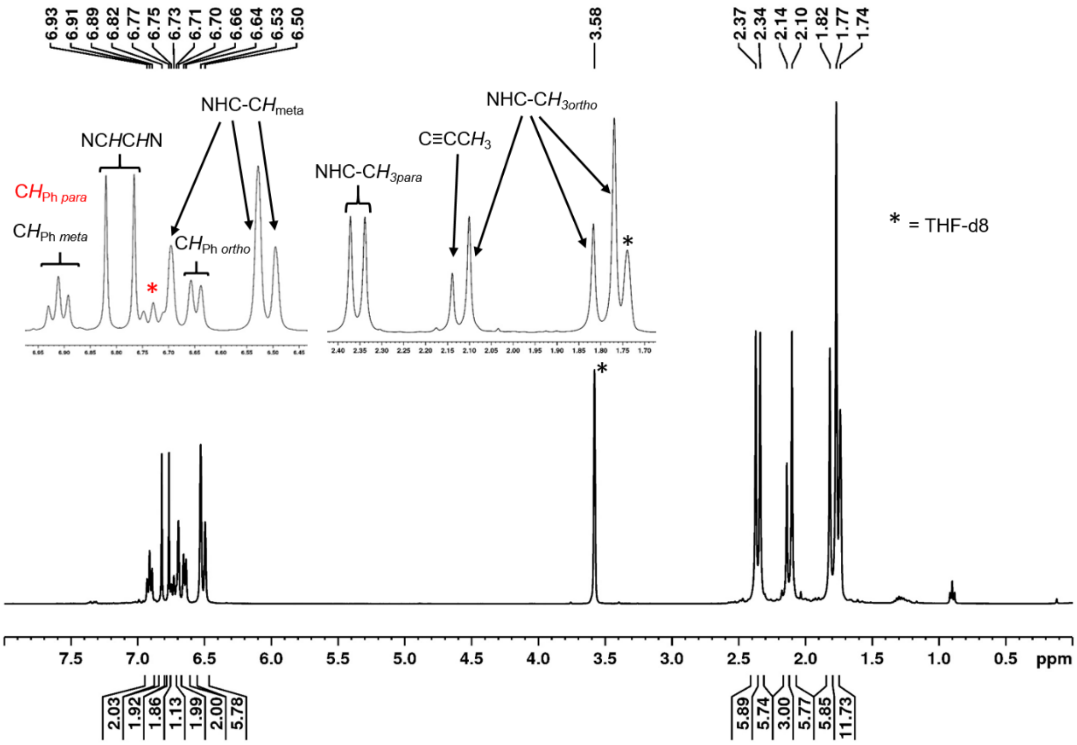

Figure S45. ^{1}H NMR spectrum of $[Ni(Mes_2Im)_2(\eta^2\text{-}PhC{\equiv}CMe)$ **16** (400MHz, -80 °C, THF-d_8).

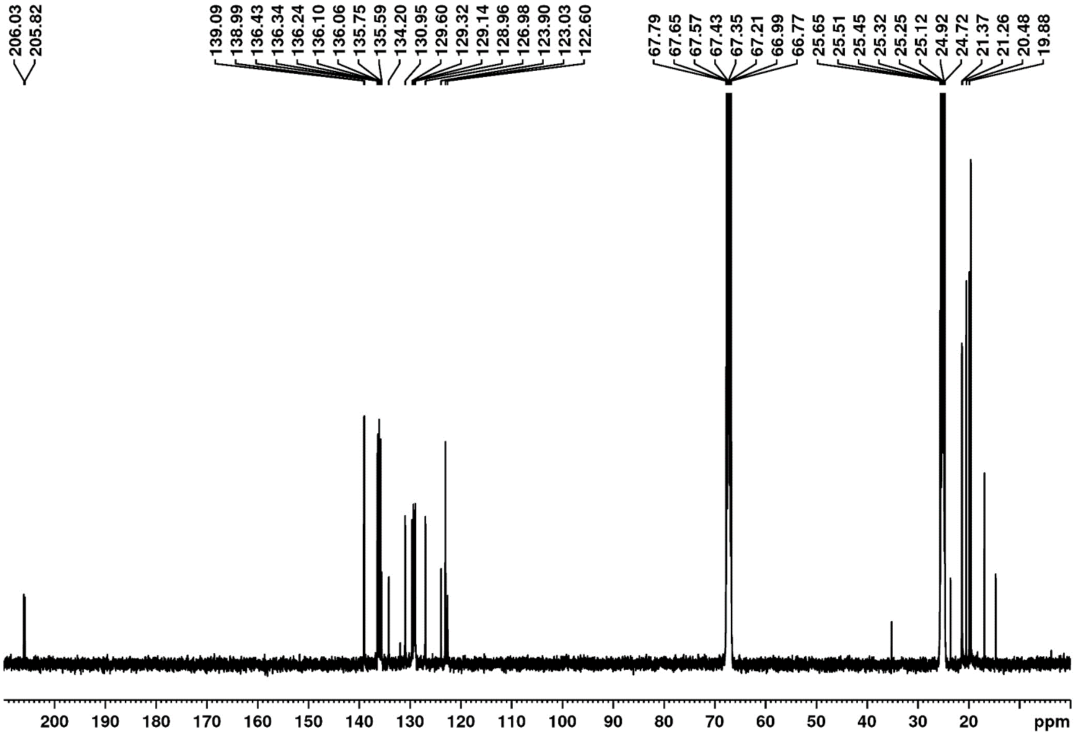

Figure S46. $^{13}C\{^1H\}$ NMR spectrum of [Ni(Mes$_2$Im)$_2$(η^2-PhC≡CMe) **16** (400MHz, -80 °C, THF-d$_8$).

[Ni(Mes$_2$Im)$_2$(η^2-HC≡C(4-^{t}Bu-C$_6$H$_4$))] 17

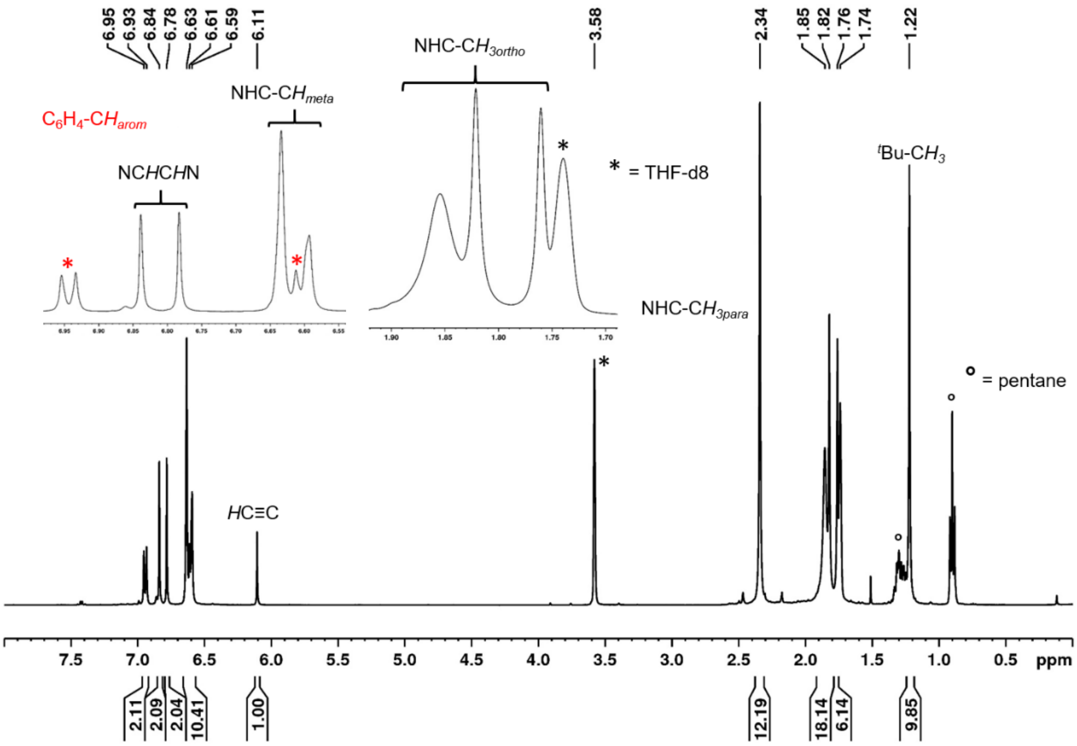

Figure S47. ^1H NMR spectrum of [Ni(Mes$_2$Im)$_2$(η^2-HC≡C(4-^{t}Bu-C$_6$H$_4$))] **17** (400MHz, - 80 °C, THF-d$_8$).

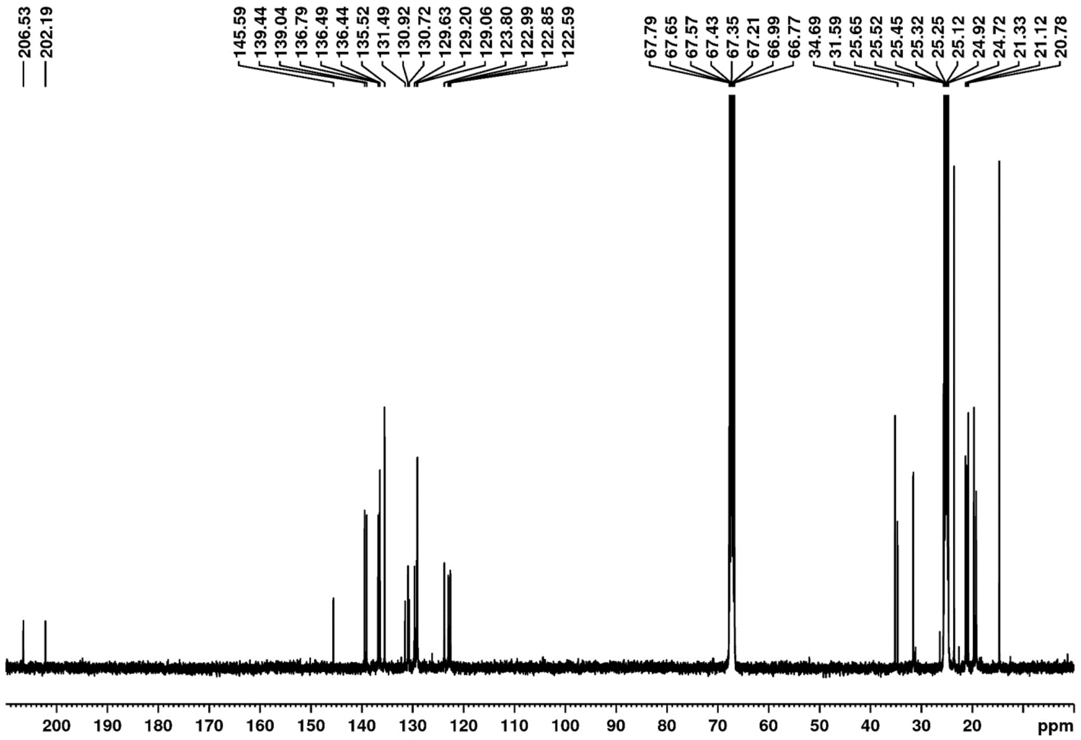

Figure S48. $^{13}C\{^1H\}$ NMR spectrum of [Ni(Mes$_2$Im)$_2$(η^2-HC≡C(4-^{t}Bu-C$_6$H$_4$))] **17** (400MHz, - 80 °C, THF-d$_8$).

[Ni(Mes$_2$Im)$_2$(η^2-HC≡CCOOMe)] 18

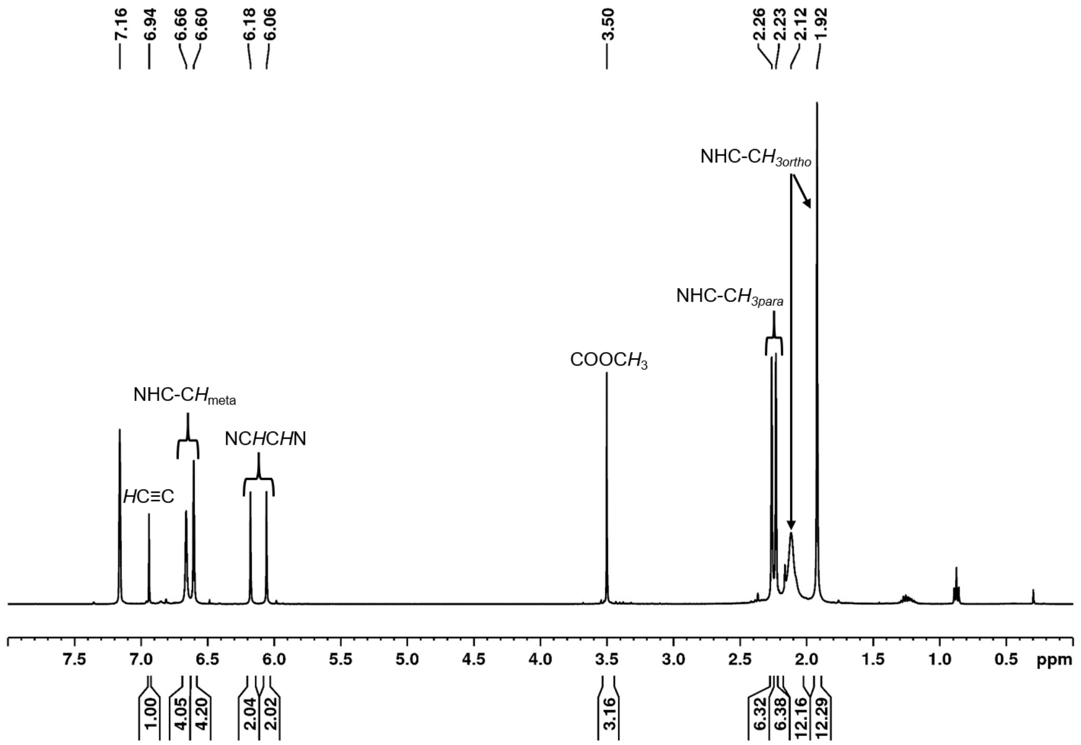

Figure S49. ^1H NMR spectrum of [Ni(Mes$_2$Im)$_2$(η^2-HC≡CCOOMe)] **18** (400MHz, 25 °C, C$_6$D$_6$).

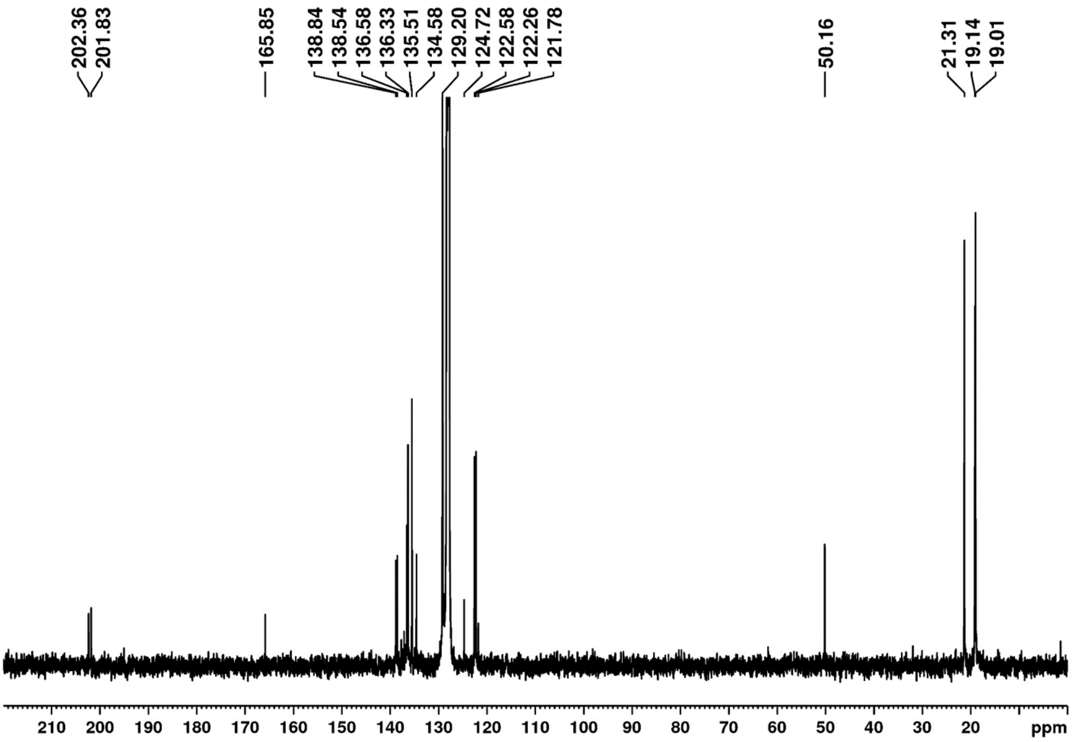

Figure S50. $^{13}C\{^1H\}$ NMR spectrum of [Ni(Mes$_2$Im)$_2$(η^2-HC≡CCOOMe)] **18** (100MHz, 25 °C, C_6D_6).

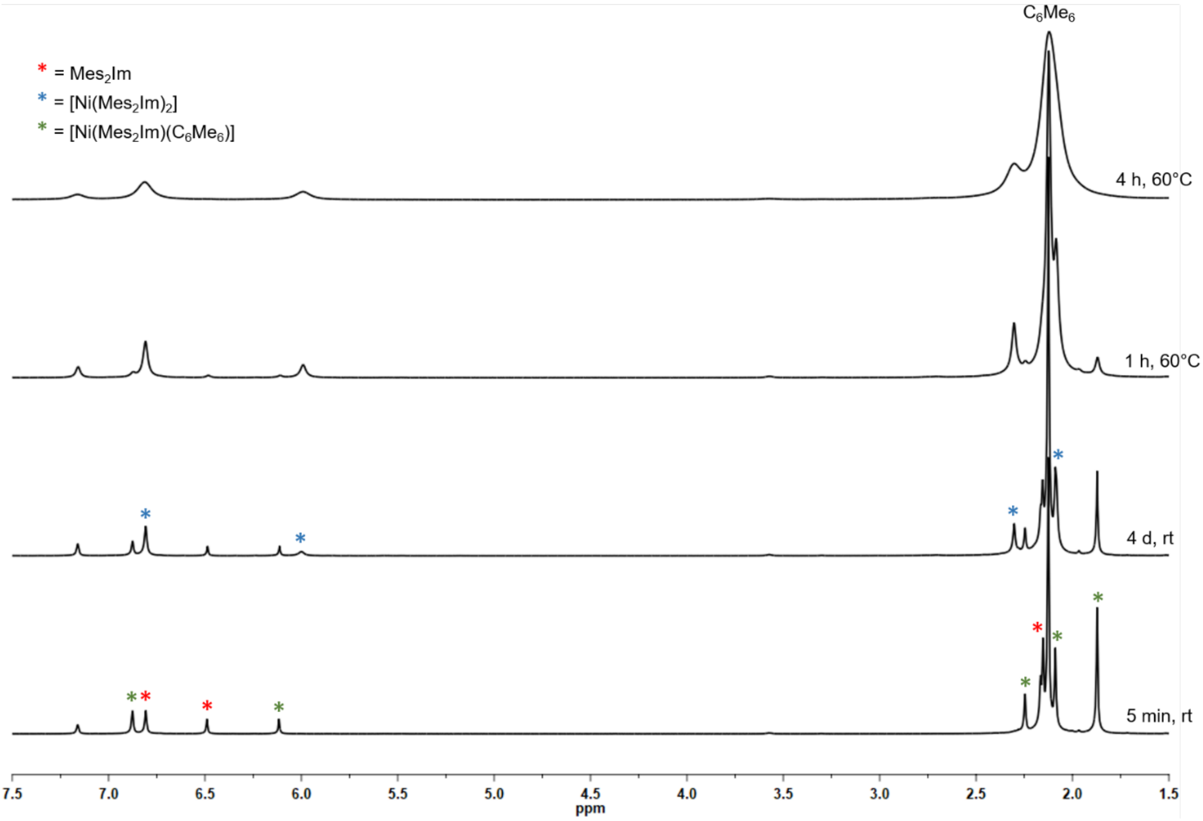

Figure S51. Time resolved ^1H NMR spectrum of the reaction of [Ni(Mes$_2$Im)$_2$] **2** with 2-butyne (3 equiv.) (400MHz, 25 °C, C_6D_6).

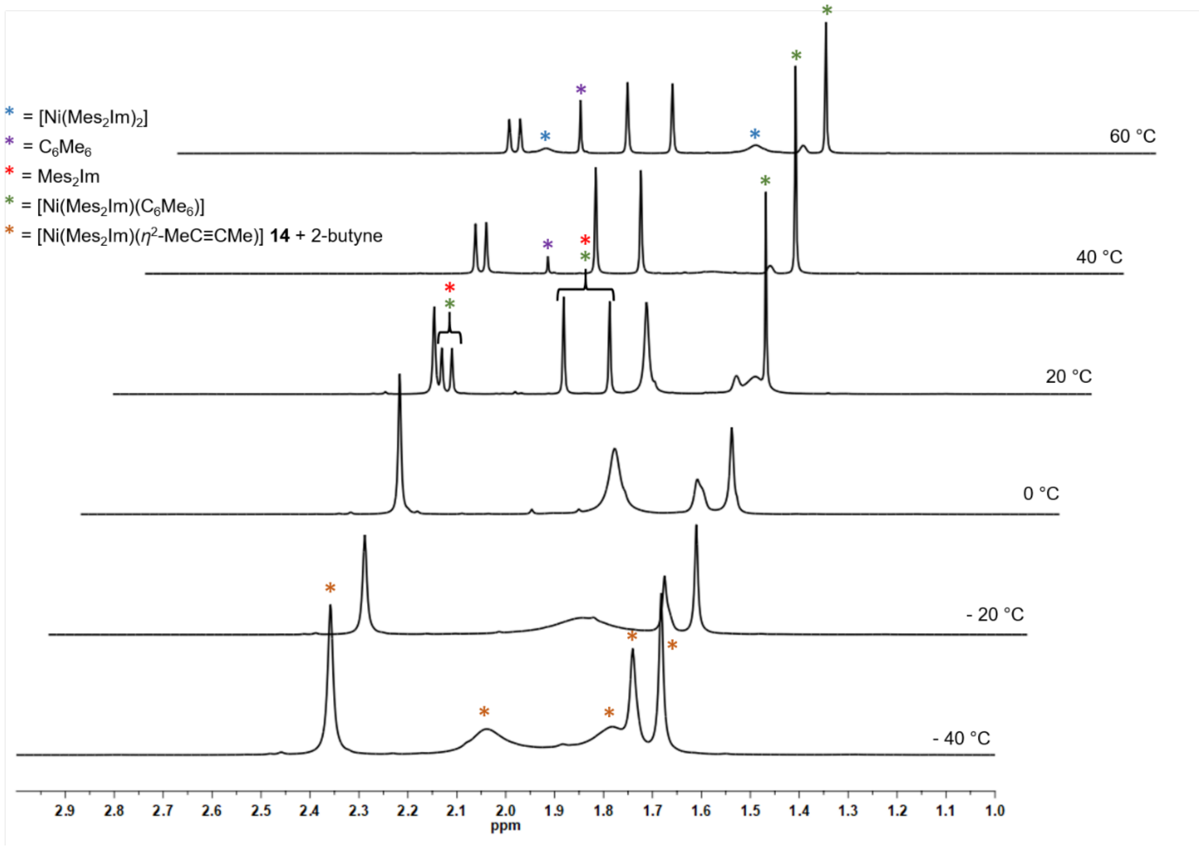

Figure S52. Variable temperature ^{1}H NMR spectrum of the reaction of [Ni(Mes$_2$Im)$_2$] **2** with 2-butyne (3 equiv.) (400MHz, 25 °C, THF-d$_8$).

Hexamethylbenzene

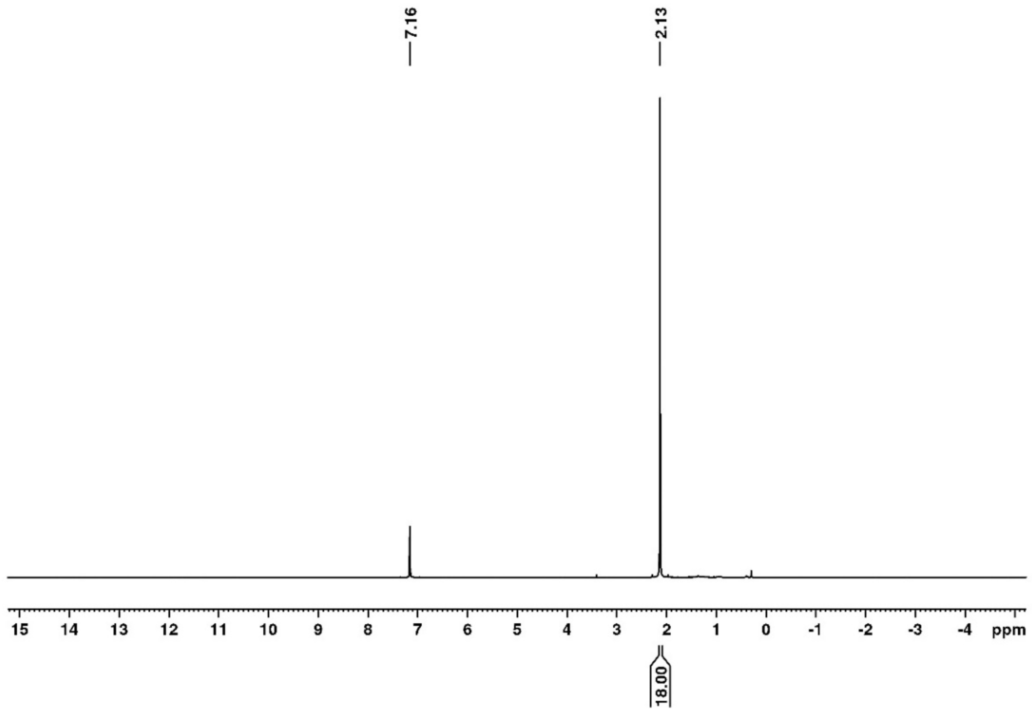

Figure S53. ^{1}H NMR spectrum of hexamethylbenzene (400MHz, 25 °C, C_6D_6).

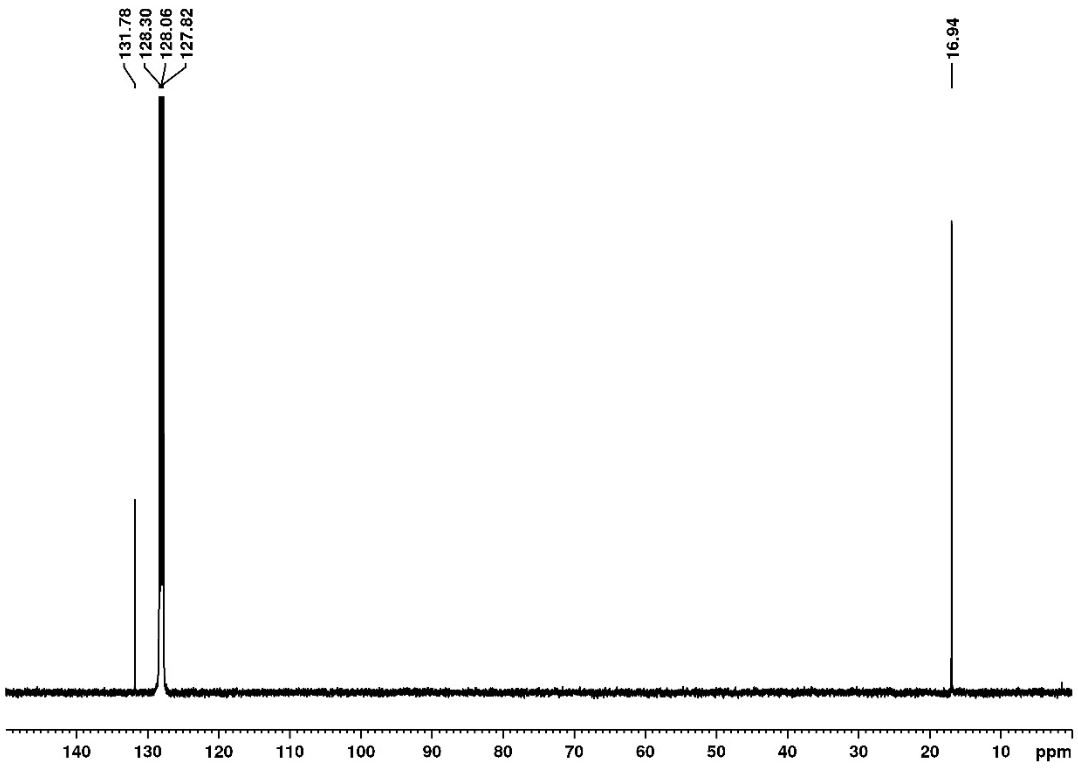

Figure S54. $^{13}C\{^{1}H\}$ NMR spectrum of hexamethylbenzene (100MHz, 25 °C, C_6D_6).

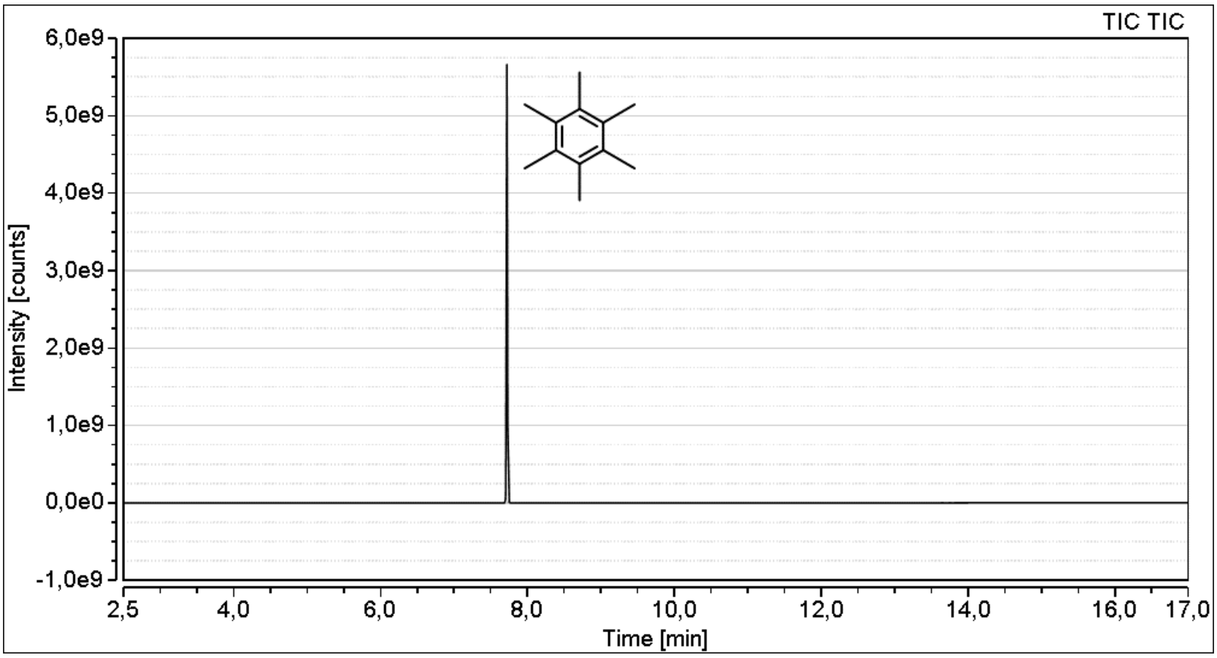

Figure S55. GC/MS trace of the cyclotrimerization of 2-butyne after removal of the catalyst.

1,2,4-Triphenylbenzene and 1,3,5-triphenylbenzene

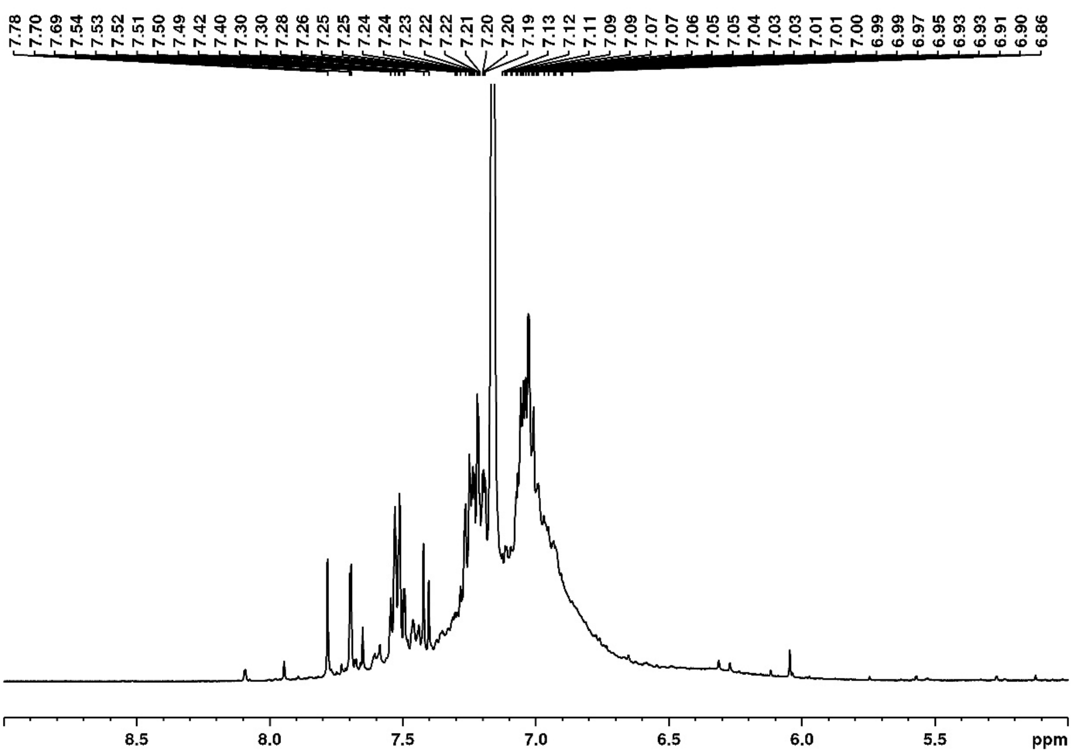

Figure S56. ^{1}H NMR spectrum of a mixture of 1,2,4- and 1,3,5-triphenylbenzene (400MHz, 25 °C, C_6D_6).

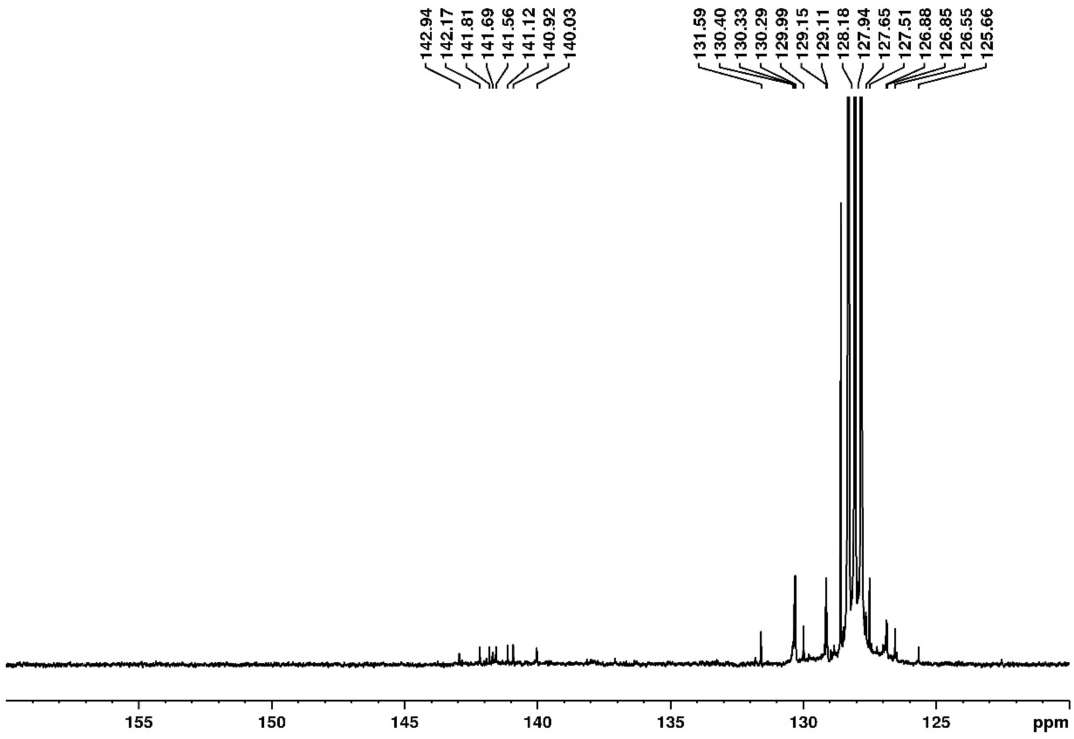

Figure S57. $^{13}C\{^{1}H\}$ NMR spectrum of a mixture of 1,2,4- and 1,3,5-triphenylbenzene (100MHz, 25 °C, C_6D_6).

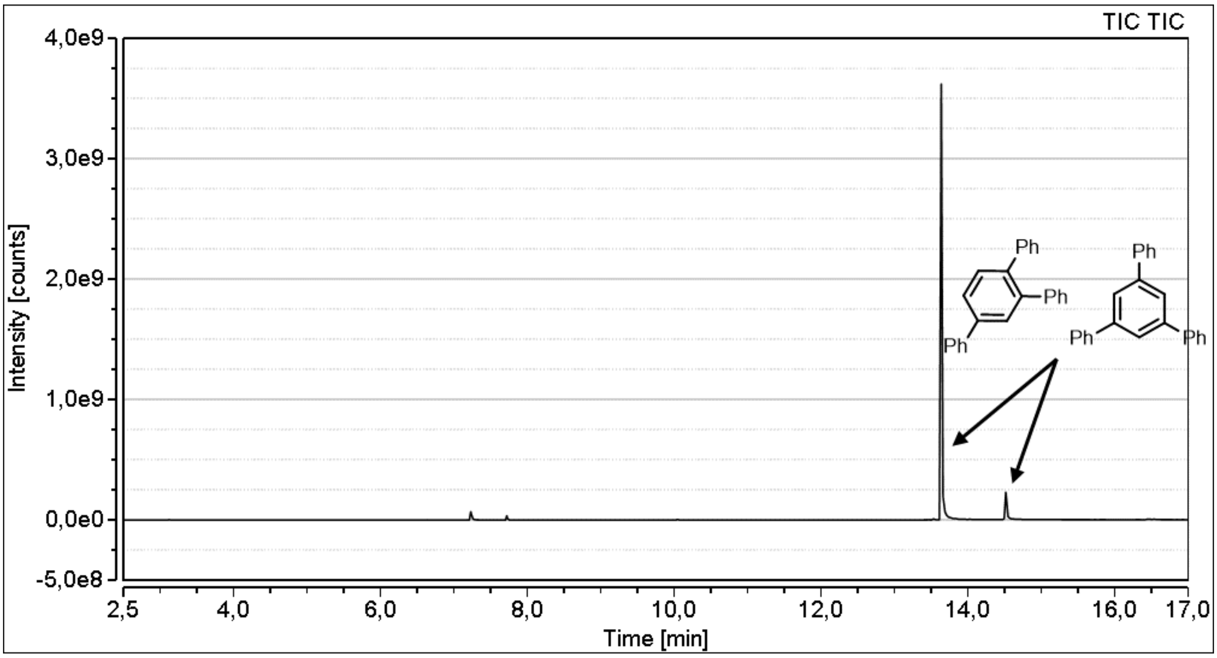

Figure S58. GC/MS trace of the cyclotrimerization of phenylacetylene after removal of the catalyst.

1,2,4-Tripropylbenzene and 1,3,5-tripropyllbenzene

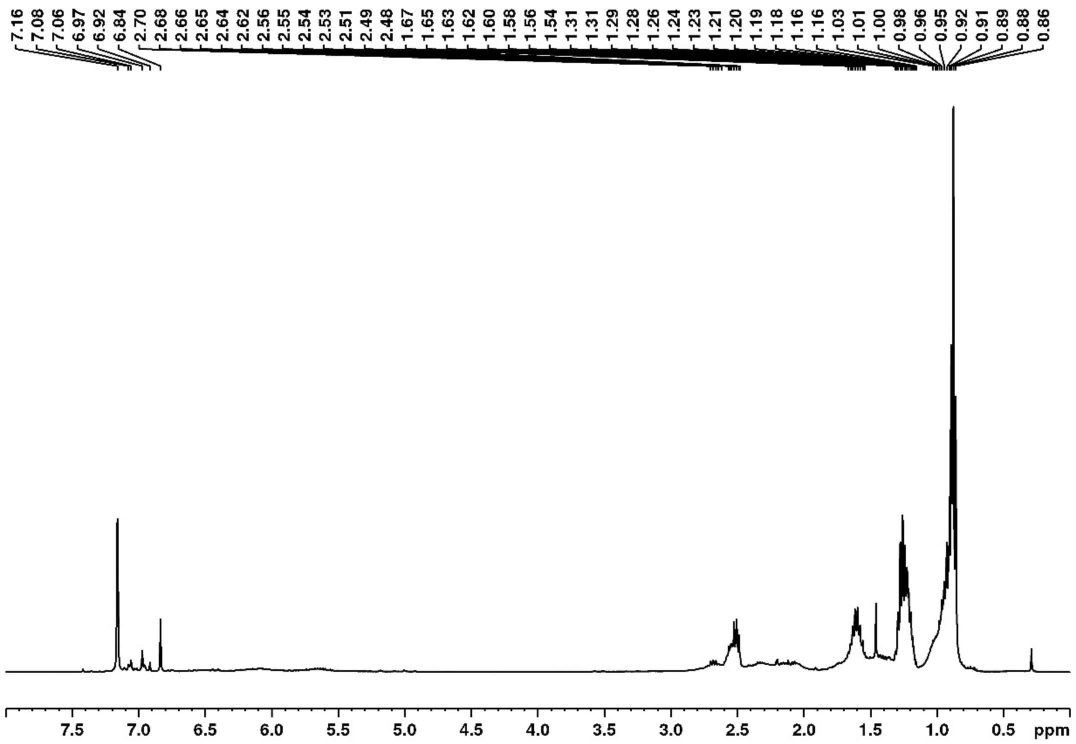

Figure S59. ^{1}H NMR spectrum of a mixture of 1,2,4- and 1,3,5-tripropyllbenzene (400MHz, 25 °C, C_6D_6).

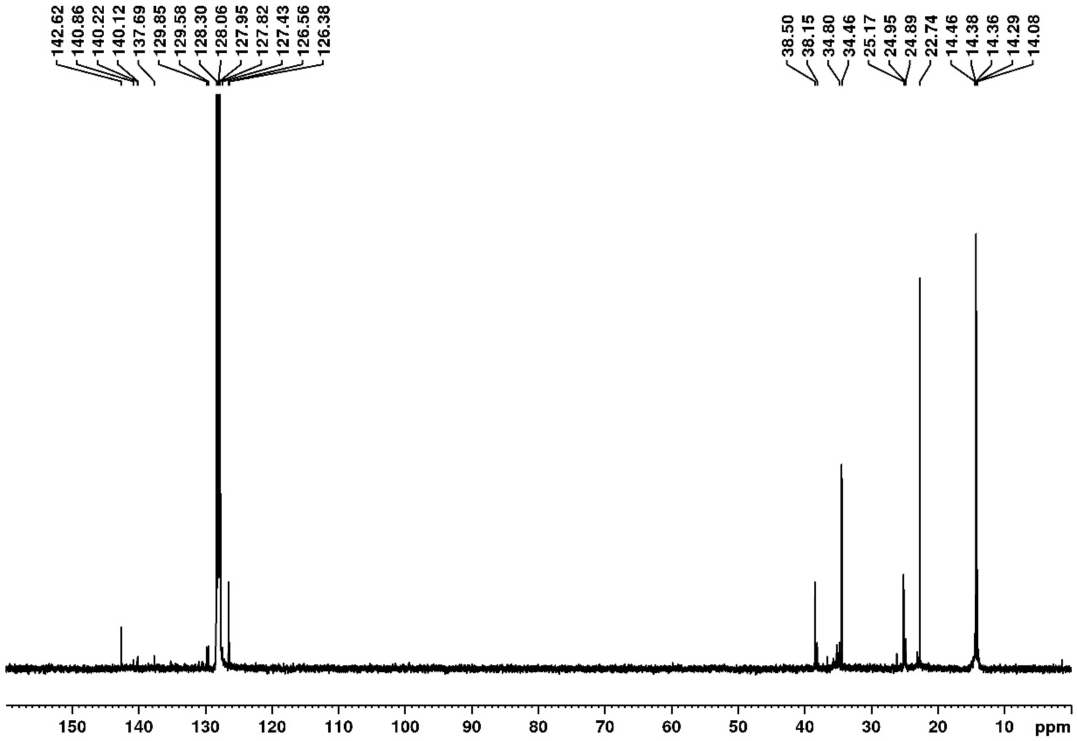

Figure S60. $^{13}C\{^{1}H\}$ NMR spectrum of a mixture of 1,2,4- and 1,3,5-tripropyllbenzene (100MHz, 25 °C, C_6D_6).

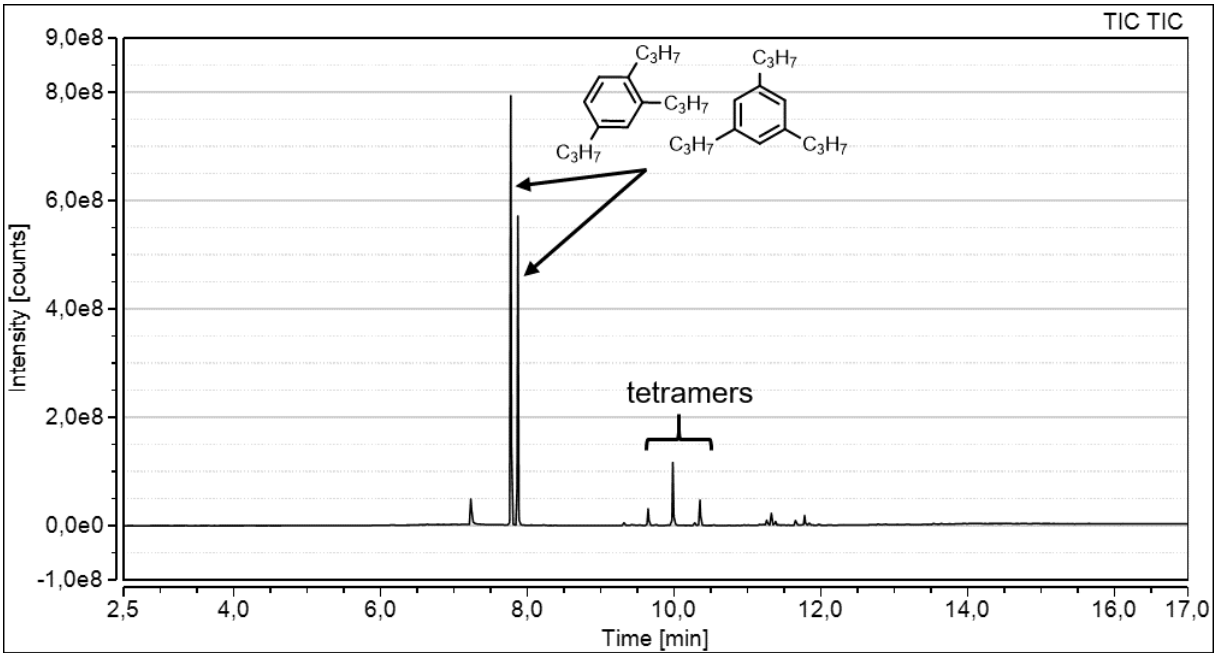

Figure S61. GC/MS trace of the cyclotrimerization of 1-pentyne after removal of the catalyst.

Hexapropylbenzene

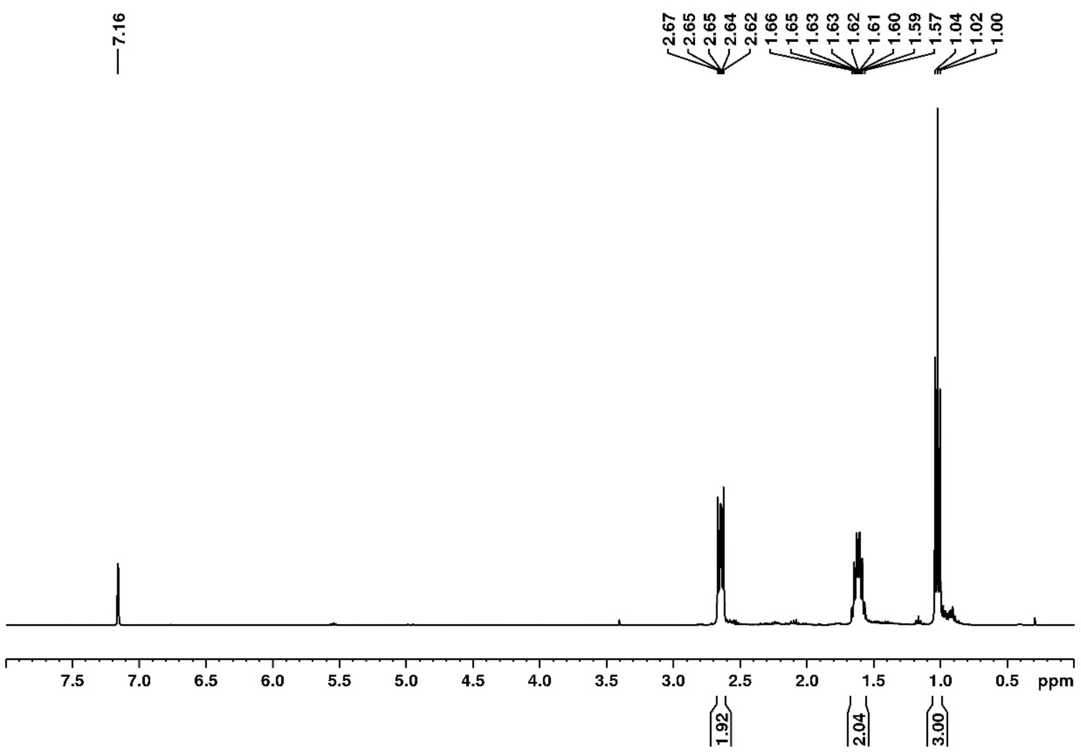

Figure S62. ^{1}H NMR spectrum of hexapropylbenzene (400MHz, 25 °C, C_6D_6).

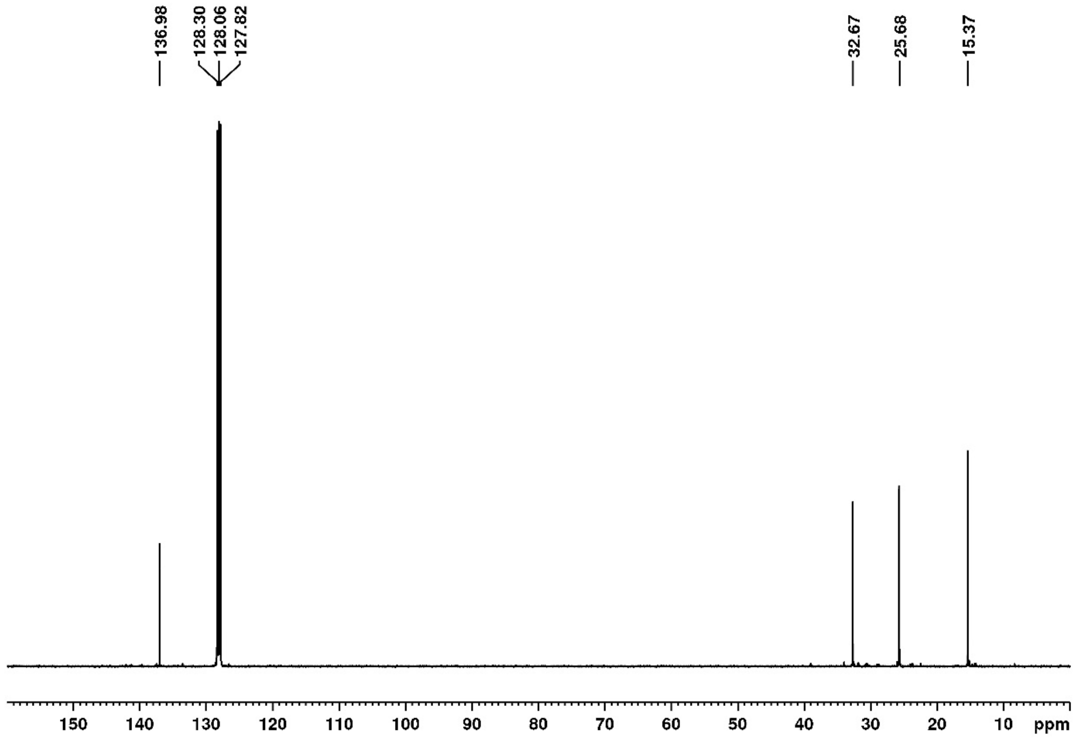

Figure S63. $^{13}C\{^{1}H\}$ NMR spectrum of hexapropylbenzene (100MHz, 25 °C, C_6D_6).

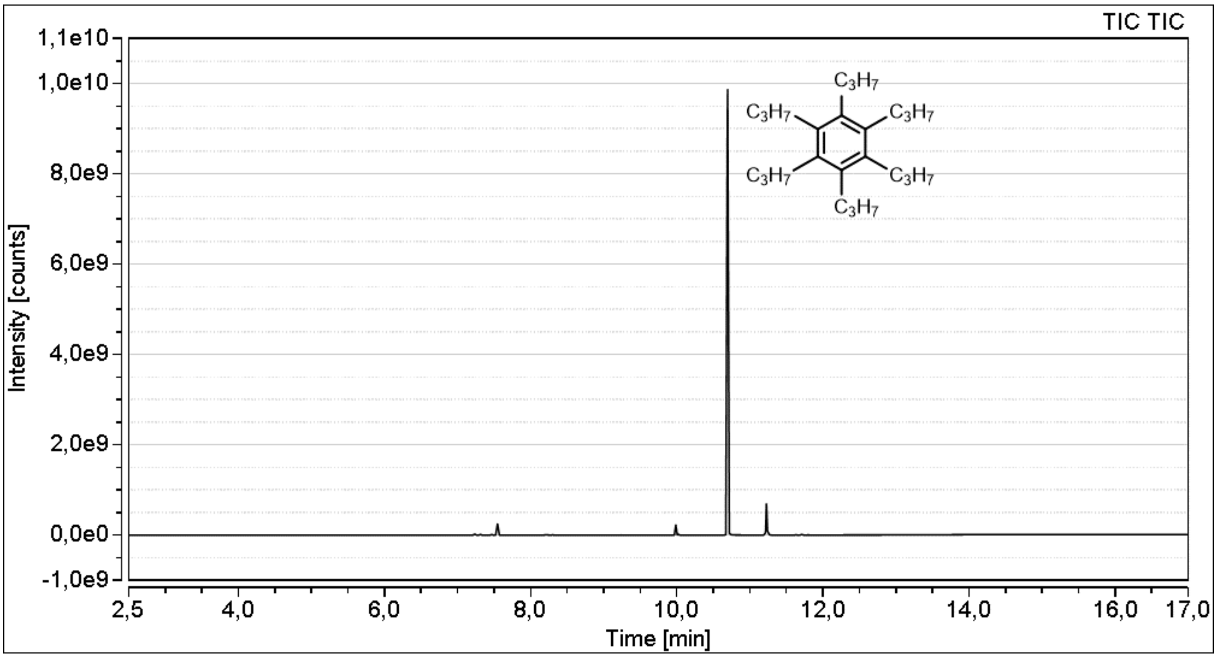

Figure S64. GC/MS trace of the cyclotrimerization of 4-octine after removal of the catalyst.

Trimethyl-1,2,4-benzenetricarboxylate and trimethyl-1,3,5-benzenetricarboxylate

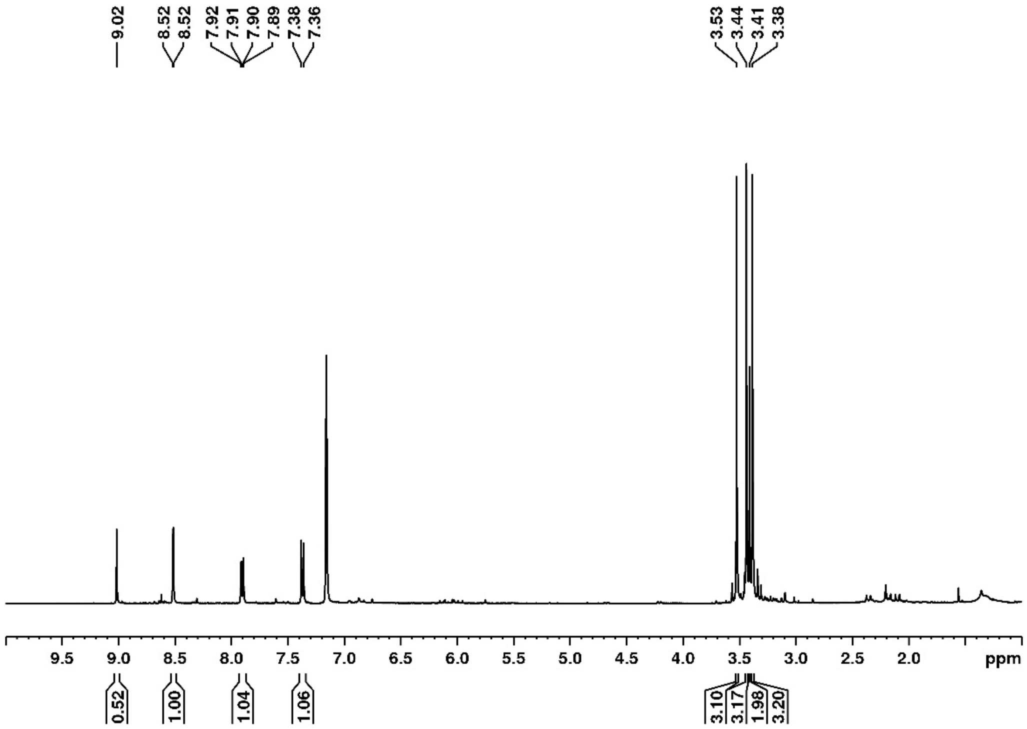

Figure S65. ^{1}H NMR spectrum of a mixture of trimethyl-1,2,4- and trimethyl-1,3,5-benzenetricarboxylate (400MHz, 25 °C, C_6D_6).

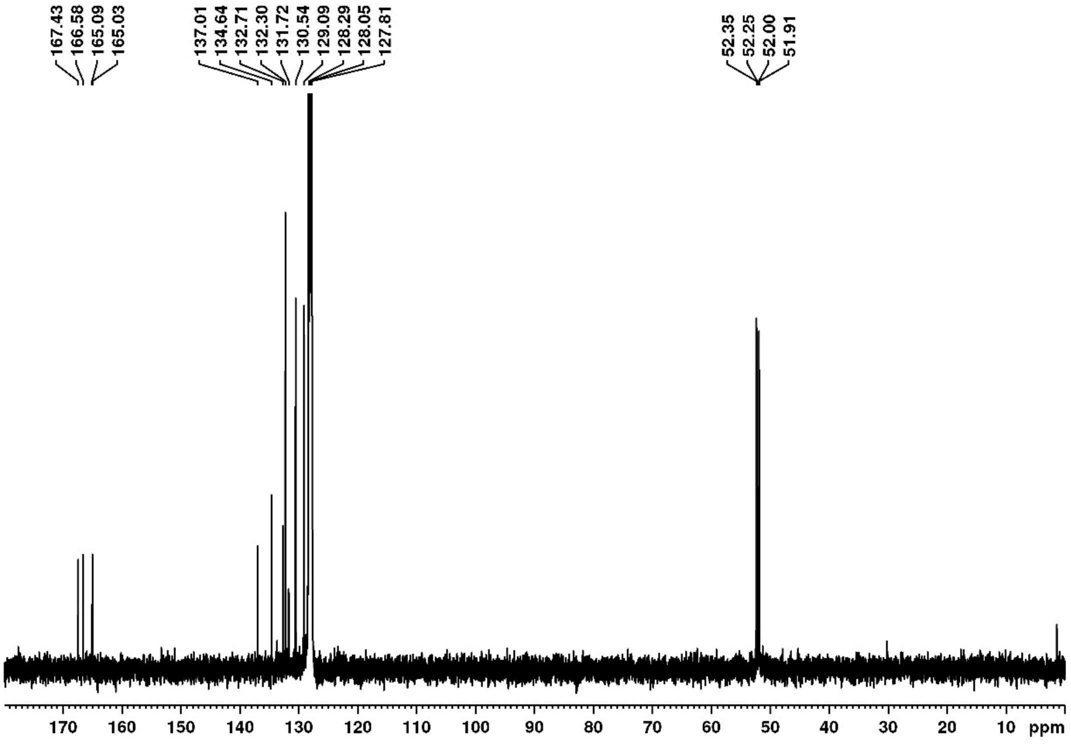

Figure S66. $^{13}C\{^1H\}$ NMR spectrum of a mixture of trimethyl-1,2,4- and trimethyl-1,3,5-benzenetricarboxylate (100MHz, 25 °C, C_6D_6).

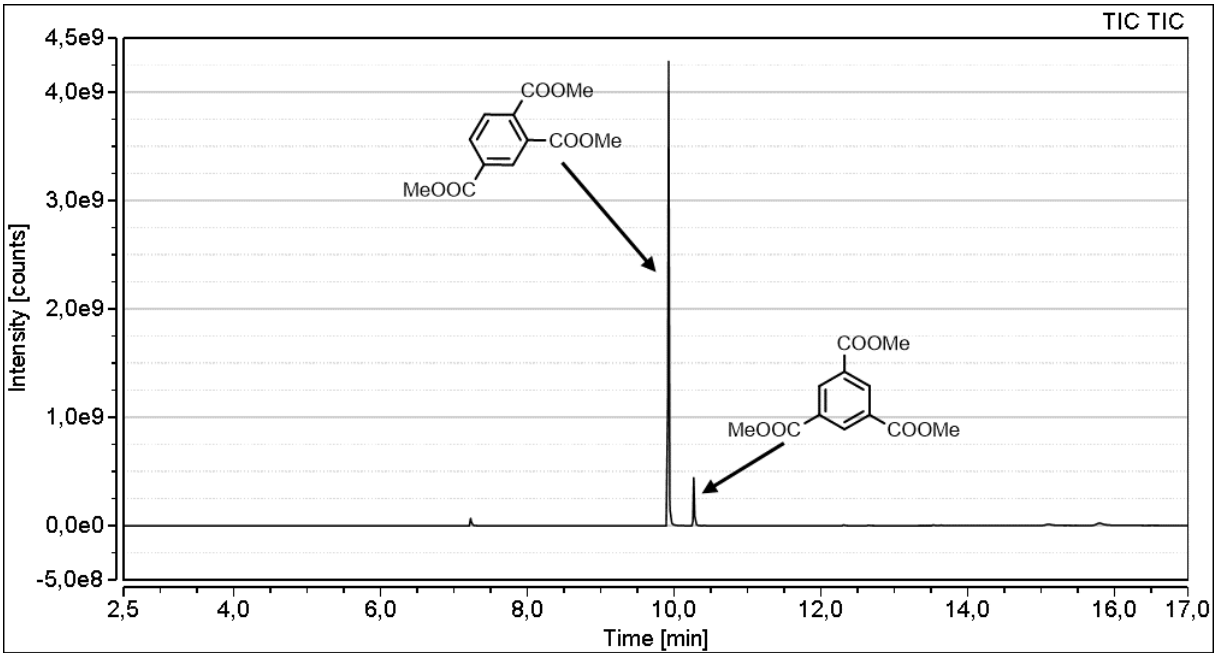

Figure S67. GC/MS trace of the cyclotrimerization of methylpropiolate after removal of the catalyst.

Hexamethyl-benzenehexacarboxylate

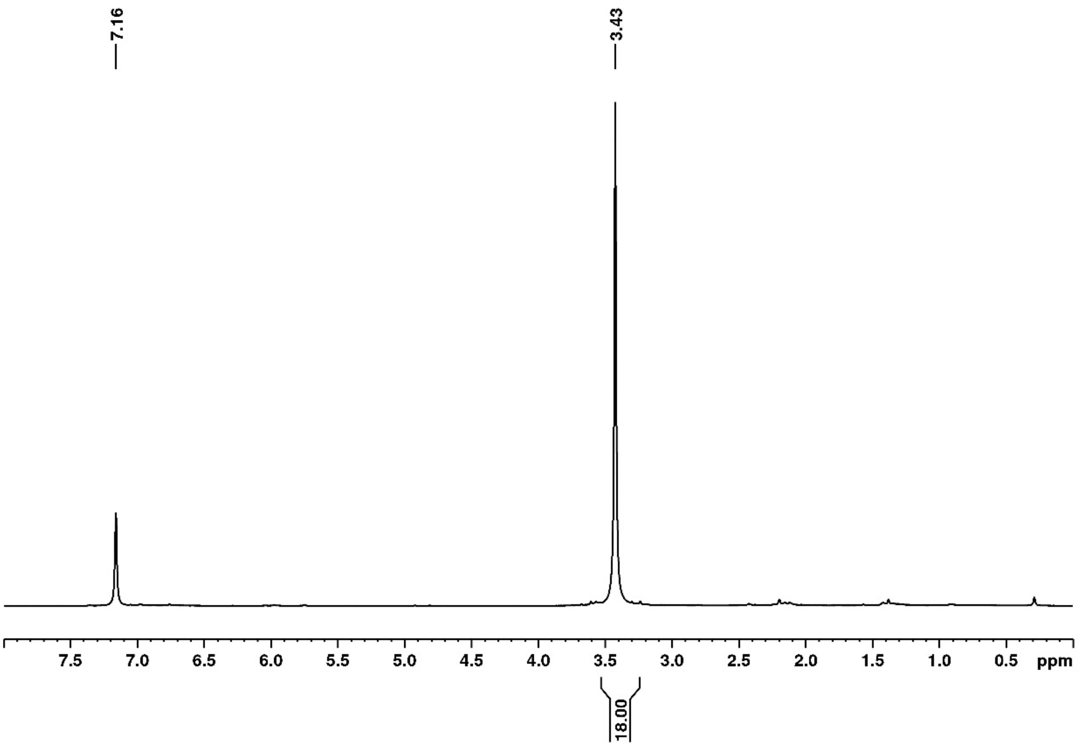

Figure S68. ^{1}H NMR spectrum of hexamethyl-benzenehexacarboxylate (400MHz, 25 °C, C_6D_6).

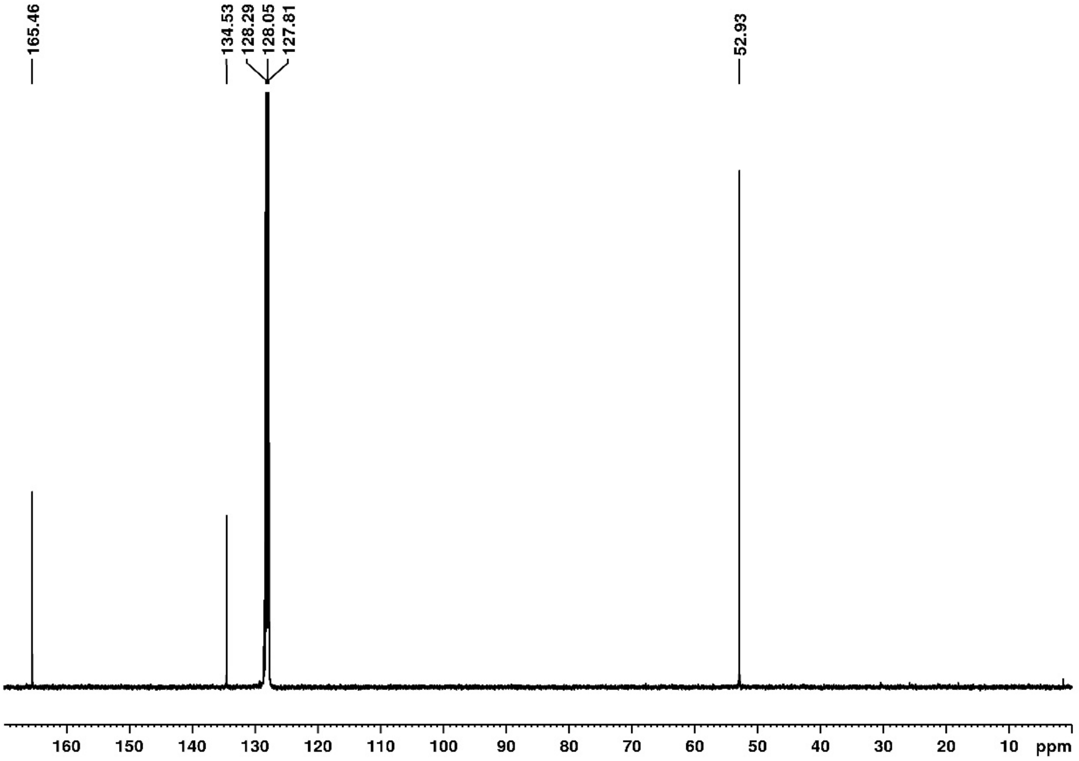

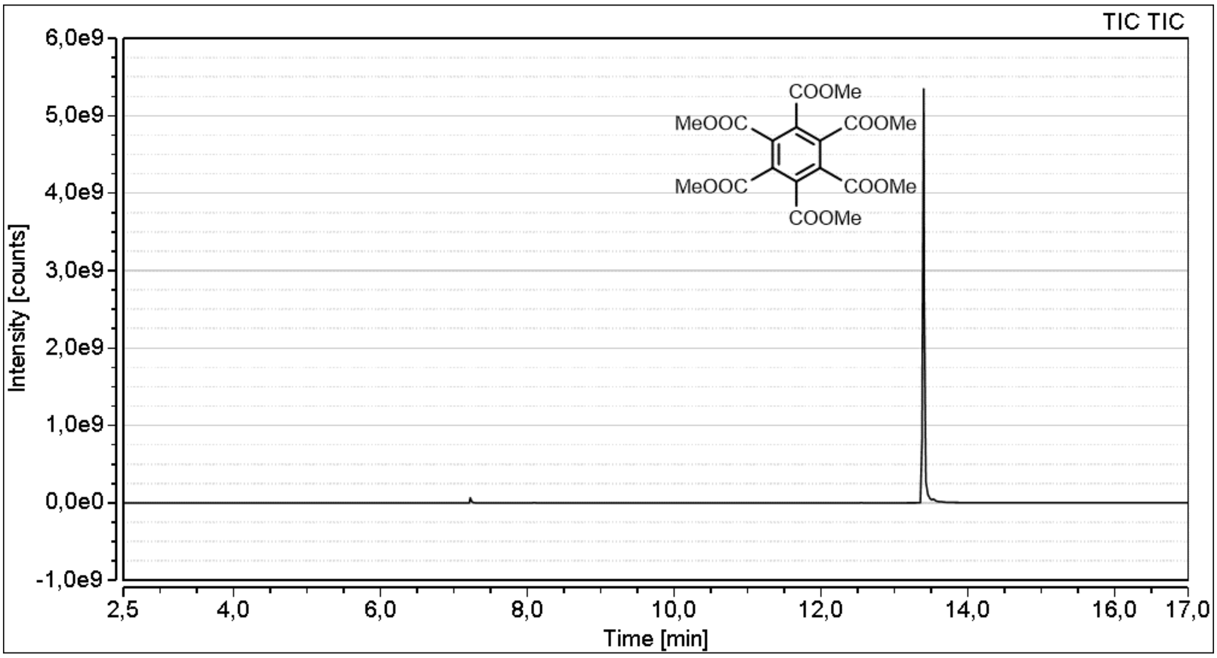

Figure S70. GC/MS trace of the cyclotrimerization of dimethyl acetylenedicarboxylate after removal of the catalyst.

Hexaphenylbenzene

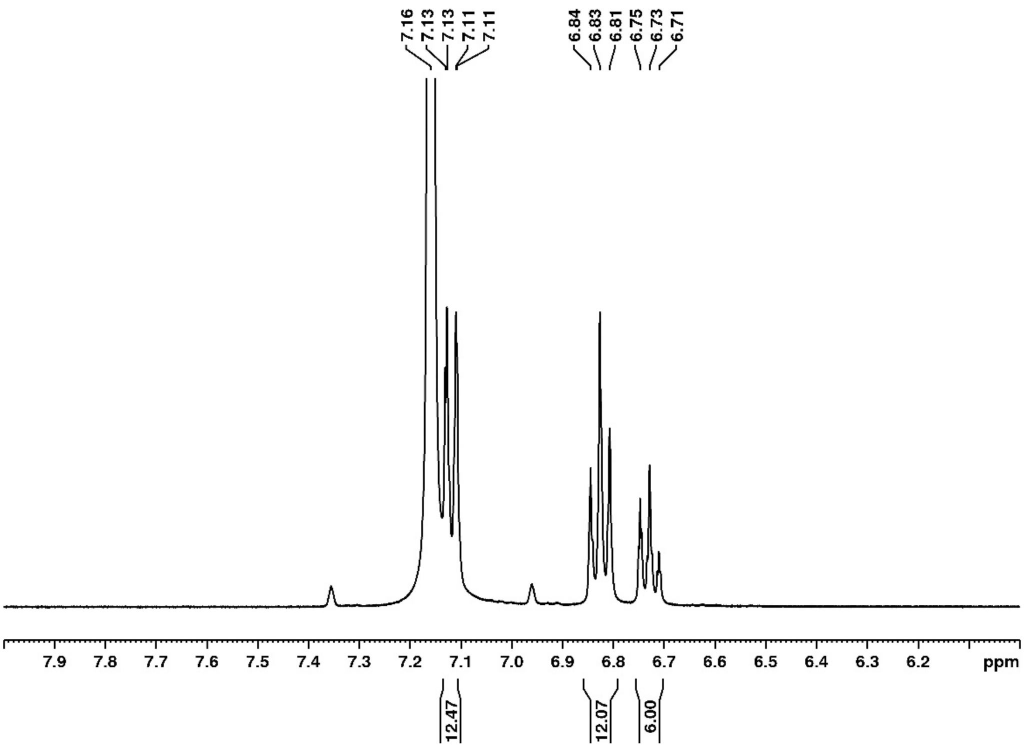

Figure S71. ^{1}H NMR spectrum of hexaphenylbenzene (400MHz, 25 °C, C_6D_6).

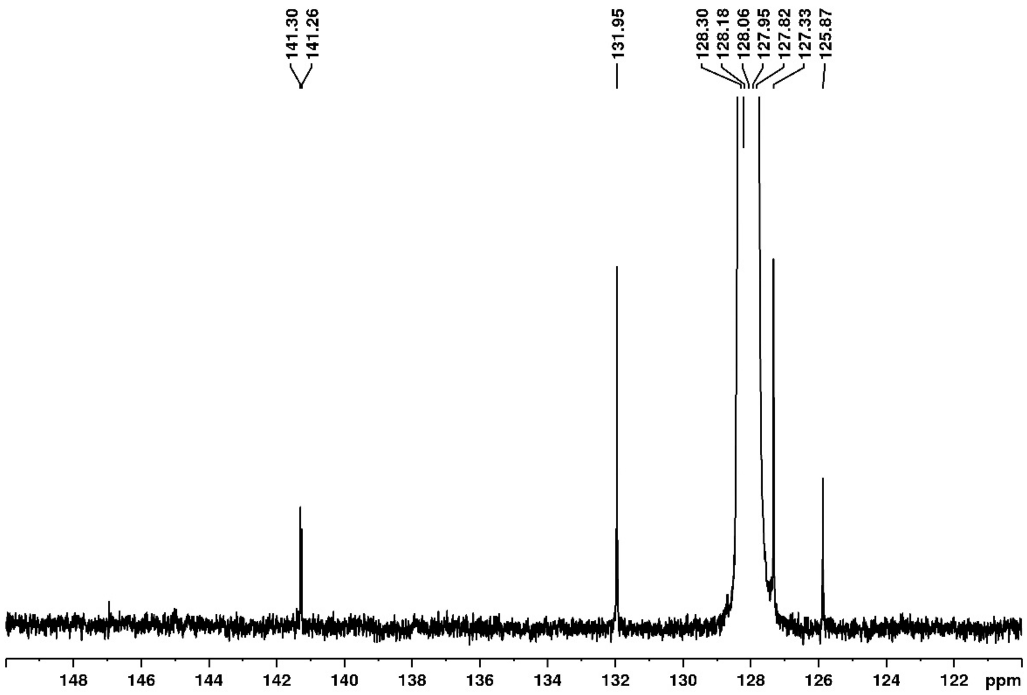

Figure S72. $^{13}C\{^1H\}$ NMR spectrum of hexaphenylbenzene (100MHz, 25 °C, C_6D_6).

4) Computational Details

Calculations were carried out using the TURBOMOLE V7.5.1 program suite, a development of the University of Karlsruhe and the Forschungszentrum Karlsruhe GmbH, 1989-2007, TURBOMOLE GmbH, since 2007; available from https://www.turbomole.org.[7] Geometry optimizations were performed using (RI-)DFT calculations[8] on a m4 grid employing the BP86 [9] functional and a def2-TZVP basis set for Ni, and def2-SVP basis sets for C and N. [10] For the carbonyl complexes [(NHC)Ni(CO)$_3$] a def2-TZVP basis set for all atoms eas employed.

Vibrational frequencies were calculated at the same level with the AOFORCE[11] module and all structures represented true minima without imaginary frequencies. Natural population analysis,[12] NBO[13] and Wiberg bond indices[14] have been evaluated from the DFT ground state electron density.

%*V*bur[15] and Steric Maps[16] of the idealized complexes [(NHC)Ni(CO)$_3$] (Ni–NHC distances fixed to 2.0 Å) were calculated with the aid of the web application Samb*V*ca[17] (Bondi radii scaled by 1.17, sphere radius R = 3.5 Å).

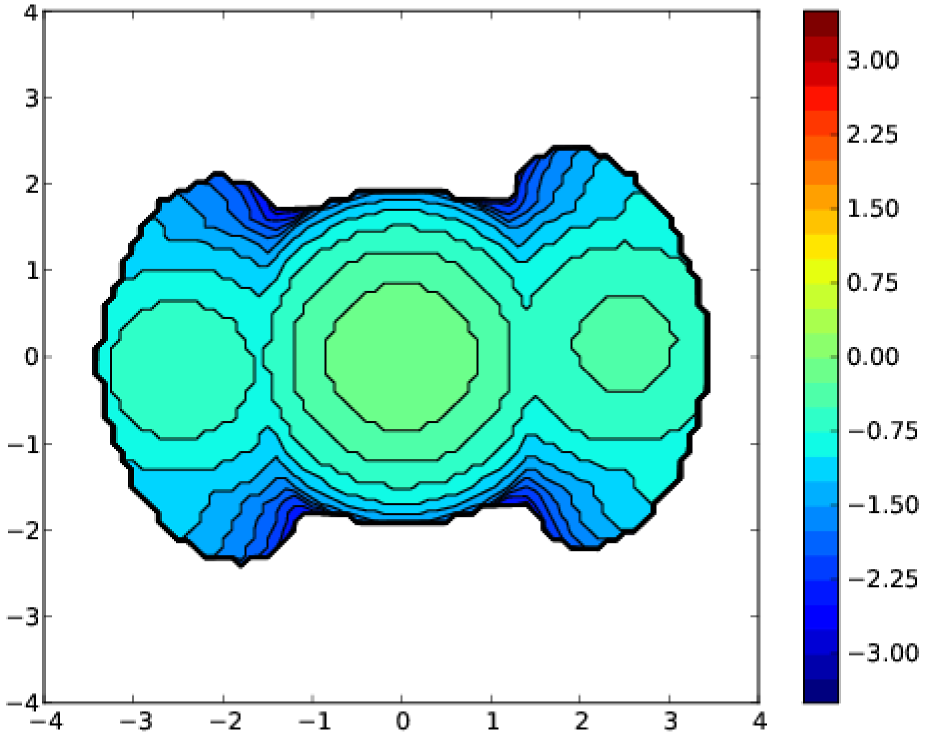

Figure S73. Steric Map of ^{i}Pr$_2$Im in [(^{i}Pr$_2$Im)Ni(CO)$_3$].

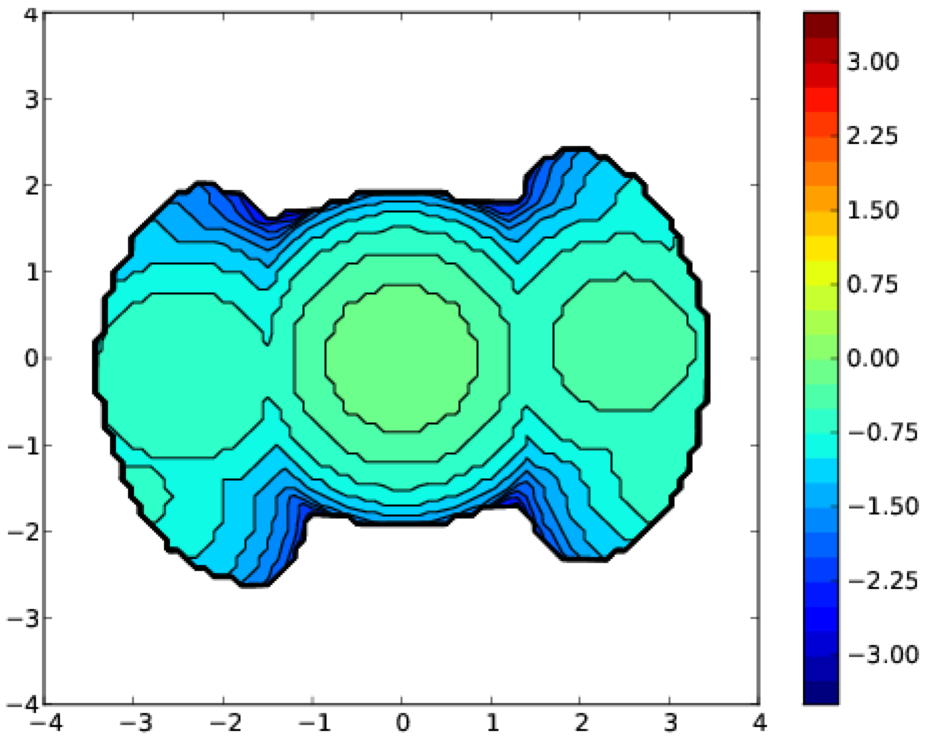

Figure S74. Steric Map of ${}^{i}Pr_2Im^{Me}$ in $[({}^{i}Pr_2Im^{Me})Ni(CO)_3]$.

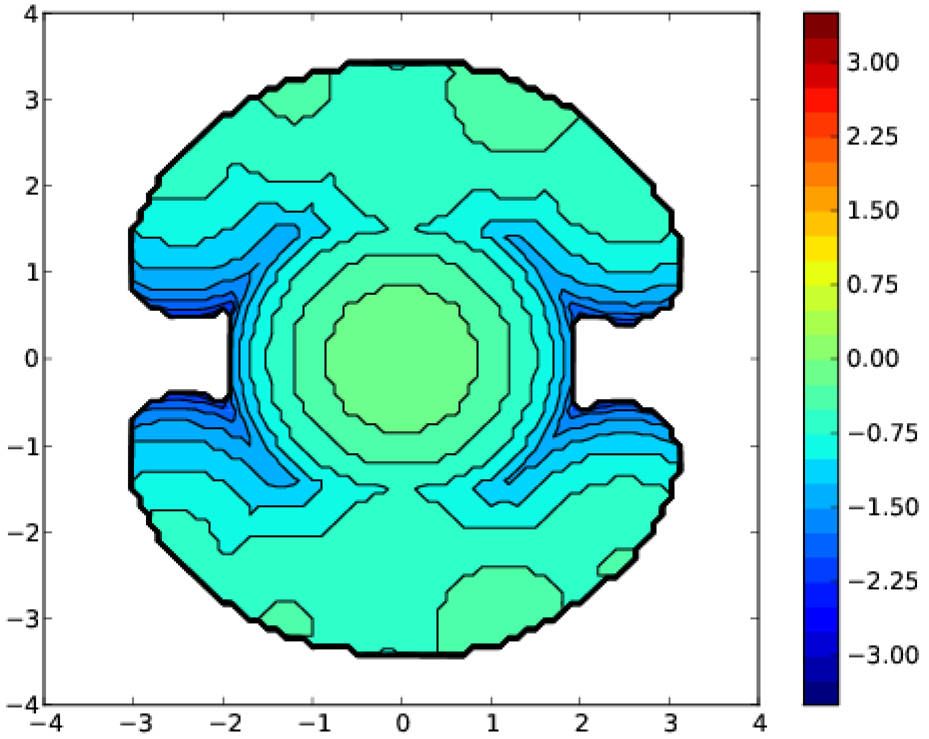

Figure S75. Steric Map of Mes_2Im in $[(Mes_2Im)Ni(CO)_3]$.

5) Cartesian Coordinates of the DFT optimized geometries

C_2Me_2

Energy = -155.8660188229

C	-1.2091819	-0.3774315	0.0000041
C	0.0152235	-0.3774287	-0.0000039
C	1.4757341	-0.3774220	0.0000038
H	1.8838920	0.0754130	-0.9285346
H	1.8838689	0.2003051	0.8564545
H	1.8839100	-1.4079785	0.0720703
C	-2.6696671	-0.3774380	-0.0000038
H	-3.0778050	-0.9549676	-0.8565792
H	-3.0778284	-0.8304796	0.9284259
H	-3.0778459	0.6531278	-0.0718371

$^{i}Pr_2Im^{Me}$

Energy = -540.3163717317

N	0.5273638	-2.8354825	-0.7555413
N	-0.9121912	-2.7256730	0.8370365
C	-0.1366930	-1.9260235	0.0321804
C	0.1775752	-4.1663021	-0.4632339
C	-0.7492186	-4.0956119	0.5620334
C	1.4535311	-2.3436869	-1.7957236
H	1.4501506	-1.2517783	-1.6011206
C	0.9090321	-2.5662356	-3.2149253
C	2.8903557	-2.8527813	-1.6104433
H	3.2349182	-2.6867986	-0.5703390
H	2.9963885	-3.9308350	-1.8457644
H	3.5727066	-2.3008139	-2.2882392
H	0.8877972	-3.6381488	-3.4995059
H	1.5490903	-2.0413199	-3.9530272

H	-0.1192176	-2.1632040	-3.3030274
C	-1.8153570	-2.0943542	1.8205471
H	-1.5471565	-1.0231997	1.7162911
C	-1.5194132	-2.5176487	3.2667212
C	-3.2949795	-2.2442779	1.4348355
H	-3.4600695	-1.9057487	0.3928695
H	-3.6537362	-3.2903728	1.5213929
H	-3.9255071	-1.6228051	2.1028241
H	-1.8167040	-3.5648544	3.4759483
H	-2.0836122	-1.8703449	3.9683851
H	-0.4404423	-2.4087203	3.4947316
C	-1.4274955	-5.2234484	1.2773094
H	-1.0574331	-5.3550975	2.3168062
H	-2.5268835	-5.0900010	1.3370363
H	-1.2434590	-6.1772908	0.7456735
C	0.7404281	-5.3887703	-1.1207556
H	0.6945184	-5.3362565	-2.2275938
H	1.8013907	-5.5723976	-0.8461666
H	0.1673223	-6.2849161	-0.8129151

Mes_2Im

Energy = -923.5446387791

C	-1.9035365	0.0673221	-0.1527120
C	-0.0266837	1.4224445	0.0349699
C	-1.1226280	2.2401033	0.0993040
H	1.0431576	1.6484739	0.0903732
H	-1.2036294	3.3265616	0.2078913
N	-2.2376021	1.3986357	-0.0153829
N	-0.5255967	0.1215693	-0.1172977
C	-3.5988242	1.8694274	0.0103536
C	-4.3226155	1.9519963	-1.2031771
C	-4.1774674	2.2411622	1.2463522
C	-5.6446144	2.4322780	-1.1529148

C	-5.5031251	2.7179052	1.2410706
C	-6.2547092	2.8224828	0.0556018
H	-6.2156110	2.5034914	-2.0934252
H	-5.9636003	3.0087711	2.1996571
C	0.3112950	-1.0456163	-0.2286183
C	0.4436828	-1.9077022	0.8851431
C	0.9758404	-1.3048283	-1.4511097
C	1.2774344	-3.0350417	0.7533653
C	1.7995054	-2.4440837	-1.5293263
C	1.9702379	-3.3192786	-0.4390322
H	1.3840334	-3.7150024	1.6145155
H	2.3143731	-2.6587846	-2.4803902
C	-3.4075255	2.1089911	2.5394146
H	-2.9613563	1.0977608	2.6334410
H	-2.5664848	2.8313928	2.6015452
H	-4.0650784	2.2836070	3.4125310
C	-3.6990984	1.5212579	-2.5082766
H	-4.3851149	1.7017848	-3.3579982
H	-2.7507767	2.0633183	-2.7065840
H	-3.4428607	0.4418258	-2.4775777
C	0.7824334	-0.4050087	-2.6491456
H	-0.2957544	-0.2373961	-2.8493292
H	1.2339229	0.5978843	-2.4962786
H	1.2418747	-0.8454799	-3.5547474
C	-0.3065373	-1.6401001	2.1671202
H	-0.0460753	-2.3830964	2.9451215
H	-0.0853961	-0.6279520	2.5660102
H	-1.4016249	-1.6749830	1.9899747
C	2.8860323	-4.5177813	-0.5428153
H	3.9417291	-4.2343594	-0.3389703
H	2.6129837	-5.3062705	0.1860629
H	2.8619172	-4.9641103	-1.5573152
C	-7.6877733	3.3039064	0.0797818

H	-7.9336866	3.9017115	-0.8209216
H	-8.3966544	2.4476899	0.1028484
H	-7.8968122	3.9255202	0.9727969

[Ni(^{i}Pr$_2$Im^{Me})$_2$(η^2-HC≡CPh)]

Energy = -2897.585540402

Ni	-0.0520379	0.0161692	1.0895410
C	1.3844752	-0.6615649	0.0096734
N	2.6941238	-0.2078944	-0.0109800
C	3.5068318	-1.0047046	-0.8337483
C	2.6923125	-1.9930375	-1.3502115
N	1.4082544	-1.7576308	-0.8324160
C	3.1137029	0.9239236	0.8399493
H	2.1486838	1.2422790	1.2937119
C	4.0290465	0.4829080	1.9915341
H	3.5782141	-0.3638227	2.5431043
H	4.1614480	1.3220410	2.7042622
H	5.0386637	0.1796612	1.6462783
C	3.6875650	2.1007961	0.0379742
H	4.6947035	1.8899800	-0.3742846
H	3.7831798	2.9871913	0.6972081
H	3.0211018	2.3725996	-0.8043368
C	0.1989009	-2.5818542	-1.0057997
H	-0.5714779	-1.9516201	-0.5097660
C	0.2887011	-3.9022133	-0.2263030
H	1.0603333	-4.5863147	-0.6356709
H	-0.6833184	-4.4347963	-0.2683216
H	0.5250545	-3.7026025	0.8374637
C	-0.2043129	-2.7633315	-2.4756375
H	-0.2330083	-1.7884517	-3.0016910
H	-1.2192491	-3.2070391	-2.5284106
H	0.4726968	-3.4419124	-3.0313389
C	-1.4672080	0.7003330	-0.0171663

N	-1.4586214	1.8174081	-0.8322005
C	-2.7162744	2.0508416	-1.4124966
C	-3.5455893	1.0426932	-0.9610825
N	-2.7683560	0.2346160	-0.1151422
C	-0.2380502	2.6358877	-0.9517722
H	0.5011452	2.0116811	-0.4024369
C	0.2462067	2.7823878	-2.4010476
H	0.3049885	1.7945983	-2.8996456
H	1.2616536	3.2281675	-2.4093492
H	-0.4021293	3.4441548	-3.0092907
C	-0.3580613	3.9749307	-0.2093273
H	-1.0946707	4.6591116	-0.6783995
H	0.6215306	4.4950142	-0.2076491
H	-0.6557043	3.8079024	0.8443699
C	-3.2028156	-0.9592063	0.6414594
H	-2.2635502	-1.2567623	1.1589267
C	-4.2426313	-0.6416239	1.7253918
H	-3.9110933	0.2040249	2.3568930
H	-4.3681667	-1.5248757	2.3837363
H	-5.2405004	-0.4036922	1.3032822
C	-3.6420787	-2.1121398	-0.2741195
H	-4.6220619	-1.9219968	-0.7564845
H	-3.7470783	-3.0408758	0.3224115
H	-2.8972834	-2.2994030	-1.0723432
C	0.5221298	-0.3505559	2.8469002
C	-0.5792810	0.3552825	2.9058844
C	-3.0419479	3.1652971	-2.3576326
H	-2.5761289	3.0310770	-3.3576134
H	-4.1362071	3.2203392	-2.5145722
H	-2.7168639	4.1544575	-1.9763429
C	-4.9816010	0.8047569	-1.3109522
H	-5.1262451	-0.1074432	-1.9282452
H	-5.6266409	0.6994347	-0.4154338

H	-5.3728128	1.6574537	-1.8980362
C	4.9570453	-0.7636403	-1.1170066
H	5.1256267	0.1401337	-1.7409655
H	5.5584837	-0.6397418	-0.1940526
H	5.3813872	-1.6228248	-1.6709330
C	3.0566552	-3.0886142	-2.3032908
H	2.6979023	-4.0817802	-1.9648786
H	2.6500738	-2.9238980	-3.3243199
H	4.1573615	-3.1552105	-2.3995681
H	1.3076446	-0.7978191	3.4708242
C	-1.5251140	0.9908888	3.8078540
C	-1.6663067	0.5498212	5.1535271
C	-2.3334718	2.0814922	3.3892440
C	-2.5669396	1.1690065	6.0302221
H	-1.0522097	-0.2974994	5.4962018
C	-3.2266936	2.7074184	4.2714745
H	-2.2371534	2.4287998	2.3492951
C	-3.3525064	2.2542383	5.5970555
H	-2.6580164	0.8040543	7.0660595
H	-3.8346176	3.5572218	3.9213787
H	-4.0575411	2.7419181	6.2881211

[Ni(^{i}Pr$_2$Im^{Me})$_2$(H)(C≡CPh)]

Energy = -2897.558160189

Ni	-0.1036986	0.4259365	-0.0823082
H	-0.0186297	-1.0479164	-0.0304834
C	1.7041380	0.0804564	-0.0233430
C	2.9197807	-0.1856551	0.0911651
C	4.3052114	-0.4963814	0.2164468
C	4.9981253	-0.3229645	1.4504075
C	5.0555231	-0.9950426	-0.8889035
C	6.3598995	-0.6301799	1.5671795
H	4.4392009	0.0578706	2.3185955

C	6.4179678	-1.2962758	-0.7639459
H	4.5402238	-1.1439290	-1.8497885
C	7.0830629	-1.1172804	0.4628948
H	6.8663888	-0.4866535	2.5353745
H	6.9699936	-1.6801070	-1.6371433
H	8.1534782	-1.3566822	0.5579616
C	0.1298300	2.3505158	-0.2626250
N	0.6420397	3.0306508	-1.3437354
N	-0.0066869	3.3098260	0.7121746
C	0.8337547	4.3902570	-1.0477351
C	1.0445453	2.3087341	-2.5732592
C	0.4282176	4.5671180	0.2610495
C	-0.4077698	2.9250113	2.0806931
C	1.3280789	5.4250952	-2.0097305
H	0.6974073	1.2730613	-2.3622441
C	0.4020976	5.8311069	1.0622166
H	-0.7455869	1.8766849	1.9323644
H	2.2695707	5.1240103	-2.5106241
H	0.5886206	5.6529174	-2.8068401
H	1.5302542	6.3721526	-1.4740215
H	-0.6282612	6.2095754	1.2341421
H	0.8787423	5.7117107	2.0561136
H	0.9563614	6.6267937	0.5289079
C	-1.9785691	0.1222704	0.1106224
N	-3.0093487	0.3278195	-0.7791584
N	-2.5791014	-0.4959800	1.1883880
C	-4.2262574	-0.1681134	-0.2810962
C	-2.7437980	0.9026674	-2.1122536
C	-3.9516597	-0.6875460	0.9692247
C	-1.7640866	-0.9927779	2.3203655
C	-5.5467816	-0.0880400	-0.9813565
H	-1.6864155	1.2271675	-2.0135640
C	-4.9080569	-1.2904695	1.9503992

H	-0.7610615	-0.5693251	2.0899349
H	-5.9542864	0.9451523	-1.0131585
H	-5.4952913	-0.4529797	-2.0268251
H	-6.2913514	-0.7144011	-0.4539305
H	-4.5514201	-2.2607054	2.3501567
H	-5.1001965	-0.6266974	2.8200532
H	-5.8834111	-1.4762556	1.4615550
C	-1.6205124	-2.5202928	2.2896806
H	-0.8991516	-2.8434405	3.0669450
H	-2.5788622	-3.0441266	2.4834210
H	-1.2284774	-2.8467996	1.3067197
C	-2.2206576	-0.4477836	3.6801949
H	-3.1660929	-0.9046648	4.0340540
H	-1.4463138	-0.6690333	4.4419213
H	-2.3597019	0.6512752	3.6463773
C	-3.5944076	2.1444569	-2.4122470
H	-3.2093079	2.6433482	-3.3245158
H	-4.6592333	1.9040631	-2.6007674
H	-3.5404909	2.8728839	-1.5791231
C	-2.8005192	-0.1623996	-3.2176039
H	-2.1384429	-1.0145232	-2.9679173
H	-3.8265021	-0.5514669	-3.3801556
H	-2.4548475	0.2714623	-4.1777767
C	0.3261429	2.8226510	-3.8291937
H	-0.7672417	2.9040517	-3.6669425
H	0.6987060	3.8125240	-4.1598102
H	0.4958670	2.1152154	-4.6656520
C	2.5692656	2.2424097	-2.7309838
H	3.0217501	3.2349307	-2.9314254
H	3.0251418	1.8071605	-1.8207172
H	2.8218923	1.5840564	-3.5865812
C	0.7978214	2.8883402	3.0311738
H	1.2225249	3.8966344	3.2142120

H	0.4931386	2.4725573	4.0131493
H	1.5924158	2.2404476	2.6115881
C	-1.5954424	3.7363984	2.6155220
H	-2.4255948	3.7596075	1.8815610
H	-1.9756255	3.2626892	3.5431629
H	-1.3264291	4.7797646	2.8731171

[Ni(^{i}Pr$_2$Im^{Me})$_2$(=C=C{H}Ph)]

Energy = -2897.567035525

Ni	-0.2511538	-0.2050885	0.9856177
C	1.2916571	-0.7639897	-0.0121370
N	2.5851285	-0.3054231	0.1507604
C	3.4986684	-1.0683783	-0.5945041
C	2.7600273	-2.0347021	-1.2506660
N	1.4188023	-1.8204081	-0.8912551
C	2.8709640	0.7866068	1.1059331
H	1.8481323	1.0763022	1.4386943
C	3.6206499	0.2955861	2.3515542
H	3.0771660	-0.5424198	2.8279017
H	3.6843386	1.1162385	3.0941599
H	4.6567974	-0.0303599	2.1272230
C	3.5345814	2.0014248	0.4419688
H	4.5908856	1.8120638	0.1640824
H	3.5310039	2.8574123	1.1465150
H	2.9886757	2.3082484	-0.4726755
C	0.2349371	-2.6235681	-1.2509461
H	-0.5917364	-2.0080070	-0.8326651
C	0.2181203	-3.9773701	-0.5257136
H	1.0293803	-4.6511869	-0.8700498
H	-0.7448554	-4.4954585	-0.7105469
H	0.3240616	-3.8293147	0.5667660
C	0.0178921	-2.7371429	-2.7663696
H	0.0689942	-1.7414697	-3.2501138

H	-0.9886274	-3.1587899	-2.9638853
H	0.7516252	-3.4049135	-3.2598242
C	-1.5672332	0.6067848	-0.1564810
N	-1.5163285	1.7771531	-0.8872207
C	-2.7681188	2.0981036	-1.4380898
C	-3.6302795	1.0861041	-1.0608980
N	-2.8788727	0.1895647	-0.2855809
C	-0.2732879	2.5689641	-0.9280210
H	0.4576826	1.8657495	-0.4735322
C	0.2008071	2.8812254	-2.3543958
H	0.2119801	1.9652250	-2.9779304
H	1.2350207	3.2798299	-2.3193212
H	-0.4229087	3.6434507	-2.8618091
C	-0.3538357	3.8082212	-0.0240059
H	-1.0768625	4.5601699	-0.4016926
H	0.6377382	4.3016546	0.0348055
H	-0.6549408	3.5170583	1.0014208
C	-3.3619132	-0.9895137	0.4647538
H	-2.4209334	-1.3792232	0.9154122
C	-4.2742480	-0.5908809	1.6324922
H	-3.7679500	0.1594576	2.2704829
H	-4.4883357	-1.4799853	2.2591153
H	-5.2468883	-0.1758599	1.2973315
C	-3.9600033	-2.0762414	-0.4383727
H	-4.9478860	-1.7955006	-0.8558037
H	-4.1056446	-3.0054281	0.1487023
H	-3.2826604	-2.3106681	-1.2839409
C	-3.0581346	3.2929687	-2.2918822
H	-2.6044214	3.2199729	-3.3036486
H	-4.1507202	3.4003805	-2.4328751
H	-2.6934533	4.2361479	-1.8369082
C	-5.0697834	0.9112818	-1.4328817
H	-5.2249275	0.0781933	-2.1514435

H	-5.7155038	0.7101774	-0.5546138
H	-5.4502108	1.8315443	-1.9159224
C	4.9716430	-0.8150038	-0.6776925
H	5.2127365	0.1172934	-1.2319182
H	5.4469881	-0.7344085	0.3204645
H	5.4689845	-1.6472329	-1.2114976
C	3.2442673	-3.0920823	-2.1930515
H	2.8536862	-4.0977177	-1.9366041
H	2.9619879	-2.8897268	-3.2486279
H	4.3489040	-3.1528257	-2.1581100
C	-0.4769948	-0.4461872	2.6888687
C	-0.7007849	-0.6391110	4.0062630
H	-1.6163630	-0.1760821	4.4327086
C	0.1287178	-1.3849882	4.9642069
C	-0.1604053	-1.3388834	6.3528778
C	1.2293979	-2.1841148	4.5504157
C	0.6176738	-2.0427985	7.2841390
H	-1.0129245	-0.7317831	6.6990919
C	2.0059296	-2.8840934	5.4819347
H	1.4482464	-2.2538903	3.4734199
C	1.7093708	-2.8188885	6.8575260
H	0.3683597	-1.9852303	8.3559505
H	2.8516171	-3.4981761	5.1320493
H	2.3193029	-3.3736430	7.5872159

Isomer of [Ni($^{i}Pr_2Im^{Me}$)$_2$(η^2-HC≡CPh)]

Energy = -2897.626501547

Ni	-0.0949297	0.4570717	0.9090477
C	1.3348920	-0.4369862	0.0346901
N	2.6749078	-0.3337533	0.3684259
C	3.4534123	-1.2860017	-0.3090925
C	2.5908484	-2.0022924	-1.1139189
N	1.3134343	-1.4626829	-0.8986118

C	3.2418593	0.6055422	1.3601852
H	3.9103394	-0.0124376	1.9998040
C	4.0878931	1.6959956	0.6810976
H	4.9396010	1.2880414	0.1030958
H	4.4996041	2.3826584	1.4486068
H	3.4559305	2.2918646	-0.0086600
C	2.1808574	1.2618872	2.2812573
H	1.8699913	2.2310521	1.8343296
H	2.7262546	1.5286178	3.2200554
C	0.0340780	-1.9430886	-1.4514937
H	-0.6756699	-1.1760707	-1.0717296
C	-0.3740590	-3.2987808	-0.8552079
H	0.2999895	-4.1225924	-1.1673479
H	-1.3977509	-3.5675170	-1.1856109
H	-0.3728368	-3.2491763	0.2516025
C	-0.0098574	-1.9135142	-2.9858716
H	0.3240860	-0.9296294	-3.3713795
H	-1.0526083	-2.0735654	-3.3278249
H	0.6113762	-2.7019010	-3.4558342
C	-1.5250967	1.1133045	-0.1858286
N	-1.4427530	2.2108875	-1.0286615
C	-2.6711133	2.4872860	-1.6482752
C	-3.5593588	1.5299447	-1.1973206
N	-2.8432678	0.7075655	-0.3113292
C	-0.1804325	2.9656443	-1.1255736
H	0.5148965	2.3068423	-0.5530517
C	0.3520632	3.0751592	-2.5608773
H	0.3707429	2.0815003	-3.0510517
H	1.3908179	3.4629392	-2.5419513
H	-0.2421032	3.7657702	-3.1921385
C	-0.2544435	4.3162650	-0.3981373
H	-0.9340347	5.0339391	-0.9024129
H	0.7508585	4.7834835	-0.3602182

H	-0.6079838	4.1763090	0.6423153
C	-3.3590924	-0.4140097	0.4998970
H	-2.4427075	-0.7637295	1.0246861
C	-4.3554880	0.0435305	1.5728712
H	-3.9328763	0.8719557	2.1716585
H	-4.5602729	-0.7926214	2.2705792
H	-5.3252729	0.3671938	1.1421535
C	-3.8997549	-1.5696554	-0.3557543
H	-4.8706834	-1.3269197	-0.8324851
H	-4.0640315	-2.4597694	0.2843126
H	-3.1846598	-1.8498194	-1.1541654
C	0.9405352	0.4400406	2.5569161
C	-0.3107751	1.0904528	2.8459775
C	-2.9177837	3.5941651	-2.6255549
H	-2.4311107	3.4167463	-3.6088313
H	-4.0034187	3.6974942	-2.8149174
H	-2.5558943	4.5747923	-2.2550647
C	-4.9930193	1.3452487	-1.5867090
H	-5.1527456	0.4424784	-2.2146194
H	-5.6647131	1.2553201	-0.7092732
H	-5.3392079	2.2152697	-2.1769704
C	4.9263742	-1.4663477	-0.1201855
H	5.5304303	-0.6559097	-0.5819670
H	5.2059317	-1.5077123	0.9535858
H	5.2556113	-2.4179767	-0.5793811
C	2.9213117	-3.1150560	-2.0592488
H	2.2682967	-4.0004596	-1.9222402
H	2.8393601	-2.8095626	-3.1244434
H	3.9642817	-3.4493229	-1.8983456
H	1.1298818	-0.5518281	3.0120201
C	-1.3636744	0.5053798	3.6952529
C	-1.4884059	-0.8927866	3.9306063
C	-2.3031914	1.3479356	4.3508170

C	-2.4839908	-1.4083787	4.7724787
H	-0.7874225	-1.5842969	3.4372910
C	-3.3000326	0.8321078	5.1918502
H	-2.2301784	2.4375549	4.1987040
C	-3.4009867	-0.5531554	5.4131863
H	-2.5449941	-2.4971383	4.9340500
H	-4.0028637	1.5213678	5.6875929
H	-4.1791307	-0.9607572	6.0767538
H	-0.3152710	2.1969982	2.8457137

[Ni(^{i}Pr$_2$Im^{Me})$_2$]

Energy = -2589.354277139

Ni	0.0000000	0.0000000	0.0000000
N	-0.7705439	-0.7705439	2.7226096
N	0.7705439	0.7705439	2.7226096
C	0.0000000	0.0000000	1.8664210
C	-0.4895643	-0.4895643	4.0670905
C	0.4895643	0.4895643	4.0670905
N	-0.7705439	0.7705439	-2.7226096
N	0.7705439	-0.7705439	-2.7226096
C	0.0000000	0.0000000	-1.8664210
C	-0.4895643	0.4895643	-4.0670905
C	0.4895643	-0.4895643	-4.0670905
C	1.7334339	1.7334339	2.1556321
H	0.3195377	3.3986336	2.2122523
C	3.1906883	1.3753860	2.4768445
C	1.3753860	3.1906883	2.4768445
H	1.5256021	3.4457413	3.5460582
H	3.4457413	1.5256021	3.5460582
H	2.0171304	3.8733097	1.8830012
H	3.3986336	0.3195377	2.2122523
H	3.8733097	2.0171304	1.8830012
H	1.5761422	1.5761422	1.0592348

C	-1.7334339	-1.7334339	2.1556321
H	-1.5761422	-1.5761422	1.0592348
C	-3.1906883	-1.3753860	2.4768445
C	-1.3753860	-3.1906883	2.4768445
H	-0.3195377	-3.3986336	2.2122523
H	-1.5256021	-3.4457413	3.5460582
H	-2.0171304	-3.8733097	1.8830012
H	-3.4457413	-1.5256021	3.5460582
H	-3.8733097	-2.0171304	1.8830012
H	-3.3986336	-0.3195377	2.2122523
C	-1.7334339	1.7334339	-2.1556321
H	-1.5761422	1.5761422	-1.0592348
C	-3.1906883	1.3753860	-2.4768445
C	-1.3753860	3.1906883	-2.4768445
H	-0.3195377	3.3986336	-2.2122523
H	-1.5256021	3.4457413	-3.5460582
H	-2.0171304	3.8733097	-1.8830012
H	-3.4457413	1.5256021	-3.5460582
H	-3.8733097	2.0171304	-1.8830012
H	-3.3986336	0.3195377	-2.2122523
C	1.7334339	-1.7334339	-2.1556321
H	1.5761422	-1.5761422	-1.0592348
C	3.1906883	-1.3753860	-2.4768445
C	1.3753860	-3.1906883	-2.4768445
H	0.3195377	-3.3986336	-2.2122523
H	1.5256021	-3.4457413	-3.5460582
H	2.0171304	-3.8733097	-1.8830012
H	3.4457413	-1.5256021	-3.5460582
H	3.8733097	-2.0171304	-1.8830012
H	3.3986336	-0.3195377	-2.2122523
C	1.1460222	1.1460222	5.2410967
H	2.2439106	0.9811770	5.2635432
H	0.9811770	2.2439106	5.2635432

H	0.7361751	0.7361751	6.1837835
C	-1.1460222	-1.1460222	5.2410967
H	-2.2439106	-0.9811770	5.2635432
H	-0.9811770	-2.2439106	5.2635432
H	-0.7361751	-0.7361751	6.1837835
C	1.1460222	-1.1460222	-5.2410967
H	2.2439106	-0.9811770	-5.2635432
H	0.9811770	-2.2439106	-5.2635432
H	0.7361751	-0.7361751	-6.1837835
C	-1.1460222	1.1460222	-5.2410967
H	-2.2439106	0.9811770	-5.2635432
H	-0.9811770	2.2439106	-5.2635432
H	-0.7361751	0.7361751	-6.1837835

[Ni($^{i}Pr_2Im^{Me}$)$_2$(η^2-MeC≡CMe)]

Energy = -2745.252850637

Ni	-0.0002134	0.0005244	1.0568118
C	1.4478625	-0.6952557	0.0039917
N	2.7588754	-0.2382627	-0.0427265
C	3.5749933	-1.0719419	-0.8256081
C	2.7660570	-2.0917192	-1.2862142
N	1.4818181	-1.8379257	-0.7781712
C	3.1525810	0.9913232	0.6719874
H	2.2042950	1.2679429	1.1865558
C	4.2141005	0.7495028	1.7550399
H	3.9305975	-0.1005971	2.4034405
H	4.2992717	1.6507219	2.3956393
H	5.2206332	0.5527497	1.3333033
C	3.5414081	2.1316379	-0.2817371
H	4.5189874	1.9581066	-0.7760525
H	3.6248423	3.0819512	0.2838767
H	2.7779375	2.2677500	-1.0720671
C	0.2746452	-2.6700884	-0.9152185

H	-0.4932748	-2.0199835	-0.4388555
C	0.3655520	-3.9606087	-0.0875370
H	1.1345653	-4.6615607	-0.4727395
H	-0.6073608	-4.4932180	-0.1063857
H	0.6048021	-3.7213059	0.9672631
C	-0.1337399	-2.9064895	-2.3758156
H	-0.1726239	-1.9503426	-2.9347857
H	-1.1446339	-3.3614489	-2.4095128
H	0.5475011	-3.5976008	-2.9109929
C	-1.4477971	0.6951255	0.0025850
N	-1.4817790	1.8377901	-0.7795711
C	-2.7656477	2.0905702	-1.2890538
C	-3.5742948	1.0701405	-0.8293758
N	-2.7583812	0.2370778	-0.0456095
C	-0.2751377	2.6709895	-0.9150265
H	0.4927249	2.0214925	-0.4377065
C	0.1349236	2.9078603	-2.3750766
H	0.1755178	1.9517751	-2.9340334
H	1.1453913	3.3638611	-2.4074320
H	-0.5463299	3.5982872	-2.9111200
C	-0.3681736	3.9613495	-0.0873339
H	-1.1373063	4.6616845	-0.4734205
H	0.6043084	4.4947888	-0.1049112
H	-0.6085266	3.7217258	0.9671388
C	-3.1521567	-0.9920804	0.6698067
H	-2.2036789	-1.2688535	1.1839494
C	-4.2127849	-0.7491945	1.7535009
H	-3.9280878	0.1007500	2.4015640
H	-4.2983979	-1.6502424	2.3942843
H	-5.2193932	-0.5515536	1.3323388
C	-3.5422091	-2.1326797	-0.2830603
H	-4.5202751	-1.9591721	-0.7764025
H	-3.6252727	-3.0827193	0.2830661

H	-2.7795928	-2.2693427	-1.0741316
C	0.5464504	-0.3532596	2.8525359
C	-0.5474126	0.3561495	2.8520379
C	-3.1368020	3.2392460	-2.1748809
H	-2.7023983	3.1544467	-3.1941123
H	-4.2362594	3.2868286	-2.2928715
H	-2.8115037	4.2162147	-1.7625060
C	-5.0325393	0.8592199	-1.0948803
H	-5.2423978	-0.1122406	-1.5894074
H	-5.6463488	0.8941773	-0.1705648
H	-5.4112781	1.6519967	-1.7676116
C	5.0339342	-0.8627068	-1.0886391
H	5.2461773	0.1103379	-1.5789567
H	5.6466127	-0.9025602	-0.1637426
H	5.4119568	-1.6534671	-1.7641393
C	3.1373745	-3.2408149	-2.1714322
H	2.8113258	-4.2175316	-1.7590401
H	2.7037902	-3.1561109	-3.1910162
H	4.2368986	-3.2889341	-2.2885431
C	1.5566997	-1.0245062	3.7197007
H	2.1934728	-0.2848850	4.2568338
H	1.0698761	-1.6435306	4.5072553
H	2.2389931	-1.6924963	3.1534956
C	-1.5578117	1.0282778	3.7183542
H	-2.1949248	0.2891976	4.2558270
H	-1.0711266	1.6477791	4.5056218
H	-2.2397815	1.6959806	3.1514232

[Ni(^{i}Pr$_2$Im^{Me})$_2$(η^2-MeC≡CMe)] constrained geometry

Energy = -2745.251784879

Ni	-0.0004252	0.0000742	1.0356053
C	1.5006990	-0.7737289	0.1308181
N	2.8055587	-0.3023006	0.1016359

C	3.6837291	-1.2300682	-0.4836851
C	2.9205254	-2.3278266	-0.8283523
N	1.6005393	-2.0255001	-0.4543108
C	3.1158164	1.0561244	0.5833724
H	2.1451257	1.3611128	1.0371794
C	4.1748613	1.0883327	1.6942912
H	3.9366286	0.3552633	2.4885129
H	4.1910379	2.0957281	2.1577207
H	5.1985016	0.8847118	1.3207064
C	3.4352735	2.0184865	-0.5711158
H	4.4102343	1.7948433	-1.0511496
H	3.4860960	3.0606638	-0.1945657
H	2.6499514	1.9695231	-1.3504198
C	0.4071731	-2.8817133	-0.5605525
H	-0.3943619	-2.1776459	-0.2408443
C	0.4328855	-4.0407448	0.4464750
H	1.2288464	-4.7811287	0.2241963
H	-0.5356229	-4.5814318	0.4285445
H	0.5900828	-3.6522845	1.4718108
C	0.1083247	-3.3267290	-1.9988498
H	0.1187762	-2.4603669	-2.6899608
H	-0.9000461	-3.7858192	-2.0427243
H	0.8250571	-4.0833639	-2.3761974
C	-1.5007916	0.7740040	0.1296655
N	-1.6000162	2.0255104	-0.4561358
C	-2.9197202	2.3279469	-0.8310915
C	-3.6833580	1.2305113	-0.4863634
N	-2.8057439	0.3028492	0.0999434
C	-0.4064281	2.8814449	-0.5620695
H	0.3948037	2.1773471	-0.2416928
C	-0.1067488	3.3258868	-2.0003723
H	-0.1170341	2.4592843	-2.6911839
H	0.9017441	3.7847423	-2.0438896

H	-0.8231160	4.0825497	-2.3783553
C	-0.4324573	4.0408546	0.4445173
H	-1.2281881	4.7812805	0.2215549
H	0.5361460	4.5813823	0.4268818
H	-0.5902466	3.6527998	1.4699166
C	-3.1165230	-1.0554182	0.5817946
H	-2.1462973	-1.3603066	1.0366641
C	-4.1767216	-1.0873043	1.6916210
H	-3.9394011	-0.3539160	2.4858235
H	-4.1932623	-2.0945265	2.1554130
H	-5.2000009	-0.8839490	1.3169073
C	-3.4348048	-2.0180870	-0.5727643
H	-4.4091780	-1.7944219	-1.0539812
H	-3.4862125	-3.0601465	-0.1959672
H	-2.6485748	-1.9694607	-1.3511723
C	0.5533993	-0.3399201	2.8399994
C	-0.5554780	0.3400763	2.8396179
C	-3.3655310	3.5982134	-1.4863065
H	-2.9844332	3.7033920	-2.5246610
H	-4.4705256	3.6290525	-1.5403019
H	-3.0422742	4.5009019	-0.9281037
C	-5.1580160	1.0384944	-0.6597768
H	-5.4068062	0.0995363	-1.1960179
H	-5.7036336	1.0157482	0.3076141
H	-5.5805511	1.8714877	-1.2533715
C	5.1584440	-1.0378152	-0.6563428
H	5.4073389	-0.0992468	-1.1932225
H	5.7034598	-1.0141223	0.3113622
H	5.5815575	-1.8712030	-1.2489704
C	3.3669693	-3.5982840	-1.4827648
H	3.0435768	-4.5007960	-0.9243574
H	2.9864787	-3.7039849	-2.5212889
H	4.4720004	-3.6289226	-1.5361271

C	1.5948852	-0.9923156	3.6819005
H	2.1254558	-0.2541113	4.3258649
H	1.1460198	-1.7404000	4.3741162
H	2.3654963	-1.5219561	3.0840450
C	-1.5974965	0.9925550	3.6807935
H	-2.1286494	0.2543780	4.3243069
H	-1.1490393	1.7405295	4.3733917
H	-2.3675843	1.5223437	3.0823964

[Ni($^{i}Pr_2Im^{Me}$)(η^2-MeC≡CMe)]

Energy = -2204.886311068

Ni	0.1164710	0.0595564	1.0624828
C	-0.0152947	0.0402405	-0.8255266
N	0.1136498	1.1047652	-1.6948299
C	-0.0680441	0.7024758	-3.0276827
C	-0.3128622	-0.6583530	-2.9967953
N	-0.2721669	-1.0396898	-1.6461351
C	0.3544423	2.4600850	-1.1612943
H	0.4899024	2.2570028	-0.0731195
C	1.6532135	3.0879497	-1.6846220
H	2.5045868	2.3894273	-1.5623506
H	1.8804488	4.0069725	-1.1073570
H	1.5883385	3.3794971	-2.7521699
C	-0.8723954	3.3711509	-1.3106693
H	-1.0828851	3.6310798	-2.3683247
H	-0.7054028	4.3212669	-0.7636443
H	-1.7711807	2.8832450	-0.8846792
C	-0.5148777	-2.3689876	-1.0512549
H	-0.3081882	-2.1779689	0.0279152
C	-1.9837553	-2.8003923	-1.1697706
H	-2.6522804	-2.0114928	-0.7721381
H	-2.1540898	-3.7242922	-0.5805330
H	-2.2805708	-3.0164126	-2.2165656

C	0.4789413	-3.4330801	-1.5360523
H	0.3074740	-3.7307987	-2.5899509
H	0.3759865	-4.3469740	-0.9166923
H	1.5214996	-3.0707968	-1.4383942
C	0.1305861	-0.5502492	2.8683021
C	0.3580905	0.7088302	2.8379519
C	0.0346062	1.5997458	-4.2211253
H	1.0758829	1.9356740	-4.4146369
H	-0.3077678	1.0651438	-5.1276867
H	-0.5898384	2.5108961	-4.1224595
C	-0.5385198	-1.5866911	-4.1488939
H	0.3184173	-2.2733660	-4.3184497
H	-1.4412700	-2.2170330	-4.0161600
H	-0.6788867	-1.0065834	-5.0808666
C	-0.0622349	-1.8676594	3.5188170
H	0.7063626	-2.5995731	3.1913214
H	-0.0009878	-1.7877707	4.6266427
H	-1.0506015	-2.3071292	3.2669265
C	0.6432139	2.0394269	3.4245881
H	-0.1364607	2.7817267	3.1515858
H	0.6911144	1.9932938	4.5349926
H	1.6118335	2.4449416	3.0626046

[Ni($^{i}Pr_2Im^{Me}$)(η^2-MeC≡CMe)$_2$]

Energy = -2360.786634310

Ni	-0.0032425	0.2787289	0.0093148
C	-0.9883666	0.3647449	-1.6763814
C	-0.7405448	1.5597381	-1.2807924
C	0.9766767	0.4858125	1.6874662
C	0.6809546	1.6492983	1.2349530
C	-0.8587351	3.0168345	-1.5264103
H	0.1352496	3.5077956	-1.5852404
H	-1.3932574	3.2258453	-2.4792028

H	-1.4131414	3.5296828	-0.7127155
C	-1.5967355	-0.5531147	-2.6683207
H	-0.8746325	-1.3151282	-3.0318193
H	-2.4464865	-1.1219483	-2.2325130
H	-1.9849293	-0.0041137	-3.5543748
C	0.7402105	3.1201587	1.4093589
H	-0.2727181	3.5730546	1.4449426
H	1.2648235	3.3965742	2.3504215
H	1.2746271	3.6145655	0.5712417
C	1.6212828	-0.3574982	2.7218318
H	0.9343772	-1.1372344	3.1145915
H	2.5008947	-0.9024671	2.3155884
H	1.9763854	0.2477535	3.5848283
C	0.0345574	-1.6309610	0.0545648
N	-0.9285856	-2.4809039	0.5473635
N	1.0304425	-2.4644644	-0.3994963
C	-0.5435635	-3.8256499	0.4084412
C	-2.1560751	-1.9333884	1.1664047
C	0.6978663	-3.8154059	-0.1988521
C	2.2367120	-1.8986596	-1.0433313
C	-1.3597041	-5.0058142	0.8349912
H	-2.0570081	-0.8471362	0.9411230
C	1.5595069	-4.9812671	-0.5720133
H	2.0951419	-0.8078751	-0.8684073
H	-1.6810110	-4.9384389	1.8944486
H	-2.2764550	-5.1355390	0.2220048
H	-0.7648699	-5.9333899	0.7343274
H	1.8778861	-4.9504423	-1.6340316
H	2.4807740	-5.0465777	0.0445135
H	1.0014078	-5.9256647	-0.4273604
C	2.2499121	-2.1314285	-2.5606333
H	3.0980279	-1.5812502	-3.0159406
H	2.3678880	-3.2021592	-2.8260107

H	1.3165930	-1.7555028	-3.0222591
C	3.5434150	-2.3279525	-0.3621524
H	3.8187843	-3.3792001	-0.5803026
H	4.3732118	-1.6901391	-0.7274681
H	3.4814606	-2.2030719	0.7368109
C	-3.4452106	-2.4441474	0.5082050
H	-3.6793715	-3.4942610	0.7746551
H	-4.2991474	-1.8230965	0.8456857
H	-3.3885379	-2.3673482	-0.5954457
C	-2.1601184	-2.0960759	2.6928500
H	-1.2420083	-1.6632292	3.1347353
H	-3.0288644	-1.5586438	3.1238130
H	-2.2362494	-3.1570983	3.0078989

[Ni(Mes$_2$Im)$_2$]

Energy = -3355.808652075

Ni	0.0000000	0.0000000	0.0000000
C	0.0000000	0.0000000	-1.8520874
C	0.0000000	0.0000000	1.8520874
N	0.7673534	-0.7673534	-2.7235953
C	0.4839117	-0.4839117	-4.0624770
H	0.9927534	-0.9927534	-4.8868021
C	-0.4839117	0.4839117	-4.0624770
H	-0.9927534	0.9927534	-4.8868021
N	-0.7673534	0.7673534	-2.7235953
N	0.7673534	0.7673534	2.7235953
C	0.4839117	0.4839117	4.0624770
H	0.9927534	0.9927534	4.8868021
C	-0.4839117	-0.4839117	4.0624770
H	-0.9927534	-0.9927534	4.8868021
N	-0.7673534	-0.7673534	2.7235953
C	-1.7403192	-1.7403192	2.3036197
C	-1.3366450	-3.0854482	2.1248361

C	-3.0854482	-1.3366450	2.1248361
C	-2.3184112	-4.0276533	1.7636008
C	-4.0276533	-2.3184112	1.7636008
C	-3.6688107	-3.6688107	1.5825518
H	-2.0139823	-5.0770741	1.6158783
H	-5.0770741	-2.0139823	1.6158783
C	1.7403192	1.7403192	2.3036197
C	3.0854482	1.3366450	2.1248361
C	1.3366450	3.0854482	2.1248361
C	4.0276533	2.3184112	1.7636008
C	2.3184112	4.0276533	1.7636008
C	3.6688107	3.6688107	1.5825518
H	5.0770741	2.0139823	1.6158783
H	2.0139823	5.0770741	1.6158783
C	-1.7403192	1.7403192	-2.3036197
C	-3.0854482	1.3366450	-2.1248361
C	-1.3366450	3.0854482	-2.1248361
C	-4.0276533	2.3184112	-1.7636008
C	-2.3184112	4.0276533	-1.7636008
C	-3.6688107	3.6688107	-1.5825518
H	-5.0770741	2.0139823	-1.6158783
H	-2.0139823	5.0770741	-1.6158783
C	1.7403192	-1.7403192	-2.3036197
C	3.0854482	-1.3366450	-2.1248361
C	1.3366450	-3.0854482	-2.1248361
C	4.0276533	-2.3184112	-1.7636008
C	2.3184112	-4.0276533	-1.7636008
C	3.6688107	-3.6688107	-1.5825518
H	5.0770741	-2.0139823	-1.6158783
H	2.0139823	-5.0770741	-1.6158783
C	-3.4819132	-0.1108701	-2.2790102
H	-4.5715040	-0.2452313	-2.1380381
H	-3.2103977	-0.5105963	-3.2781342

H	-2.9494258	-0.7379151	-1.5314409
C	0.1108701	3.4819132	-2.2790102
H	0.5105963	3.2103977	-3.2781342
H	0.2452313	4.5715040	-2.1380381
H	0.7379151	2.9494258	-1.5314409
C	-4.7092132	4.7092132	-1.2340332
H	-4.2742924	5.5429301	-0.6473291
H	-5.1546127	5.1546127	-2.1506130
H	-5.5429301	4.2742924	-0.6473291
C	-0.1108701	-3.4819132	-2.2790102
H	-0.7379151	-2.9494258	-1.5314409
H	-0.5105963	-3.2103977	-3.2781342
H	-0.2452313	-4.5715040	-2.1380381
C	4.7092132	-4.7092132	-1.2340332
H	5.1546127	-5.1546127	-2.1506130
H	5.5429301	-4.2742924	-0.6473291
H	4.2742924	-5.5429301	-0.6473291
C	3.4819132	0.1108701	-2.2790102
H	3.2103977	0.5105963	-3.2781342
H	2.9494258	0.7379151	-1.5314409
H	4.5715040	0.2452313	-2.1380381
C	0.1108701	-3.4819132	2.2790102
H	0.5105963	-3.2103977	3.2781342
H	0.2452313	-4.5715040	2.1380381
H	0.7379151	-2.9494258	1.5314409
C	-3.4819132	0.1108701	2.2790102
H	-4.5715040	0.2452313	2.1380381
H	-3.2103977	0.5105963	3.2781342
H	-2.9494258	0.7379151	1.5314409
C	-4.7092132	-4.7092132	1.2340332
H	-5.1546127	-5.1546127	2.1506130
H	-5.5429301	-4.2742924	0.6473291
H	-4.2742924	-5.5429301	0.6473291

C	-0.1108701	3.4819132	2.2790102
H	-0.5105963	3.2103977	3.2781342
H	-0.2452313	4.5715040	2.1380381
H	-0.7379151	2.9494258	1.5314409
C	3.4819132	-0.1108701	2.2790102
H	4.5715040	-0.2452313	2.1380381
H	3.2103977	-0.5105963	3.2781342
H	2.9494258	-0.7379151	1.5314409
C	4.7092132	4.7092132	1.2340332
H	4.2742924	5.5429301	0.6473291
H	5.1546127	5.1546127	2.1506130
H	5.5429301	4.2742924	0.6473291

[Ni(Mes$_2$Im)$_2$(η^2-MeC≡CMe)]

Energy = -3511.681819271

Ni	0.0633710	0.3910998	0.9312298
C	0.7206179	0.5102435	2.7415413
C	-0.3385365	1.2479541	2.6100668
C	1.5547323	-0.3930134	-0.0069409
N	1.7101793	-1.5144816	-0.8341511
N	2.8522158	0.1544769	-0.0338346
C	3.0042157	-1.6130832	-1.3636454
C	0.8015389	-2.6095799	-1.0499943
C	3.7117783	-0.5621409	-0.8701711
C	3.3985277	1.2081818	0.7868227
H	3.3011171	-2.4335090	-2.0221505
C	0.4629983	-3.4612838	0.0371631
C	0.4301353	-2.9369788	-2.3779346
H	4.7480053	-0.2542165	-1.0344331
C	3.0206771	2.5554111	0.5676930
C	4.3943633	0.8747063	1.7400692
C	-0.2358894	-4.6472740	-0.2456228
C	0.8666425	-3.1329831	1.4502156

C	-0.2731039	-4.1392316	-2.6005452
C	0.7902514	-2.0612567	-3.5572507
C	3.6367631	3.5575479	1.3374954
C	1.9824992	2.8901430	-0.4590930
C	4.9889545	1.9200842	2.4773284
C	4.8270019	-0.5524318	1.9940279
C	-0.6003432	-5.0184760	-1.5553038
H	-0.4891834	-5.3143040	0.5942039
H	0.5759179	-3.9440724	2.1444939
H	1.9636616	-2.9799308	1.5305533
H	0.3987183	-2.1793754	1.7809418
H	-0.5617848	-4.3966217	-3.6332912
H	0.0091817	-2.1221351	-4.3410048
H	0.9107049	-1.0020381	-3.2655546
H	1.7448295	-2.3775992	-4.0308498
C	4.6245889	3.2660108	2.2987802
H	3.3368220	4.6049212	1.1686023
H	1.0138199	2.4145306	-0.1762502
H	1.8299907	3.9811949	-0.5529857
H	2.2466848	2.4813037	-1.4557182
H	5.7574827	1.6642597	3.2254141
H	3.9832881	-1.2615345	1.8924033
H	5.6121349	-0.8841842	1.2816629
H	5.2473497	-0.6563693	3.0134115
C	-1.2999958	-6.3318977	-1.8207060
C	5.2521310	4.3671501	3.1228422
H	-1.6970590	-6.3850230	-2.8535682
H	-0.6061825	-7.1900356	-1.6876756
H	-2.1443897	-6.4946603	-1.1197017
H	4.5423390	4.7500503	3.8876600
H	6.1567647	4.0158025	3.6570038
H	5.5412390	5.2335476	2.4928844
C	-1.6395279	0.7633600	0.1068206

N	-2.8164965	0.0441826	0.3715554
N	-2.1552160	1.9315277	-0.4820401
C	-3.9669455	0.7469057	-0.0044215
C	-2.9605588	-1.3483368	0.7348920
C	-3.5554090	1.9284437	-0.5392555
C	-1.4314338	3.0011339	-1.1178826
H	-4.9681205	0.3277156	0.1327429
C	-3.1455312	-2.2764757	-0.3217788
C	-3.1275469	-1.7386705	2.0866173
H	-4.1203304	2.7581948	-0.9732195
C	-1.5117159	4.3133481	-0.5904389
C	-0.7728716	2.7507728	-2.3504482
C	-3.4765074	-3.6052958	0.0004278
C	-3.0241593	-1.8433398	-1.7623298
C	-3.4408747	-3.0866437	2.3538537
C	-3.0025339	-0.7564091	3.2192193
C	-0.8879890	5.3596531	-1.3018186
C	-2.2654263	4.6191186	0.6814462
C	-0.1692552	3.8294148	-3.0192884
C	-0.7267833	1.3628596	-2.9317677
C	-3.6248447	-4.0356262	1.3318314
H	-3.6333894	-4.3215908	-0.8218211
H	-3.1165497	-2.7083527	-2.4454851
H	-3.8043866	-1.1011792	-2.0350578
H	-2.0433634	-1.3611995	-1.9449334
H	-3.5727758	-3.3931487	3.4047093
H	-3.3222239	-1.2101378	4.1769225
H	-1.9504073	-0.4148884	3.3172777
H	-3.6147287	0.1510126	3.0424900
C	-0.2108168	5.1447214	-2.5144575
H	-0.9418014	6.3803408	-0.8883286
H	-2.1824646	3.7959419	1.4131849
H	-1.8862051	5.5478490	1.1509126

H	-3.3495576	4.7713255	0.4886253
H	0.3426345	3.6337648	-3.9761096
H	-0.1982363	0.6737605	-2.2397667
H	-1.7471450	0.9467671	-3.0642460
H	-0.2195011	1.3580601	-3.9154723
C	-3.9579716	-5.4741307	1.6565490
C	0.4656003	6.2776878	-3.2519278
H	-4.5572243	-5.5567202	2.5854946
H	-4.5261950	-5.9582503	0.8371568
H	-3.0357609	-6.0754783	1.8139456
H	1.5717961	6.2061925	-3.1718797
H	0.2222166	6.2615013	-4.3343480
H	0.1669819	7.2653662	-2.8492956
C	-1.2513083	2.1834567	3.3206918
H	-1.2983791	1.9758029	4.4138687
H	-0.8954342	3.2312156	3.2118646
H	-2.2894453	2.1573079	2.9294464
C	1.7348063	0.0282836	3.7158837
H	2.0366035	-1.0242119	3.5333882
H	2.6610591	0.6393922	3.6637301
H	1.3575837	0.0931064	4.7615366

[Ni(NHC)(η^2-MeC≡CMe)]

Energy = -2588.115054243

Ni	0.2745316	0.1168232	0.5892299
C	0.3845024	0.1208300	2.4861940
C	0.7088109	1.2744470	2.0357583
C	-0.0439664	-0.5115797	-1.1466814
N	-0.5247238	-1.7537835	-1.5416277
N	0.1223276	0.1557593	-2.3528977
C	-0.6474041	-1.8441416	-2.9322750
C	-0.8529722	-2.8210003	-0.6340447
C	-0.2419244	-0.6419111	-3.4426449

C	0.6110312	1.5042963	-2.4703691
H	-1.0194493	-2.7466248	-3.4262392
C	0.0604602	-3.8910982	-0.4749210
C	-2.0908415	-2.7904796	0.0530013
H	-0.1776095	-0.2824170	-4.4739101
C	-0.3180805	2.5640837	-2.5880687
C	2.0092816	1.7317302	-2.4937997
C	-0.2984570	-4.9457549	0.3857219
C	1.3917653	-3.8869350	-1.1879388
C	-2.3977616	-3.8682619	0.9058308
C	-3.0381143	-1.6279814	-0.1063783
C	0.1856066	3.8707444	-2.7439664
C	-1.8035192	2.3031776	-2.5193151
C	2.4590026	3.0555361	-2.6485701
C	2.9809624	0.5888924	-2.3311822
C	-1.5203591	-4.9554090	1.0862592
H	0.4061719	-5.7835333	0.5166970
H	2.0192260	-4.7390946	-0.8636874
H	1.2765732	-3.9512153	-2.2905038
H	1.9448728	-2.9470375	-0.9816415
H	-3.3603480	-3.8567043	1.4431479
H	-3.9900363	-1.8098563	0.4282118
H	-2.5734687	-0.7000785	0.2959267
H	-3.2683371	-1.4279685	-1.1733358
C	1.5666497	4.1393891	-2.7787877
H	-0.5289469	4.7051450	-2.8359765
H	-2.0672827	1.8014637	-1.5643915
H	-2.3779402	3.2467725	-2.5881854
H	-2.1470276	1.6331011	-3.3351120
H	3.5451902	3.2440370	-2.6691635
H	2.8596304	0.1203691	-1.3301751
H	2.8046724	-0.2118491	-3.0791230
H	4.0271691	0.9351232	-2.4339926

C	-1.8654688	-6.0839281	2.0312946
C	2.0887146	5.5495543	-2.9363684
H	-2.9612973	-6.2228103	2.1202164
H	-1.4219406	-7.0447394	1.7020236
H	-1.4787621	-5.8810265	3.0538128
H	2.6055118	5.8924333	-2.0145792
H	2.8278382	5.6188564	-3.7614782
H	1.2728649	6.2675563	-3.1489422
C	1.1231805	2.6945104	2.1120886
H	1.5784701	2.9331211	3.0982918
H	1.8579388	2.9530242	1.3224674
H	0.2527438	3.3702203	1.9705325
C	0.1464640	-0.9191847	3.5140356
H	-0.5947192	-1.6729285	3.1794790
H	1.0846648	-1.4690729	3.7437372
H	-0.2199713	-0.4724908	4.4644157

[Ni(Mes$_2$Im)(η^2-MeC≡Me)$_2$]

Energy = -2744.002033256

Ni	-0.0001492	0.3537989	-0.0003403
N	-1.0856366	-2.4334373	0.0533291
N	1.0861828	-2.4330799	-0.0523171
C	0.0001193	-1.5612241	0.0002454
C	-0.6794654	-3.7720890	0.0396034
H	-1.3948163	-4.5966306	0.0942219
C	0.6804986	-3.7718674	-0.0378336
H	1.3961563	-4.5961758	-0.0919550
C	-2.4845693	-2.0801779	0.0643011
C	-3.0387574	-1.3978394	1.1790725
C	-4.4134472	-1.0983381	1.1564298
H	-4.8444644	-0.5642955	2.0191173
C	-5.2507883	-1.4675344	0.0867903
C	-4.6757216	-2.1841559	-0.9768288

H	-5.3127990	-2.5045368	-1.8176527
C	-3.3046506	-2.5101145	-1.0119397
C	-2.2063152	-1.0150523	2.3729103
H	-1.5629229	-1.8554600	2.7045295
H	-2.8487960	-0.7097859	3.2209225
H	-1.5207798	-0.1759988	2.1198330
C	-6.7148891	-1.0930852	0.0817245
H	-7.2964548	-1.7171604	-0.6250543
H	-6.8537093	-0.0329191	-0.2223502
H	-7.1671863	-1.2000492	1.0885381
C	-2.7785460	-3.3283113	-2.1718145
H	-2.8464966	-4.4179286	-1.9631554
H	-1.7200093	-3.1087315	-2.4015862
H	-3.3769826	-3.1399203	-3.0847070
C	2.4849853	-2.0793034	-0.0634463
C	3.0389459	-1.3974117	-1.1786051
C	4.4135185	-1.0973728	-1.1560874
H	4.8443761	-0.5636729	-2.0190663
C	5.2509625	-1.4656340	-0.0862024
C	4.6761346	-2.1818378	0.9778244
H	5.3133038	-2.5014516	1.8188704
C	3.3051860	-2.5083034	1.0130721
C	2.2063975	-1.0156549	-2.3726975
H	2.8487944	-0.7107413	-3.2208994
H	1.5205997	-0.1766496	-2.1201790
H	1.5632557	-1.8564686	-2.7037698
C	6.7149339	-1.0906729	-0.0813326
H	7.2967031	-1.7141329	0.6258223
H	6.8533803	-0.0302816	0.2221261
H	7.1672871	-1.1980692	-1.0880749
C	2.7793305	-3.3260221	2.1734017
H	1.7206716	-3.1067778	2.4029226
H	3.3775863	-3.1367787	3.0862380

H	2.8478202	-4.4157363	1.9654267
C	-0.8616293	0.5778614	-1.7522552
C	-0.5955100	1.7353647	-1.2695904
C	0.8611569	0.5791991	1.7515138
C	0.5948482	1.7363275	1.2680608
C	-0.6501782	3.2025236	-1.4765971
H	0.3544302	3.6705109	-1.4255018
H	-1.0819974	3.4429321	-2.4728146
H	-1.2752268	3.7097651	-0.7126838
C	-1.4227345	-0.1897559	-2.8876291
H	-0.7898229	-1.0573506	-3.1679718
H	-2.4277423	-0.5901907	-2.6393312
H	-1.5333262	0.4517531	-3.7896766
C	0.6491219	3.2036350	1.4740974
H	-0.3556819	3.6712266	1.4231406
H	1.0812858	3.4448268	2.4699758
H	1.2736536	3.7106180	0.7095964
C	1.4224133	-0.1875840	2.8873782
H	0.7897275	-1.0551867	3.1682122
H	2.4275369	-0.5878945	2.6393523
H	1.5327985	0.4544921	3.7890467

[(Mes$_2$Im)Ni(CO)$_3$]

Energy = -2773.443313274

Ni	-0.8479312	-0.3144910	-0.0695931
C	-0.1789302	-1.9803283	0.0348698
C	-2.6501067	-0.4105927	-0.0746047
C	-0.3414983	0.6128537	1.3926818
O	0.2209195	-3.0610047	0.1536969
O	-0.0782017	1.1396357	2.3884813
O	-3.7977979	-0.5136459	0.0276331
C	-0.1797337	0.5568614	-1.7246224
N	-0.7518673	0.5735854	-2.9761789

N	0.9866235	1.2635343	-1.9099646
C	0.0311595	1.2647495	-3.8974949
C	-2.0134877	-0.0251931	-3.3344817
C	1.1276345	1.6999948	-3.2249485
C	1.9673306	1.5527132	-0.8933395
H	-0.2635758	1.3776990	-4.9335462
C	-2.0340777	-1.3553801	-3.7869157
C	-3.1776960	0.7604177	-3.2737233
H	1.9873445	2.2712941	-3.5528790
C	1.8626437	2.7555114	-0.1732636
C	3.0287830	0.6538289	-0.6933950
C	-3.2681292	-1.8961627	-4.1673068
C	-0.7758801	-2.1796461	-3.8599663
C	-4.3844571	0.1718602	-3.6678155
C	-3.1379789	2.1842527	-2.7837083
C	2.8491985	3.0351730	0.7788550
C	0.7207739	3.7104716	-0.4034982
C	3.9879104	0.9811308	0.2724989
C	3.1387803	-0.6211269	-1.4874242
C	-4.4536530	-1.1541590	-4.1111969
H	-3.3000772	-2.9309594	-4.5171385
H	-0.9919409	-3.1816033	-4.2507955
H	-0.0206844	-1.7146294	-4.5114847
H	-0.3181310	-2.2898543	-2.8662500
H	-5.2984952	0.7686259	-3.6178750
H	-4.1426360	2.6244172	-2.7936107
H	-2.7497225	2.2357604	-1.7556507
H	-2.4833966	2.8135327	-3.4053895
C	3.9150271	2.1608446	1.0222329
H	2.7754785	3.9628802	1.3515220
H	-0.2465132	3.2201790	-0.2195981
H	0.7997840	4.5757387	0.2660976
H	0.7003118	4.0814871	-1.4393894

H	4.8166441	0.2891917	0.4420874
H	2.2590503	-1.2618676	-1.3313469
H	3.2042746	-0.4236596	-2.5681279
H	4.0309469	-1.1858970	-1.1903121
C	-5.7750174	-1.7751044	-4.4874269
C	4.9393480	2.4681495	2.0850146
H	-6.4545746	-1.0361911	-4.9337739
H	-5.6438110	-2.5990206	-5.2017575
H	-6.2790679	-2.1872954	-3.5988409
H	4.5996461	2.1092283	3.0695581
H	5.8997771	1.9799575	1.8715683
H	5.1140796	3.5490531	2.1751375

[(^{i}Pr$_2$Im^{Me})Ni(CO)$_3$]

Energy = -2311.157495022

Ni	-0.8920932	-0.5027551	0.0275943
C	-0.7331029	-2.2678467	-0.2996731
C	-2.6071963	-0.2013619	0.4899843
C	0.1475197	0.0621716	1.3870707
O	-0.6652792	-3.4082065	-0.4845184
O	0.7862670	0.4221626	2.2822184
O	-3.6891328	-0.0947084	0.8903882
C	-0.2643077	0.4597405	-1.6170629
N	-0.9195604	1.3460363	-2.4361238
N	0.9802428	0.3578002	-2.1893097
C	-0.1086545	1.7730937	-3.4785106
C	-2.3107081	1.8007974	-2.2546536
C	1.0892014	1.1502881	-3.3226311
C	2.0777188	-0.4833680	-1.6719592
H	-2.6342342	1.2964495	-1.3361947
H	1.6487578	-0.9545673	-0.7774058
C	-2.3673239	3.3146048	-2.0352379
H	-2.0459418	3.8690042	-2.9294743

H	-3.3985757	3.6175630	-1.8084175
H	-1.7284589	3.6129851	-1.1934635
C	-3.1933787	1.3338424	-3.4148214
H	-4.2393906	1.6065431	-3.2192476
H	-2.8970697	1.8024607	-4.3651394
H	-3.1387818	0.2435839	-3.5336919
C	3.2746692	0.3764198	-1.2574958
H	4.0434237	-0.2566913	-0.7938597
H	3.7328417	0.8789973	-2.1223023
H	2.9762414	1.1421127	-0.5292230
C	2.4514203	-1.5728319	-2.6803090
H	2.8742220	-1.1450331	-3.6015425
H	3.2079406	-2.2391882	-2.2439377
H	1.5754851	-2.1779141	-2.9487991
H	-0.4403674	2.4736301	-4.2344880
H	1.9918062	1.2094856	-3.9176626

[1] J. Attner, U. Radius, *Chem.* **2001**, *7*, 783-790.

[2] a) L. Hintermann, *Beilstein. J. Org. Chem.* **2007**, *3*, 22; b) A. J. Arduengo, H. V. R. Dias, R. L. Harlow, M. Kline, *J. Am. Chem. Soc.* **1992**, *114*, 5530-5534.

[3] T. Schaub, Radius, U., Brucks, A., Choules, M. P., Olsen, M. T., & Rauchfuss, T. B. , *Inorg. Synth.* **2010**, *35*, 78-91.

[4] a) R. A. Schunn, S. D. Ittel, M. A. Cushing, R. Baker, R. J. Gilbert, D. P. Madden, *Inorg. Synth.* **1990**, *28*, 94-98; b) J. W. Wielandt, D. Ruckerbauer, T. Zell, U. Radius, *Inorg. Synth.* **2010**, *35*, 120-125.

[5] A. J. Arduengo, S. F. Gamper, J. C. Calabrese, F. Davidson, *J. Am. Chem. Soc.* **1994**, *116*, 4391-4394.

[6] G. Sheldrick, *Acta Crystallogr., Sect. A,* **2015**, *71*, 3-8.

[7] a) Furche, F.; Ahlrichs, R.; Hättig, C.; Klopper, W.; Sierka, M.; Weigend, F., Turbomole, *WIREs Comput. Mol. Sci.* **2014**, *4*, 91-100. b) Ahlrichs, R.; M. Bär, Häser, M.; Horn, H.; Kölmel, C., Electronic structure calculations on workstation computers: The program system Turbomole, *Chem. Phys. Lett.* **1989**, *162*, 165-169.

[8] a) Häser, M.; Ahlrichs, R., Improvements on the direct SCF method, *J. Comput. Chem.* **1989**, 10, 104-111. b) Treutler O.; Ahlrichs, R., Efficient molecular numerical integration schemes, *J. Chem. Phys. C.* **1995**, *102*, 346-354. c) Sierka, M.; Hogekamp A.; Ahlrichs, R., Fast evaluation of the Coulomb potential for electron densities using multipole accelerated resolution of identity approximation, *J. Chem. Phys.* **2003**, *118*, 9136-9148.

[9] a) Becke, A. D., Density-functional exchange-energy approximation with correct asymptotic behavior, *Phys. Rev. A* **1988**, *38*, 3098-3100. b) Perdew, J. P., Density-functional approximation for the correlation energy of the inhomogeneous electron gas, *Phys. Rev. B* **1986**, *33*, 8822-8824; *erratum*: Perdew, J. P. *Phys. Rev. B* **1986**, *34*, 7406.

[10] a) Schäfer, A.; Huber, C.; Ahlrichs, R., Fully optimized contracted Gaussian basis sets of triple zeta valence quality for atoms Li to Kr, *J. Chem. Phys.* **1994**, *100*, 5829-5835. b) Eichkorn, K.; Weigend, F.; Treutler, O.; Ahlrichs, R., Auxiliary basis sets for main row atoms and transition metals and their use to approximate Coulomb potentials, *Theor. Chem. Acc.* **1997**, *97*, 119-124. c) Eichkorn, K.; Treutler, O.; Öhm, H.; Häser M.; Ahlrichs; R., Auxiliary basis sets to approximate Coulomb potentials, *Chem. Phys. Letters* **1995**, *242*, 652-660. d) Weigend, F.; Ahlrichs, R., Balanced basis sets of split valence, triple zeta valence and quadruple zeta valence quality for H to Rn: Design and assessment of accuracy, *Phys. Chem. Chem. Phys.* **2005**, *7*, 3297-3305.

[11] Deglmann, P.; May, K.; Furche F.; Ahlrichs, R., Nuclear second analytical derivative calculations using auxiliary basis set expansions, *Chem. Phys. Letters* **2004**, *384*, 103-107.

[12] Reed, A. E.; Weinstock, R. B.; Weinhold, F., Natural population analysis, *J. Chem. Phys.* **1985**, 83, 735-746.

[13] NBO 6.0. E. D. Glendening, J. K. Badenhoop, A. E. Reed, J. E. Carpenter, J. A. Bohmann, C. M. Morales, C. R. Landis, and F. Weinhold (Theoretical Chemistry Institute, University of Wisconsin, Madison, WI, 2013); http://nbo6.chem.wisc.edu/.

[14] Wiberg, K. A., Application of the Pople-Santry-Segal CNDO Method to the Cyclopropylcarbinyl and Cyclobutyl Cation and to Bicyclobutane, *Tetrahedron* **1968**, *24*, 1083-1096.

[15] A. Poater, F. Ragone, S. Giudice, C. Costabile, R. Dorta, S. P. Nolan, L. Cavallo, *Organometallics* **2008**, *27*, 2679-2681.

[16] A. Poater, F. Ragone, R. Mariz, R. Dorta, L. Cavallo, **2010**, *16*, 14348-14353.

[17] A. Poater, B. Cosenza, A. Correa, S. Giudice, F. Ragone, V. Scarano, L. Cavallo, *Eur. J. Inorg. Chem.* **2009**, *2009*, 1759-1766.
